# Discovery of novel West Nile Virus protease inhibitor based on isobenzonafuranone and triazolic derivatives of eugenol and indan-1,3-dione scaffolds

**DOI:** 10.1371/journal.pone.0223017

**Published:** 2019-09-26

**Authors:** André S. de Oliveira, Poliana A. R. Gazolla, Ana Flávia C. da S. Oliveira, Wagner L. Pereira, Lívia C. de S. Viol, Angélica F. da S. Maia, Edjon G. Santos, Ítalo E. P. da Silva, Tiago A. de Oliveira Mendes, Adalberto M. da Silva, Roberto S. Dias, Cynthia C. da Silva, Marcelo D. Polêto, Róbson R. Teixeira, Sergio O. de Paula

**Affiliations:** 1 Departamento de Biologia Geral, Universidade Federal de Viçosa, Viçosa, MG, Brazil; 2 Instituto Federal de Educação, Ciência e Tecnologia do Norte de Minas Gerais, Fazenda Biribiri, MG, Brazil; 3 Departamento de Bioquímica Biologia Molecular, Universidade Federal de Viçosa, Viçosa, MG, Brazil; 4 Departamento de Química, Universidade Federal de Viçosa, Viçosa, MG, Brazil; 5 Instituto Federal de Educação, Ciência e Tecnologia Catarinense, Araquari, SC, Brazil; Katholieke Universiteit Leuven Rega Institute for Medical Research, BELGIUM

## Abstract

The West Nile Virus (WNV) NS2B-NS3 protease is an attractive target for the development of therapeutics against this arboviral pathogen. In the present investigation, the screening of a small library of fifty-eight synthetic compounds against the NS2-NB3 protease of WNV is described. The following groups of compounds were evaluated: 3-(2-aryl-2-oxoethyl)isobenzofuran-1(3*H*)-ones; eugenol derivatives bearing 1,2,3-triazolic functionalities; and indan-1,3-diones with 1,2,3-triazolic functionalities. The most promising of these was a eugenol derivative, namely 4-(3-(4-allyl-2-methoxyphenoxy)-propyl)-1-(2-bromobenzyl)-1*H*-1,2,3-triazole (**35**), which inhibited the protease with IC_50_ of 6.86 *μ*mol L^-1^. Enzyme kinetic assays showed that this derivative of eugenol presents competitive inhibition behaviour. Molecular docking calculations predicted a recognition pattern involving the residues His^51^ and Ser^135^, which are members of the catalytic triad of the WNV NS2B-NS3 protease.

## Introduction

The West Nile Virus (WNV) is a member of the same family as the Dengue virus (DENV), Zika virus (ZIKV) and Yellow Fever virus (YFV), the *Flaviviridae* family, *Flavivirus* genus. They are arboviruses that present RNA as a genome [1]. Diseases caused by *Flavivirus* are the major causes of fatality in poverty-stricken regions across Africa, Asia and some parts of the Americas. The combined potential health risk associated with arthropod-borne viruses like DENV, WNV, and ZIKV is enormous. These arboviruses are either emerging or re-emerging in many regions [2].

Three WNV strains are known to be capable of causing unforeseen and large epidemics, leading to serious public health problems. Since 2004, lineages 1 and 3 have been circulating in Europe and, since 2010, beginning in a major epidemic in Greece, lineage 2 has been circulating in several European countries. [3, 4]. The WNV crossed the Atlantic and reached the Western Hemisphere in 1999, when a group of patients with encephalitis was reported in the New York City metropolitan area. Within three years, the virus spread to Canada and Mexico, followed by animal cases in Central and South America [5, 6]. Recently, the first human case of WNV was reported in Brazil, with the development of encephalitis. It is possible that sporadic cases or small groups of the WNV disease had already occurred in different regions of the country without being properly diagnosed [7].

WNV is a genetically and geographically diverse virus. Four or five distinct WNV genetic lines have been proposed, based on phylogenetic analyses of published isolates. Their genomes differ from each other by about 20–25%, and are well correlated with the geographic point of isolation [8–10]. They are enveloped viruses whose genome consists of single-stranded, positive-polarity RNA approximately 11 kb. This RNA contains a single open reading frame encoding a precursor polyprotein, which is processed by viral and host proteases, giving rise to three structural proteins: capsidial protein (C), envelope glycoprotein (E) and pre-membrane/membrane protein (prM/M); and seven non-structural proteins, NS1, NS2A, NS2B, NS3, NS4A, NS4B and NS5, which are involved in the replicative cycle of the virus[11]. Viral protease performs the cleavage of some sites: NS2A-NS2B, NS2B-NS3, NS3-NS4A and NS4B-NS5. It also cleaves the signal sequences at the C-prM position and the NS4A-NS4B, within NS2A, and within the NS3 itself [12, 13].

Despite the tremendous efforts invested in *Flavivirus* research, no clinically approved antiviral chemotherapeutics are available for humans, and disease treatment is limited to supportive care [13]. Inhibition of viral enzymes has proved to be one important approach toward the development of antiviral therapies [2, 13–15]. Non-structural proteins encoded by these RNA viruses are essential for their replication and maturation, and thus may offer ideal targets for developing antiviral drugs [2]. *Flavivirus* genomes are translated into a single polyprotein that needs to be cleaved by viral and host proteases. Because it processes most of the polyprotein cleavages, viral protease is necessary and essential for virus replication [16, 17].

**Fig 1:**
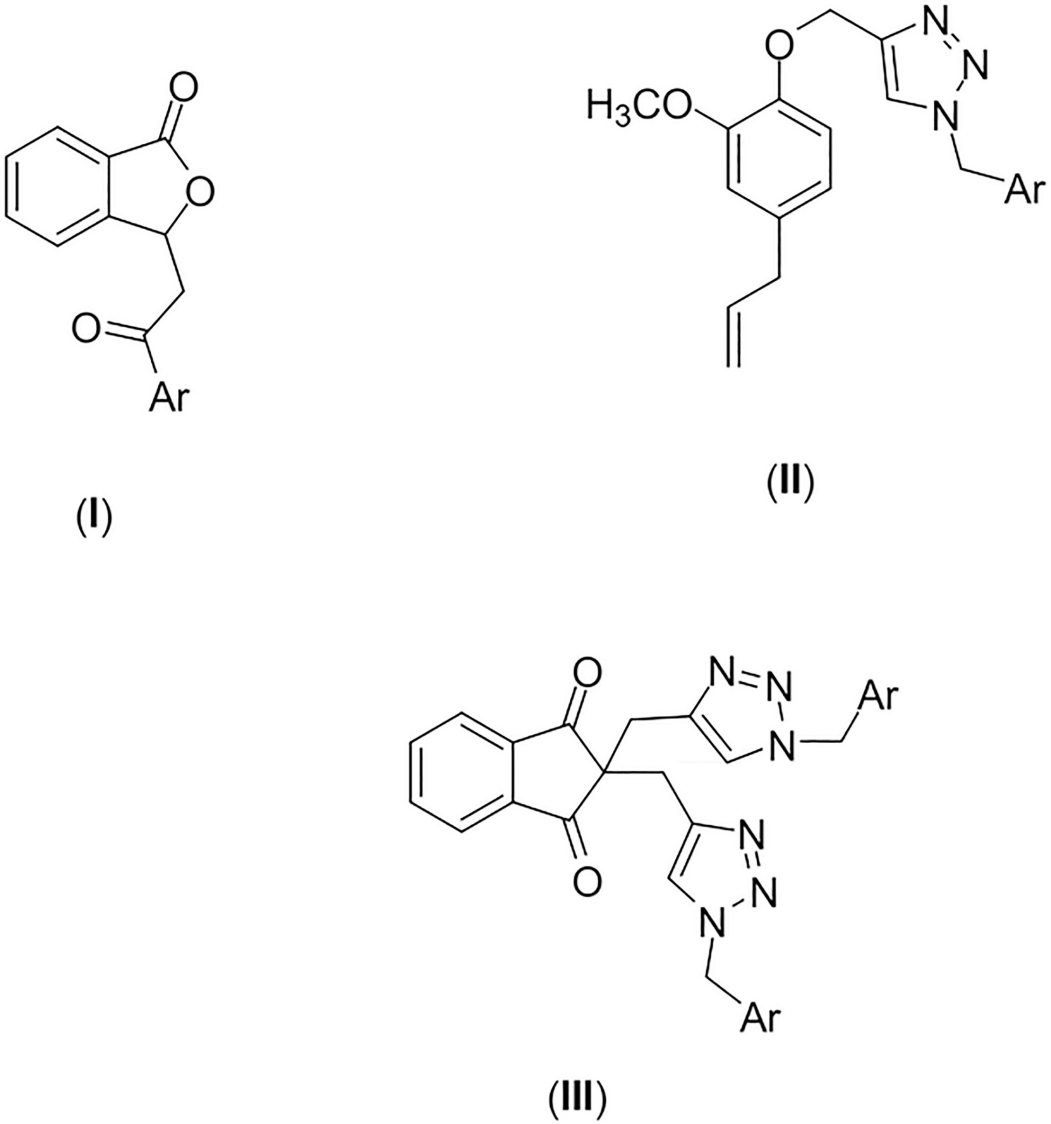
General structures of compounds evaluated as inhibitors of WNV NS2-NB3 protease. 3-(2-aryl-2-oxoethyl)isobenzofuran-1(3*H*)-ones (**I**); eugenol derivatives bearing 1,2,3-triazolic functionalities (**II**); indan-1,3-diones with 1,2,3-triazolic functionalities (**III**).

Considering the premises, the screening of a small library of fifty-eight synthetic compounds against the NS2-NB3 protease of WNV is described in the present investigation. The following groups of compounds were evaluated: (**I**) 3-(2-aryl-2-oxoethyl)isobenzofuran-1(3*H*)-ones; (**II**) eugenol derivatives bearing 1,2,3-triazolic functionalities; and (**III**) indan-1,3-diones with 1,2,3-triazolic functionalities. Fig 1 displays the general structures of the evaluated compounds.

The isobenzofuran-1-(3*H*)-ones, 1,2,3-triazolic derivatives, eugenol, and indan-1,3-diones are substances that have been described in the literature as being endowed with antiviral activities [8, 18–28]. Isobenzofuran-1-(3*H*) -one derivatives have several biological activities [18, 29–35], highlighting antiviral action for HIV[18]. Indan-1,3-diones derivatives have been specifically associated with antiviral activity. Studies have indicated efficient action of indan-1,3-diones against the enzyme integrase of the HIV-1 virus [36]; against the structure of human papillomavirus (HPV) [37–39]; and also against the Hepatitis C virus (HCV) protease [40], and recently for WNV protease [41]. Eugenol has been tested against the Herpes virus (HSV), HSV-1 (five viral isolates) and HSV-2 (five viral isolates), providing complete protection against one isolate of each type, HSV-1 and 2, and protection between 16.5% and 87.7% for the remaining isolates [21–23]. The triazole ring was associated with the molecules due to the antimicrobial activity already described for 1,2,3-triazole compounds, especially studies that demonstrated antiviral action against the Dengue virus [19, 20]. This fact prompted the authors to evaluate the effect of the compounds presenting general structures (**I**), (**II**) and (**III**) on the NS2-NB3 WNV protease.

## Materials and methods

### Synthesis

Solvents were purchased from Vetec (Rio de Janeiro, Brazil). Benzyl alcohols, pent-4-yn-1-ol, methanesulfonyl chloride, sodium azide, triethylamine, propargyl bromide, acetophenones, and indan-1,3-dione were procured from Sigma Aldrich (St. Louis, MO, United States) and used as received. Eugenol was extracted via hydrodistillation from cloves purchased in the local market in Viçosa, Minas Gerais state, Brazil, and subsequently purified by column chromatography (*vide infra*). ^1^H- and ^13^C-NMR spectra were recorded on a Varian Mercury 300 MNR Spectrometer (Varian, Palo Alto, CA, United States) at 300 MHz and 75 MHz, respectively, using CDCl_3_, C_6_D_6_ or DMSO-*d*_*6*_ as solvents. NMR data are presented as follows: chemical shift (*δ*) in ppm, multiplicity, the number of protons, and *J* values in Hertz (Hz). Multiplicities are shown as the following abbreviations: s (singlet), brs (broad singlet), d (doublet), d_ap_ (apparent doublet), dd (doublet of a doublets), t (triplet), brd (broad doublet), ddt_ap_ (apparent doublet of doublets of triplets), q (quartet), quint (quintet), and m (multiplet). Some signals in the ^13^C NMR spectra were described as multiplets due to the ^19^F-^13^C coupling. IR spectra were obtained using a Varian 660-IR equipped with GladiATR (Varian, Palo Alto, CA, USA) scanning from 4000 to 500 cm^−1^. Analytical thin-layer chromatography analysis was conducted on aluminum-backed, pre-coated silica gel plates using different solvent systems. TLC plates were visualized using potassium permanganate solution, phosphomolybdic acid solution and/or UV light. Flash column chromatography was performed using silica gel 60 (60–230 mesh). Melting points were determined using a MQAPF-302 melting point apparatus (Microquimica, Santa Catarina, Brazil) and are uncorrected. Solvents were dried using standard procedures described in the literature [42].

#### Synthesis of 3-(2-oxo-aryl)-isobenzofuran-1-(3*H*)-ones 1–18

**Fig 2:**
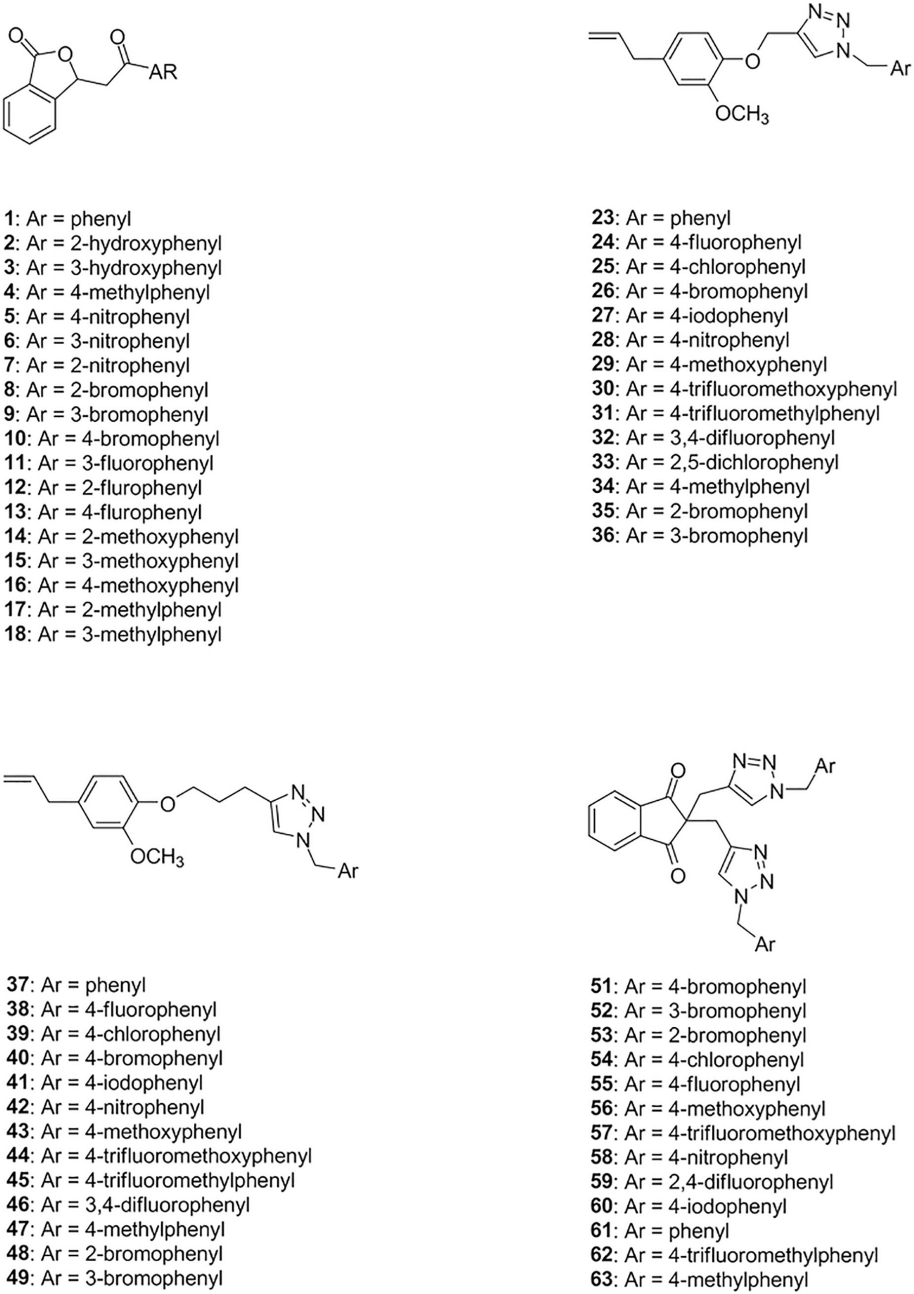
Structures of compounds *1*–*18*, *23*–*49*, and *51*–*63*.

The preparation of compounds **1**–**18** (Fig 2) was carried out via ZrOCl_2_·8H_2_O catalyzed condensation reactions between phthalaldehydic acid and different acetophenones as previously reported [43].

#### Extraction and purification of eugenol (19)

Eugenol (**19**) was extracted via hydrodistillation from dried flower buds of *Eugenia caryophyllata*, commonly known as cloves, purchased in the local market in Viçosa, Minas Gerais state, Brazil. Thus, 60.0 g of cloves were mixed with 500 mL of distilled water in a round-bottom flask which was connected to the hydrodistillation apparatus. The mixture was heated for three hours. The obtained hydrolate was transferred to a separatory funnel and the aqueous layer was extracted with dichloromethane (3 x 30 mL). The organic extracts were combined and the resulting organic layer was dried over anhydrous sodium sulfate, filtered, and concentrated under reduced pressure. The resulting oil was submitted to column chromatography eluted with hexane-ethyl acetate (6:1 v/v). The described procedure afforded 7.12 g of eugenol (**19**), which corresponded to approximately a 12% yield in relation to the initial mass of cloves used in the extraction process.

#### Synthesis of 4-allyl-2-methoxy-1-(prop-2-yn-1-yloxy)benzene (20)

A 50 mL round-bottom flask was charged with eugenol (**19**) (1.20 g, 7.32 mmol), sodium hydroxide (0.313 g, 7.83 mmol) and 25 mL of methanol. The resulting mixture was heated to 40 ^o^C and magnetically stirred for 30 minutes. After this time, methanol was removed under reduced pressure and 10.0 mL of anhydrous ethanol was added for the removal of the residual water. The ethanol was removed under reduced pressure. Then, the round-bottom flask, under a nitrogen atmosphere, was charged with anhydrous acetonitrile (25.0 mL) and propargyl bromide (800 *μ*L, 8.79 mmol) was added slowly. The mixture was magnetically stirred at room temperature for 18 hours. TLC analysis revealed the completion of the reaction after this time. The reaction mixture was concentrated under reduced pressure and the residue was partitioned between 25.0 mL of sodium hydroxide solution (0.1 mol L^-1^) and 25 mL of diethyl ether. The layers were separated and the aqueous phase was extracted with diethyl ether (2 x 25.0 mL). The organic extracts were combined and the resulting organic layer was washed with brine (25.0 mL), dried over anhydrous sodium sulfate, filtered and concentrated under reduced pressure. The residue was purified by column chromatography eluted with hexane-ethyl acetate (6:1 v/v). The described procedure afforded 1.29 g (6.37 mmol, 87% yield) of compound **20**.

#### Synthesis of pent-4-yn-1-yl methanesulfonate (21)

Pent-4-yn-1-ol (1.68 g, 20.0 mmol) and dichloromethane (20 mL) were added to a 100 mL round-bottom flask under nitrogen atmosphere. The mixture was cooled to -50°C and triethylamine (5.60 mL, 40.0 mmol) was added. After that, methanesulfonyl chloride was added slowly (2.32 mL, 30.0 mmol) to the reaction mixture under continuous stirring. The progress of the reaction was monitored by TLC. After completion of it, 10 mL of distilled water were added. The organic phase was washed with 1% HCl solution (3 x 15 mL) followed by saturated aqueous NaHCO_3_ (3 x 5 mL), dried over anhydrous sodium sulfate, and concentrated under reduced pressure. The residue was purified by column chromatography eluted with hexane-ethyl acetate-dicloromethane (3:1:3 v/v) to give compound **21** in 92% yield (3.00 g, 18.0 mmol).

#### Synthesis 4-allyl-2-methoxy-1-(pent-4-yn-1-yloxy)benzene (22)

This compound was prepared using a similar procedure to that described for the preparation of compound **20**. In this case, the alkylating agent corresponded to compound **21**. Substance **22** was obtained in 78% yield (1.64 g, 7.13 mmol) after purification by column chromatography eluted with hexane-ethyl acetate (6:1 v/v).

#### Synthesis of compounds 23–51 exemplified by the synthesis of compound 4-((4-allyl-2-methoxyphenoxy)methyl)-1-benzyl-1*H*-1,2,3-triazole (23)

A 10 mL round bottom flask was charged with 4-allyl-2-methoxy-1-(prop-2-yn-1-yloxy)benzene (**20**) (0.150 g, 0.740 mmol), benzyl azide (0.0990g; 0.740 mmol), sodium ascorbate (0.0590 g, 0.300 mmol), 1.00 mL of etanol and 1.00 mL of distilled water. Then, CuSO_4_·5H_2_O (0.0370 g, 0.150 mmol) was added. The reaction mixture was vigorously stirred at room temperature. After completion of the reaction, as noticed was by TLC analysis, the mixture was extracted with dichloromethane (3 x 10.0 mL). The organic extracts were combined and the resulting organic layer was washed with aqueous sodium carbonate saturated solution, dried over anhydrous sodium sulphate, filtrated and concentrated under reduced pressure. The residue was purified by silica gel column chromatography eluted with hexane-ethyl acetate-dichloromethane (3:1:3 v/v). Compound **23** was obtained in 91% yield (0.228g, 0.680 mmol). Selected IR and NMR spectra is show at A-C Figs in S1 File.

Compounds **24**–**51** were prepared using a procedure similar to that described for the synthesis of **23**. The structures of these compounds were confirmed by NMR (^1^H and ^13^C), and IR analyses and are supported by the following data.

**Data for 4-((4-allyl-2-methoxyphenoxy)methyl)-1-(4-fluorobenzyl)-1*H*-1,2,3-triazole (24)**

White solid, m.p. 92.1–92.7°C, purified by column chromatography eluted with hexane-ethyl acetate-dichloromethane (3:1:3 v/v), TLC: R_f_ = 0.39 (hexane-ethyl acetate-dichloromethane 3:1:3 v/v). Selected IR and NMR spectra is show at D-F Figs in S1 File.

**Data for 4-((4-allyl-2-methoxyphenoxy)methyl)-1-(4-chlorobenzyl)-1*H*-1,2,3-triazole (25)**

White solid, m.p. 112.3–112.6°C, purified by column chromatography eluted with hexane-ethyl acetate-dichloromethane (3:1:3 v/v), TLC: R_f_ = 0.37 (hexane-ethyl acetate-dichloromethane 3:1:3 v/v). Selected IR and NMR spectra is show at G-I Figs in S1 File.

**Data for 4-((4-allyl-2-methoxyphenoxy)methyl)-1-(4-bromobenzyl)-1*H*-1,2,3-triazole (26)**

White solid, m.p. 119.1–120.2°C, purified by column chromatography eluted with hexane-ethyl acetate-dichloromethane (3:1:3 v/v), TLC: R_f_ = 0.59 (hexane-ethyl acetate-dichloromethane 3:1:3 v/v). Selected IR and NMR spectra is show at J-L Figs in S1 File.

**Data for 4-((4-allyl-2-methoxyphenoxy)methyl)-1-(4-iodobenzyl)-1*H*-1,2,3-triazole (27)**

White solid, m.p. 129.2–131.1°C, purified by column chromatography eluted with hexane-ethyl acetate-dichloromethane (3:1:3 v/v), TLC: R_f_ = 0.37 (hexane-ethyl acetate-dichloromethane 3:1:3 v/v). Selected IR and NMR spectra is show at M-O Figs in S1 File.

**Data for 4-((4-allyl-2-methoxyphenoxy)methyl)-1-(4-nitrobenzyl)-1*H*-1,2,3-triazole (28)**

White solid, m.p. 120.9–121.9°C, purified by column chromatography eluted with hexane-ethyl acetate-dichloromethane (3:1:3 v/v) TLC: R_f_ = 0.14 (hexane-ethyl acetate-dichloromethane 3:1:3 v/v). Selected IR and NMR spectra is show at P-R Figs in S1 File.

**Data for 4-((4-allyl-2-methoxyphenoxy)methyl)-1-(4-methoxybenzyl)-1*H*-1,2,3-triazole (29)**

White solid, m.p. 92.4–93.1°C, purified by column chromatography eluted with hexane-ethyl acetate-dichloromethane (3:1:3 v/v), TLC: R_f_ = 0.36 (hexane-ethyl acetate-dichloromethane 3:1:3 v/v). Selected IR and NMR spectra is show at S-U Figs in S1 File.

**Data for 4-((4-allyl-2-methoxyphenoxy)methyl)-1-(4-(trifluoromethoxy)benzyl)-1*H*-1,2,3-triazole (30)**

White solid, m.p. 126.4–127.3°C, purified by column chromatography eluted with hexane-ethyl acetate-dichloromethane (3:1:3 v/v), TLC: R_f_ = 0.49 (hexane-ethyl acetate-dichloromethane 3:1:3 v/v). Selected IR and NMR spectra is show at V-W Figs in S1 File.

**Data for 4-((4-allyl-2-methoxyphenoxy)methyl)-1-(4-(trifluoromethyl)benzyl)-1*H*-1,2,3-triazole (31)**

White solid, m.p. 142.7–143.0°C, purified by column chromatography eluted with hexane-ethyl acetate-dichloromethane (3:1:3 v/v), TLC: R_f_ = 0.43 (hexane-ethyl acetate-dichloromethane 3:1:3 v/v). Selected IR and NMR spectra is show at Y-AA Figs in S1 File.

**Data for 4-((4-allyl-2-methoxyphenoxy)methyl)-1-(3,4-difluorobenzyl)-1H-1,2,3-triazole (32)**

White solid, m.p. 104.9–105.2°C, purified by column chromatography eluted with hexane-ethyl acetate-dichloromethane (3:1:3 v/v), TLC: R_f_ = 0.30 (hexane-ethyl acetate-dichloromethane 3:1:3 v/v). Selected IR and NMR spectra is show at AB-AD Figs in S1 File.

**Data for 4-((4-allyl-2-methoxyphenoxy)methyl)-1-(2,5-dichlorobenzyl)-1*H*-1,2,3-triazole (33)**

White solid, m.p. 87.9–88.4°C, purified by column chromatography eluted with hexane-ethyl acetate-dichloromethane (3:1:3 v/v), TLC: R_f_ = 0.59 (hexane-ethyl acetate-dichloromethane 3:1:3 v/v). Selected IR and NMR spectra is show at AE-AG Figs in S1 File.

**Data for 4-((4-allyl-2-methoxyphenoxy)methyl)-1-(4-methylbenzyl)-1*H*-1,2,3-triazole (34)**

White solid, m.p. 81.4–82,3°C, purified by column chromatography eluted with hexane-ethyl acetate-dichloromethane (3:1:3 v/v), TLC: R_f_ = 0.66 (hexane-ethyl acetate-dichloromethane 3:1:3 v/v). Selected IR and NMR spectra is show at AH-AJ Figs in S1 File.

**Data for 4-((4-allyl-2-methoxyphenoxy)methyl)-1-(2-bromobenzyl)-1*H*-1,2,3-triazole (35)**

White solid, m.p. 67.5–68.6°C, TLC: R_f_ = 0.70 (hexane-ethyl acetate-dichloromethane 3:1:3 v/v). Selected IR and NMR spectra is show at AK-AM Figs in S1 File.

**Data for 4-((4-allyl-2-methoxyphenoxy)methyl)-1-(3-bromobenzyl)-1*H*-1,2,3-triazole (36)**

White solid, m.p. 97.5–97.9°C, purified by column chromatography eluted with hexane-ethyl acetate-dichloromethane (3:1:3 v/v), TLC: R_f_ = 0.70 (hexane-ethyl acetate-dichloromethane 3:1:3 v/v). Selected IR and NMR spectra is show at AN-AP Figs in S1 File.

**Data for 4-(3-(4-allyl-2-methoxyphenoxy)propyl)-1-benzyl-1*H*-1,2,3-triazole (37)**

White solid, m.p. 71.4–72.6°C, purified by column chromatography eluted with hexane-ethyl acetate-dichloromethane- (3:1:3 v/v), TLC: R_f_ = 0.16 (hexane-ethyl acetate-dichloromethane 3:1:3 v/v). Selected IR and NMR spectra is show at AQ-AS Figs in S1 File.

**Data for 4-(3-(4-allyl-2-methoxyphenoxy)propyl)-1-(4-fluorobenzyl)-1*H*-1,2,3-triazole (38)**

White solid, m.p. 96.4–96,8°C, purified by column chromatography eluted with hexane-ethyl acetate-dichloromethane (3:1:3 v/v), TLC: R_f_ = 0.21 (hexane-ethyl acetate-dichloromethane 3:1:3 v/v). Selected IR and NMR spectra is show at AT-AV Figs in S1 File.

**Data for 4-(3-(4-allyl-2-methoxyphenoxy)propyl)-1-(4-chlorobenzyl)-1*H*-1,2,3-triazole (39)**

White solid, m.p. 81.8–82.2°C, purified by column chromatography eluted with hexane-ethyl acetate-dichloromethane (3:1:3 v/v), TLC: R_f_ = 0.46 (hexane-ethyl acetate-dichloromethane 3:1:3 v/v). Selected IR and NMR spectra is show at AX-AY Figs in S1 File.

**Data for 4-(3-(4-allyl-2-methoxyphenoxy)propyl)-1-(4-bromobenzyl)-1*H*-1,2,3-triazole (40)**

White solid, m.p. 89.4–90.3°C, purified by column chromatography eluted with hexane-ethyl acetate-dichloromethane (3:1:3 v/v), TLC: R_f_ = 0.46 (hexane-ethyl acetate-dichloromethane 3:1:3 v/v). Selected IR and NMR spectra is show at AZ-BB Figs in S1 File.

**Data for 4-(3-(4-allyl-2-methoxyphenoxy)propyl)-1-(4-iodobenzyl)-1*H*-1,2,3-triazole (41)**

White solid, m.p. 98.3–99.2°C, purified by column chromatography eluted with hexane-ethyl acetate-dichloromethane (3:1:3 v/v), TLC: R_f_ = 0.45 (hexane-ethyl acetate-dichloromethane 3:1:3 v/v). Selected IR and NMR spectra is show at BC-BE Figs in S1 File.

**Data for 4-(3-(4-allyl-2-methoxyphenoxy)propyl)-1-(4-nitrobenzyl)-1*H*-1,2,3-triazole (42)**

White solid, m.p. 79.7–80.4°C, purified by column chromatography eluted with hexane-ethyl acetate-dichloromethane (3:1:3 v/v), TLC: R_f_ 0.29 (hexane-ethyl acetate-dichloromethane 3:1:3 v/v). Selected IR and NMR spectra is show at BF-BH Figs in S1 File.

**Data for 4-(3-(4-allyl-2-methoxyphenoxy)propyl)-1-(4-methoxybenzyl)-1*H*-1,2,3-triazole (43)**

White solid, m.p. 88.9–90.4°C, purified by column chromatography eluted with hexane-ethyl acetate-dichloromethane (3:1:3 v/v), TLC: R_f_ = 0.22 (hexane-ethyl acetate-dichloromethane 3:1:3 v/v). Selected IR and NMR spectra is show at BI-BK Figs in S1 File.

**Data for 4-(3-(4-allyl-2-methoxyphenoxy)propyl)-1-(4-(trifluoromethoxy)benzyl)-1*H*-1,2,3-triazole (44)**

White solid, m.p. 93.4–94.9°C, purified by column chromatography eluted with hexane-ethyl acetate-dichloromethane (3:1:3 v/v), TLC: R_f_ = 0.49 (hexane-ethyl acetate-dichloromethane 3:1:3 v/v). Selected IR and NMR spectra is show at BL-BN Figs in S1 File.

**Data for 4-(3-(4-allyl-2-methoxyphenoxy)propyl)-1-(4-(trifluoromethyl)benzyl)-1*H*-1,2,3-triazole (45)**

White solid, m.p. 111.3–112.5°C, purified by column chromatography eluted with hexane-ethyl acetate-dichloromethane (3:1:3 v/v), TLC: R_f_ = 0.50 (hexane-ethyl acetate-dichloromethane 3:1:3 v/v). Selected IR and NMR spectra is show at BO-BQ Figs in S1 File.

**Data for 4-(3-(4-allyl-2-methoxyphenoxy)propyl)-1-(3,4-difluorobenzyl)-1*H*-1,2,3-triazole (46)**

White solid, m.p. 97.1–97.8°C, purified by column chromatography eluted with hexane-ethyl acetate-dichloromethane (3:1:3 v/v), TLC: R_f_ = 0.41 (hexane-ethyl acetate-dichloromethane 3:1:3 v/v). Selected IR and NMR spectra is show at BR-BT Figs in S1 File.

**Data for 4-(3-(4-allyl-2-methoxyphenoxy)propyl)-1-(4-methylbenzyl)-1*H*-1,2,3-triazole (47)**

White solid, m.p. 59.6–60.9°C, purified by column chromatography eluted with hexane-ethyl acetate-dichloromethane (3:1:3 v/v), TLC: R_f_ = 0.42 (hexane-ethyl acetate-dichloromethane 3:1:3 v/v). Selected IR and NMR spectra is show at BU-BX Figs in S1 File.

**Data for 4-(3-(4-allyl-2-methoxyphenoxy)propyl)-1-(2-bromobenzyl)-1*H*-1,2,3-triazole (48)**

White solid, m.p. 47.8–49.1°C, purified by column chromatography eluted with hexane-ethyl acetate-dichloromethane (3:1:3 v/v), TLC: R_f_ = 0.61 (hexane-ethyl acetate-dichloromethane 3:1:3 v/v). Selected IR and NMR spectra is show at BW-BZ Figs in S1 File.

**Data for 4-(3-(4-allyl-2-methoxyphenoxy)propyl)-1-(3-bromobenzyl)-1*H*-1,2,3-triazole (49)**

White solid, m.p. 65.9–67.1°C, purified by column chromatography eluted with hexane-ethyl acetate-dichloromethane (3:1:3 v/v), TLC: R_f_ = 0.32 (hexane-ethyl acetate-dichloromethane 3:1:3 v/v). Selected IR and NMR spectra is show at CA-CC Figs in S1 File.

#### 2,2-di(prop-2-yn-1-yl)-1*H*-indene-1,3(2*H*)-dione (50)

To a mixture of indan-1,3-dione (0.146 g, 1.00 mmol) and distilled water (40 mL), it was added potassium hydroxide (0.140 g, 0.250 mmol) and propargyl bromide (0.364 mL, 4.00 mmol). The resulting mixture was magnetically stirred at 40 ^o^C for 24h. After this time, the reaction mixture was cooled down to room temperature and it was neutralized with aqueous solution of HCl 0.100 mol L^-1^. The aqueous phase was extracted with ethyl acetate (3 x 20 mL). The organic extracts were combined and the resulting organic phase was washed with brine (15 mL), dried over anhydrous sodium sulfate, filtered, and concentrated under reduced pressure. Compound **50** was not submitted to any purification process and it was obtained in 93% yield (0.207 g, 0.930 mmol).

#### Synthesis of compounds 51–63 exemplified by the synthesis of 2,2-bis((1-(4-bromobenzyl)-1*H*-1,2,3-triazol-4-yl)methyl)-1*H*-indene-1,3(2*H*)-dione (51)

To a 10 mL round bottom-flask, containing 1.5 mL of dichloromethane and 1.5 mL of distilled water, it was added 4-bromobenzylazide (168.8 mg, 0.800 mmol), alkyne **50** (88.8 mg, 0.400 mmol), and sodium ascorbate (60.0 mg, 0.300 mmol). Then, it was added CuSO_4_·5H_2_O (38.0 mg, 0.150 mmol) and the resulting mixture was vigorously stirred at room temperature for 2 hours. After that, it was added to the reaction mixture sodium carbonate saturated aqueous solution (20 mL) and the resulting aqueous phase was extracted with dichloromethane (3 x 15 mL) followed by ethyl acetate (3 x 15 mL). The organic extracts were combined and the resulting organic phase was dried over anhydrous sodium sulfate, filtered, and concentrated under reduced pressure. The residue was purified by silica-gel column chromatography eluted with hexane-ethyl-acetate-dichloromethane 3:1:3 v/v. Compound **51** was obtained in 51% yield (143.1 mg, 0.220 mmol). Selected IR and NMR spectra is show at CD-CF Figs in S1 File.

Compounds **52**–**63** were synthesized employing a procedure similar to that described for the preparation of **51**. The structures of substances **52**–**63** are supported by the following data.

**Data for 2,2-bis((1-(3-bromobenzyl)-1*H*-1,2,3-triazol-4-yl)methyl)-1*H*-indene-1,3(2*H*)-dione (52)**

White solid, m.p. 185.6–187.2 ^o^C, purified by silica gel column chromatography eluted with hexane-ethyl-acetate-dichloromethane (3:1:1 v/v), TLC: R_f_ = 0.08 (hexane-ethyl acetate-dichloromethane 3:1:1 v/v). Selected IR and NMR spectra is show at CG-CI Figs in S1 File.

**Data for 2,2-bis((1-(2-bromobenzyl)-1*H*-1,2,3-triazol-4-yl)methyl)-1*H*-indene-1,3(2*H*)-dione (53)**

Pale yellow solid, m.p. 178.4–179.7 ^o^C, purified by silica gel column chromatography eluted with hexane-ethyl-acetate-dichoromethane (3:1:1 v/v), TLC: R_f_ = 0.11 (hexane-ethyl acetate-dichloromethae 3:1:1 v/v). Selected IR and NMR spectra is show at CJ-CL Figs in S1 File.

**Data for 2,2-bis((1-(4-chlorobenzyl)-1*H*-1,2,3-triazol-4-yl)methyl)-1*H*-indene-1,3(2*H*)-dione (54)**

White solid, m.p. 222.3–223.9 ^o^C, purified by silica gel column chromatography eluted with hexane-ethyl-acetate-dichloromethane (3:1:1 v/v), TLC: R_f_ = 0.02 (hexane-ethyl acetate-dichoromethane 3:1:1 v/v). Selected IR and NMR spectra is show at CM-CO Figs in S1 File.

**Data for 2,2-bis((1-(4-fluorobenzyl)-1*H*-1,2,3-triazol-4-yl)methyl)-1*H*-indene-1,3(2*H*)-dione (55)**

White solid, m.p. 223.0–224.5 ^o^C, purified by silica gel column chromatography eluted with hexane-ethyl-acetate-dichloromethane (3:1:1 v/v), TLC: R_f_ = 0.01 (hexane-ethyl acetate-dichoromethane 3:1:1 v/v). Selected IR and NMR spectra is show at CP-CR Figs in S1 File.

**Data for 2,2-bis((1-(4-methoxybenzyl)-1*H*-1,2,3-triazol-4-yl)methyl)-1*H*-indene-1,3(2*H*)-dione (56)**

White solid, m.p. 118.5–119.8 ^o^C, purified by silica gel column chromatography eluted with hexane-ethyl-acetate-dichloromethane (3:1:1 v/v), TLC: R_f_ = 0.01 (hexane-ethyl acetate-dichloromethane 3:1:1 v/v). Selected IR and NMR spectra is show at CS-CU Figs in S1 File.

**Data for 2,2-bis((1-(4-trifluoromethoxybenzyl)-1*H*-1,2,3-triazol-4-yl)methyl)-1*H*-indene-1,3(2*H*)-dione (57)**

White solid, m.p. 253.7–254.5 ^o^C, purified by silica gel column chromatography eluted with hexane-ethyl-acetate-dichloromethane (3:1:1 v/v), TLC: R_f_ = 0.02 (hexane-ethyl acetate-dichloromethane 3:1:1 v/v). Selected IR and NMR spectra is show at CV-CW Figs in S1 File.

**Data for 2,2-bis((1-(4-nitrobenzyl)-1*H*-1,2,3-triazol-4-yl)methyl)-1*H*-indene-1,3(2*H*)-dione (58)**

White solid, m.p. 271.2–272.4 ^o^C, purified by silica gel column chromatography eluted with hexane-ethyl-acetate-dichloromethane (3:1:1 v/v), TLC: R_f_ = 0.01 (hexane-ethyl acetate-dichloromethane 3:1:1 v/v). Selected IR and NMR spectra is show at CY-DA Figs in S1 File.

**Data for 2,2-bis((1-(2,4-difluorobenzyl)-1*H*-1,2,3-triazol-4-yl)methyl)-1*H*-indene-1,3(2*H*)-dione (59)**

White solid, m.p. 178.3–179.5 ^o^C, purified by silica gel column chromatography eluted with hexane-ethyl-acetate-dichloromethane (3:1:1 v/v), TLC: R_f_ = 0.06 (hexane-ethyl acetate-dichloromethane 3:1:1 v/v). Selected IR and NMR spectra is show at DB-DD Figs in S1 File.

**Data for 2,2-bis((1-(4-iodobenzyl)-1*H*-1,2,3-triazol-4-yl)methyl)-1*H*-indene-1,3(2*H*)-dione (60)**

White solid, m.p. 211.3–212.2 ^o^C, purified by silica gel column chromatography eluted with hexane-ethyl-acetate-dichloromethane (3:1:1 v/v), TLC: R_f_ = 0.06 (hexane-ethyl acetate 3:1:1 v/v). Selected IR and NMR spectra is show at DE-DG Figs in S1 File.

**Data for 2,2-bis((1-(2-benzyl)-1*H*-1,2,3-triazol-4-yl)methyl)-1*H*-indene-1,3(2*H*)-dione (61)**

White solid, m.p. 161.5–162.8 ^o^C, purified by silica gel column chromatography eluted with hexane-ethyl-acetate-dichloromethane (3:1:1 v/v), TLC: R_f_ = 0.05 (hexane-ethyl acetate-dichloromethane 3:1:1 v/v). Selected IR and NMR spectra is show at DH-DJ Figs in S1 File.

**Data for 2,2-bis((1-(4-trifluoromethylbenzyl)-1*H*-1,2,3-triazol-4-yl)methyl)-1*H*-indene-1,3(2*H*)-dione (62)**

White solid, m.p. 243.7–244.6 ^o^C, purified by silica gel column chromatography eluted with hexane-ethyl-acetate-dichloromethane (3:1:1 v/v), TLC: R_f_ = 0.24 (hexane-ethyl acetate-dichloromethane 3:1:1 v/v). Selected IR and NMR spectra is show at DK-DM Figs in S1 File.

**Data for 2,2-bis((1-(4-methylbenzyl)-1*H*-1,2,3-triazol-4-yl)methyl)-1*H*-indene-1,3(2*H*)-dione (63)**

White solid, m.p. 170.8–171.4 ^o^C, purified by silica gel column chromatography eluted with hexane-ethyl-acetate-dichloromethane (3:1:1 v/v), TLC: R_f_ = 0.08 (hexane-ethyl acetate-dichloromethane 3:1:1 v/vSelected IR and NMR spectra is show at DN-DP Figs in S1 File.

### Evaluation of the inhibitory activity of compounds 1–18, 23–49, and 51–63 on WNV NS2B-NS3 protease

In order to assess the activity of the synthesized compounds on WNV NS2B-NS3pro, recombinant WNV NS2B-NS3 protease (catalog number SE-2907, already purified and activated) and fluorescent substrate pERTKR-AMC (catalog number ES013) were purchased from R & D Systems (Minneapolis, MN, United States). Compounds **1**–**18**, **23**–**49**, and **51**–**63** were dissolved in pure dimethyl sulfoxide (DMSO); solutions were then diluted in a buffer to obtain working solutions with a final concentration of 1% v/v DMSO. A volume of 50 *μ*L of purified NS2B-NS3pro (final concentration of 1 ng *μ*L^-1^) diluted in a buffer (50 mmol L^-1^ Tris, 30% (v/v) glycerol, pH 9.5) was incubated with 50 *μ*L of each compound (final concentration of 16 *μ*mol L^-1^) in a 96-well black plate for 30 min at 21–22°C. After this time, the assay was initiated by addition of 50 *μ*L of the substrate (40 mmol L^-1^ –initial concentration). A solution containing a buffer and DMSO was used as negative control on the same plate. The blank contained 50 *μ*L of the buffer and 100 *μ*L of the substrate. The fluorescence intensity was continuously recorded at a 360 nm excitation wavelength and at an emission wavelength of 460 nm using a SpectraMax^®^ M5 microplate reader (Molecular Devices, San José, CA, United States). Compounds which effectively inhibited the enzyme were selected for further biological assays. Analyses were performed using Microsoft Excel (Microsoft Office Software) and GraphPad Prism 6 (GraphPad Software Inc.). The assays were conducted in triplicate during three isolated experiments, and the statistical analyses were conducted by utilizing the multiple comparisons of one-way ANOVA.

### Determination of IC_50_

The inhibitory enzymatic activity of compound **35**, the one most active against the WNV NS2-NB3 protease, was evaluated at eight different concentrations (66 *μ*mol L^-1^–0.5 *μ*mol L^-1^) using the protease assay as described above. Fluorescence was measured in triplicate wells at intervals of 30 s for 5 min in three independent experiments. IC_50_ values were calculated using GraphPad Prism software 6 (GraphPad Software Inc., San Diego, CA, United States), using four-parameter nonlinear regression analysis (Hill slope method).

### Determination of K_i_

Three different concentrations (2, 4 and 8 *μ*mol L^-1^) of inhibitor **35** and five different concentrations of substrate pERTKR-AMC (20, 40, 60, 80, 100 mmol L^-1^) were tested *in vitro* against the WNV protease (37.04 nmol L^-1^ protein, 1 ng *μ*L^-1^). Fluorescence was measured in triplicate wells at an interval of 30 s. The velocity values (RFU/minute) were then calculated for each substrate/inhibitor pair. K_i_ values were calculated with GraphPad Prism software 6 (GraphPad Software Inc., San Diego, CA, United States) with non-linear regression in the competitive inhibition mode of enzyme-kinetics.

### Cytotoxicity assay

The cytotoxicity of compound **35** was assessed using an MTT assay [44]. VERO cells (5 x 10^4^ cells) were seeded in 96-well plates. Each well contained 100 *μ*L of each compound solution at different concentrations (1000, 250, 125, 63, 32, 17, 8 and 4 *μ*mol L^-1^). The compound was diluted in MEM medium with 2% FBS and 1% DMSO. After 24 h of incubation at 37 ^o^C, 100 *μ*L of the MTT solution (5%) was added to the wells. After 4 h at 37°C, the MTT solution was removed and 100 *μ*L/well of DMSO was added to solubilize the formazan. Absorbance was measured at 550 nm in a microplate reader (Multiskan^™^ GO Microplate Spectrophotometer–ThermoFisher^®^, Waltham, MA, United States). The data were analyzed and CC_50_ was determined using GraphPad Prism 6.

### Virucidal assay

The virucidal assay was performed as described by Oliveira et al. [41].

### Molecular modeling studies

Ligands were prepared in the Ligprep program (Schrödinger, New York, NY, United States) [45] employing the OPLS_2005 force field, with protonation states predicted using Epik at pH 9.5 ± 2.0. The West Nile Virus protease NS2B-NS3 PDB code 2IJO [46], chosen as the receptor, was prepared with the Protein Preparation Wizard (Schrödinger, New York, NY, United States), with removal of all waters and addition of hydrogens based on PROPKA calculations at pH 9.5. Docking calculations were performed with the Glide software (Schrödinger, New York, NY, United States) [47, 48], employing the Induced Fit docking methodology [49] and Glide SP. All the software packages used are part of the Schrödinger Release 2016–2 package (Schrödinger, New York, NY, United States) [50]. A spherical grid with 12 Å radius was centered in the Isoleucine 123 residue so that the active site was fully included within the grid. All residues within 5 Å from the center were considered flexible. Docking results were ranked based on their docking score and the top ranking poses for each compound were analyzed with the Maestro 10.6 software (Schrödinger, New York, NY, United States) [51].

## Results and discussion

### Synthesis of compounds 1–18, 23–49, and 51–63

The structures of the synthesized compounds are depicted in Fig 2.

As previously mentioned, the synthesis of isobenzofuran-(3*H*)-ones **1–18** has been previously reported [43].

**Fig 3:**
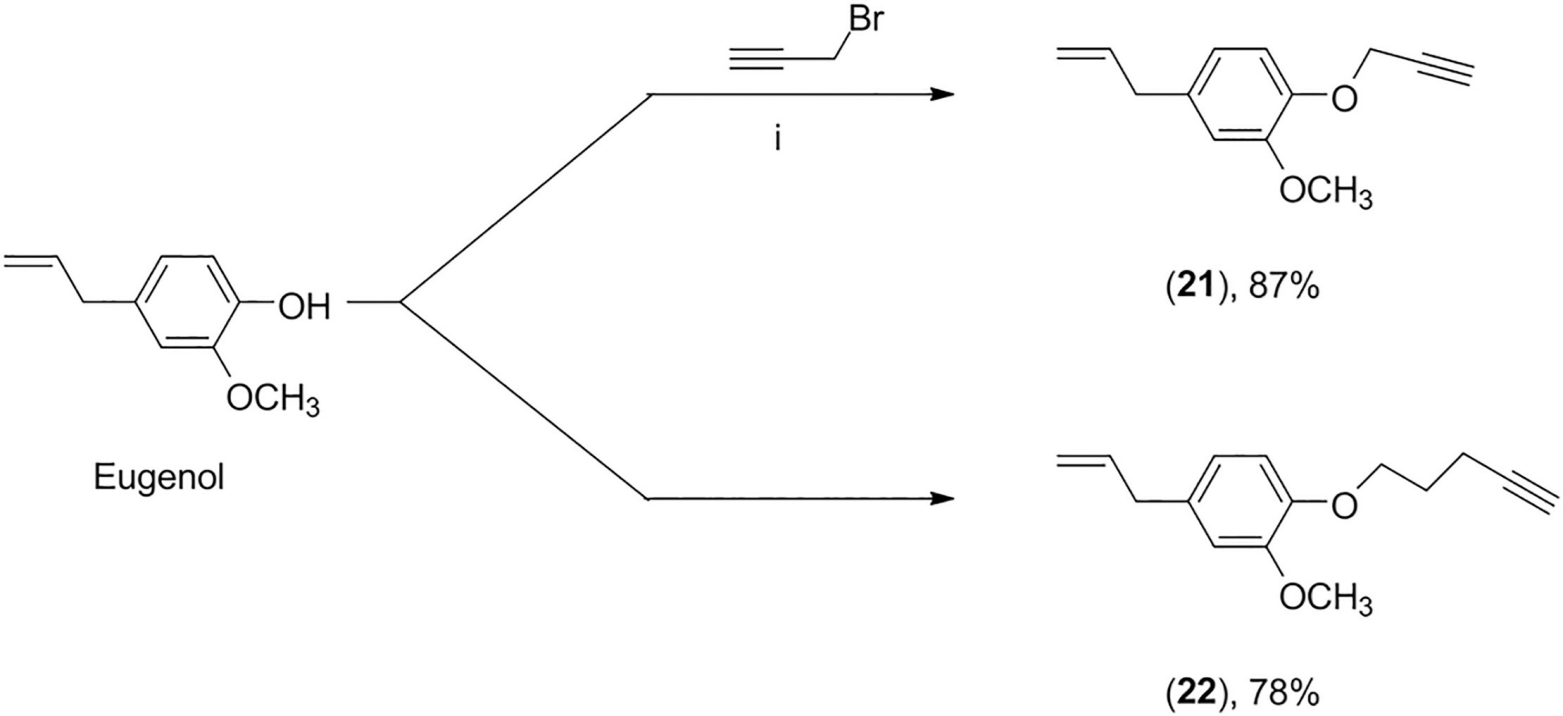
Preparation of alkylated derivatives of eugenol. i) NaOH, CH_3_OH, 40°C; acetonitrile, r.t; ii) NaOH, CH_3_OH, 40 ^o^C; acetonitrile, 70 ^o^C.

For the preparation of compounds **23–49**, we initially isolated eugenol (**19**) from dried flower buds of *Eugenia caryophyllata* via hydrodistillation. Subsequently, eugenol (**19**) was submitted to alkylation procedures, affording terminal alkynes **21** and **22** in yields of, respectively, 87% and 78% (Fig 3).

**Fig 4:**
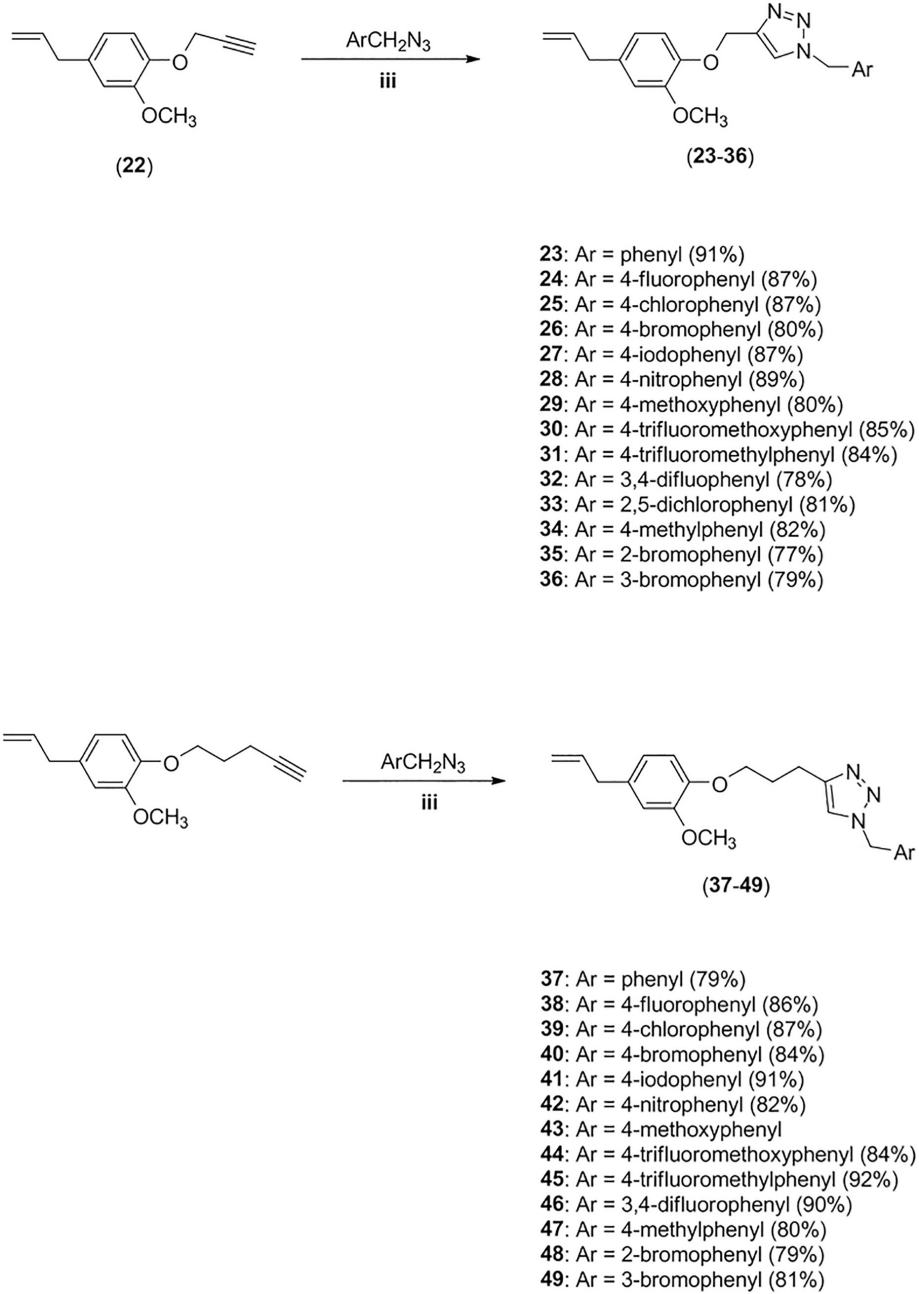
Preparation of eugenol derivatives 23–49. iii) sodium ascorbate (40 mol%), CuSO_4_·5H_2_O (20 mol%), EtOH/H_2_O (1:1 v/v).

Then, the CuAAC reactions (click reactions) between benzyl azides (ArCH_2_N_3_) and terminal alkynes **21** and **22** led to the formation of triazolic derivatives **23–49** (Fig 4). The click reactions, in general, took less than one minute for their completion.

**Fig 5:**
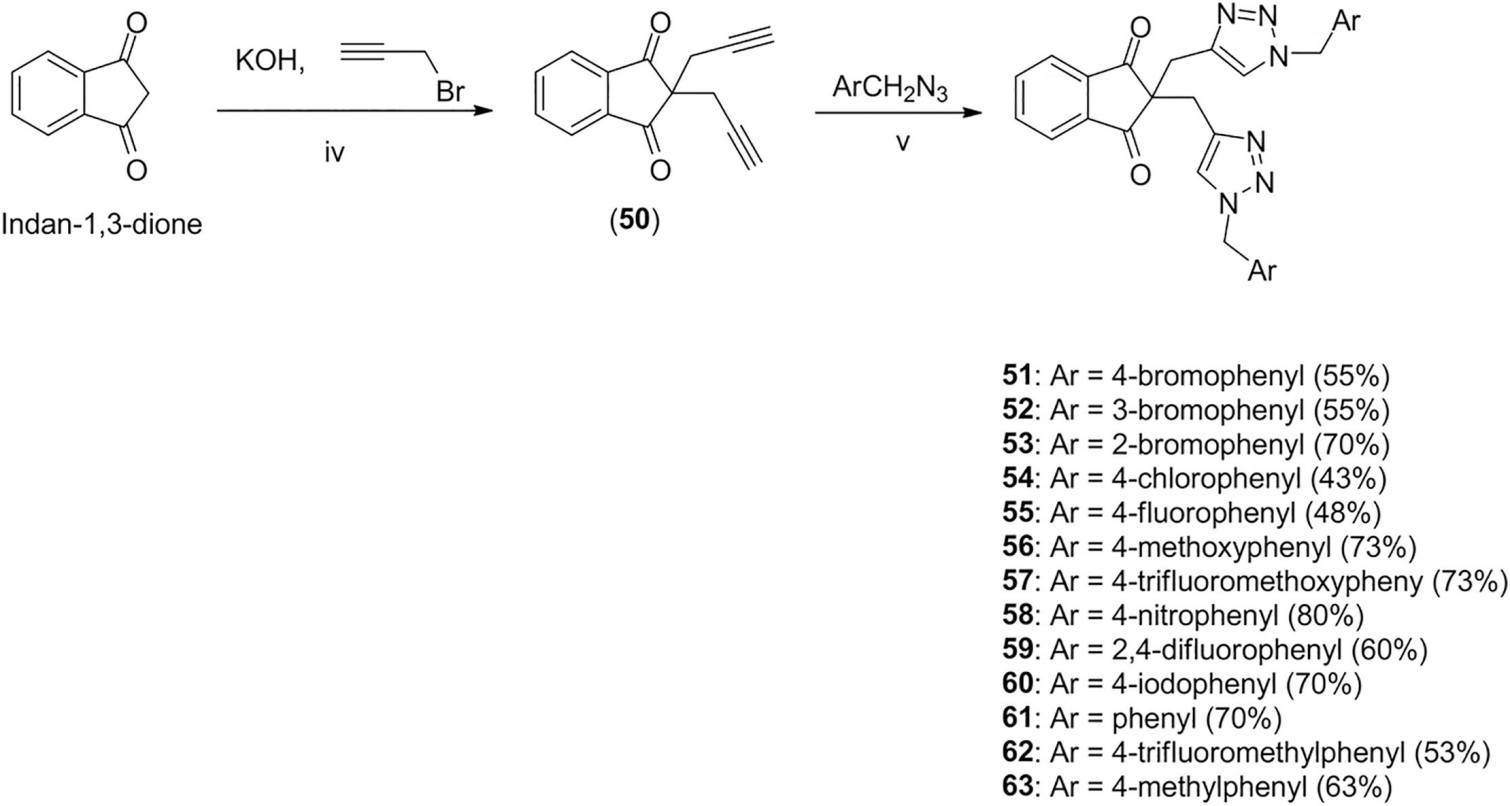
Synthesis of compounds 51–63. iv) aqueous KOH, then propargyl bromide, 40 ^o^C, 24h; v) sodium ascorbate (40 mol%), CuSO_4_·5H_2_O (20 mol%), CH_2_Cl_2_/H_2_O (1:1 v/v), 2h.

A similar sequence, namely alkylation of indan-1,3-dione followed by click reaction between compound **50** and benzyl azides, was utilized to prepared triazolic compounds **51–63** (Fig 5).

It should be mentioned that the azides, used in the preparation of compounds **23–49** and **51–63,** were obtained via the methodology previously described in the literature [52]. Once prepared, the synthesized compounds were submitted to biological assays to evaluate their inhibitory effects against the NS2-NB3 protease of WNV.

### Identification of WNV protease inhibitors

The *Flavivirus* protease is essential for the processing of the polyprotein which generates the viral proteins required for viral replication and maturation of infectious virions. Therefore, the protease is an ideal target for the discovery of antivirals against *Flavivirus* [53]. In the present investigation, we evaluated the inhibitory activity of fifty-eight compounds against the WNV NS2B-NS3 protease (eighteen 3-(2-oxo-2-aryl)-isobenzofuran-1(3*H*)-ones, compounds **1–18**; twenty-seven derivatives of eugenol with triazole rings, compounds **23–49**; thirteen derivatives of indan-1,3-diones presenting triazole rings, compounds **51–63**, Fig 2). In this regard, a purified preparation of WNV NS2B3NS3pro and the fluorogenic peptide substrate pERTKR-AMC was utilized.

**Fig 6:**
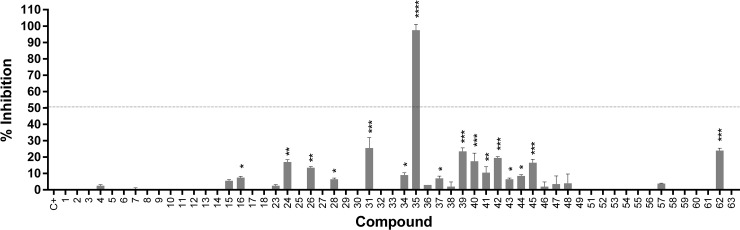
Inhibitory activity screening for WNV protease. Fifty-eight compounds were evaluated at the concentration of 16 *μ*mol L^-1^ against the WNV NS2-NB3 protease. The assays were conducted in triplicate during three isolated experiments (p value < 0.0001).

Our primary screen resulted in the identification of eighteen compounds (~31% of the evaluated compounds) that exhibited inhibitory effects on protease activity (Fig 6).

Subsequently, we took these eighteen compounds and conducted a secondary screening with further validation based on their relative strengths of inhibition (at least 50%). This resulted in the selection of compound **35**, a eugenol derivative which was utilized in further experiments.

A derivative of the natural product eugenol capable of significantly inhibiting the activity of the WNV NS2B-NS3 protease was identified. Taking the eugenol derivatives into consideration, we made variations in the size of the carbon chain that links eugenol moiety and the triazole ring. Also, the substitution pattern of the aromatic ring attached to the triazolic portion was varied. These modifications afforded a group of eugenol triazolic derivatives from which was identified the very active compound **35**. It is important to mention that further chemical modifications can be planned regarding the eugenol and triazole fragments so that new derivatives with improved activity may be obtained. Considering that there is no *Flavivirus* protease inhibitor approved for pre-clinical trial [54], the exploitation of the new scaffold herein identified, namely triazolic derivatives of eugenol, would be of considerable importance.

### Determination of IC_50_ and Ki values of the enzymatic inhibitory activity of compound 35

**Fig 7:**
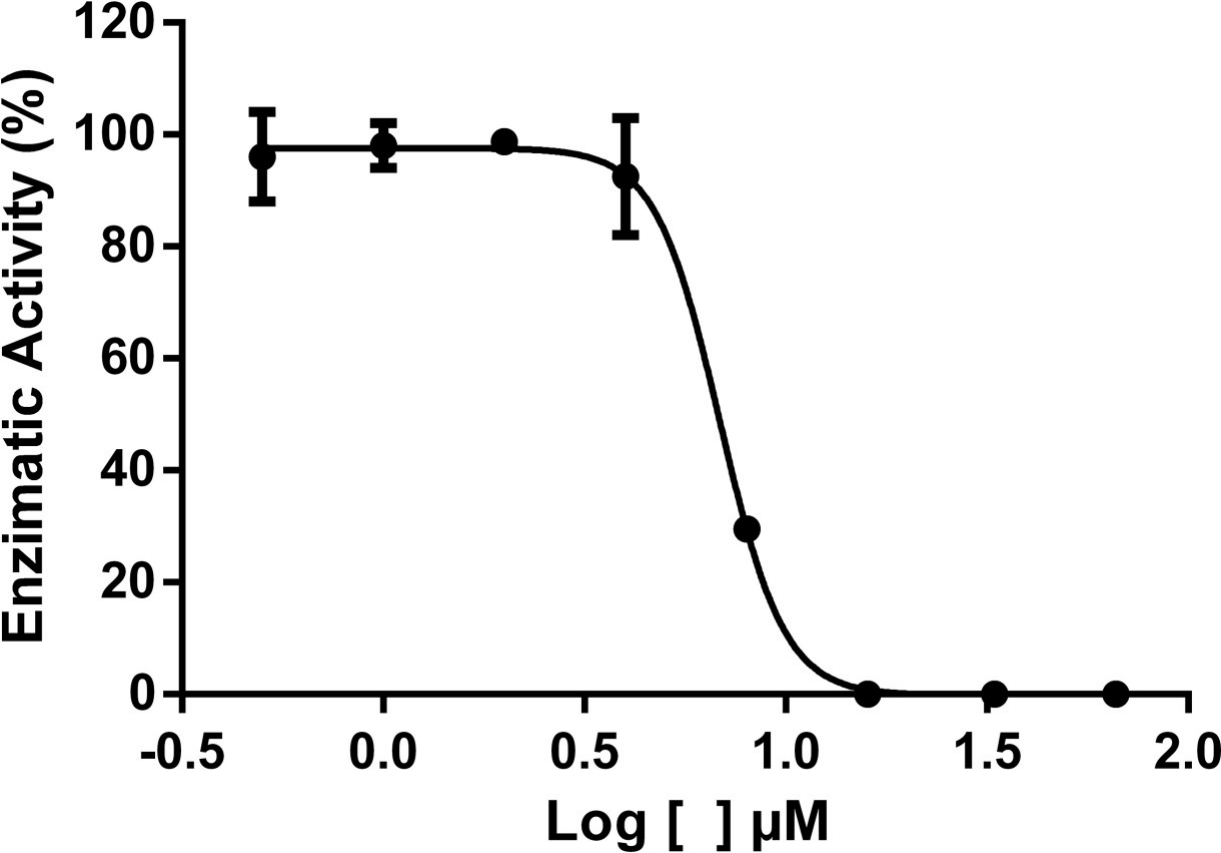
Enzymatic inhibitory profile of compound 35.

The enzymatic inhibition was evaluated in the presence of varying concentrations (0.5 *μ*mol L^-1^ to 66 *μ*mol L^1^) of compound **35**. A dose-response inhibition was noticed with IC_50_ value of 6.86 *μ*mol L^-1^ (Fig 7).

**Fig 8:**
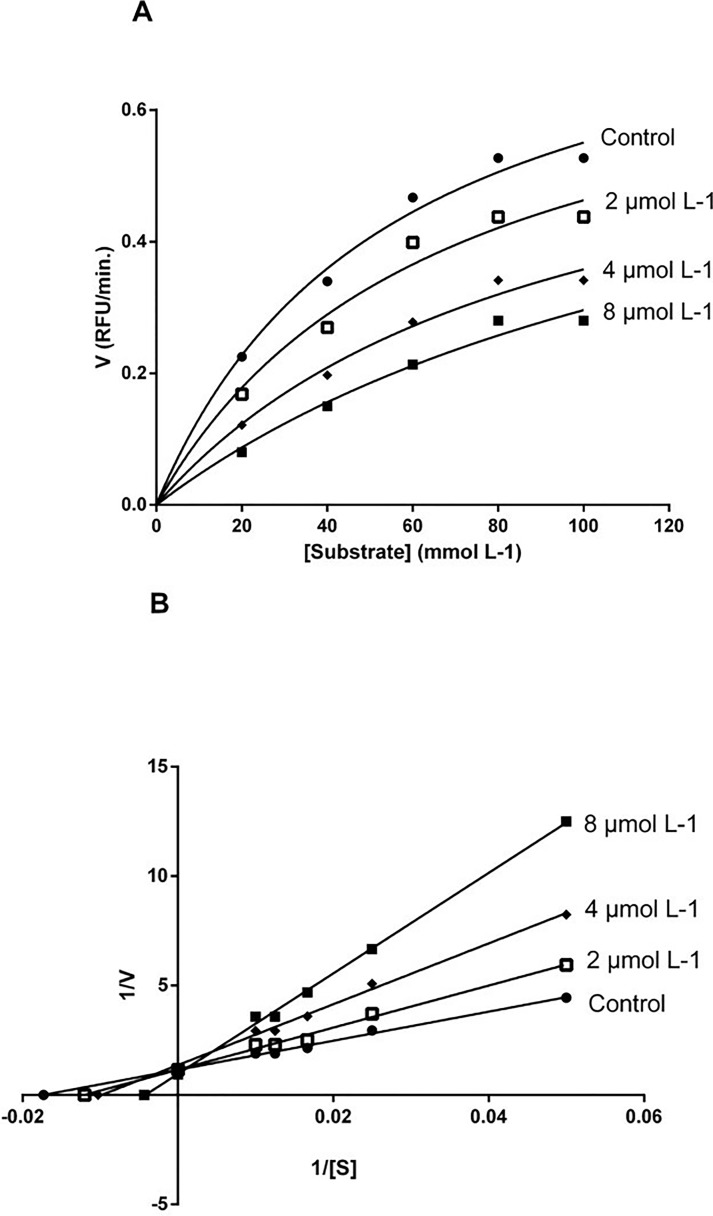
Enzymatic kinetics of WNV NS2-NB3 protease in the presence of compound 35. In **A,** the Michaelis-Menten plot is shown. In **B**, the Lineweaver-Burk plot is presented.

The enzyme kinetic assay was conducted under five different substrate concentrations and three varying concentrations of compound **35**. We used the Michaelis-Menten equation to find the values of V_MAX_ and K_M_; and with these, the Lineweaver-Burk graph was built. Compound **35** showed a decrease of enzymatic V_MAX_ and an increase in K_M_, presenting the behaviour of a competitive inhibitor (Fig 8A and 8B). The K_i_ value was 3.06 (± 0.38) *μ*mol L^-1^.

For compound 35, the enzyme kinetic data showed an increase in the K_M_ value, a typical behaviour of competitive inhibitors. In addition, this compound displayed a low K_i_ value. In a recent investigation, Balasubramanian and collaborators [53] found eight promising flavivirus protease inhibitors presenting K_i_ values within the 0.22 to 6.9 *μ*mol L^-1^ range. However, the K_i_ value may not be analyzed individually. In the study by Balasubramanian and collaborators, a compound presenting low K_i_ value (0.22 *μ*mol L^-1^) but displaying a CC_50_ of 29.16 *μ*mol L^-1^ was identified [53].

For the development of new drugs, the World Health Organization (WHO) strongly recommends the use of compounds with pharmacological effects already described and substances already approved for clinical use, so that several steps can be abbreviated in the long validation process [55]. Eugenol (**19**) has been safely used in *in vivo* experiments, and it has been recognized as a safe, effective and inexpensive anesthetic for fish, amphibians and rats [56, 57]. Also, the analgesic effect of eugenol (**19**) in different models of pain has been well documented [58–62]. A recent work with BALB/c mice used a derivative of eugenol (**19**) to treat visceral Leishmaniasis. This derivative presented low cytotoxicity for macrophages as well as for naive mice with immune-stimulatory activity. Moreover, no biochemical alterations in hepatic and renal enzymes were noticed [63]. Eugenol (**19**) is generally non-allergenic for humans, although in sensitized individuals it may cause a range of tissue reactions from low-grade local to systemic. Low concentrations of eugenol (**19**) are well known to exert local anti-inflammatory, antiseptic, and anesthetic effects on dental pulp. Also, eugenol (**19**) may have antibacterial effects that are beneficial for dental hygiene, being included in materials such as toothpastes and mouthwashes [64–66]. All these features make eugenol (**19**), as well as its derivatives, very interesting compounds to be explored in drug development.

### Cytotoxicity assay

**Fig 9:**
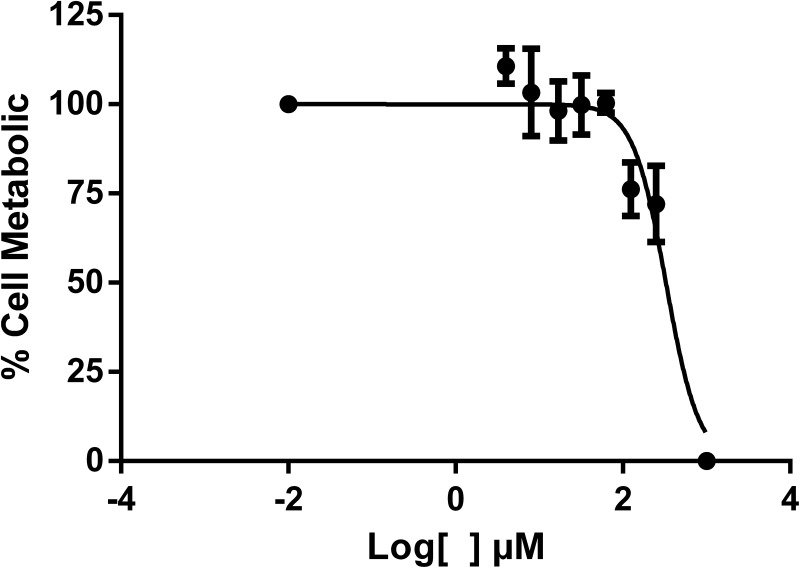
Dose-response profile of compound 35 on Vero cells.

The cytotoxicity of compound **35** on Vero cells was investigated via the colorimetric MTT assay and the determined CC_50_ value was 327.20 *μ*mol L^-1^ (Fig 9). Luo and collaborators [54] reported the best parameters for the most promising compounds against the protease of *Flavivirus*. They highlighted a compound with low cytotoxicity presenting CC_50_ superior to 300 *μ*mol L^-1^. Eugenol derivative **35** presented a similar CC_50_, and it can be considered a compound with low cytotoxicity. Therefore, although a very potent inhibitor, this compound presented considerable cytotoxicity.

### Molecular modeling

Multiple targets and inhibitory mechanisms have been proposed for *Flavivirus* proteases so far [53, 54, 67, 68], which have provided insightful structural information regarding the NS2B-NS3 catalytic site, helping computational-aided drug design efforts even further. For instance, by solving NS2B-NS3’s tridimensional structure bound to a peptide-like inhibitor, Erbel and coworkers [69] provided fundamental information regarding the protease fold, catalytic site structure and inhibition mechanisms. As found by others [46, 70–72], inhibitors often bind to His^51^ or to nearby residues such as Asp^75^, Asp^129^, Gly^153^ and Tyr^161^ (S1 Fig), hampering the bond of the substrate to the catalytic site.

**Fig 10:**
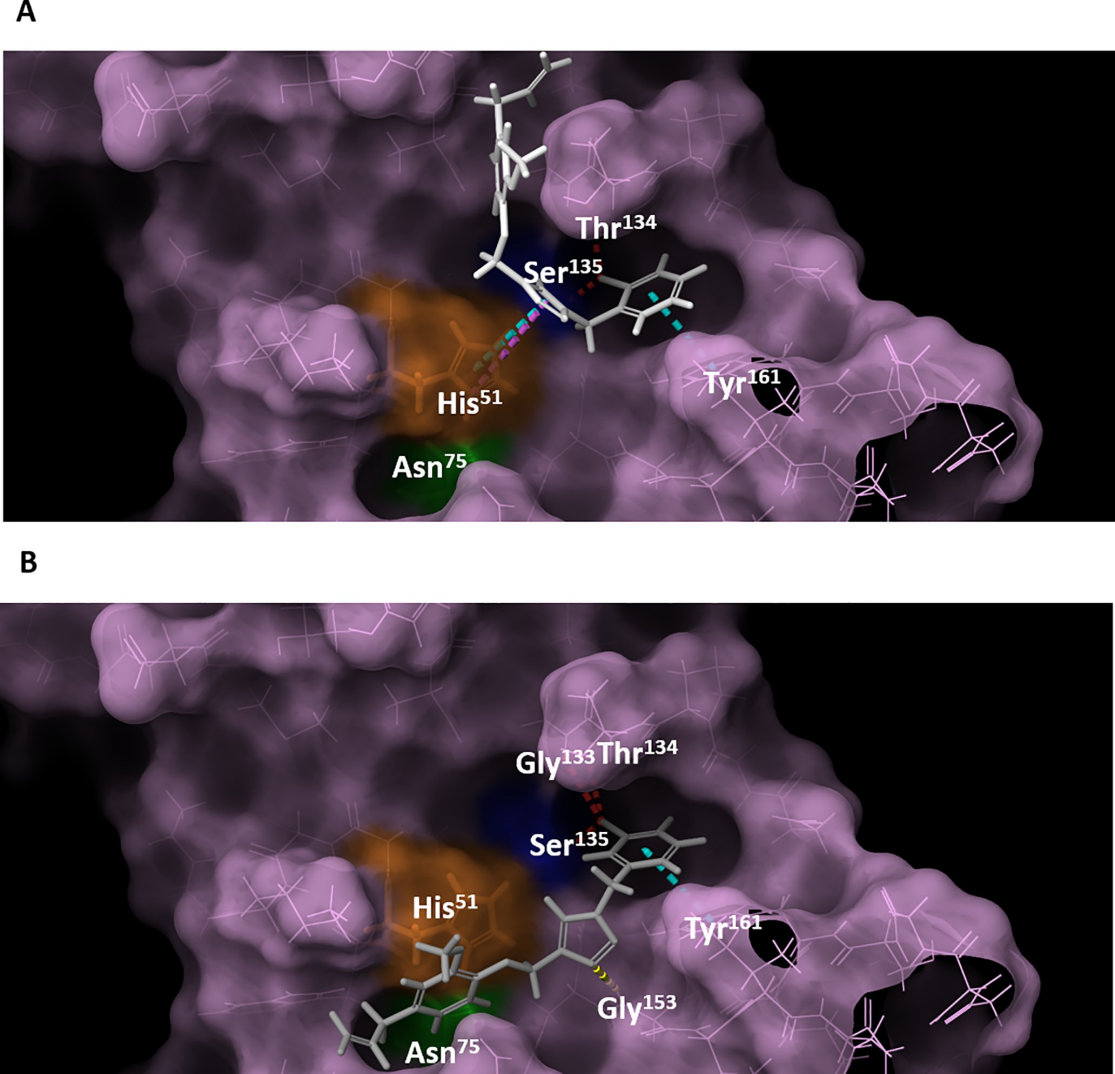
WNV protease docking with compound 35 and 36. The entire protease is shown in lilac, highlighting residues Ser^135^ (dark blue), His^51^ (orange), and Asp^75^ (green). **A.** In white is compound **35**. **B.** In gray is compound **36**. Hydrogen bonds are shown in yellow, halogen bonds are shown in red, T-shape π-π interactions are shown in cyan and cation-π interactions are shown in pink.

In order to perform our own molecular docking calculations, the crystallographic structure of the NS2B-NS3 protease was obtained from PDB 2IJO, in which NS2B-NS3 is co-crystallized with the WNV protease inhibitor aprotinin. All molecules derived from eugenol were analyzed for their probable three-dimensional binding conformation, binding energy, chemical groups involved, profile of binding and identity of the involved amino acids, as observed in table A in S1 File. The predicted recognition mechanism for compound **35** relies on interactions with His^51^, Thr^134^, Ser^135^ and Tyr^161^, as represented in Fig 10A, and the ligand occupies pocket S1 of the catalytic site. The NH^+^ of the His^51^ ring interacts with the triazole ring of **35** in cation-π and π-π type interactions, and the Tyr^161^ ring interacts with the phenyl ring of **35** via π-π type interaction, while a halogen bond can be formed between the Br atom and the OH from Ser^135^ or the NH from Thr^134^. It is important to highlight that both His^51^ and Ser^135^ are members of the catalytic triad of the NS2B-NS3 protease, which might explain the observed competitive inhibitory activity of **35**.

In addition, compound **36** showed no inhibitory activity, despite the fact that the only difference from compound **35** is the position of a bromo substituent (*orto* in **35** and *meta* in **36***)*. Our calculations suggest that the formation of a halogen bond for **36** with bromo in *meta* might induce a more stable conformation for the ligand in which the triazole ring interacts with Gly^153^ instead of His^51^ and, consequently, leaves the S1 pocket free (Fig 10B). These results suggest that the bromo substituent in *orto* is pivotal for a proper recognition of compound **35**, along with the cation-π interaction between His^51^ and the triazole ring, while the methoxyphenyl ring region might be a suitable target for further improvements.

### Virucidal assay

To determine whether compound **35** would have antiviral activity for *Flaviviruses*, a virucidal assay was performed with four serotypes of DENV, due to its close evolutionary proximity to WNV and due to the highly conserved NS2B-NS3 fold and sequence. This assay was performed by prior incubation of the compound with each viral strain, followed by its addition to the cell layer for virus adsorption and internalization. Subsequently, the compound-virus solution was removed and antiviral action was observed through the formation of lysis plates. The concentration of test compounds that inhibited 50% of the viral infection (EC_50_) was obtained by nonlinear regression, leading to the calculation of the Selectivity Index (SI).The observed results are presented in Table 1.

**Table 1:** CC_50_, EC_50_ and SI values of the compound 35 in the presence of DENV-1-4.

Compound	CC_50_ *μ*mol L^-1^	DENV-1		DENV-2		DENV-3		DENV-4	
		EC_50_ *μ*mol L^-1^	SI	EC_50_ *μ*mol L^-1^	SI	EC_50_ *μ*mol L^-1^	SI	EC_50_ *μ*mol L^-1^	SI
**35**	327,20	46,57	7	49,20	7	70,10	5	14,17	23

The compound had good SI values for DENV-1-3 and a higher value for DENV-4. All together, the results indicate that compound **35** has significant antiviral efficacy and is a promising antiviral candidate for *Flavivirus*.

## Conclusion

In this work, a small library of fifty-eight synthetic compounds (isobenzofuran-1(3*H*)-ones and triazolic derivatives of eugenol and indandione) were screened against the WNV NS2B-NS3 protease. By modifying the structure of the natural product eugenol to produce 1,2,3-triazolic derivatives, a compound presenting low cytotoxicity and considerable inhibitory protease activity was identified. Compound **35** corresponds to 4-(3-(4-allyl-2-methoxyphenoxy)propyl)-1-(2-bromobenzyl)-1*H*-1,2,3-triazole. In addition, molecular docking calculations suggested that the inhibition mechanism relies on interactions between His^51^, Thr^134^, Ser^135^ and Tyr^161^. The virucidal assay with DENV-1-4 strains indicates that compound **35** is a promising lead compound for antiviral activity against *Flavivirus*. Taken together, our results provide insightful information for further development of *Flavivirus* protease inhibitors via rational drug design. Efforts towards this end are under way in our laboratories.

## Supporting information

S1 File **Table A. Docking of the compounds 23 to 49 with WNV protease.** The entire protease is shown in lilac, highlighting residues Ser135 (dark blue), His51 (orange), and Asp75 (green). The compound is show in red. FigsA-DP Figs. Selected IR and NMR spectra.
(PDF)

S1 Fig Inhibition mechanisms observed between WNV NS2B-NS3 protease cocrystallized peptide-like molecules obtained from Protein Data Bank. A. 2YOL; B. 3E90; C. 5IDK; and D. 2FP7.
(TIFF)
