# Supplementary material for: Discovery of novel West Nile Virus protease inhibitor based on isobenzonafuranone and triazolic derivatives of eugenol and indan-1,3-dione scaffolds
Source: PLoS One. 2019 Sep 26;14(9):e0223017. doi: 10.1371/journal.pone.0223017 (PMC6762200; doi:10.1371/journal.pone.0223017)
Supplement: S1 File — Table A. Docking of the compounds 23 to 49 with WNV protease. The entire protease is shown in lilac, highlighting residues Ser135 (dark blue), His51 (orange), and Asp75 (green). The compound is show in red. FigsA-DP Figs. Selected IR and NMR spectra. (PDF) [file pone.0223017.s001.pdf]

SUPPLEMENTARY MATERIAL

Discovery of novel West Nile Virus protease inhibitor based on isobenzonafuranone and triazolic derivatives of eugenol and indan-1,3-dione scaffolds

André S. de Oliveira [1,2¶], Poliana Aparecida Rodrigues Gazolla[2¶], Ana Flávia C. da S. Oliveira[1,2¶], Wagner Luiz Pereira[2], Lívia Cristina de Souza Viol[2], Angélica Faleiros da Silva Maia[2], Edjon G. Santos[1], Ítalo Esposti P. da Silva[1], Tiago Antônio de Oliveira Mendes[4], Adalberto Manoel da Silva[3], Roberto Sousa Dias[1], Cynthia Canedo da Silva[1], Marcelo D. Polêto[1], Róbson R. Teixeira[3*], and Sergio O. de Paula[*1]

[1] Departamento de Biologia Geral, Universidade Federal de Viçosa, Av. P.H. Rolfs, S/N, Viçosa-MG (Brazil).

[2] Instituto Federal de Educação, Ciência e Tecnologia do Norte de Minas Gerais, Fazenda Biribiri, S/N, Diamantina-MG (Brazil).

[3] Departamento de Química, Universidade Federal de Viçosa, Av. P.H. Rolfs, S/N, Viçosa-MG (Brazil).

[4] Departamento de Bioquímica Biologia Molecular, Universidade Federal de Viçosa, Av. P.H. Rolfs, S/N, Viçosa-MG (Brazil).

[5] Instituto Federal de Educação, Ciência e Tecnologia Catarinense, Campus Araquari Rodovia BR 280, km 27 - Araquari-SC (Brazil).

* Corresponding authors

depaula@ufv.br (SOP)

robsonr.teixeira@ufv.br (RRT)

¶These authors contributed equally.

CONTENTS

Page

Table A…………………………………………………….. 3
Selected IR AND NMR SPECTRA……………………. 12
Figure S1...73

Table A: Docking of the compounds 23 to 49 with WNV protease					
	Ligation				
**Comp.**	**Ligation type**	**Description**	**Interaction distance (Å)**	**aa**	**Docking/Ligation Energy**
**23**	Hydrogen Bond	OH of the amino acid with the oxygen of the carbonic chain of the molecule	2,45	Thr^{132}	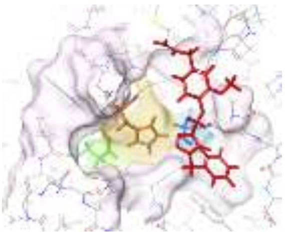 EnergyEnergy: -2,318
**24**		No chemical interaction predicted			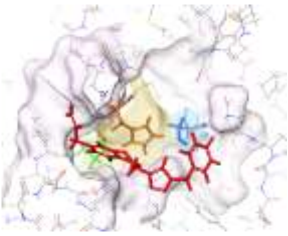 Energy: -1,081
**25**	Hydrogen Bond	NH_2 of the amino acid with the oxygen of the carbonic chain of the molecule	1,92	Asn^{84}	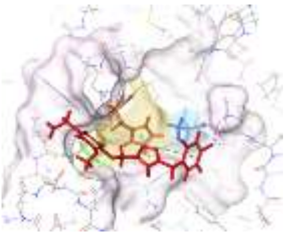 Energy: +2,058
**25**	Halogen Bond	OH of the amino acid with the chlorine of the molecule	3,26	Thr^{134}	
**25**	Halogen Bond	NH^+ of the amino acid with the iodine of the molecule	2,62	Gly^{133}	
**25**	Halogen Bond	NH^+ of the amino acid with the iodine of the molecule	3,42	Thr^{134}	
**25**	cation-π	NH^+ of the amino acid ring with the phenyl ring of the molecule	6,39	**His^{51}**	
**25**	π-π stacking	Amino acid ringwith the triazole ring of the molecule	4,11	**His^{51}**	

Table A: Docking of the compounds 23 to 49 with WNV protease					
	Ligation				
**Comp.**	**Ligation type**	**Description**	**Interaction distance (Å)**	**aa**	**Docking/Ligation Energy**
**26**	No chemical interaction predicted				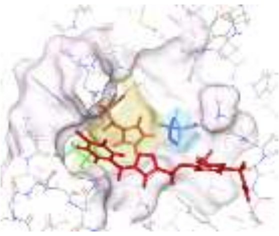 Energy: -0,429
**27**	cation-π	NH^+ of the amino acid ring with the triazole ring of the molecule	5,43	His^{51}	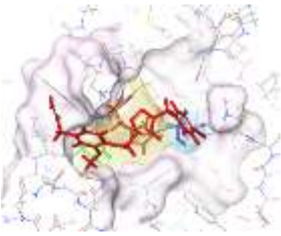 Energy: -0,055
**27**	cation-π	NH^+ of the amino acid ring with the phenyl ring of the molecule	6,20	His^{51}	
**27**	π-π stacking	Amino acid ringwith the phenyl ring of the molecule	5,15	His^{51}	
**27**	Halogen Bond	NH^+ of the amino acid with the iodine of the molecule	2,43	Ser^{135}	
**27**	Halogen Bond	NH^+ of the amino acid with the iodine of the molecule	2,82	Thr^{134}	
**28**	Hydrogen Bond	OH of the amino acid with the oxygen of the carbonic chain of the molecule	2,24	Thr^{132}	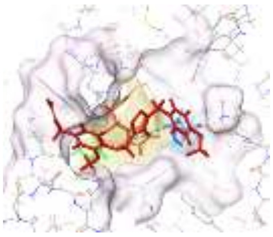 Energy: -2,732
**28**	Hydrogen Bond	O^- of the amino acid with the NO_2H_2 of the molecule	1,89	Asp^{129}	
**28**	Hydrogen Bond	O^- of the amino acid with the NO_2H_2 of the molecule	2,07	Asp^{129}	
**28**	Hydrogen Bond	NH^+ of the amino acid with the nitrogen of triazole ring of the molecule	2,46	Ser^{135}	
**28**	π-π stacking	Amino acid ring with the phenyl ring of the molecule	4,39	Tyr^{161}	
**29**	Hydrogen Bond	NH^+ of the amino acid wit the OCH_3 of the phenyl ring in the molecule	1,98	Gly^{133}	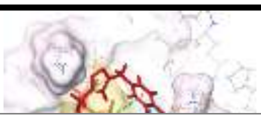

Table A: Docking of the compounds 23 to 49 with WNV protease

Comp.	Ligation: Ligation type	Ligation: Description	Ligation: Interaction distance (Å)	Ligation: aa	Docking/Ligation Energy
**29**	cation-π	NH^+ of the amino acid ring with the triazole ring of the molecule	6,13	**His^{51}**	Energy: -3,991
**29**	cation-π	NH^+ of the amino acid ring with the triazole ring of the molecule	3,81	**His^{51}**	
**29**	π-π stacking	Amino acid ring with the phenyl ring of the molecule	5,04	**His^{51}**	
**29**	π-π stacking	Amino acid ring with the eugenol ring of the molecule	3,96	**His^{51}**	
**30**	Hydrogen Bond	NH^+ of the amino acid with the OF_3 of the phenyl ring of the molecule	1,96	Gly^{133}	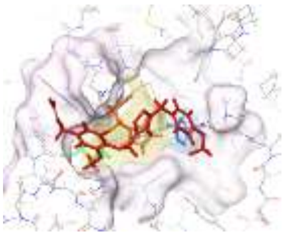 Energy: -1,459
**30**	Hydrogen Bond	NH_2 of the amino acid with the OCH_3 of the molecule	2,08	Asn^{84}	
**30**	cation-π	NH^+ of the amino acid ring with the phenyl ring of the molecule	6,01	**His^{51}**	
**30**	π-π stacking	Amino acid ringwith the phenyl ring of the molecule	4,93	**His^{51}**	
**31**	π-π stacking	Ring of the amino acid with the eugenol ring of the molecule	5,08	Tyr^{161}	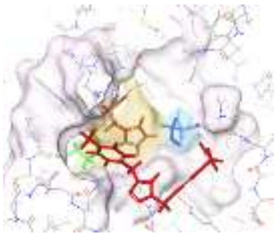 Energy: +1,993
**32**	No chemical interaction predicted				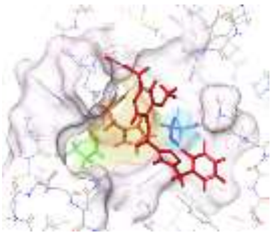 Energy: +2,136

Table A: Docking of the compounds 23 to 49 with WNV protease

Comp.	Ligation type	Description	Interaction distance (Å)	aa	Docking/Ligation Energy
**33**	Hydrogen Bond	NH^+ of the amino acid with the OCH_3 of the eugenol of the molecule	1,80	Ile^{155}	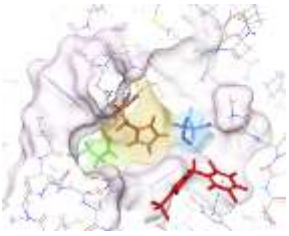 Energy: -1,473
**33**	Halogen Bond	OH of the amino acid with the iodine of the molecule	2,68	Ser^{135}	
**33**	π-π stacking	Ring of the amino acid with the phenyl ring of the molecule	3,80	Tyr^{161}	
**34**	Hydrogen Bond	NH_2 of the amino acid with the oxygen of the carbonic chain of the molecule	1,84	Asn^{84}	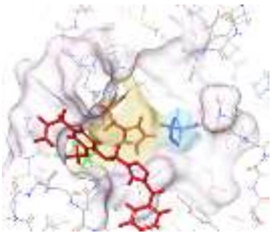 Energy: +1,863
**35**	cation-π	NH^+ of the amino acid ring with the triazole ring of the molecule	5,88	His^{51}	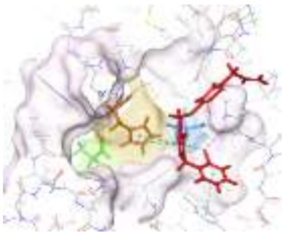 Energy: -2,343
**35**	π-π stacking	Amino acid ring with the triazole ring of the molecule	5,04	His^{51}	
**35**	π-π stacking	Amino acid ring with the phenyl ring of the molecule	4,22	Tyr^{161}	
**35**	Halogen Bond	OH of the amino acid with Bromo of the molecule	2,10	Ser^{135}	
**35**	Halogen Bond	NH^+ do of the amino acid with Bromo of the molecule	3,25	Thr^{134}	
**36**	Hydrogen Bond	NH^+ of the amino acid with the nitrogen of triazole ring of the molecule	2,70	Gly^{153}	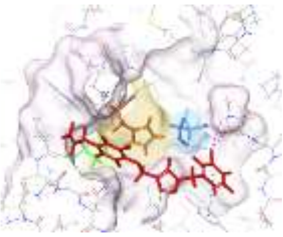 Energy: +1,825
**36**	π-π stacking	Amino acid ringwith the phenyl ring of the molecule	4,30	Tyr^{161}	
**36**	Halogen Bond	NH^+ of the amino acid with Bromo of the molecule	3,19	Gly^{133}	
**36**	Halogen Bond	NH^+ of the amino acid with Bromo of the molecule	3,01	Thr^{134}	
**36**	Halogen Bond	OH of the amino acid with Bromo of the molecule	2,56	Ser^{135}	

Table A: Docking of the compounds 23 to 49 with WNV protease					
	Ligation				
**Comp.**	**Ligation type**	**Description**	**Interaction distance (Å)**	**aa**	**Docking/Ligation Energy**
**37**	cation-π	NH_3^+ do of the amino acid with the eugenol ring of the molecule	5,31	Lys^{54}	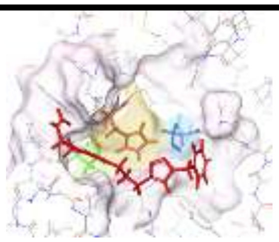 Energy: +2,012
**38**	cation-π	NH_3^+ of the amino acid with the eugenol ring of the molecule	4,79	Lys^{54}	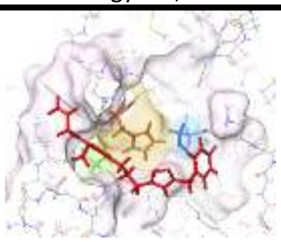 Energy: +1,608
**39**	Hydrogen Bond	OH of the amino acid with the OCH_3 of the eugenol of the molecule	2,32	Thr^{132}	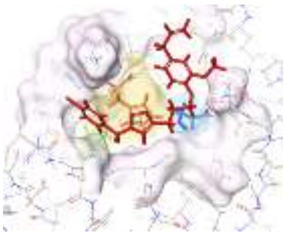 Energy: -2,863
**39**	cation-π	NH^+ of the amino acid ring with the triazole ring of the molecule	3,82	**His^{51}**	
**39**	π-π stacking	Amino acid ring with the triazole ring of the molecule	3,51	**His^{51}**	
**40**	Hydrogen Bond	NH^+ of the amino acid with the OCH_3 of the eugenol of the molecule	2,77	**His^{51}**	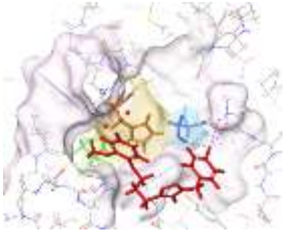 Energy: +1,347
**41**	Hydrogen Bond	NH^+ of the amino acid with the oxigênio da cadeia carbônica of the molecule	2,48	Gly^{133}	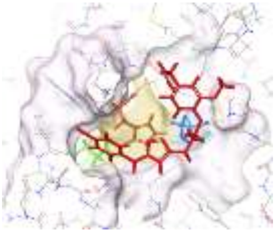 Energy: -2,436
**41**	Hydrogen Bond	OH of the amino acid with the OCH_3 of the eugenol of the molecule	2,48	Thr^{132}	
**42**	Hydrogen Bond	O^- of the amino acid with the NO_2H_2 of the molecule	2,00	Asp^{129}	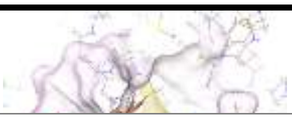

Table A: Docking of the compounds 23 to 49 with WNV protease

Comp.	Ligation: Ligation type	Ligation: Description	Ligation: Interaction distance (Å)	Ligation: aa	Docking/Ligation Energy
**42**	Hydrogen Bond	O^- of the amino acid with the NO_2H_2 of the molecule	1,79	Asp^{129}	Energy: +0,463
**42**	cation-π	NH^+ of the amino acid ring with the triazole ring of the molecule	4,49	**His^{51}**	
**42**	π-π stacking	Amino acid ringwith the triazole ring of the molecule	3,96	**His^{51}**	
**43**	Hydrogen Bond	NH^+ of the amino acid with the nitrogen of triazole ring of the molecule	2,26	Gly^{133}	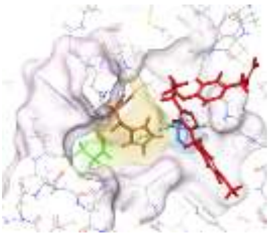 Energy: +0,304
**43**	cation-π	NH^+ of the amino acid ring with the triazole ring of the molecule	6,25	**His^{51}**	
**43**	π-π stacking	Amino acid ringwith the triazole ring of the molecule	5,15	**His^{51}**	
**44**	Hydrogen Bond	NH^+ of the amino acid with the OF_3 of the molecule	2,07	Gly^{133}	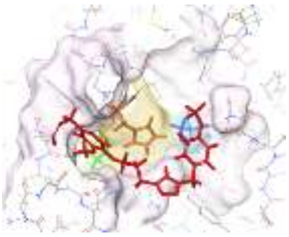 Energy: +0,877
**44**	Hydrogen Bond	NH_2 of the amino acid with thexigênio da cadeia carbônica of the molecule	1,73	Asn^{84}	
**45**	Hydrogen Bond	NH^+ of the amino acid with the nitrogênio da anel triazólico of the molecule	2,62	Gly^{133}	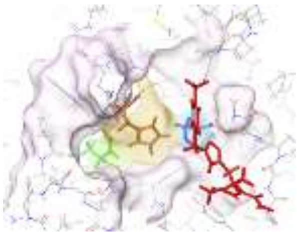 Energy: +0,392
**46**	Hydrogen Bond	NH^+ of the amino acid with the oxygen of the carbonic chain of the molecule	2,63	Gly^{133}	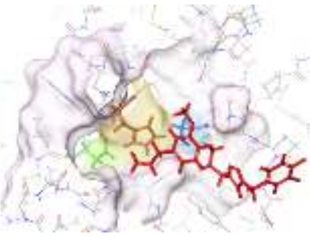 Energy: +2,811

Table A: Docking of the compounds 23 to 49 with WNV protease					
	Ligation				
**Comp.**	**Ligation type**	**Description**	**Interaction distance (Å)**	**aa**	**Docking/Ligation Energy**
**47**	Hydrogen Bond	NH$_2$ of the amino acid with the oxygen of the carbonic chain of the molecule	2,03	Asn^{84}	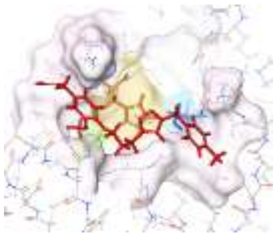
**47**	π-π stacking	Amino acid ring with the triazole ring of the molecule	3,64	**His^{51}**	Energy: -3,613
**48**	cation-π	NH$^+$ of the amino acid ring with the triazole ring of the molecule	5,26	**His^{51}**	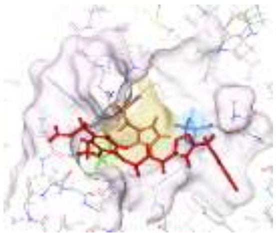
**48**	Halogen Bond	OH of the amino acid with Bromo of the molecule	2,13	**Ser^{135}**	Energy: +0,947
**49**	π-π stacking	Amino acid ring with the phenyl ring of the molecule	4,11	Tyr^{161}	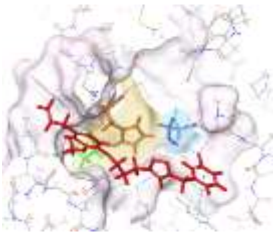
**49**	Halogen Bond	OH of the amino acid with Bromo of the molecule	2,49	**Ser^{135}**	
**49**	Halogen Bond	NH$^+$ of the amino acid with Bromo of the molecule	3,41	**Ser^{135}**	Energy: +0,969

SELECTED IR AND NMR SPECTRA

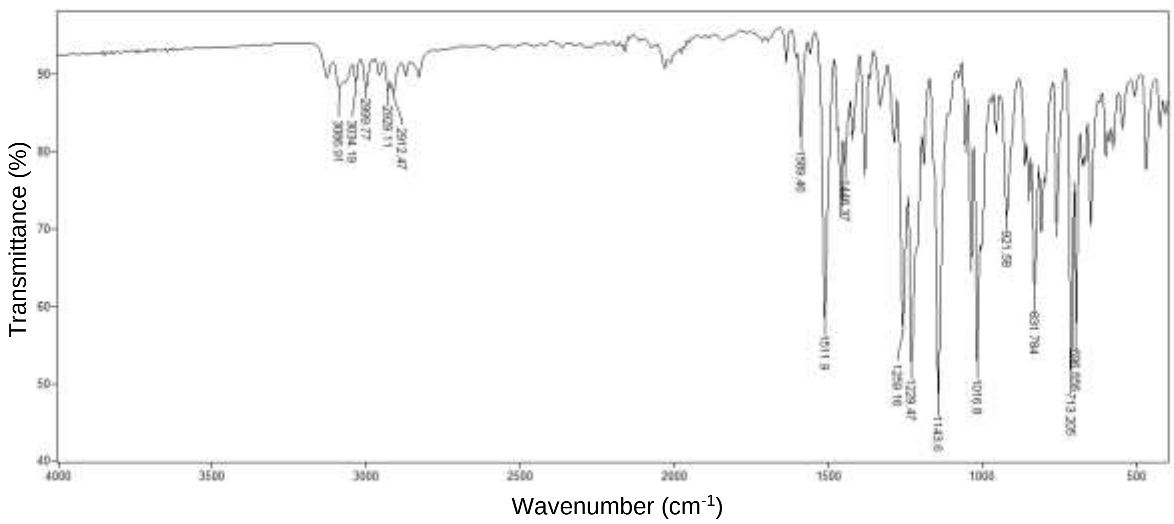

Fig. A. IR spectrum (ATR) of 4-((4-allyl-2-methoxyphenoxy)methyl)-1-benzyl-*1H*-1,2,3-triazole (**23**).

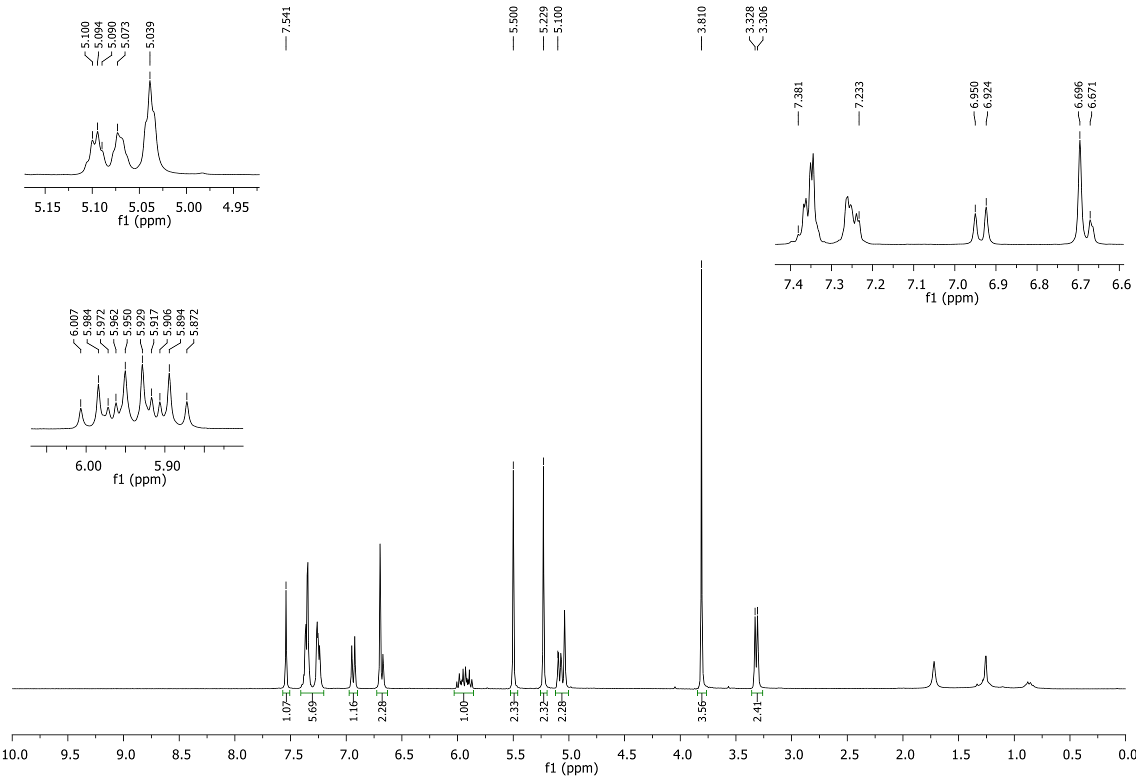

Fig. B. ^{1}H NMR spectrum (300 MHz, $CDCl_3$) of 4-((4-allyl-2-methoxyphenoxy)methyl)-1-benzyl-*1H*-1,2,3-triazole (**23**).

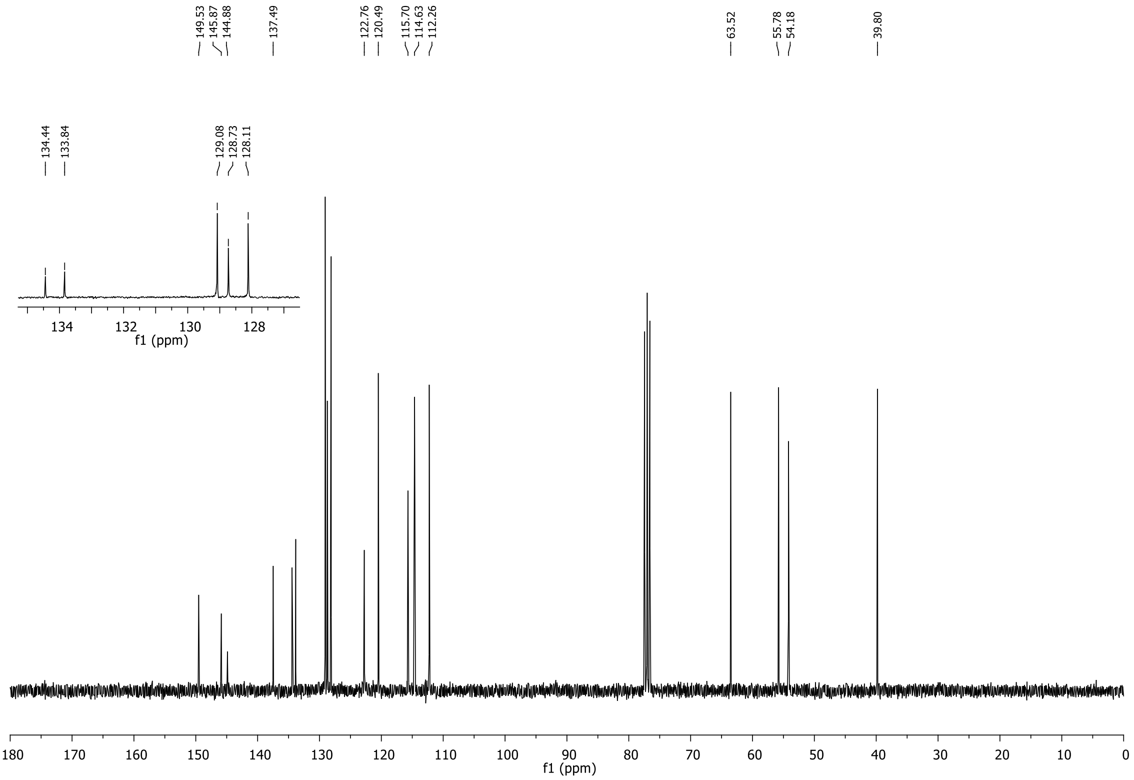

Fig. C. ^{13}C NMR spectrum (75 MHz, $CDCl_3$) of 4-((4-allyl-2-methoxyphenoxy)methyl)-1-benzyl-*1H*-1,2,3-triazole (**23**).

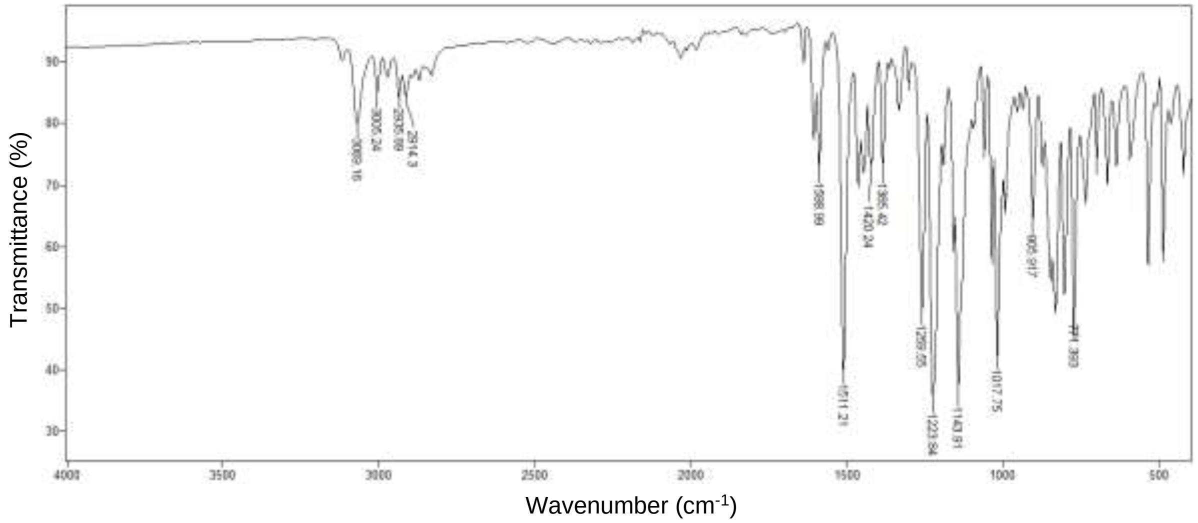

Fig. D. IR spectrum (ATR) of 4-((4-allyl-2-methoxyphenoxy)methyl)-1-(4-fluorobenzyl)-*1H*-1,2,3-triazole (**24**).

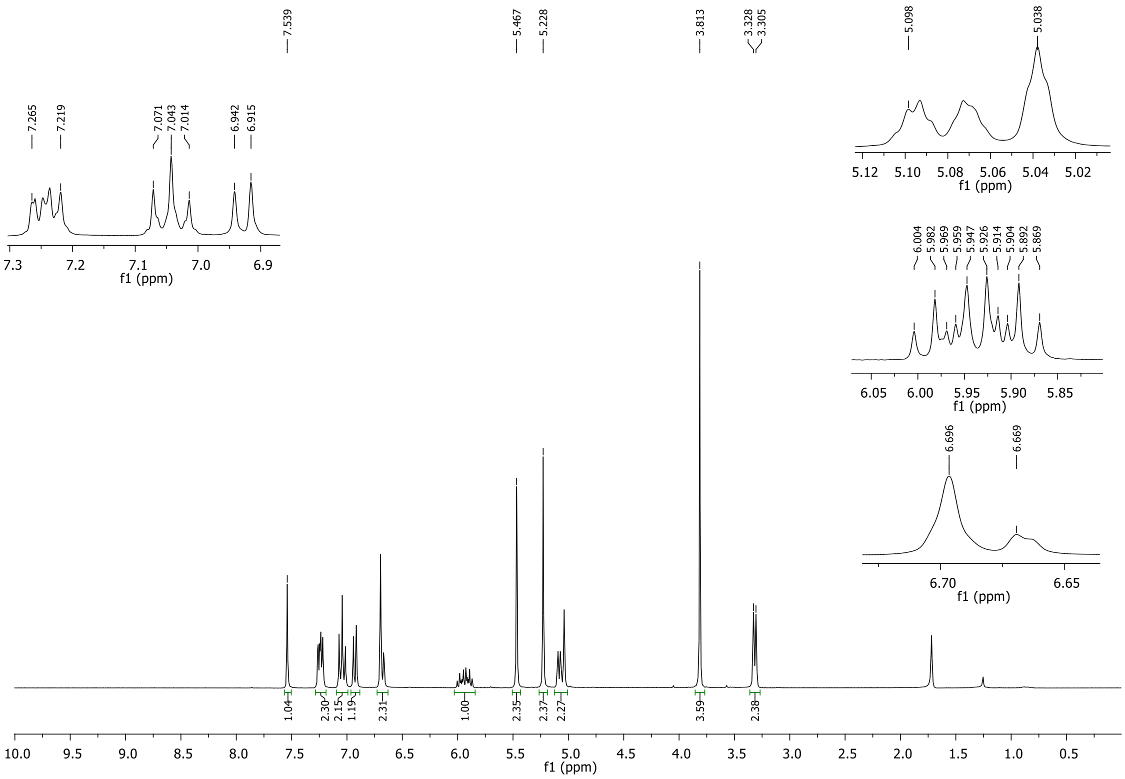

Fig. E. ^{1}H NMR spectrum (300 MHz, $CDCl_3$) of 4-((4-allyl-2-methoxyphenoxy)methyl)-1-(4-fluorobenzyl)-*1H*-1,2,3-triazole (**24**).

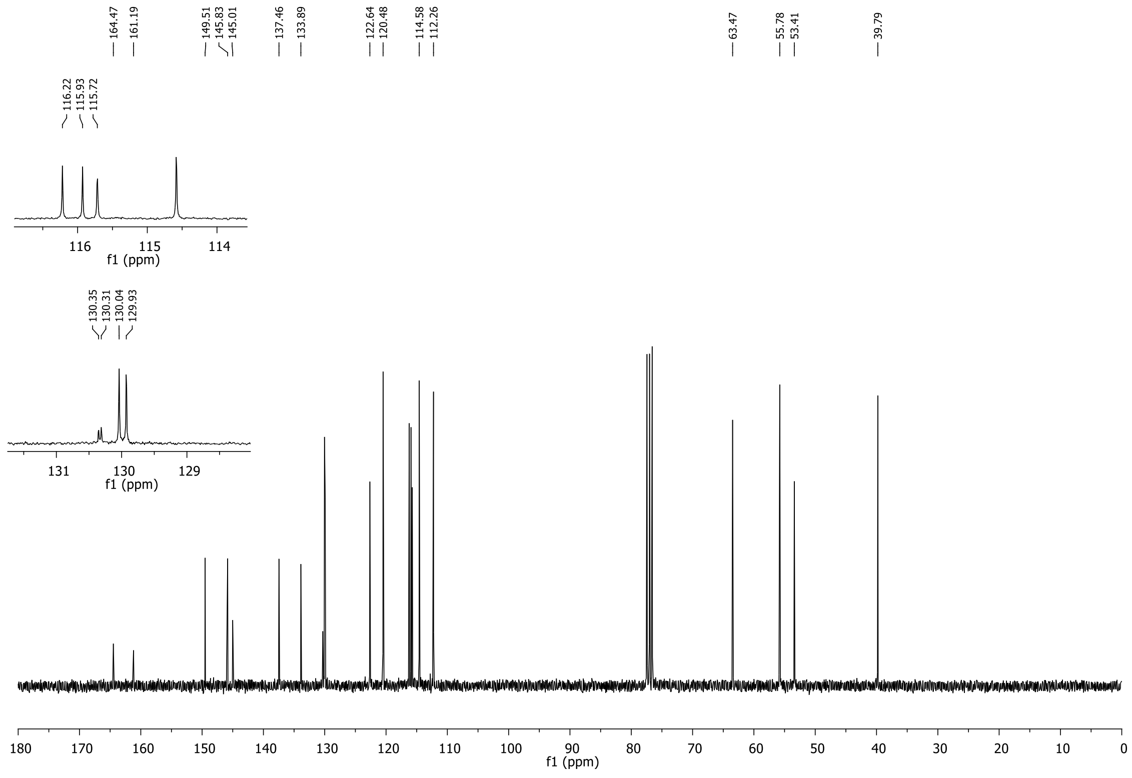

Fig. F. ^{13}C NMR spectrum (75 MHz, $CDCl_3$) of 4-((4-allyl-2-methoxyphenoxy)methyl)-1-(4-fluorobenzyl)-*1H*-1,2,3-triazole (**24**).

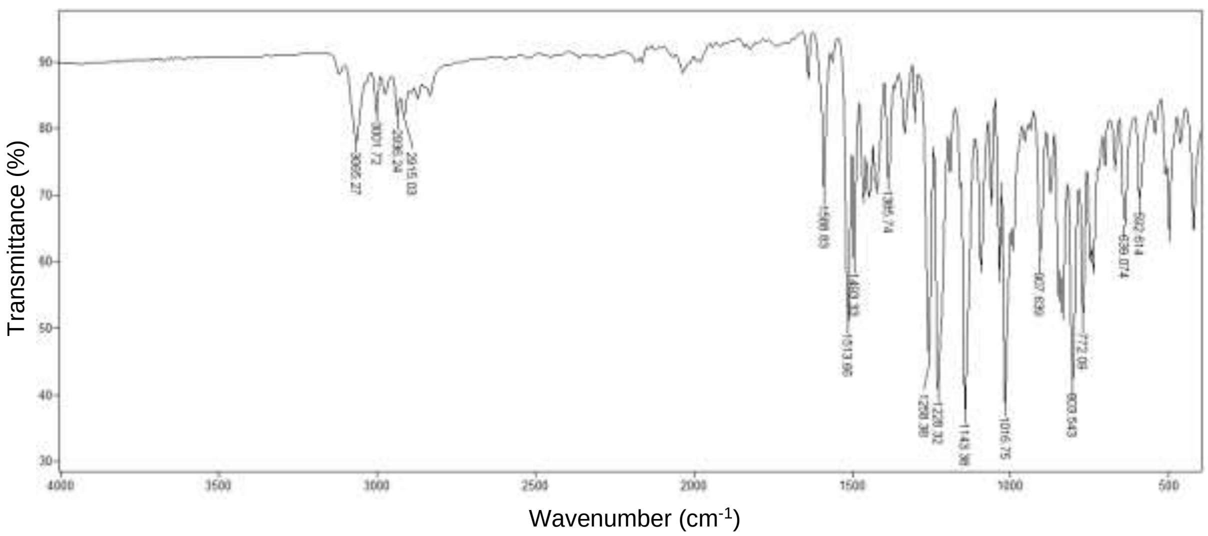

Fig. G. IR spectrum (ATR) of 4-((4-allyl-2-methoxyphenoxy)methyl)-1-(4-chlorobenzyl)-*1H*-1,2,3-triazole (**25**).

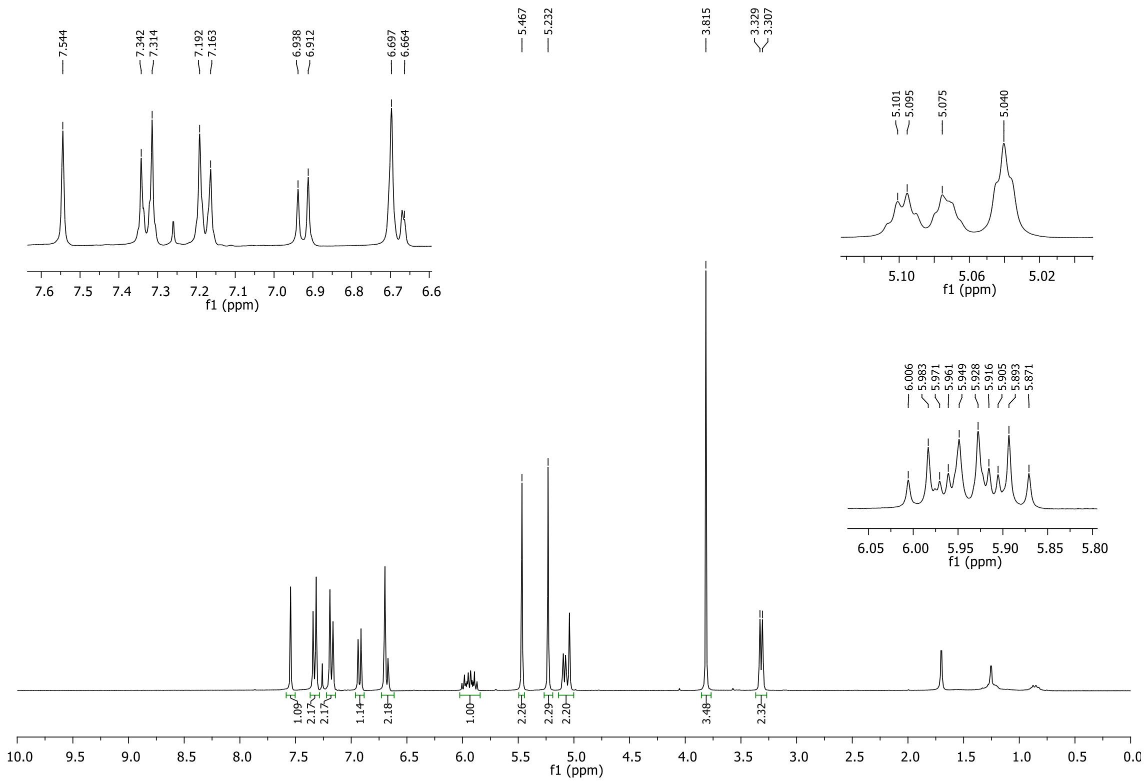

Fig. H. ^{1}H NMR spectrum (300 MHz, $CDCl_3$) of 4-((4-allyl-2-methoxyphenoxy)methyl)-1-(4-chlorobenzyl)-*1H*-1,2,3-triazole (**25**).

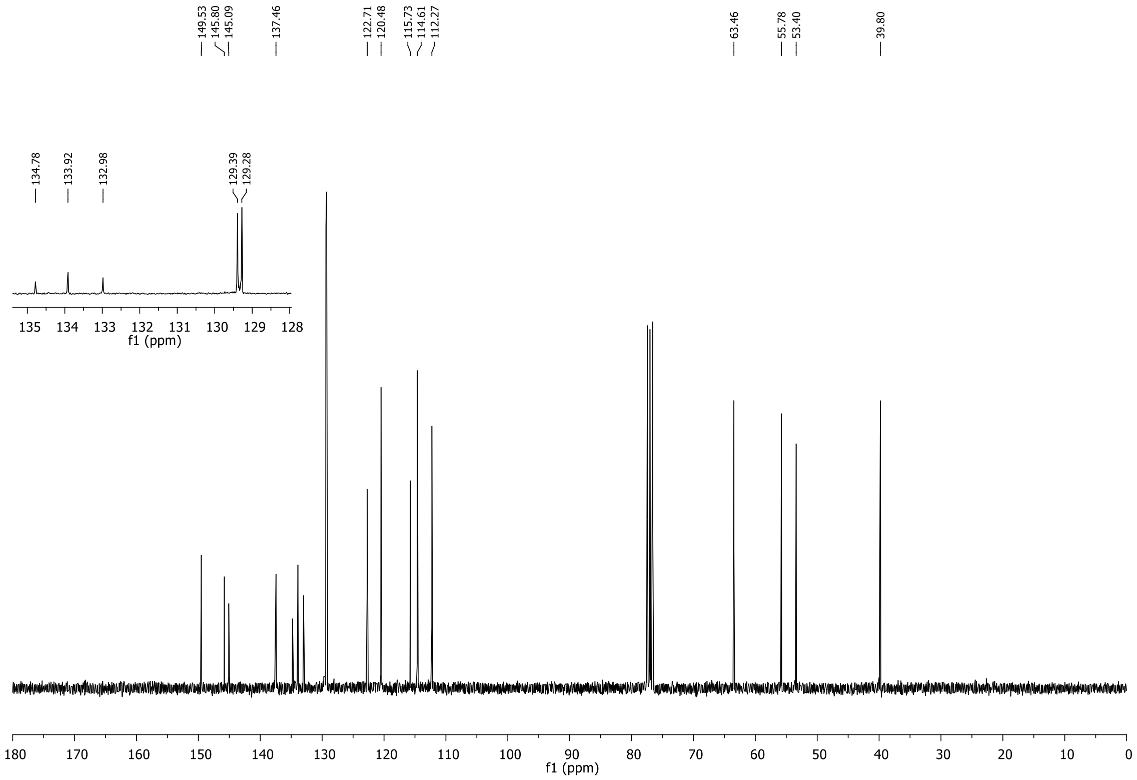

Fig. I. ^{13}C NMR spectrum (75 MHz, $CDCl_3$) of 4-((4-allyl-2-methoxyphenoxy)methyl)-1-(4-chlorobenzyl)-*1H*-1,2,3-triazole (**25**).

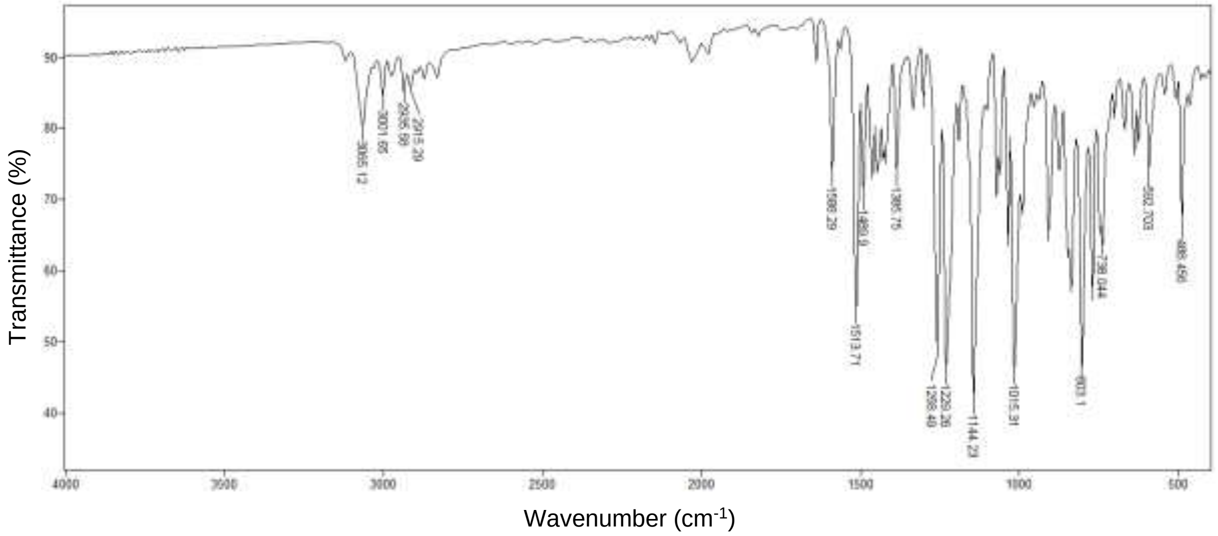

Fig. J. IR spectrum (ATR) of 4-((4-allyl-2-methoxyphenoxy)methyl)-1-(4-bromobenzyl)-*1H*-1,2,3-triazole (**26**).

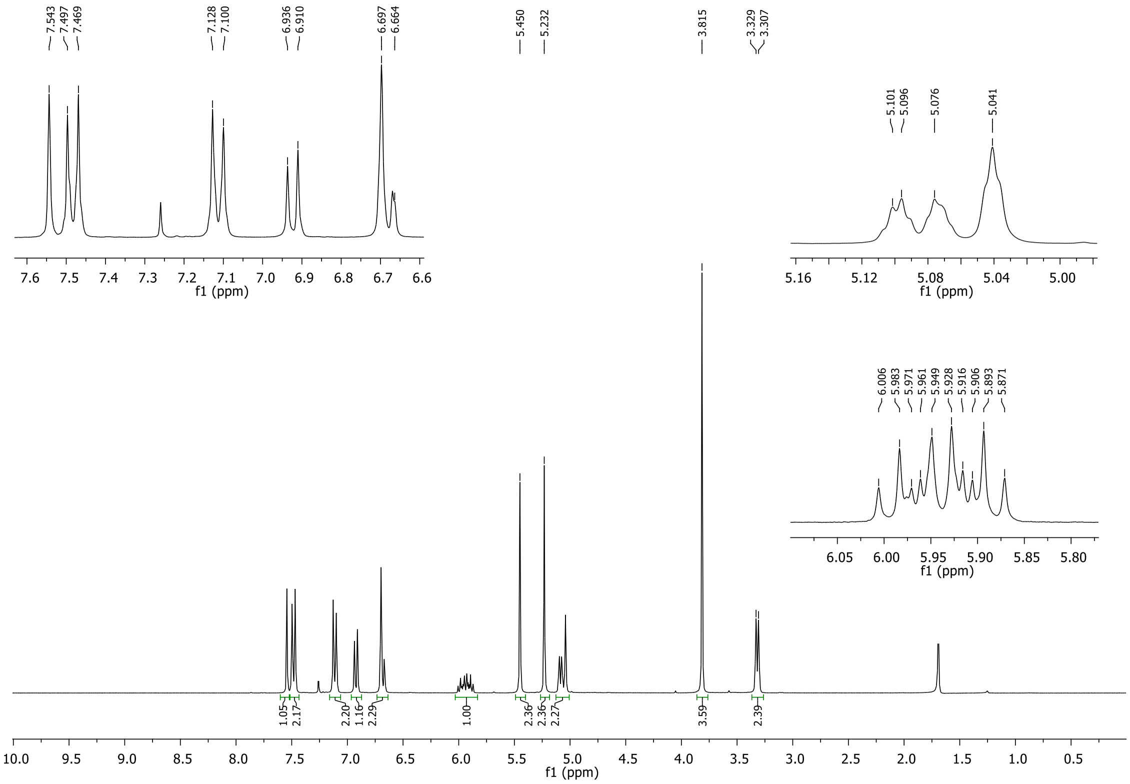

Fig. K. ^{1}H NMR spectrum (300 MHz, $CDCl_3$) of 4-((4-allyl-2-methoxyphenoxy)methyl)-1-(4-bromobenzyl)-*1H*-1,2,3-triazole (**26**).

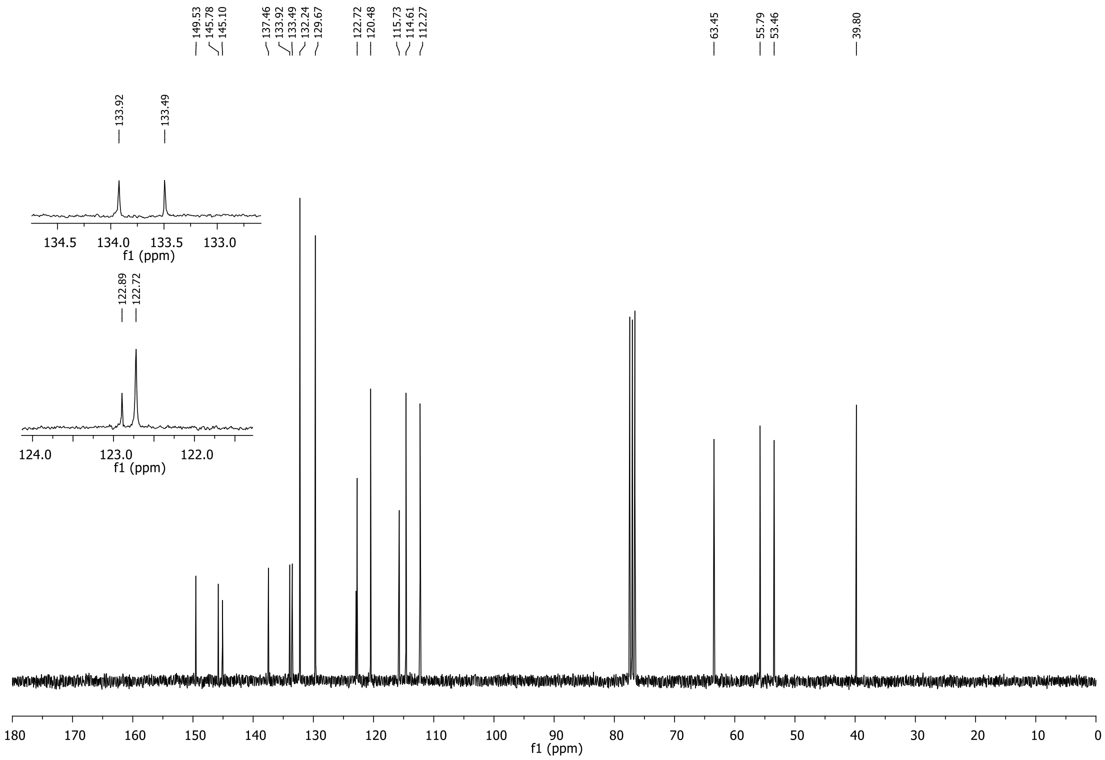

Fig. L. ^{13}C NMR spectrum (75 MHz, $CDCl_3$) of 4-((4-allyl-2-methoxyphenoxy)methyl)-1-(4-bromobenzyl)-*1H*-1,2,3-triazole (**26**).

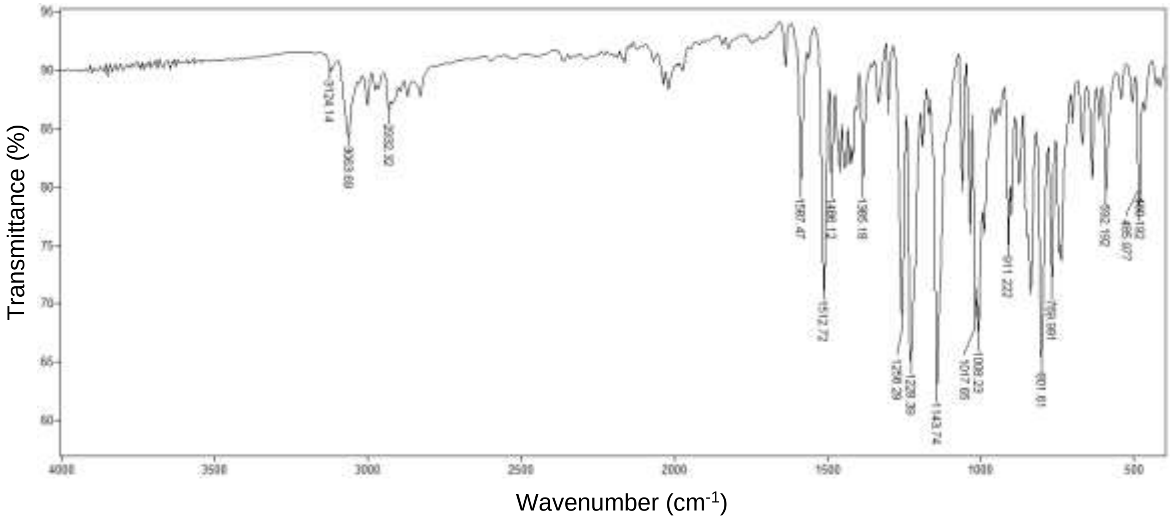

Fig. M. IR spectrum (ATR) of 4-((4-allyl-2-methoxyphenoxy)methyl)-1-(4-iodobenzyl)-*1H*-1,2,3-triazole (**27**).

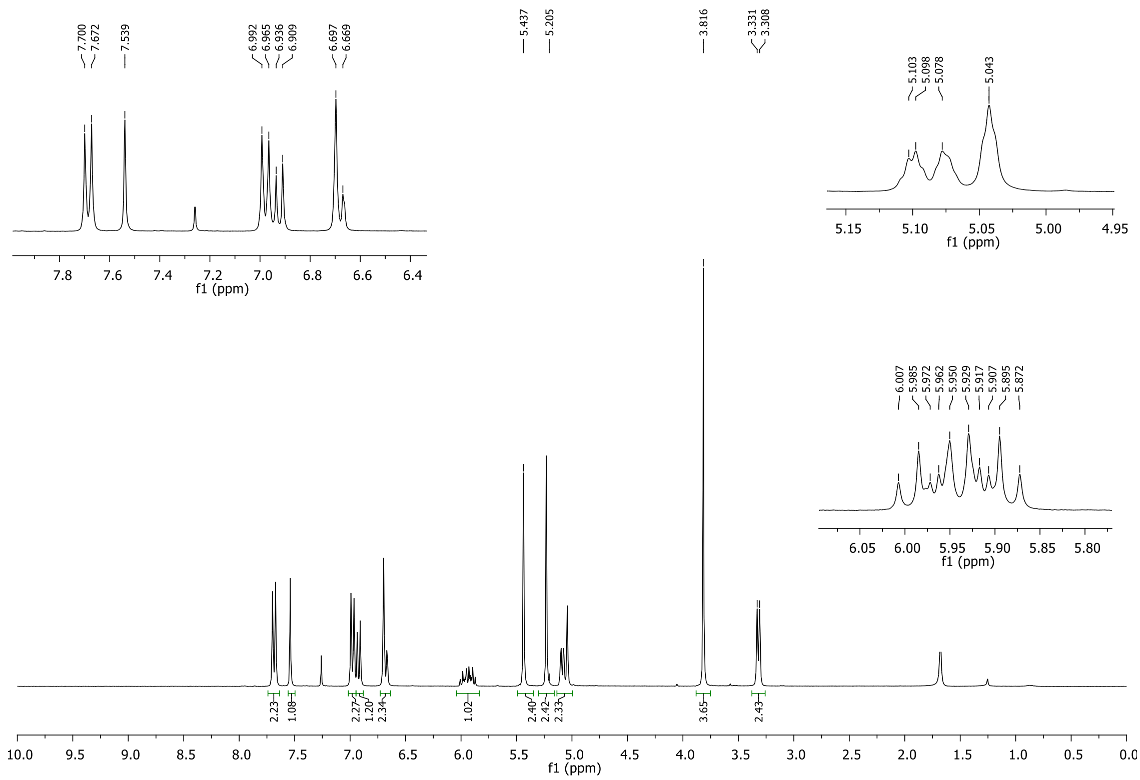

Fig. N. ^{1}H NMR spectrum (300 MHz, $CDCl_3$) of 4-((4-allyl-2-methoxyphenoxy)methyl)-1-(4-iodobenzyl)-*1H*-1,2,3-triazole (**27**).

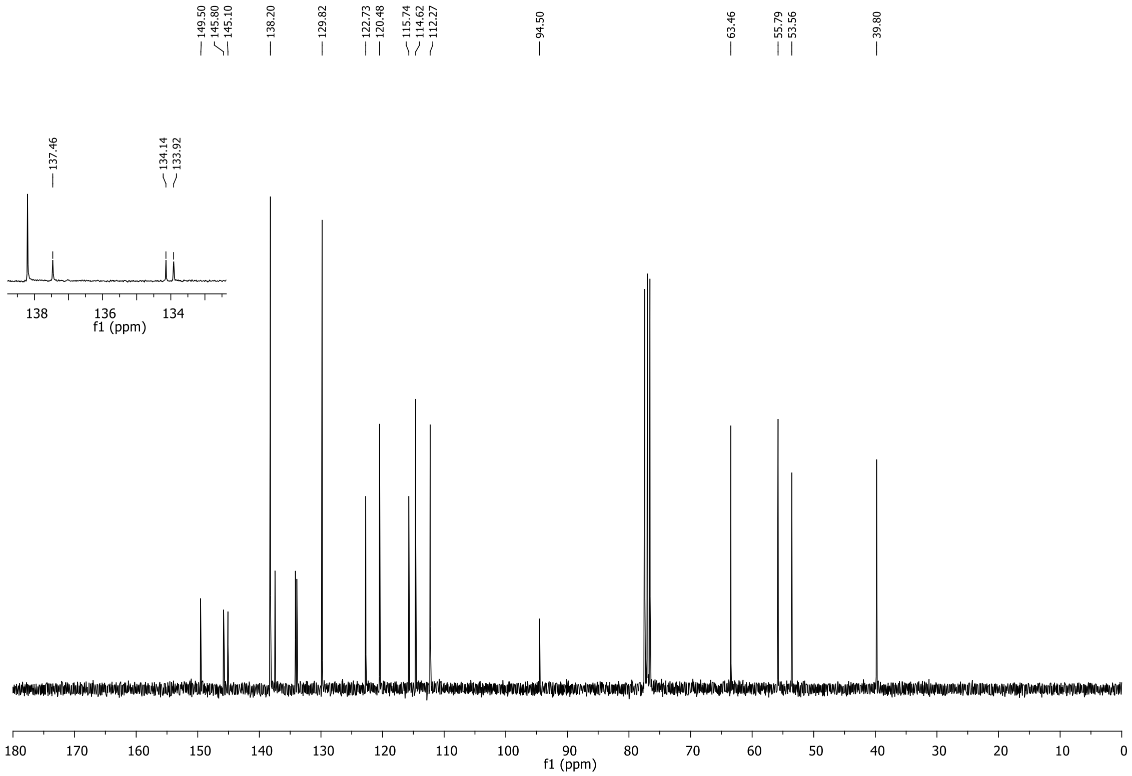

Fig. O. ^{13}C NMR spectrum (75 MHz, $CDCl_3$) of 4-((4-allyl-2-methoxyphenoxy)methyl)-1-(4-iodobenzyl)-*1H*-1,2,3-triazole (**27**).

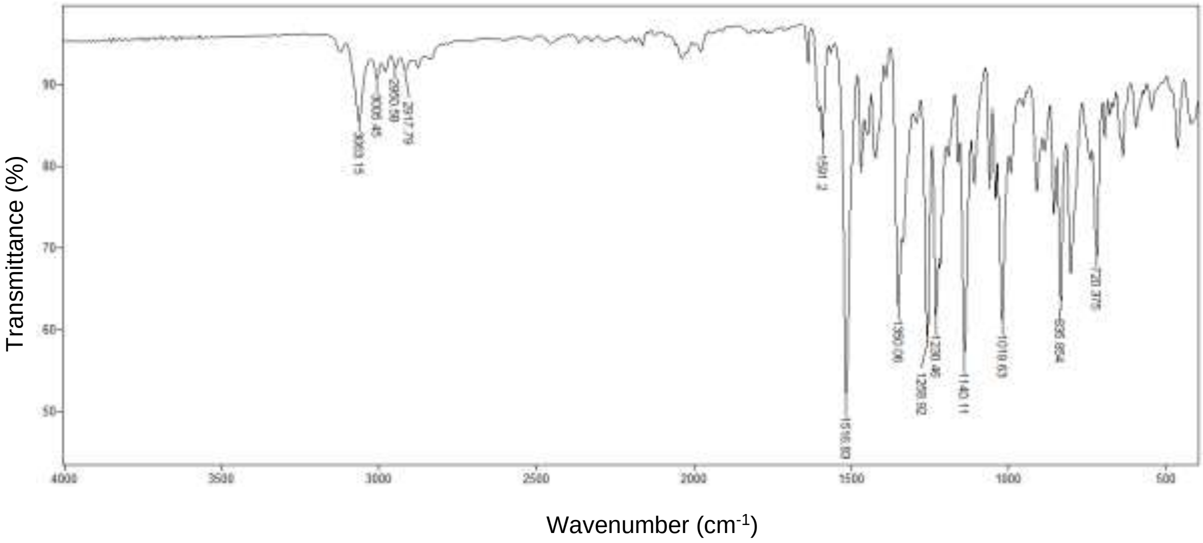

Fig. P. IR spectrum (ATR) of 4-((4-allyl-2-methoxyphenoxy)methyl)-1-(4-nitrobenzyl)-*1H*-1,2,3-triazole (**28**).

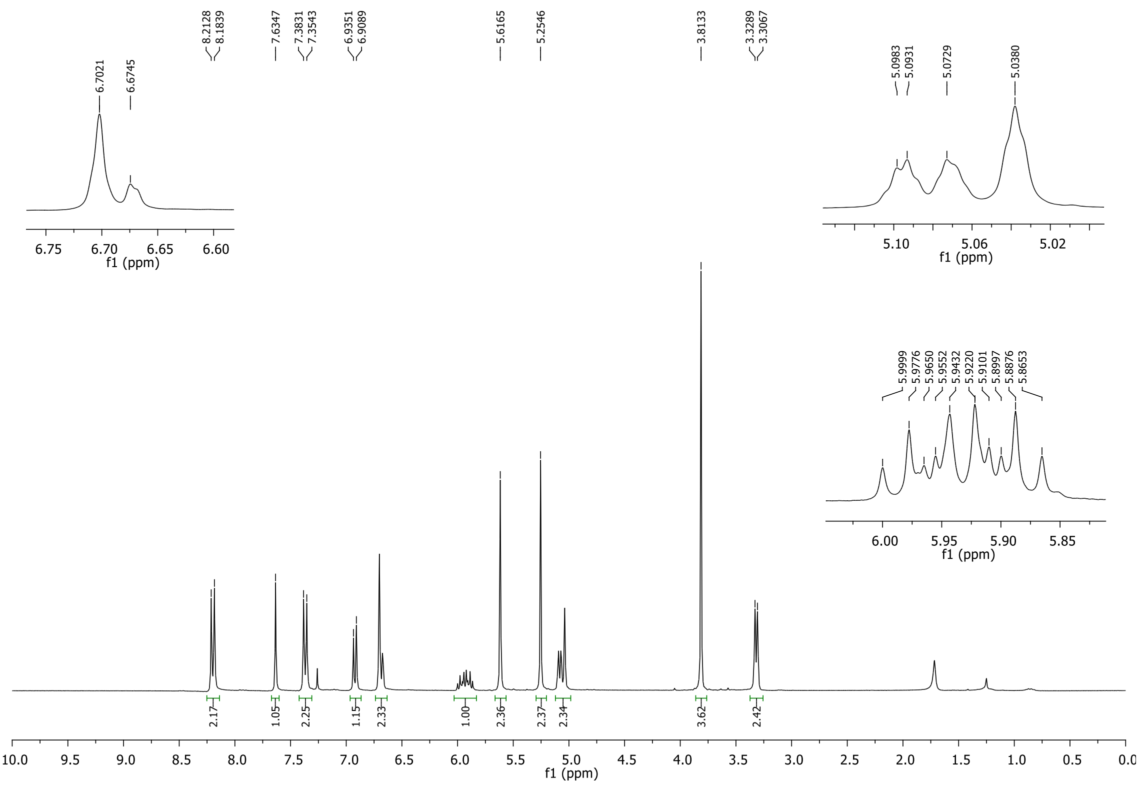

Fig. Q. ^{1}H NMR spectrum (300 MHz, $CDCl_3$) of 4-((4-allyl-2-methoxyphenoxy)methyl)-1-(4-nitrobenzyl)-*1H*-1,2,3-triazole (**28**).

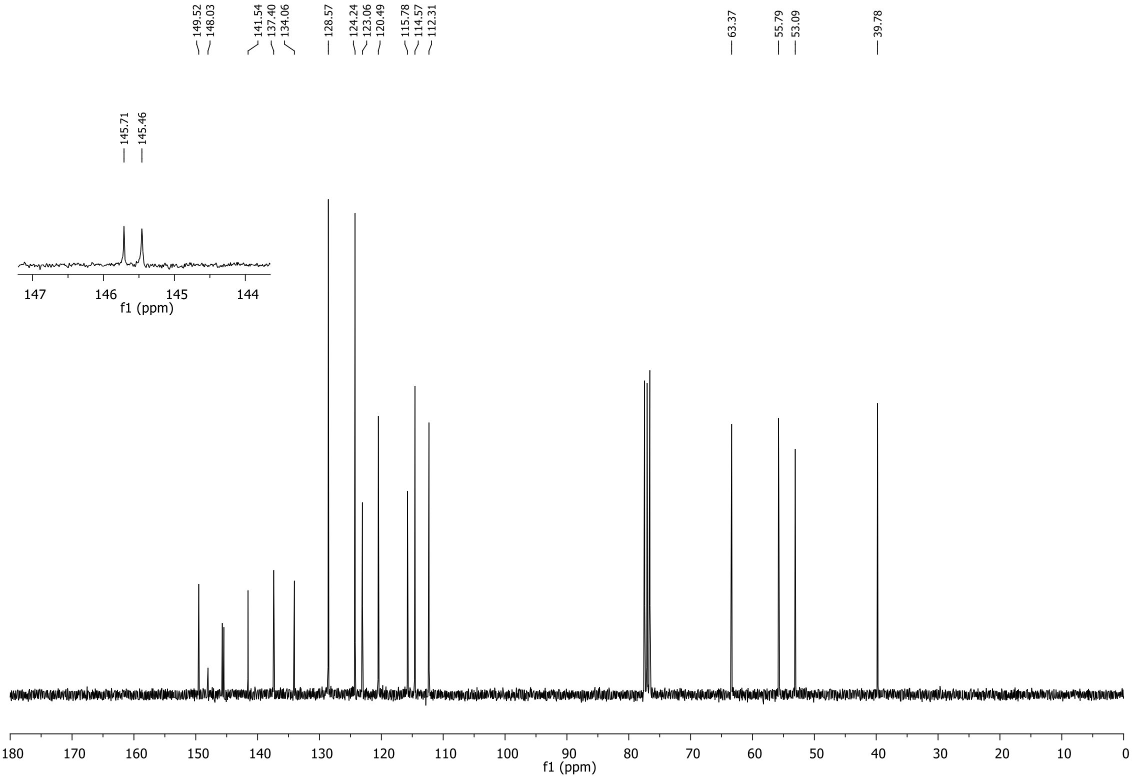

Fig. R. ^{13}C NMR spectrum (75 MHz, $CDCl_3$) of 4-((4-allyl-2-methoxyphenoxy)methyl)-1-(4-nitrobenzyl)-*1H*-1,2,3-triazole (**28**).

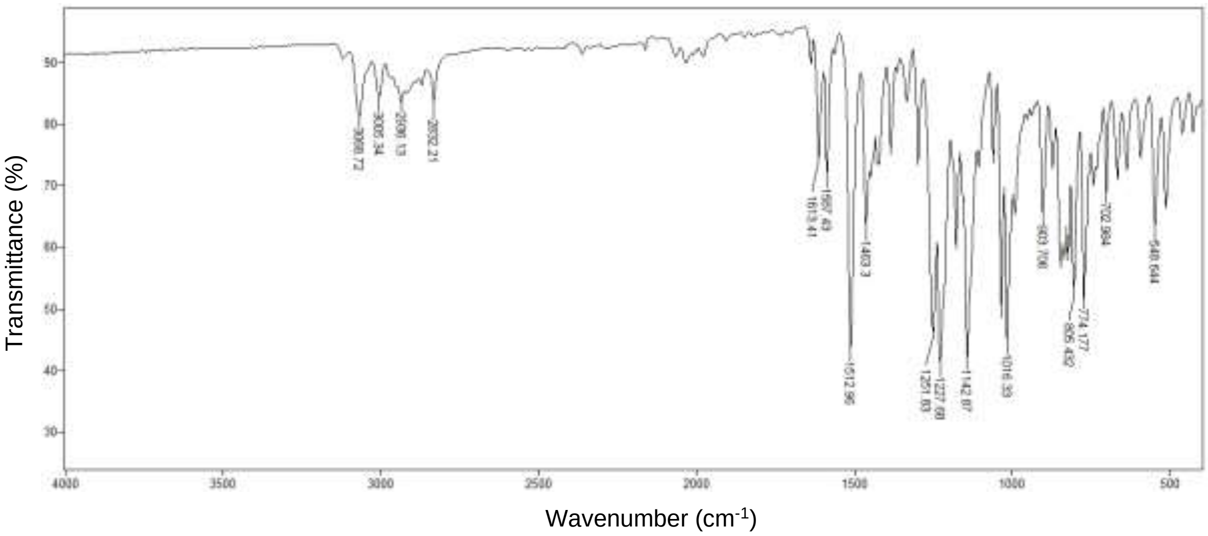

Fig. S. IR spectrum (ATR) of 4-((4-allyl-2-methoxyphenoxy)methyl)-1-(4-methoxybenzyl)-*1H*-1,2,3-triazole (**29**).

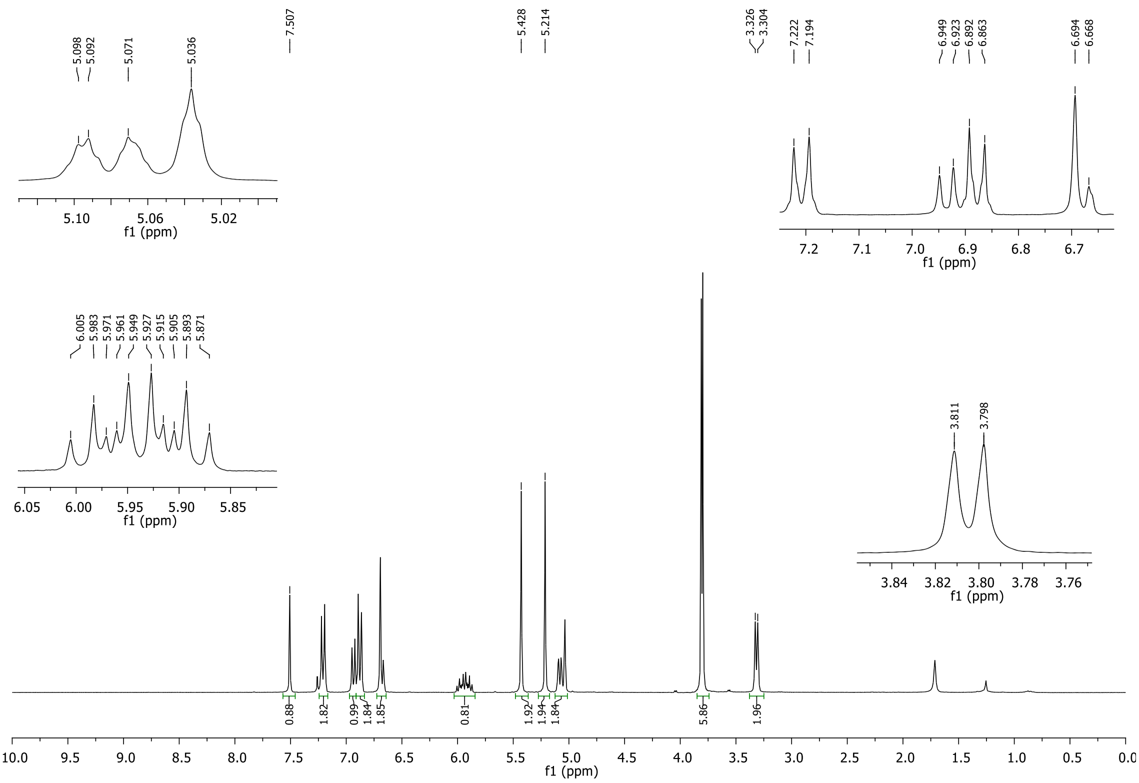

Fig. T. ^{1}H NMR spectrum (300 MHz, $CDCl_3$) of 4-((4-allyl-2-methoxyphenoxy)methyl)-1-(4-methoxybenzyl)-*1H*-1,2,3-triazole (**29**).

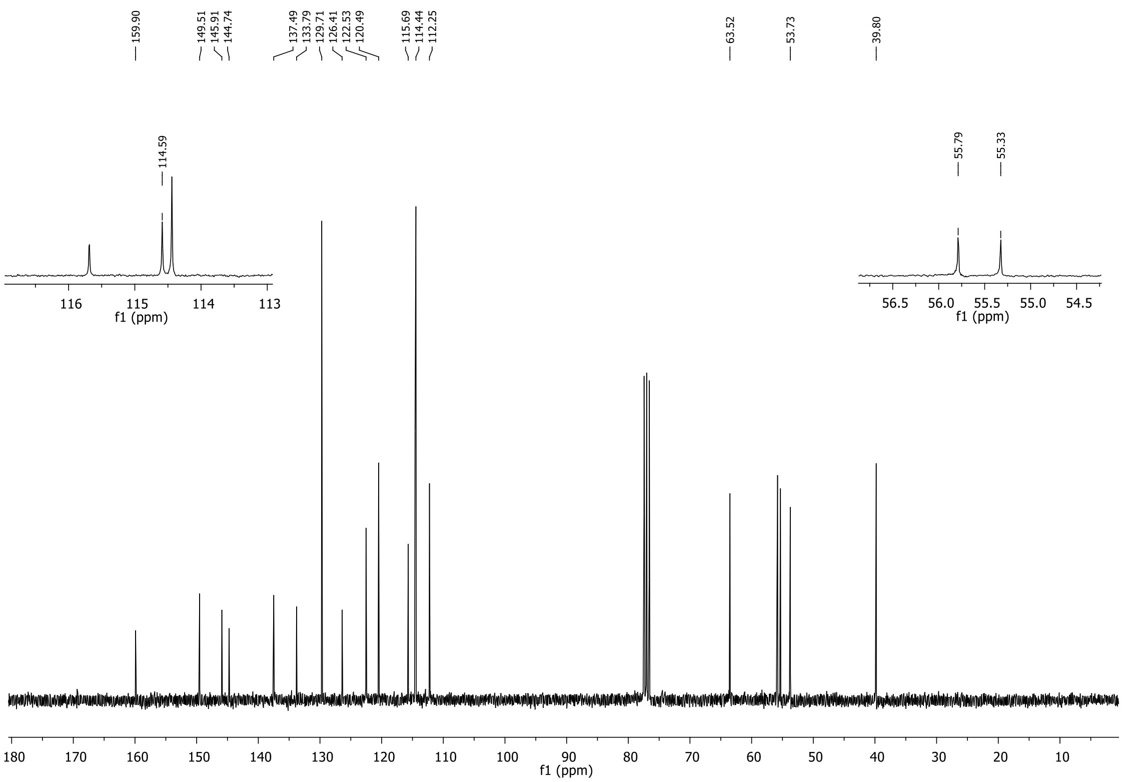

Fig. U. ^{13}C NMR spectrum (75 MHz, $CDCl_3$) of 4-((4-allyl-2-methoxyphenoxy)methyl)-1-(4-methoxybenzyl)-*1H*-1,2,3-triazole (**29**).

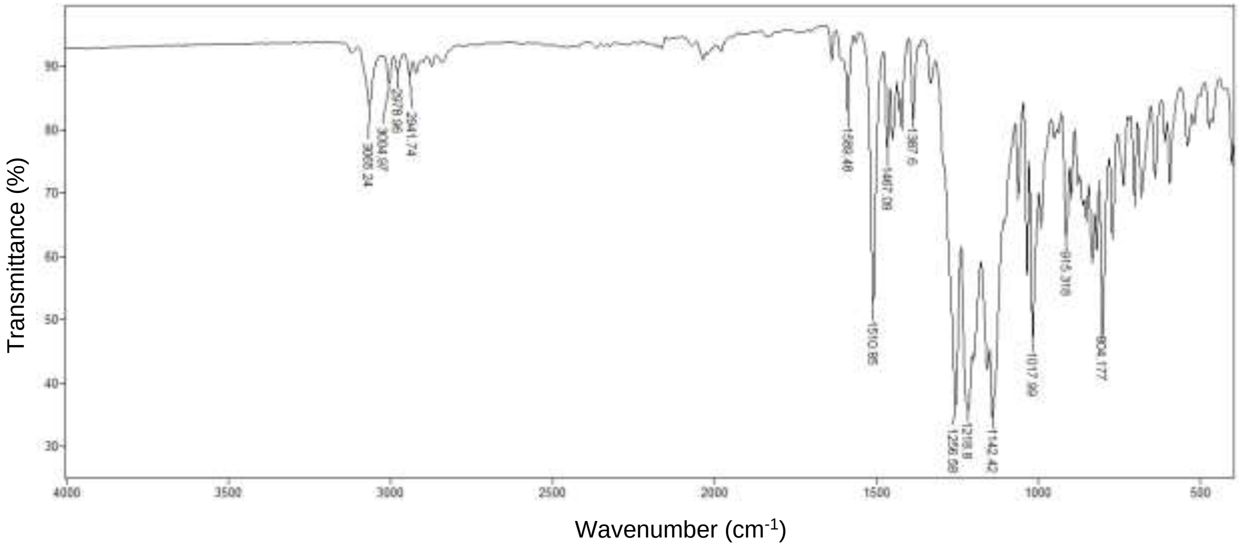

Fig. V. IR spectrum (ATR) of 4-((4-allyl-2-methoxyphenoxy)methyl)-1-(4-(trifluoromethoxy)benzyl)-*1H*-1,2,3-triazole (**30**).

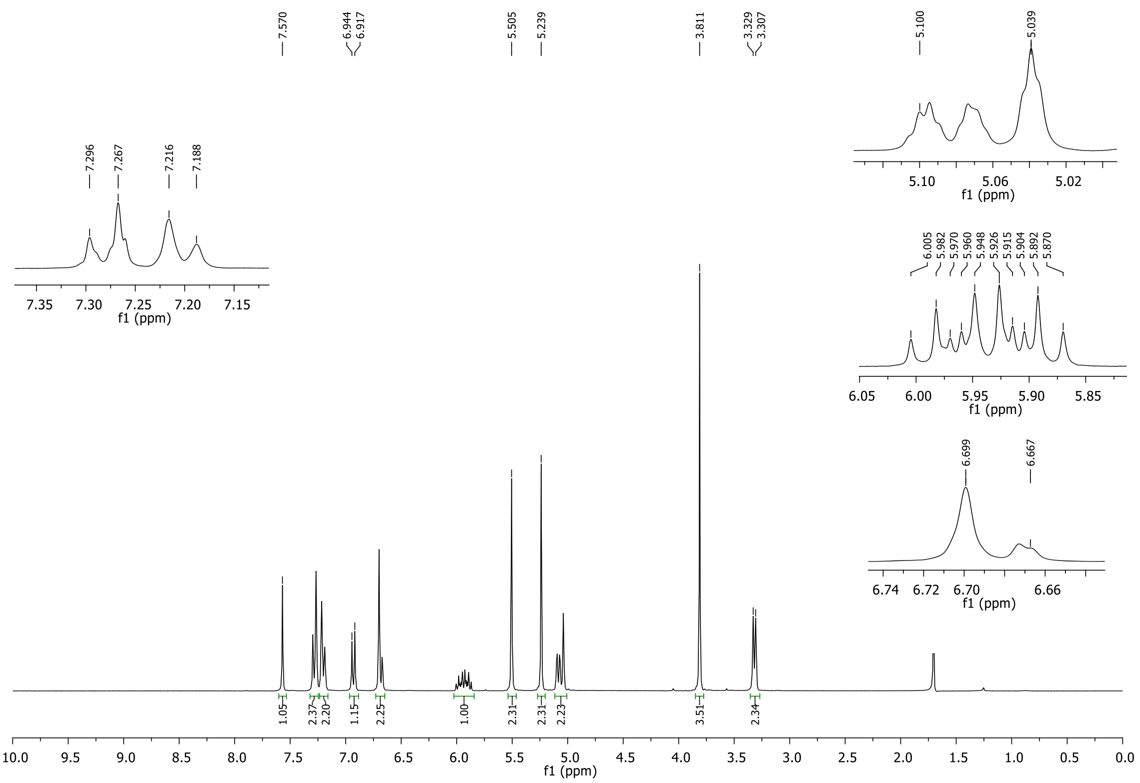

Fig. X. ^{1}H NMR spectrum (300 MHz, $CDCl_3$) 4-((4-allyl-2-methoxyphenoxy)methyl)-1-(4-(trifluoromethoxy)benzyl)-*1H*-1,2,3-triazole (**30**).

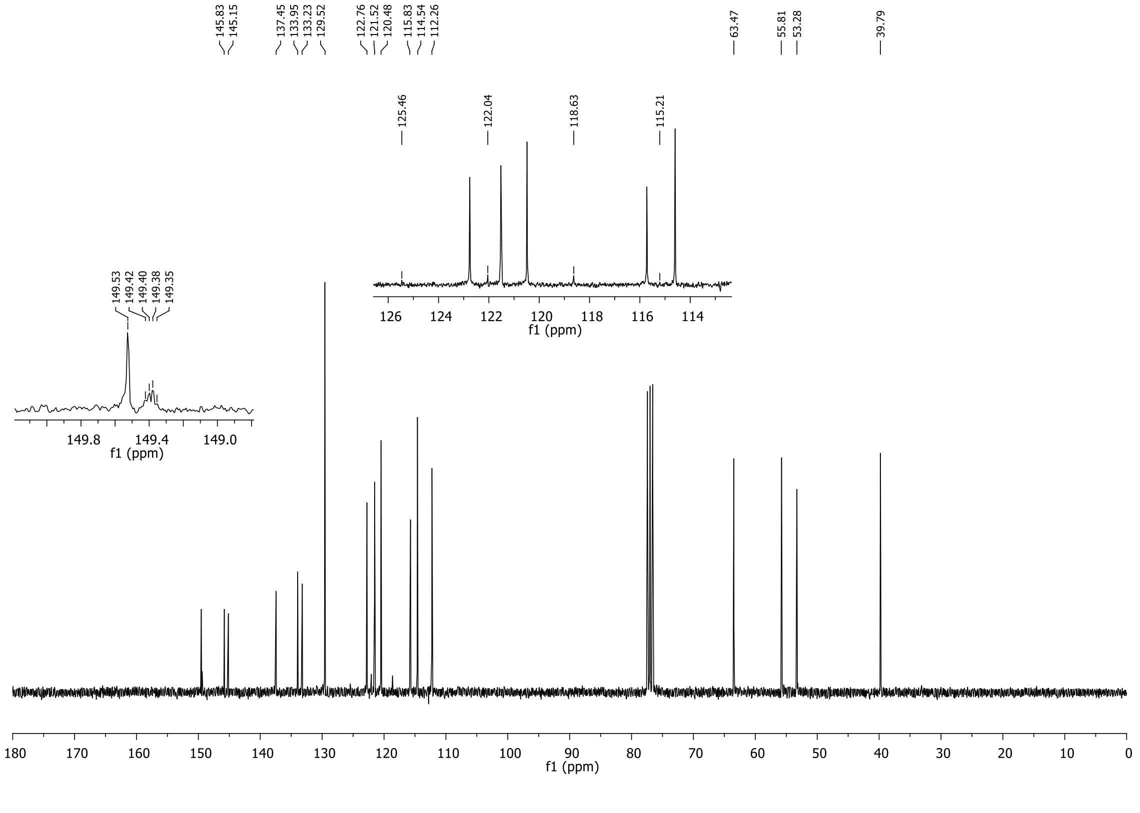

Fig. W. ^{13}C NMR spectrum (75 MHz, $CDCl_3$) of 4-((4-allyl-2-methoxyphenoxy)methyl)-1-(4-(trifluoromethoxy)benzyl)-*1H*-1,2,3-triazole (**30**).

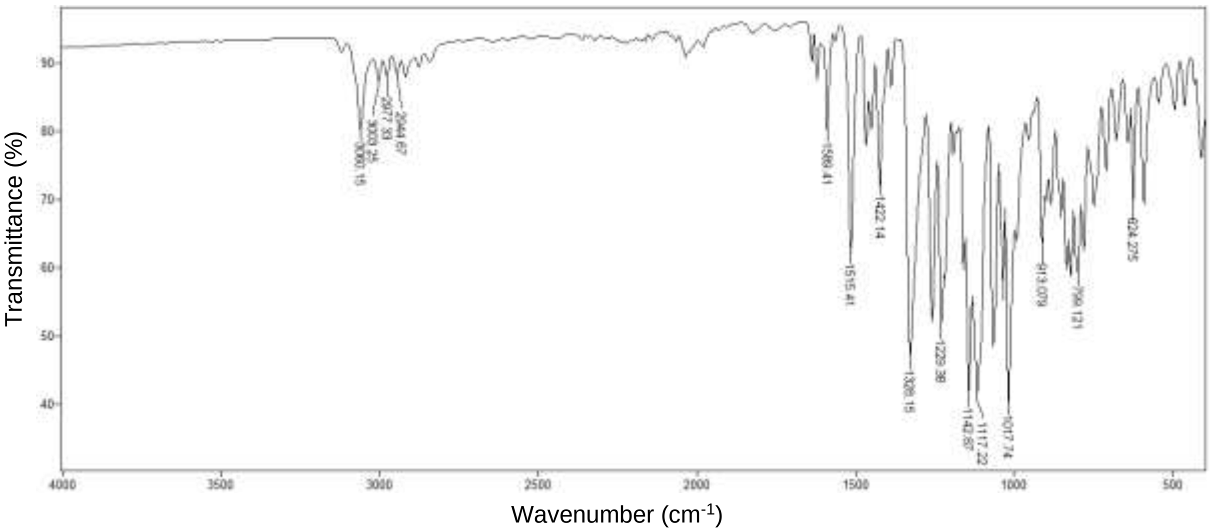

Fig. Y. IR spectrum (ATR) of 4-((4-allyl-2-methoxyphenoxy)methyl)-1-(4-(trifluoromethyl)benzyl)-*1H*-1,2,3-triazole (**31**).

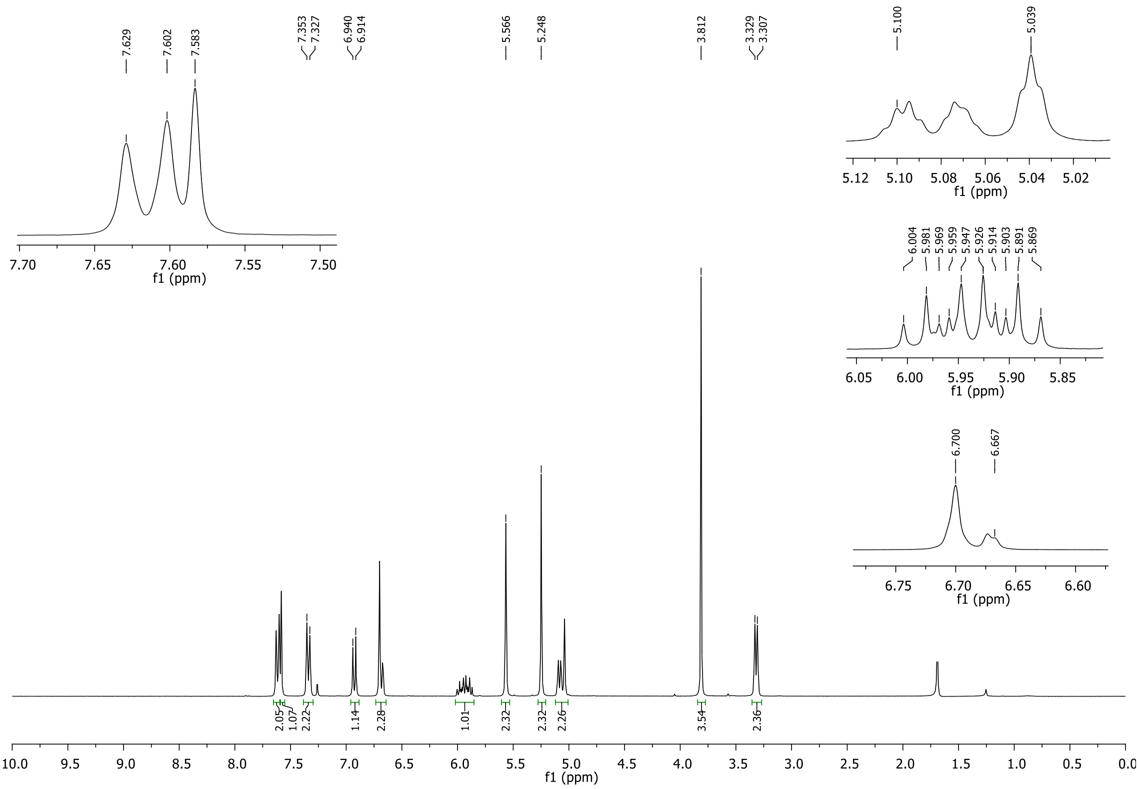

Fig. Z. ^{1}H NMR spectrum (300 MHz, $CDCl_3$) of 4-((4-allyl-2-methoxyphenoxy)methyl)-1-(4-(trifluoromethyl)benzyl)-*1H*-1,2,3-triazole (**31**).

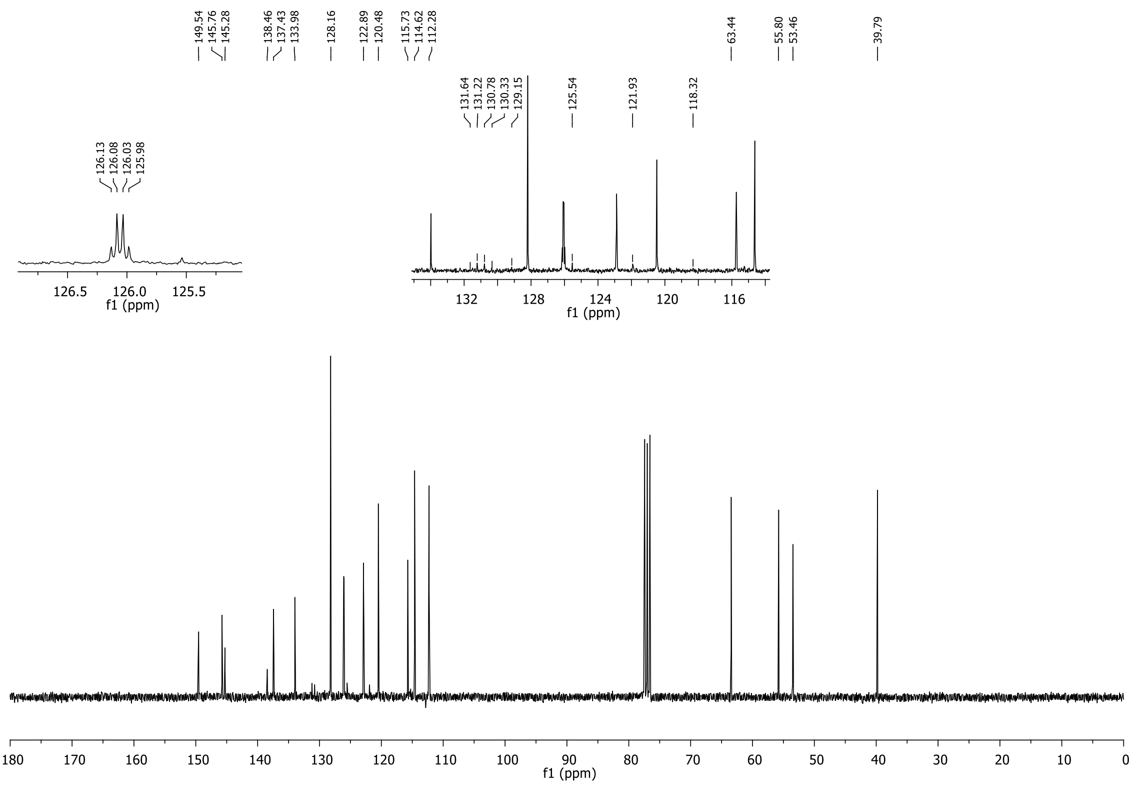

Fig. AA. ^{13}C NMR spectrum (75 MHz, $CDCl_3$) of 4-((4-allyl-2-methoxyphenoxy)methyl)-1-(4-(trifluoromethyl)benzyl)-*1H*-1,2,3-triazole (**31**).

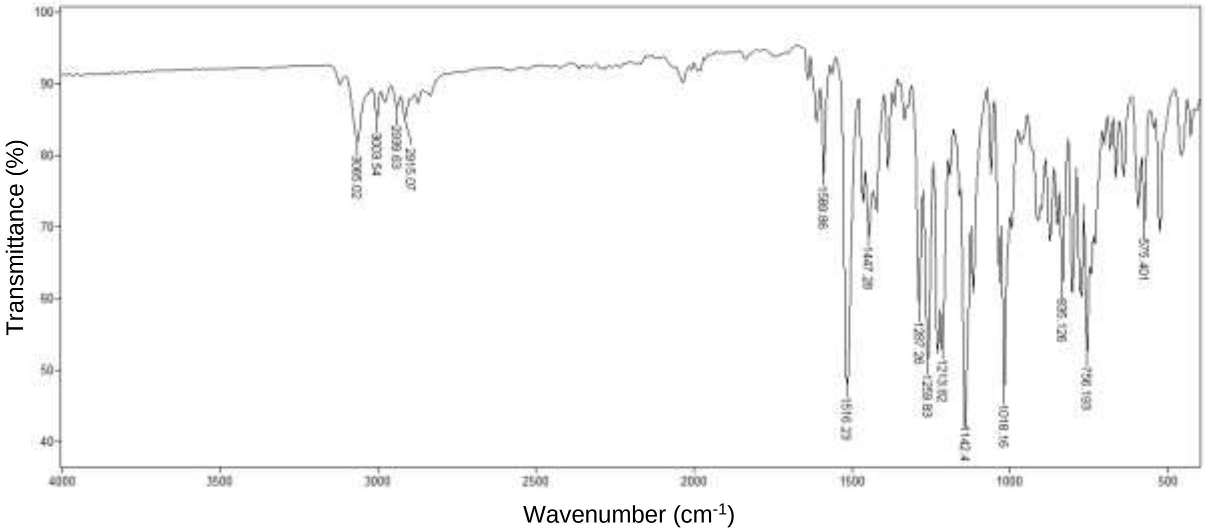

Fig. AB. IR spectrum (ATR) of 4-((4-allyl-2-methoxyphenoxy)methyl)-1-(3,4-difluorobenzyl)-*1H*-1,2,3-triazole (**32**).

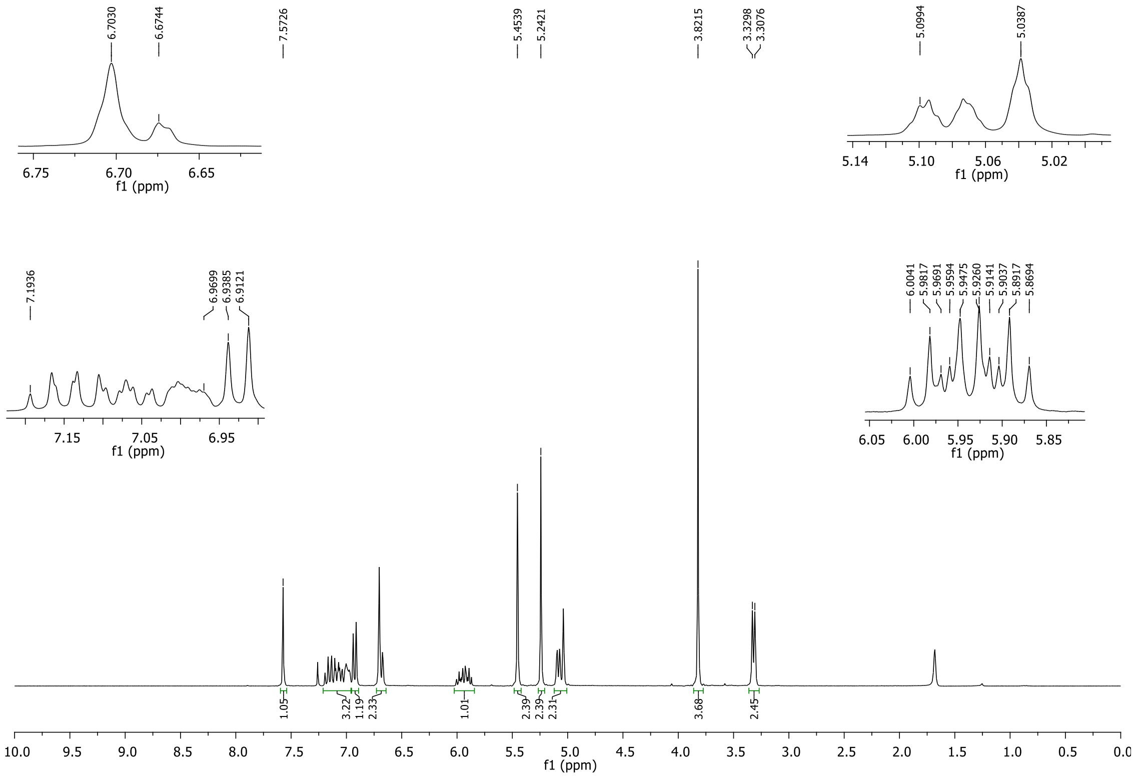

Fig. AC. ^{1}H NMR spectrum (300 MHz, $CDCl_3$) of 4-((4-allyl-2-methoxyphenoxy)methyl)-1-(3,4-difluorobenzyl)-*1H*-1,2,3-triazole (**32**).

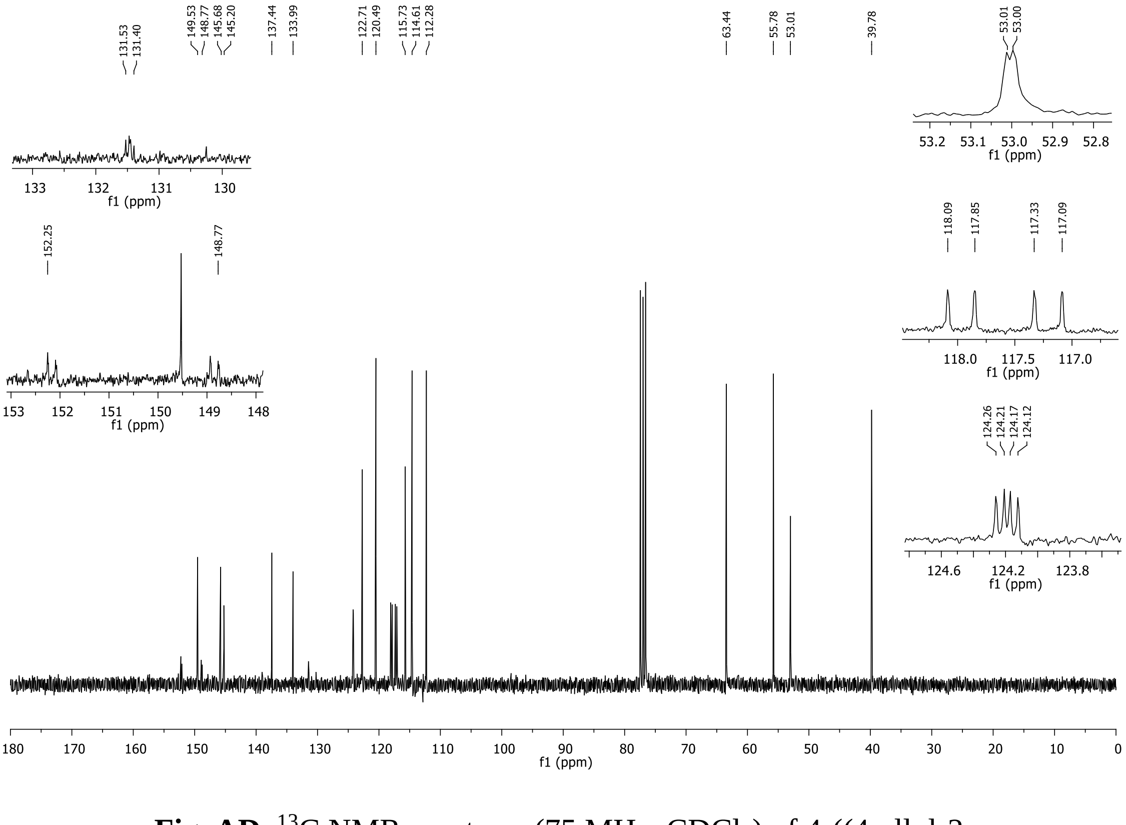

Fig. AD. ^{13}C NMR spectrum (75 MHz, $CDCl_3$) of 4-((4-allyl-2-methoxyphenoxy)methyl)-1-(3,4-difluorobenzyl)-*1H*-1,2,3-triazole (**32**).

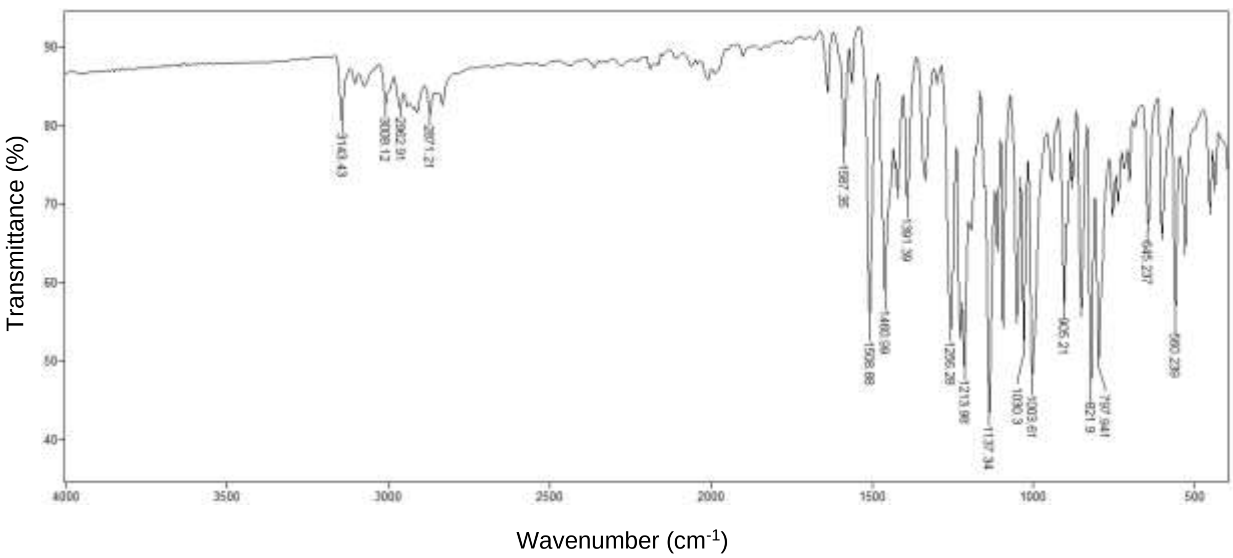

Fig. AE. IR spectrum (ATR) of 4-((4-allyl-2-methoxyphenoxy)methyl)-1-(2,5-dichlorobenzyl)-*1H*-1,2,3-triazole (**33**).

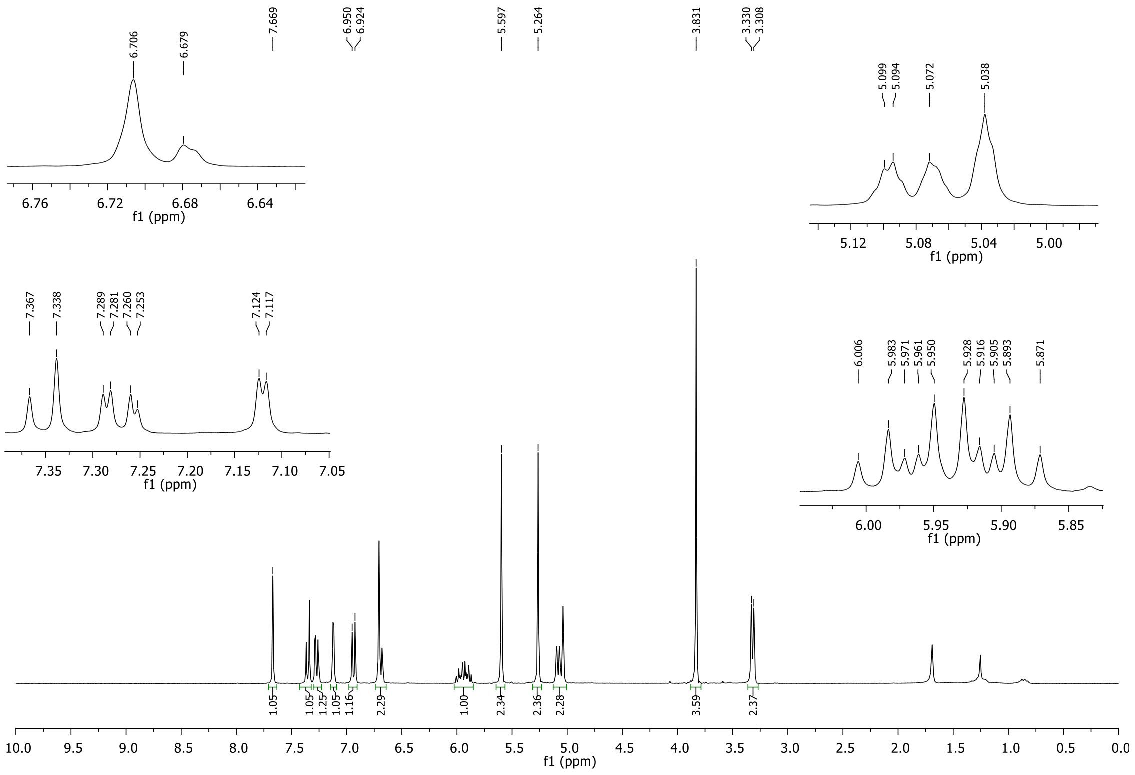

Fig. AF. ^{1}H NMR spectrum (300 MHz, $CDCl_3$) of 4-((4-allyl-2-methoxyphenoxy)methyl)-1-(2,5-dichlorobenzyl)-*1H*-1,2,3-triazole (**33**).

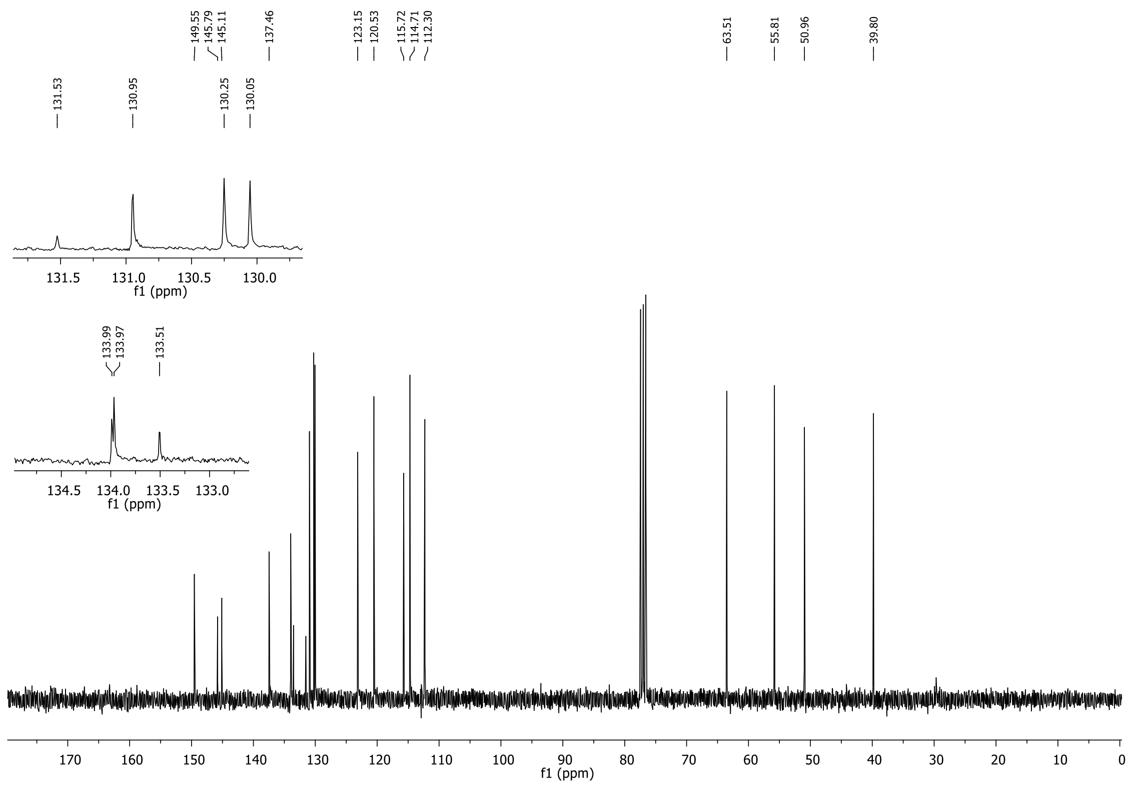

Fig. AG. ^{13}C NMR spectrum (75 MHz, $CDCl_3$) of 4-((4-allyl-2-methoxyphenoxy)methyl)-1-(2,5-dichlorobenzyl)-*1H*-1,2,3-triazole (**33**).

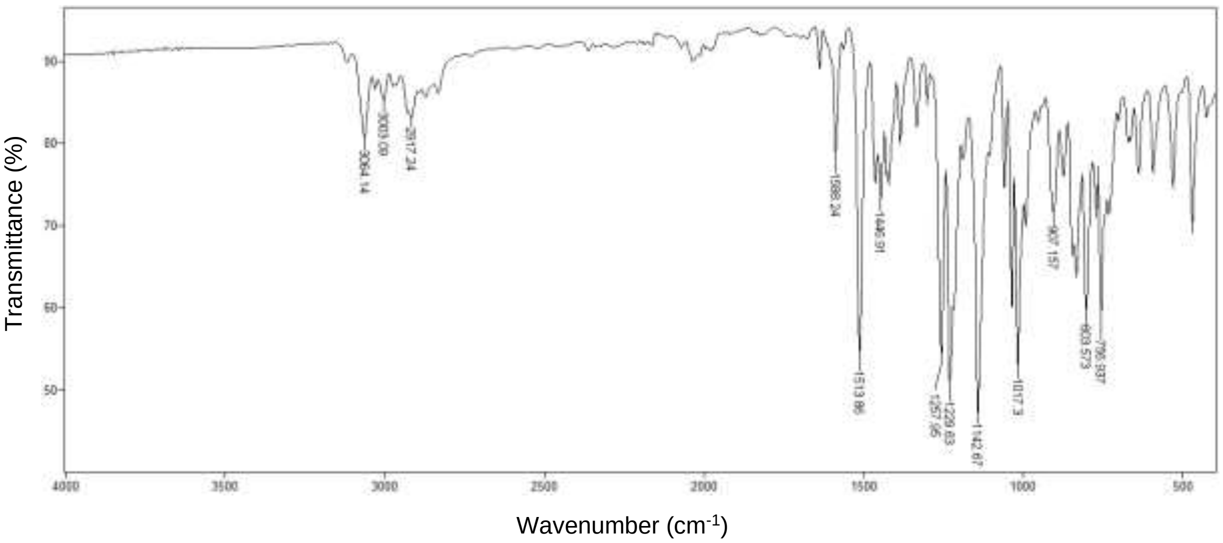

Fig. AH. IR spectrum (ATR) of 4-((4-allyl-2-methoxyphenoxy)methyl)-1-(4-methylbenzyl)-*1H*-1,2,3-triazole (**34**).

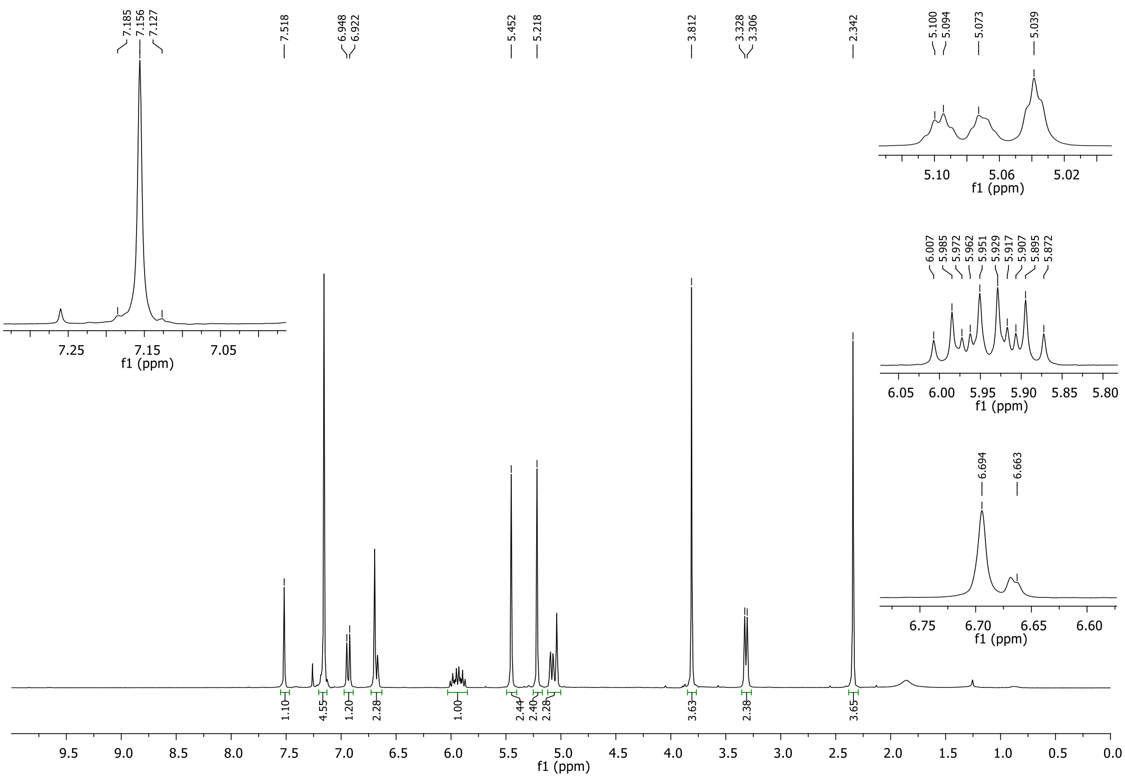

Fig. AI. ^{1}H NMR spectrum (300 MHz, $CDCl_3$) of 4-((4-allyl-2-methoxyphenoxy)methyl)-1-(4-methylbenzyl)-*1H*-1,2,3-triazole (**34**).

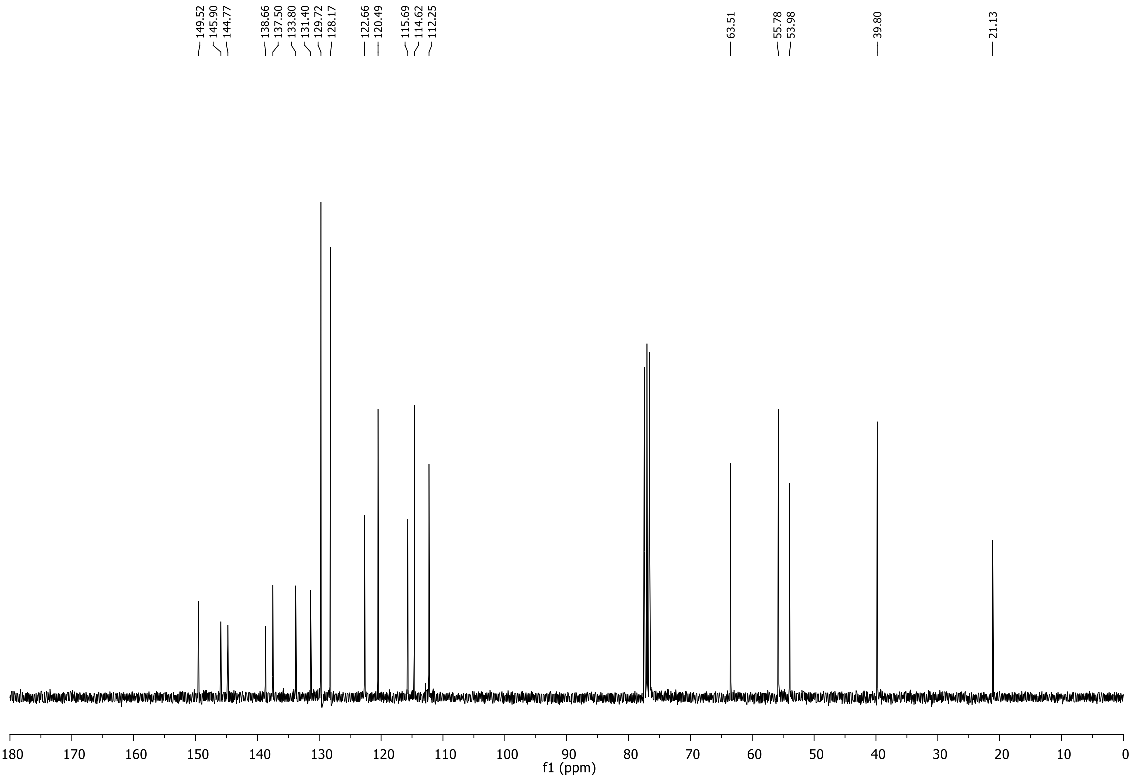

Fig. AJ. ^{13}C NMR spectrum (75 MHz, $CDCl_3$) of 4-((4-allyl-2-methoxyphenoxy)methyl)-1-(4-methylbenzyl)-*1H*-1,2,3-triazole (**34**).

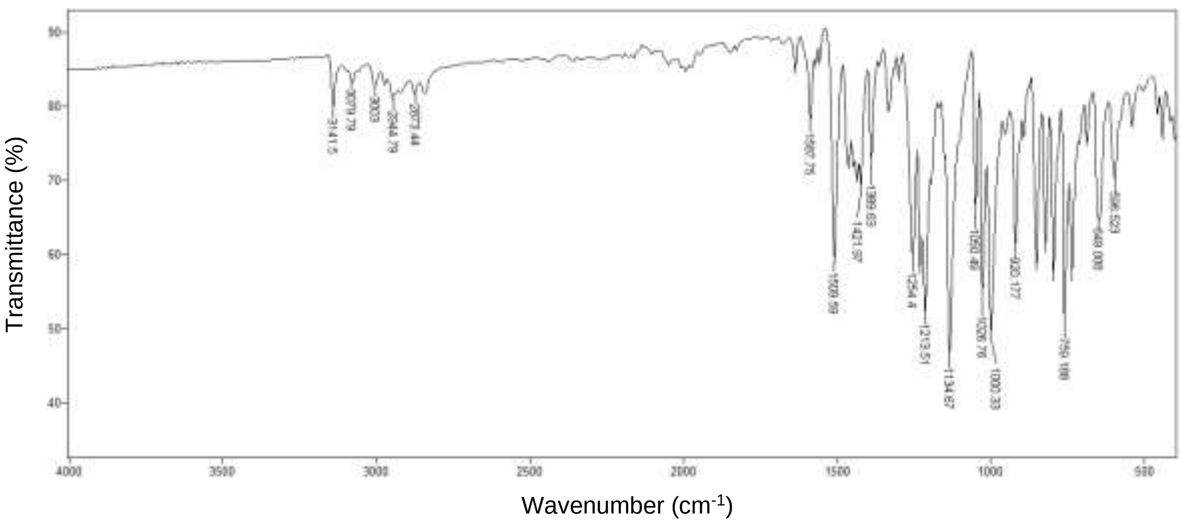

Fig. AK. IR spectrum (ATR) of 4-((4-allyl-2-methoxyphenoxy)methyl)-1-(2-bromobenzyl)-*1H*-1,2,3-triazole (**35**).

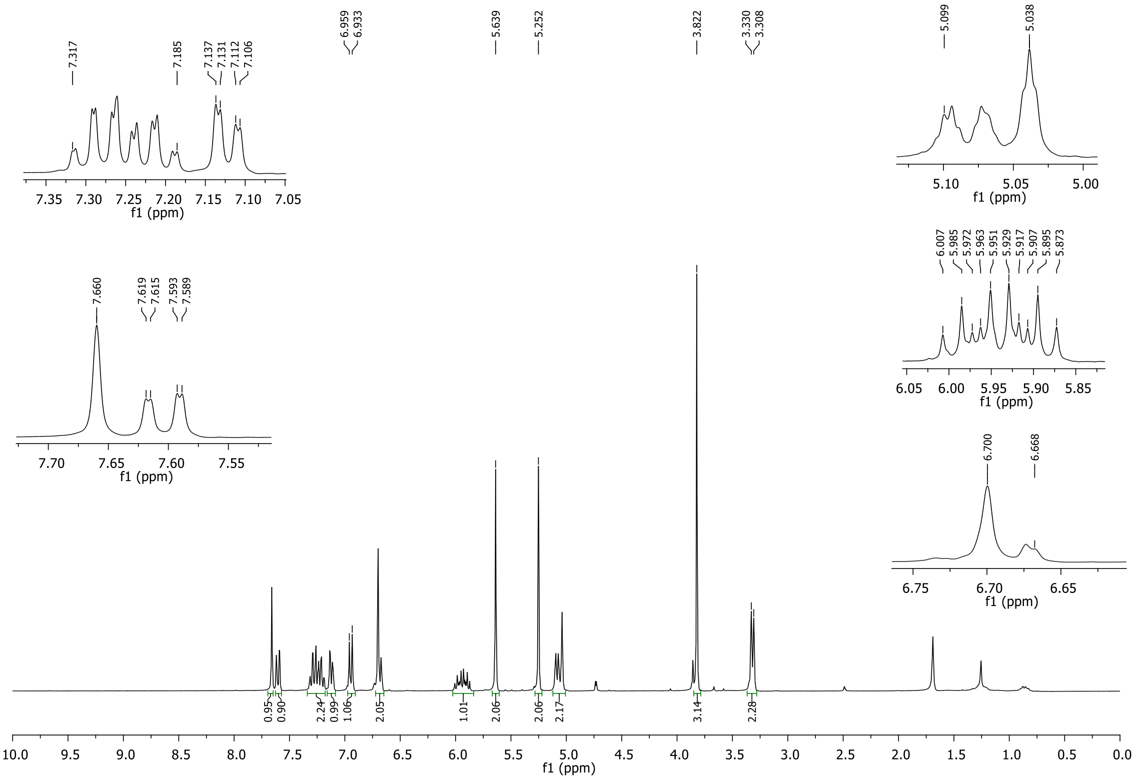

Fig. AL. ^{1}H NMR spectrum (300 MHz, $CDCl_3$) of 4-((4-allyl-2-methoxyphenoxy)methyl)-1-(2-bromobenzyl)-*1H*-1,2,3-triazole (**35**).

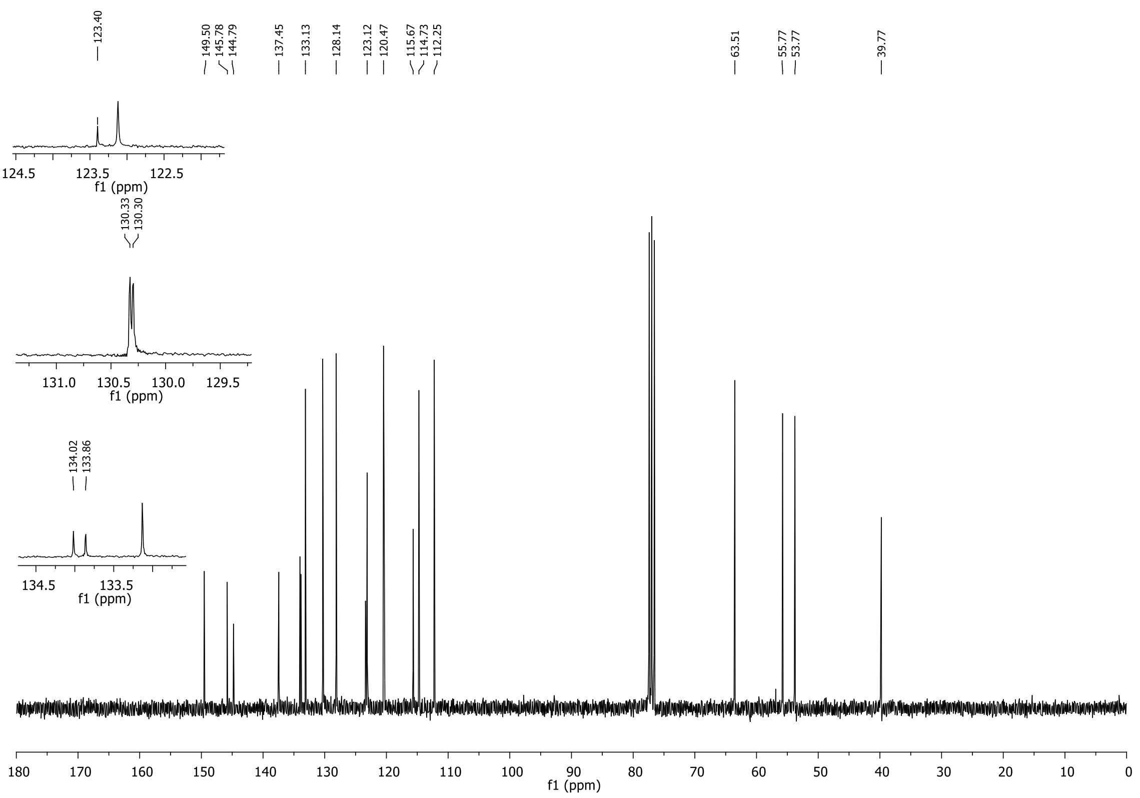

Fig. AM. ^{13}C NMR spectrum (75 MHz, $CDCl_3$) of 4-((4-allyl-2-methoxyphenoxy)methyl)-1-(2-bromobenzyl)-*1H*-1,2,3-triazole (**35**).

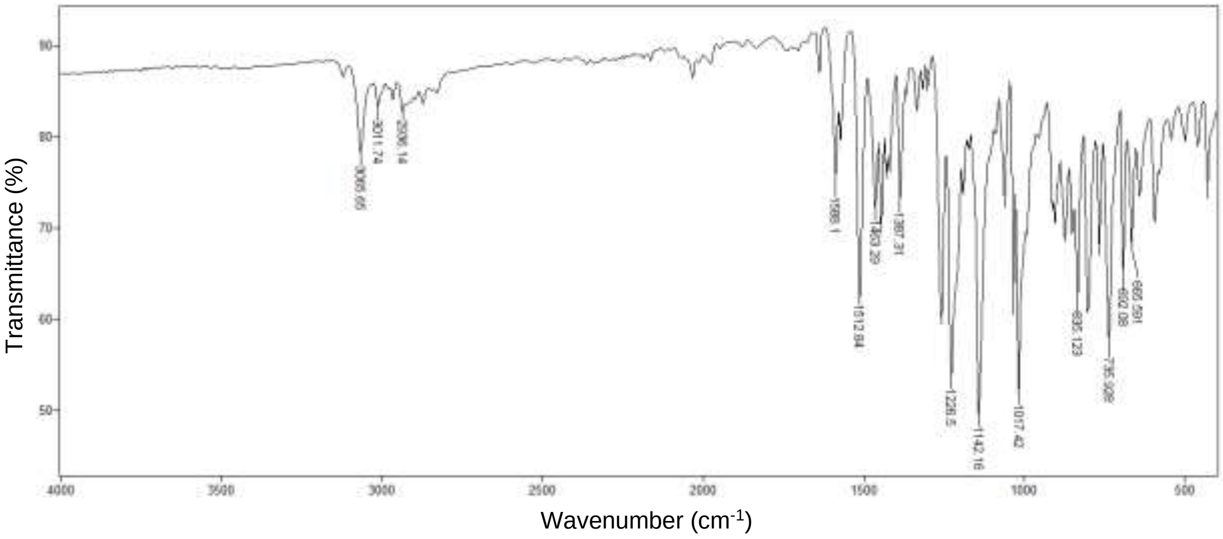

Fig. AN. IR spectrum (ATR) of 4-((4-allyl-2-methoxyphenoxy)methyl)-1-(3-bromobenzyl)-*1H*-1,2,3-triazole (**36**).

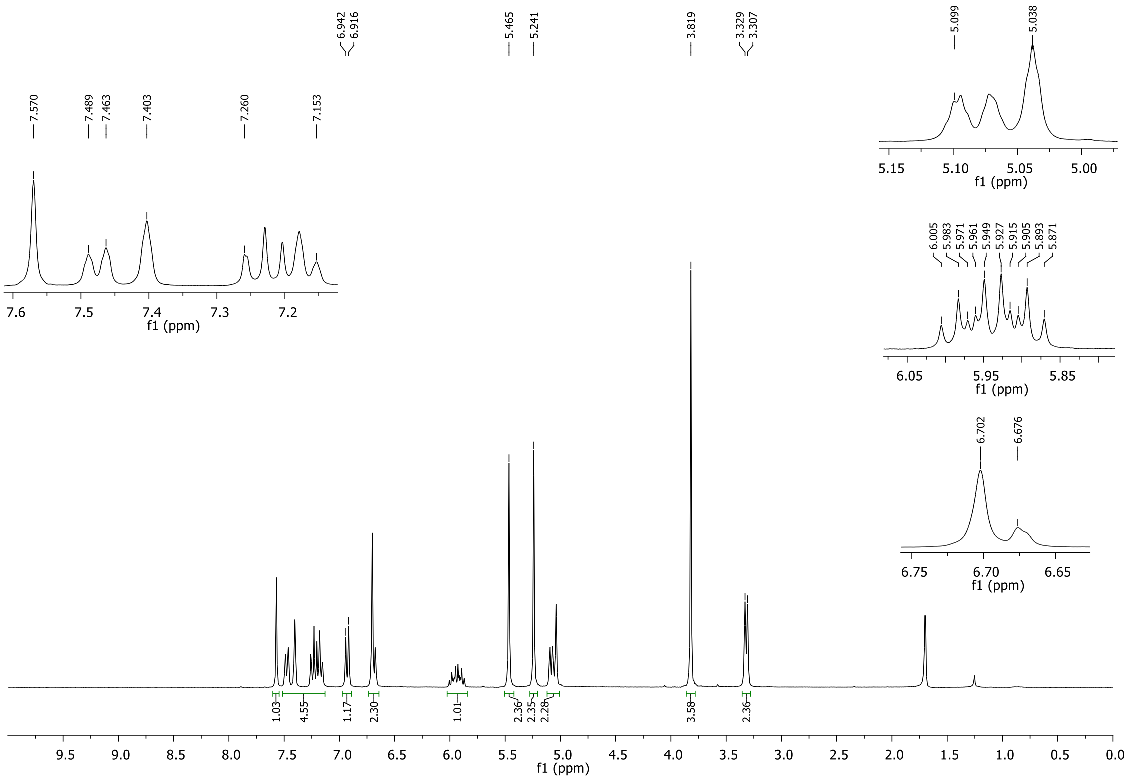

Fig. AO. ^{1}H NMR spectrum (300 MHz, $CDCl_3$) of 4-((4-allyl-2-methoxyphenoxy)methyl)-1-(3-bromobenzyl)-*1H*-1,2,3-triazole (**36**).

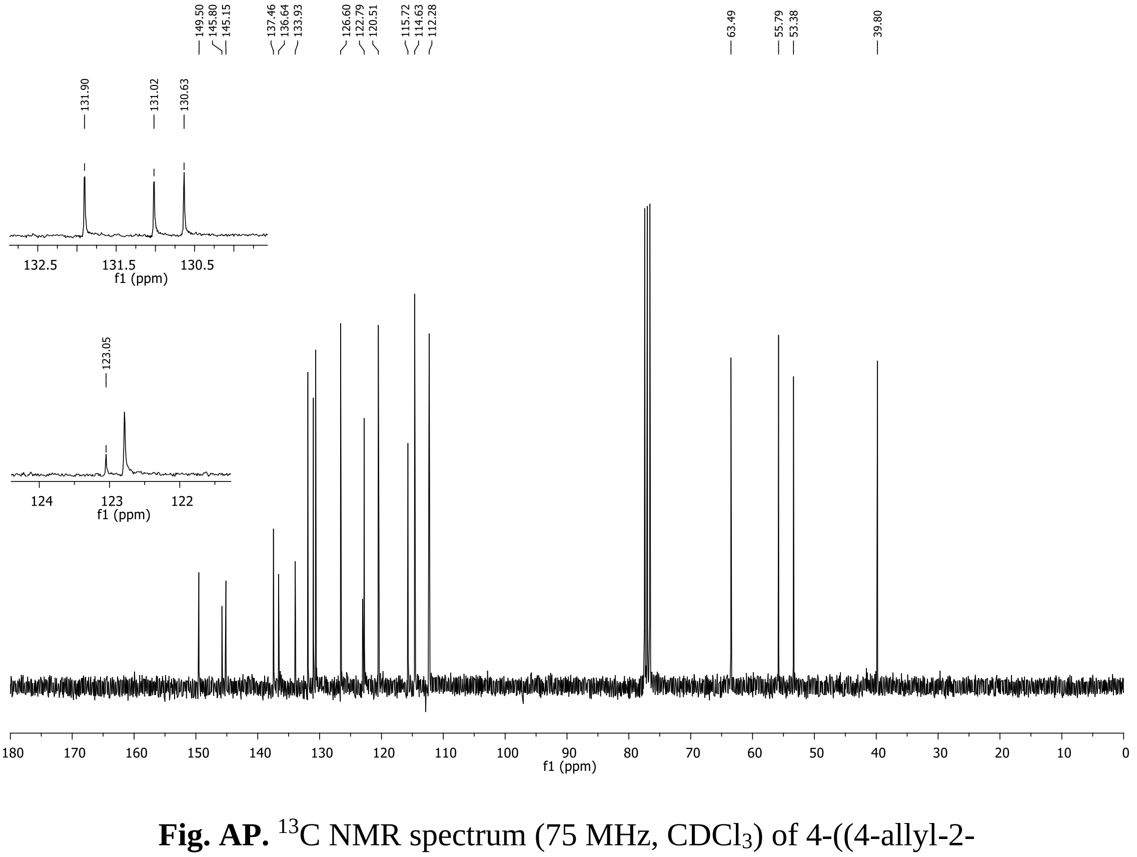

Fig. AP. ^{13}C NMR spectrum (75 MHz, $CDCl_3$) of 4-((4-allyl-2-methoxyphenoxy)methyl)-1-(3-bromobenzyl)-*1H*-1,2,3-triazole (**36**).

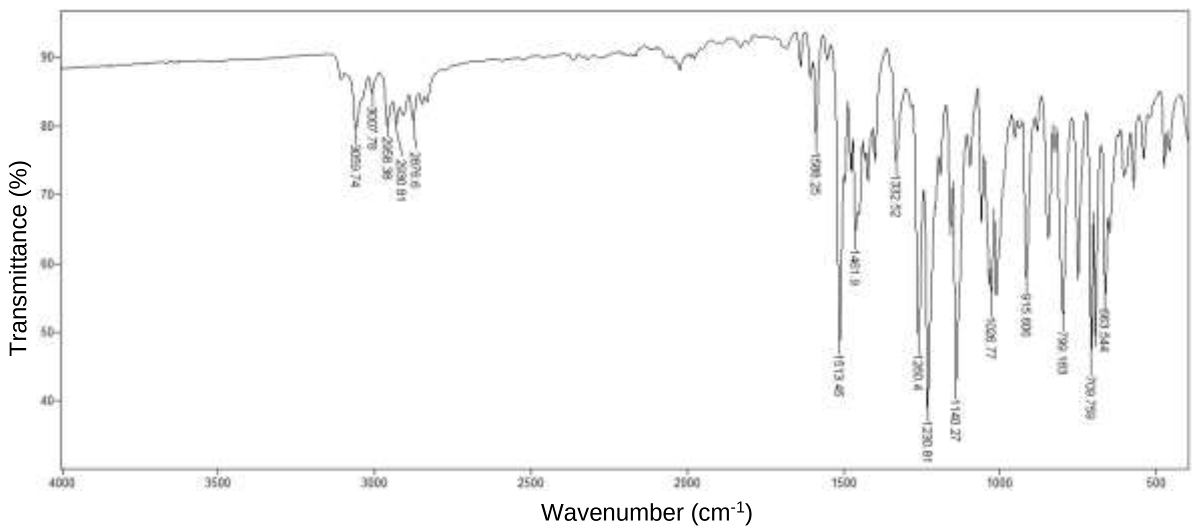

Fig. AQ. IR spectrum (ATR) of 4-(3-(4-allyl-2-methoxyphenoxy)propyl)-1-benzyl-*1H*-1,2,3-triazole (**37**).

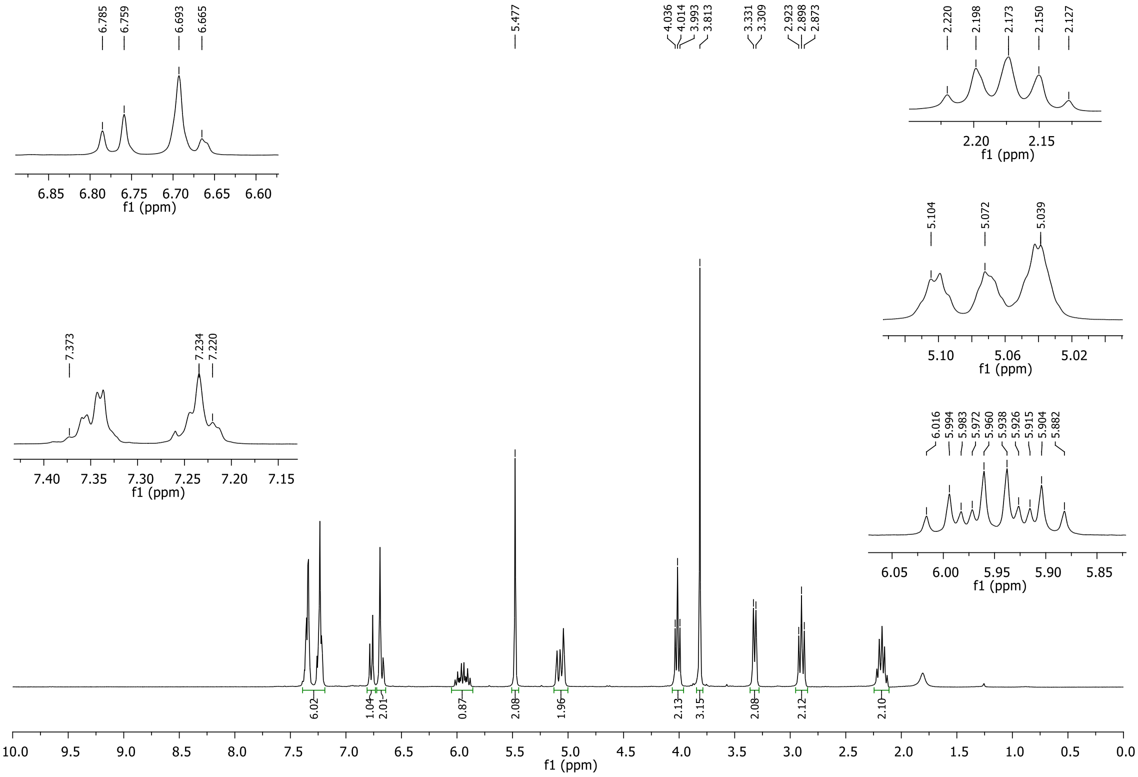

Fig. AR. ^{1}H NMR spectrum (300 MHz, $CDCl_3$) of 4-(3-(4-allyl-2-methoxyphenoxy)propyl)-1-benzyl-*1H*-1,2,3-triazole (**37**).

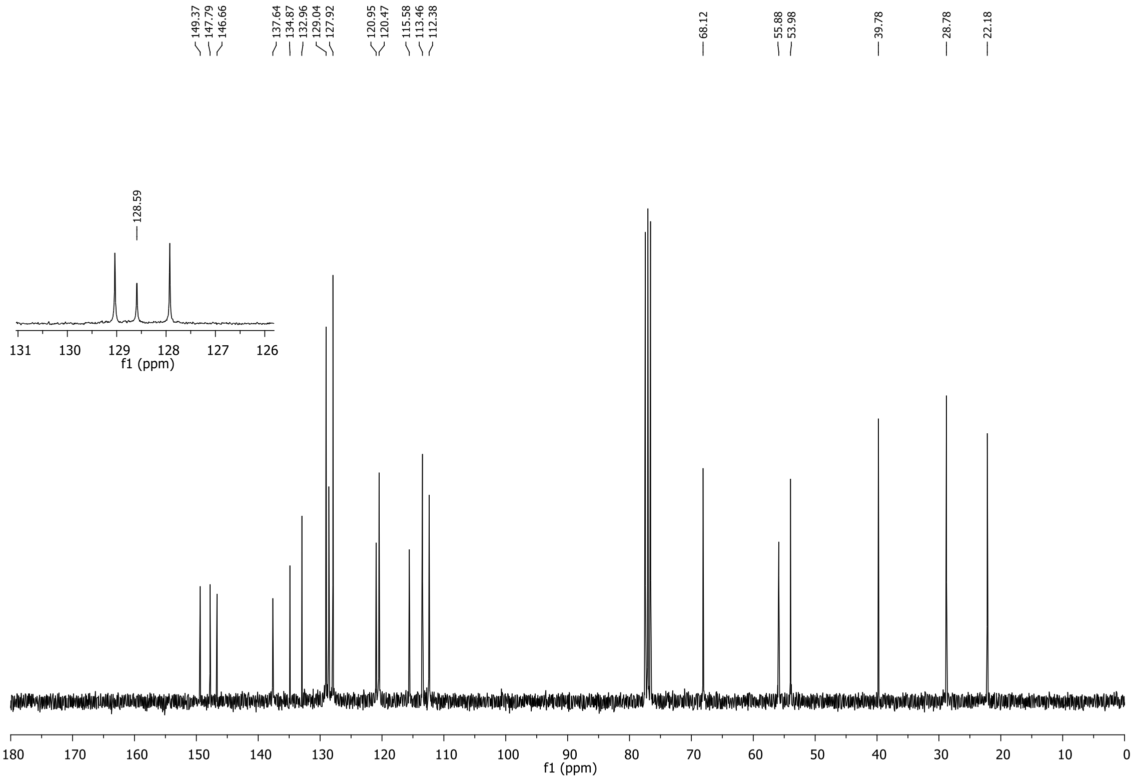

Fig. AS. ^{13}C NMR spectrum (75 MHz, $CDCl_3$) of 4-(3-(4-allyl-2-methoxyphenoxy)propyl)-1-benzyl-*1H*-1,2,3-triazole (**37**).

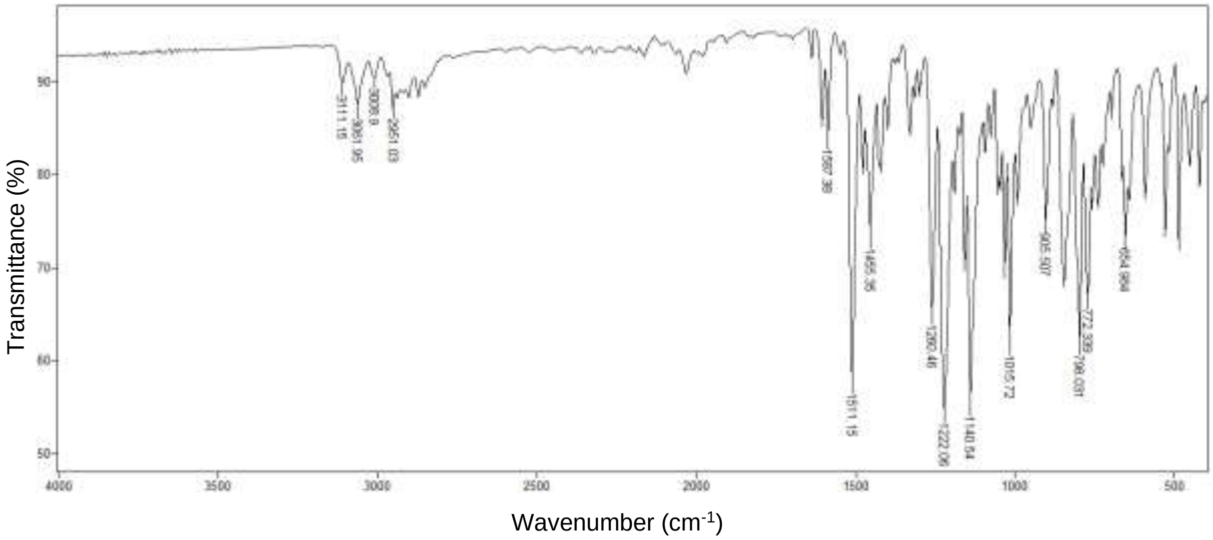

Fig. AT. IR spectrum (ATR) of 4-(3-(4-allyl-2-methoxyphenoxy)propyl)-1-(4-fluorobenzyl)-*1H*-1,2,3-triazole (**38**).

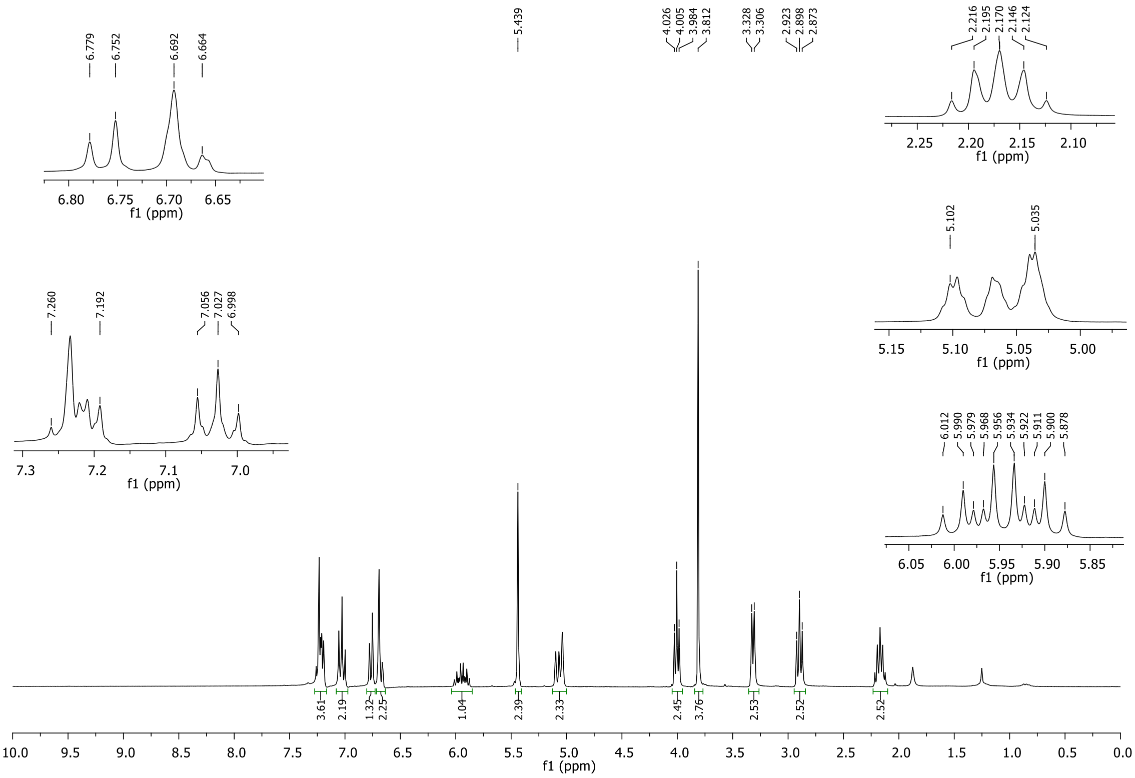

Fig. AU. ^{1}H NMR spectrum (300 MHz, $CDCl_3$) of 4-(3-(4-allyl-2-methoxyphenoxy)propyl)-1-(4-fluorobenzyl)-*1H*-1,2,3-triazole (**38**).

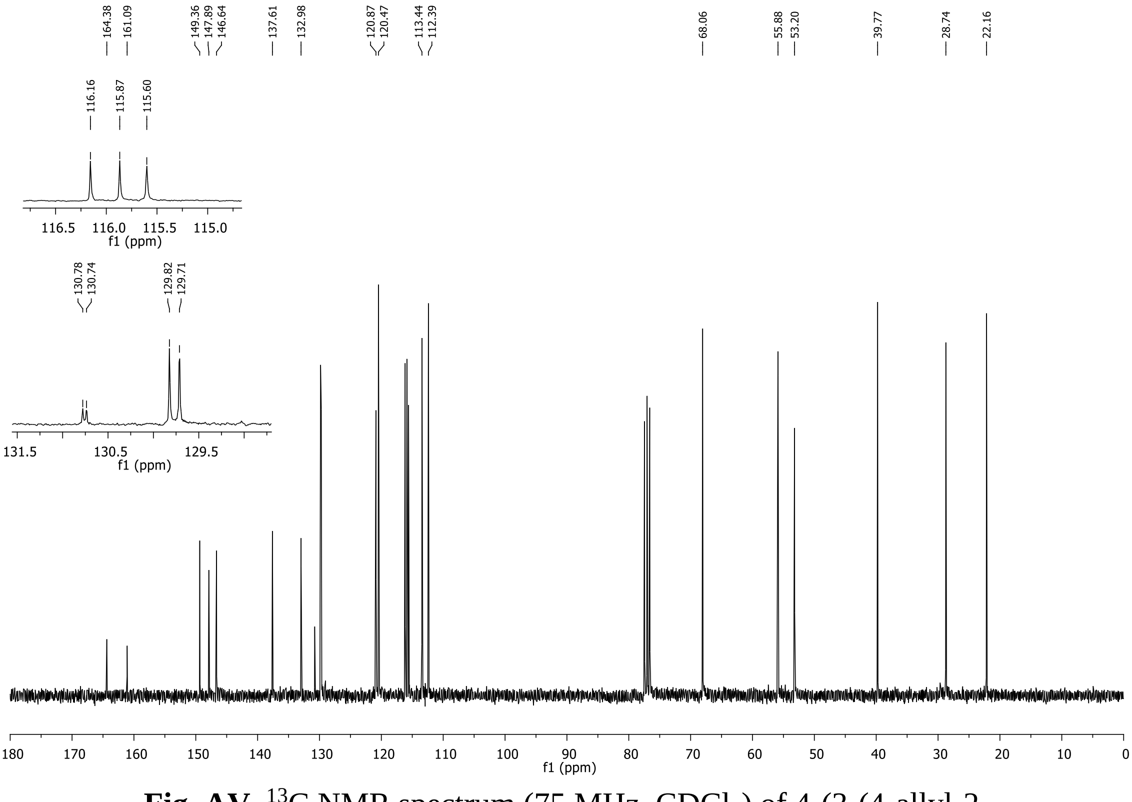

Fig. AV. ^{13}C NMR spectrum (75 MHz, $CDCl_3$) of 4-(3-(4-allyl-2-methoxyphenoxy)propyl)-1-(4-fluorobenzyl)-*1H*-1,2,3-triazole (**38**).

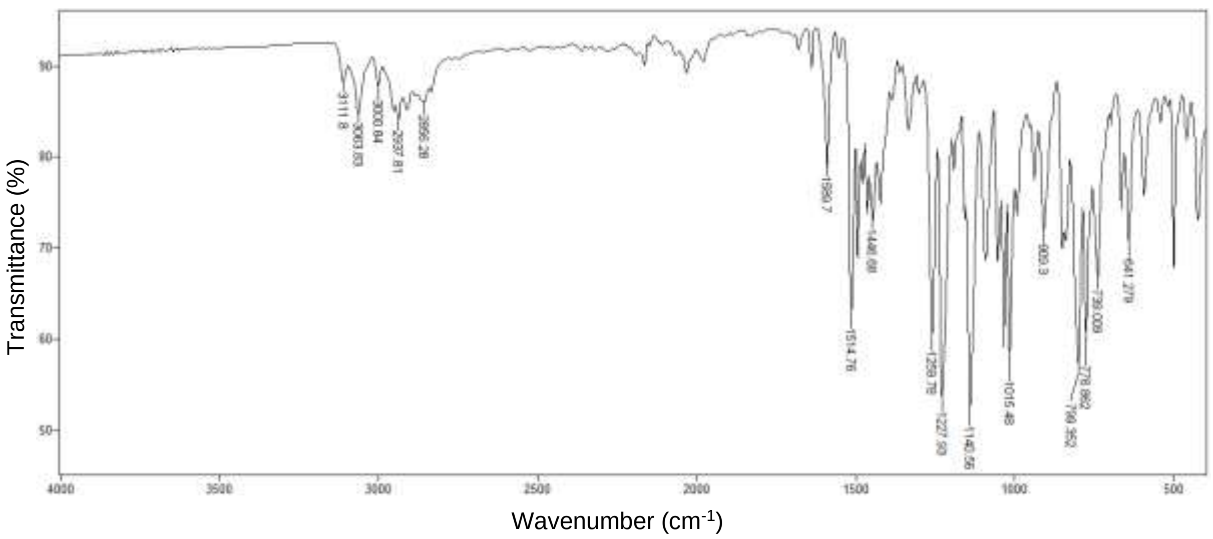

Fig. AX. IR spectrum (ATR) of 4-(3-(4-allyl-2-methoxyphenoxy)propyl)-1-(4-chlorobenzyl)-*1H*-1,2,3-triazole (**39**).

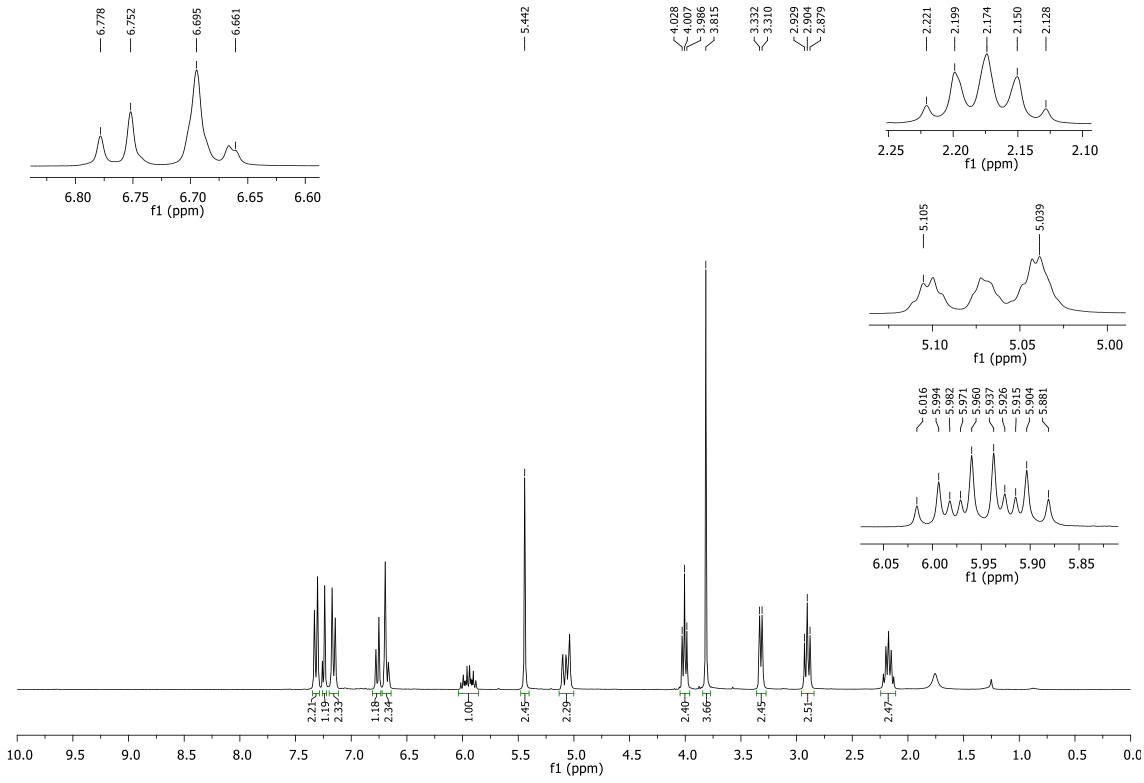

Fig. AW. ^{1}H NMR spectrum (300 MHz, $CDCl_3$) of 4-(3-(4-allyl-2-methoxyphenoxy)propyl)-1-(4-chlorobenzyl)-*1H*-1,2,3-triazole (**39**).

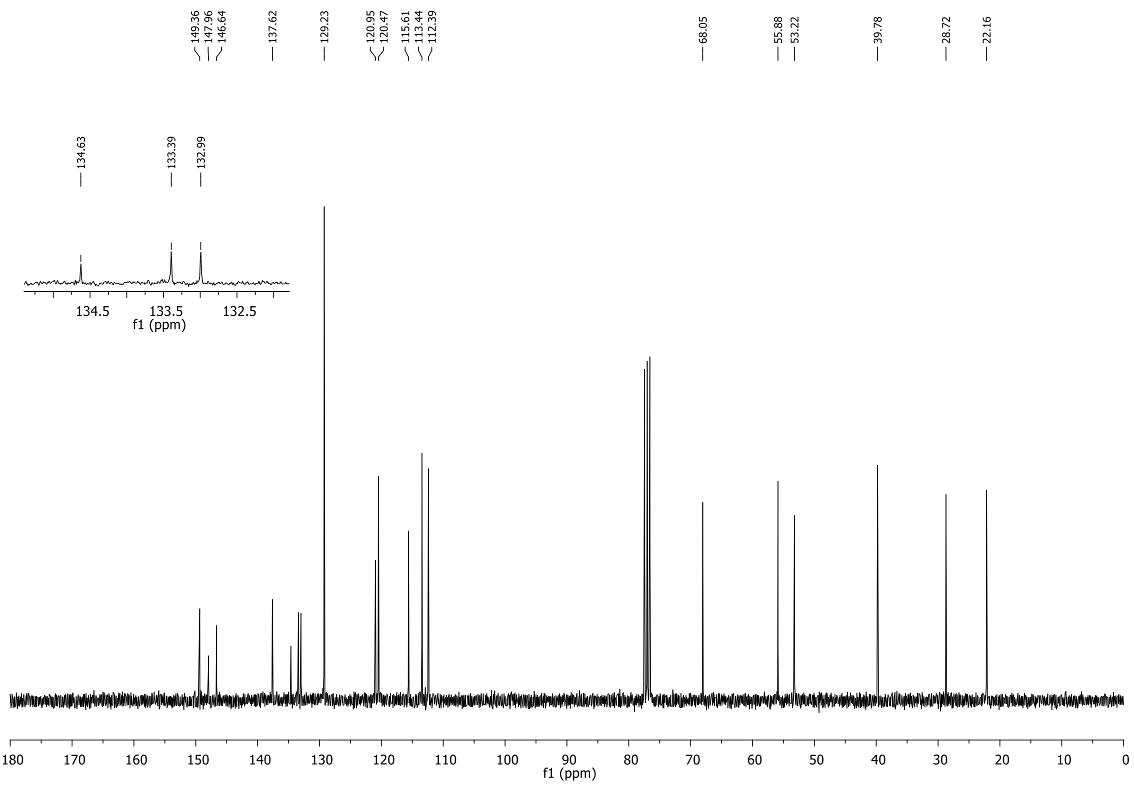

Fig. AY. ^{13}C NMR spectrum (75 MHz, $CDCl_3$) of 4-(3-(4-allyl-2-methoxyphenoxy)propyl)-1-(4-chlorobenzyl)-*1H*-1,2,3-triazole (**39**).

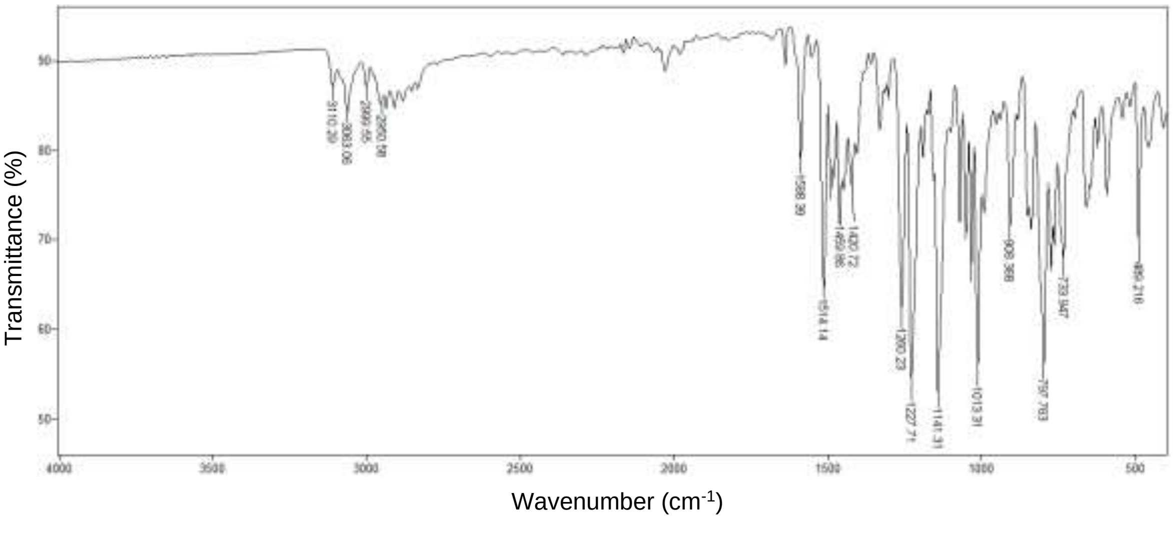

Fig. AZ. IR spectrum (ATR) of 4-(3-(4-allyl-2-methoxyphenoxy)propyl)-1-(4-bromobenzyl)-*1H*-1,2,3-triazole (**40**).

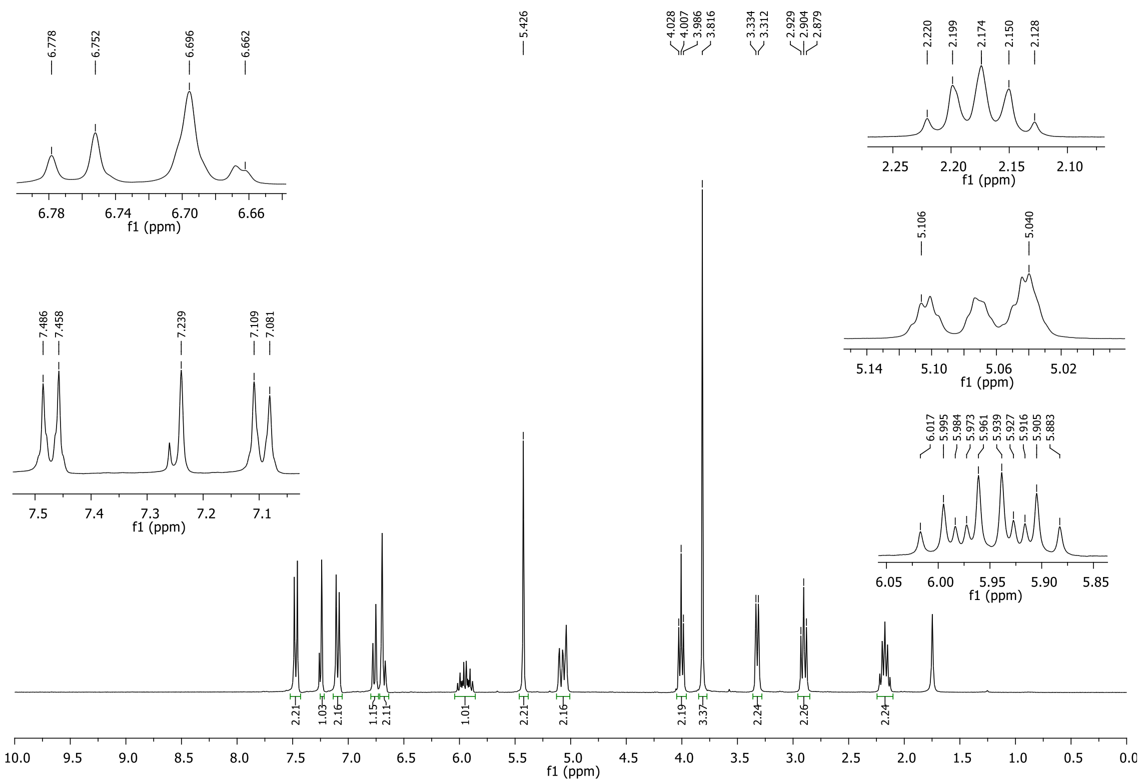

Fig. BA. ^{1}H NMR spectrum (300 MHz, $CDCl_3$) of 4-(3-(4-allyl-2-methoxyphenoxy)propyl)-1-(4-bromobenzyl)-*1H*-1,2,3-triazole (**40**).

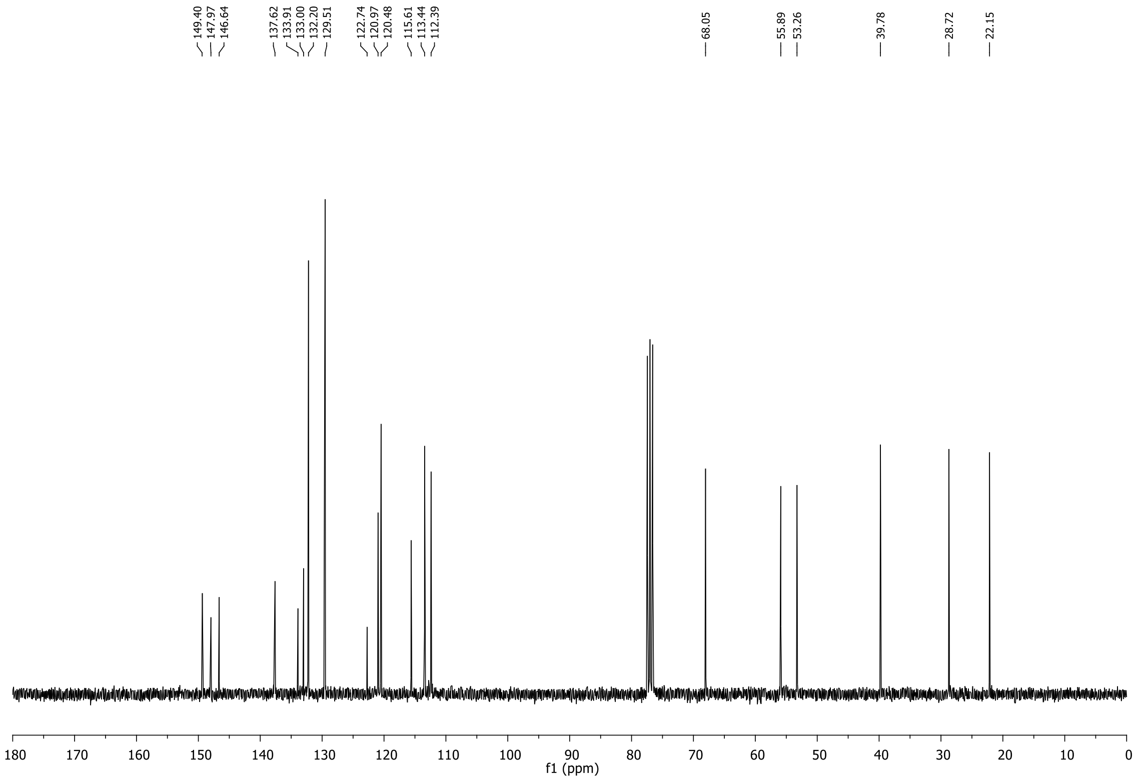

Fig. BB. ^{13}C NMR spectrum (75 MHz, $CDCl_3$) of 4-(3-(4-allyl-2-methoxyphenoxy)propyl)-1-(4-bromobenzyl)-*1H*-1,2,3-triazole (**40**).

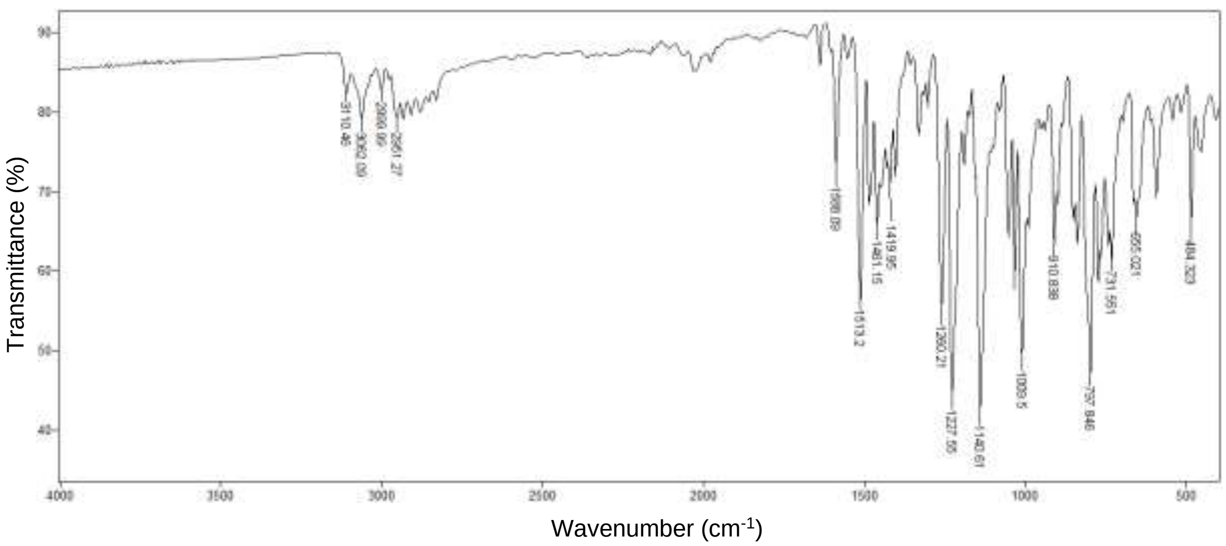

Fig. BC. IR spectrum (ATR) of 4-(3-(4-allyl-2-methoxyphenoxy)propyl)-1-(4-iodobenzyl)-*1H*-1,2,3-triazole (**41**).

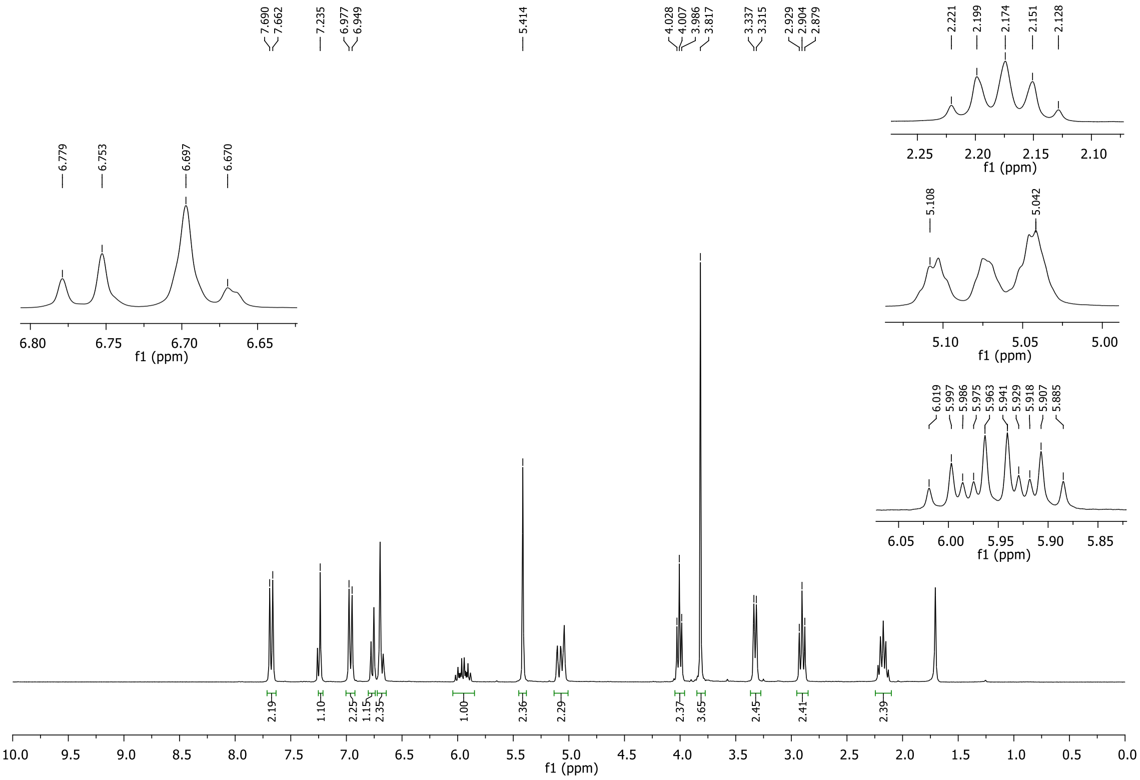

Fig. BD. ^{1}H NMR spectrum (300 MHz, $CDCl_3$) of 4-(3-(4-allyl-2-methoxyphenoxy)propyl)-1-(4-iodobenzyl)-*1H*-1,2,3-triazole (**41**).

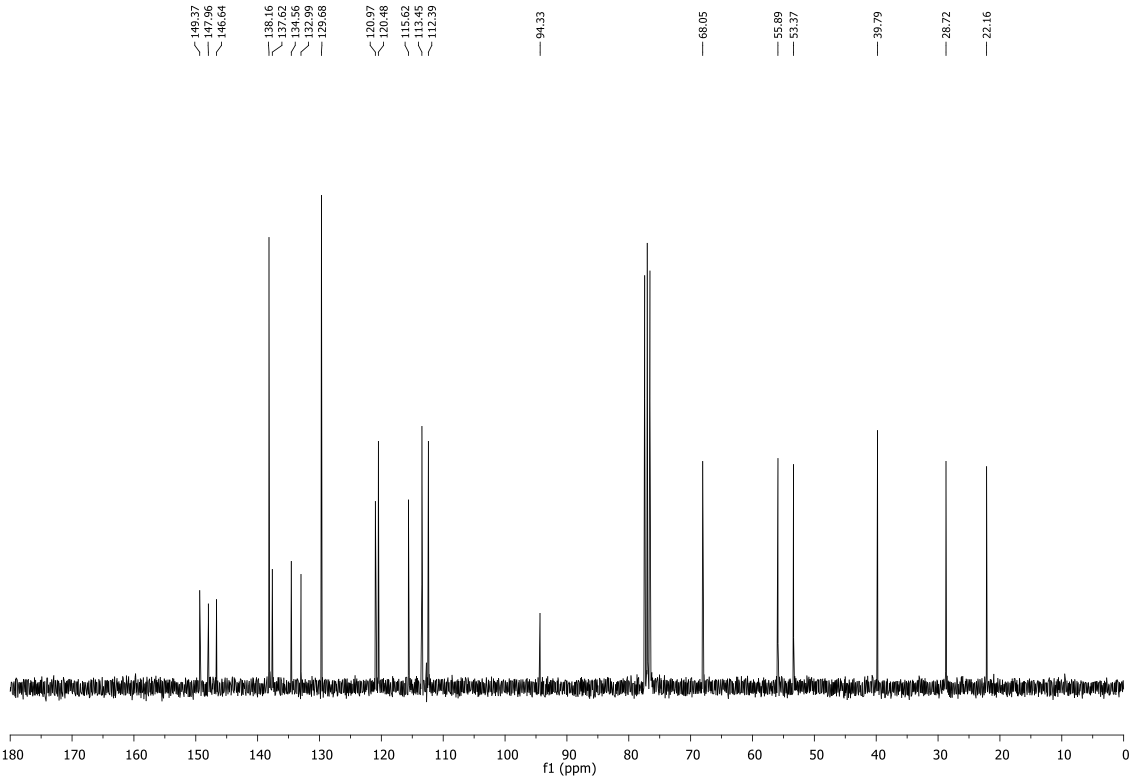

Fig. BE. ^{13}C NMR spectrum (75 MHz, $CDCl_3$) of 4-(3-(4-allyl-2-methoxyphenoxy)propyl)-1-(4-iodobenzyl)-*1H*-1,2,3-triazole (**41**).

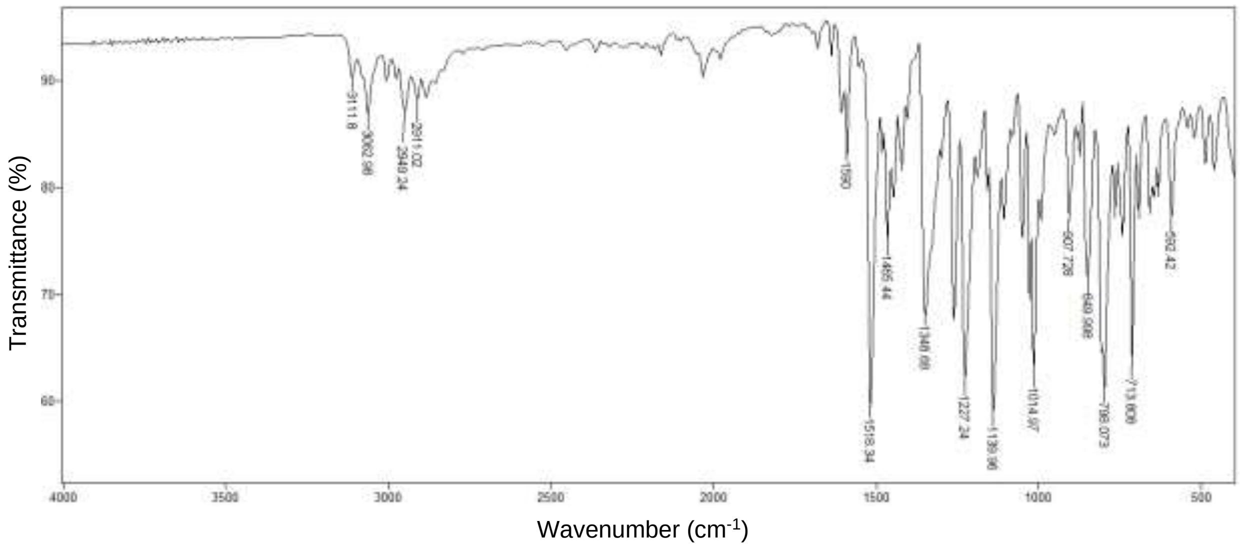

Fig. BF. IR spectrum (ATR) of 4-(3-(4-allyl-2-methoxyphenoxy)propyl)-1-(4-nitrobenzyl)-*1H*-1,2,3-triazole (**42**).

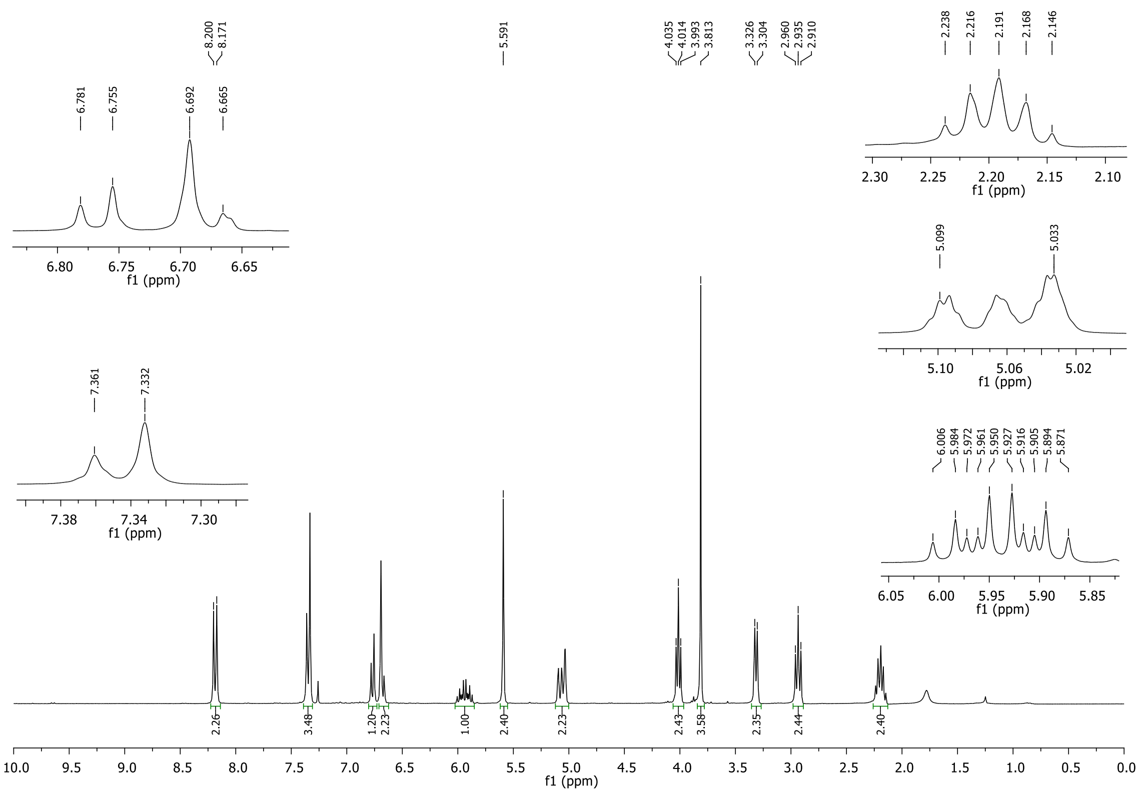

Fig. BG. ^{1}H NMR spectrum (300 MHz, $CDCl_3$) of 4-(3-(4-allyl-2-methoxyphenoxy)propyl)-1-(4-nitrobenzyl)-*1H*-1,2,3-triazole (**42**).

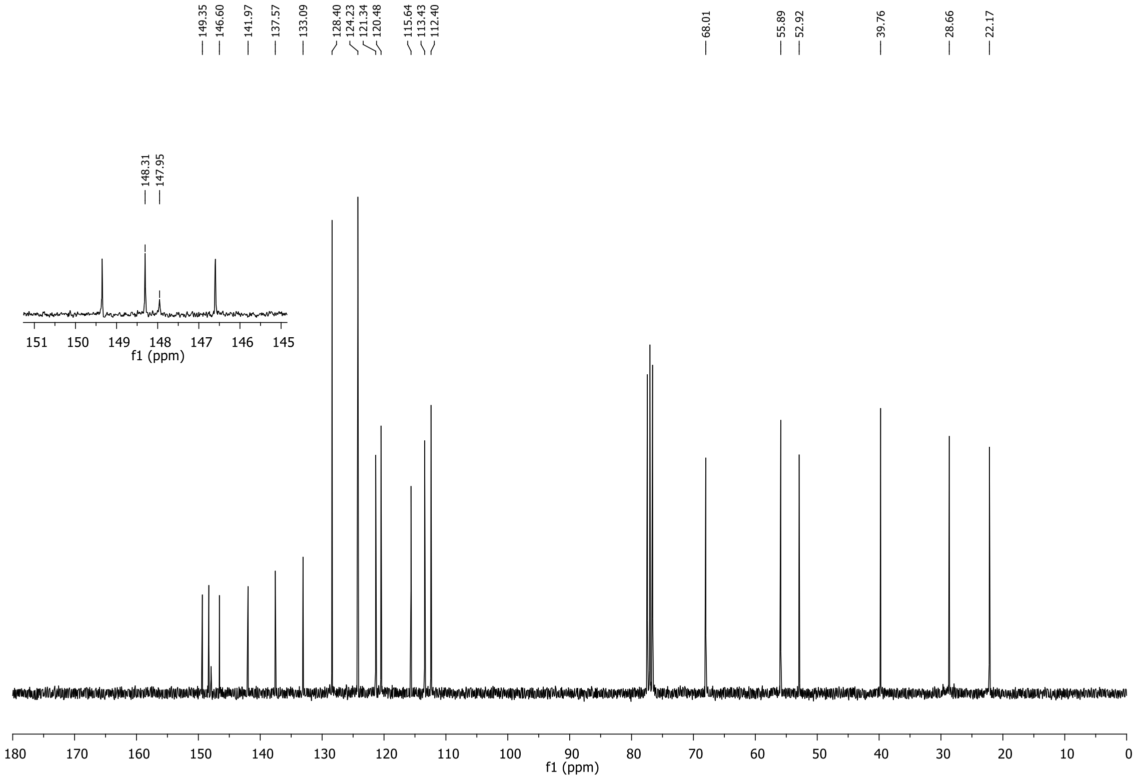

Fig. BH. ^{13}C NMR spectrum (75 MHz, $CDCl_3$) of 4-(3-(4-allyl-2-methoxyphenoxy)propyl)-1-(4-nitrobenzyl)-*1H*-1,2,3-triazole (**42**).

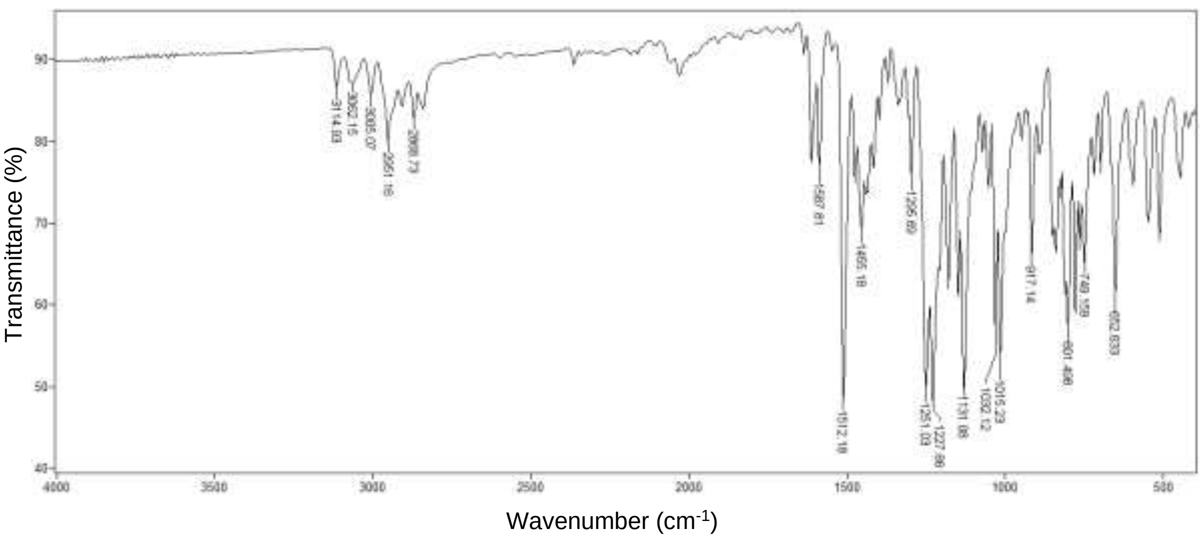

Fig. BI. IR spectrum (ATR) of 4-(3-(4-allyl-2-methoxyphenoxy)propyl)-1-(4-methoxybenzyl)-*1H*-1,2,3-triazole (**43**).

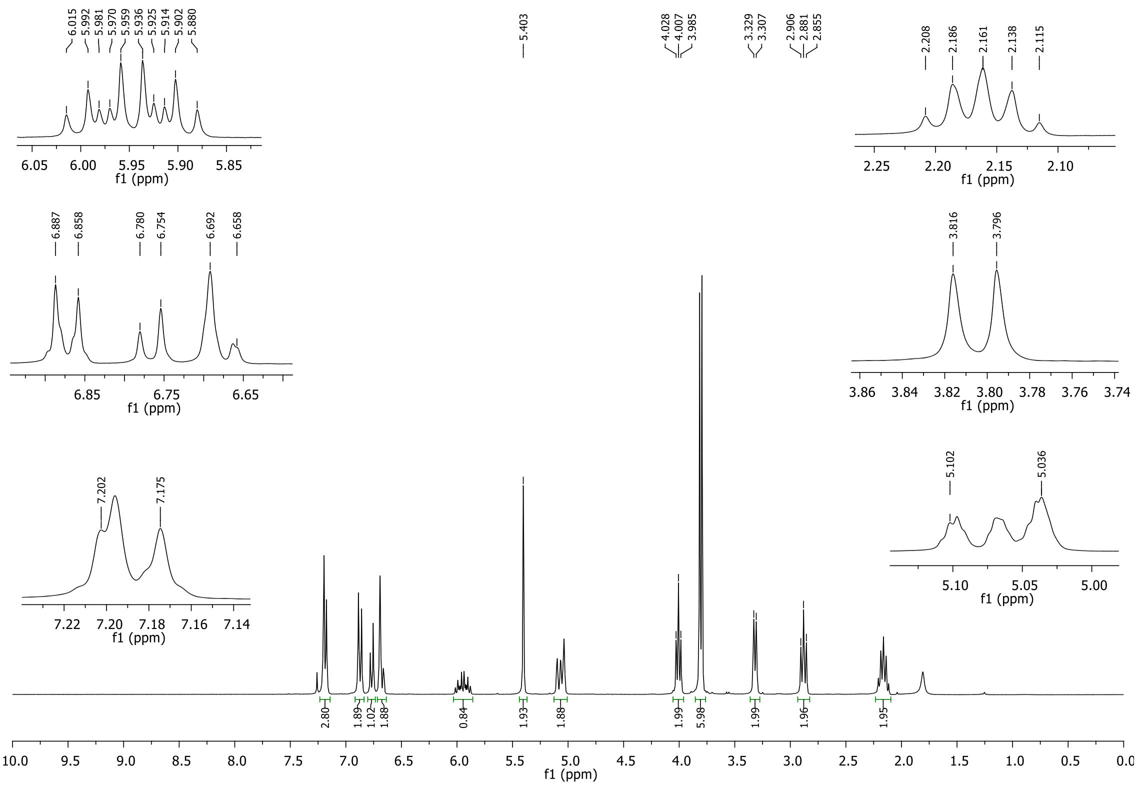

Fig. BJ. ^{1}H NMR spectrum (300 MHz, $CDCl_3$) of 4-(3-(4-allyl-2-methoxyphenoxy)propyl)-1-(4-methoxybenzyl)-*1H*-1,2,3-triazole (**43**).

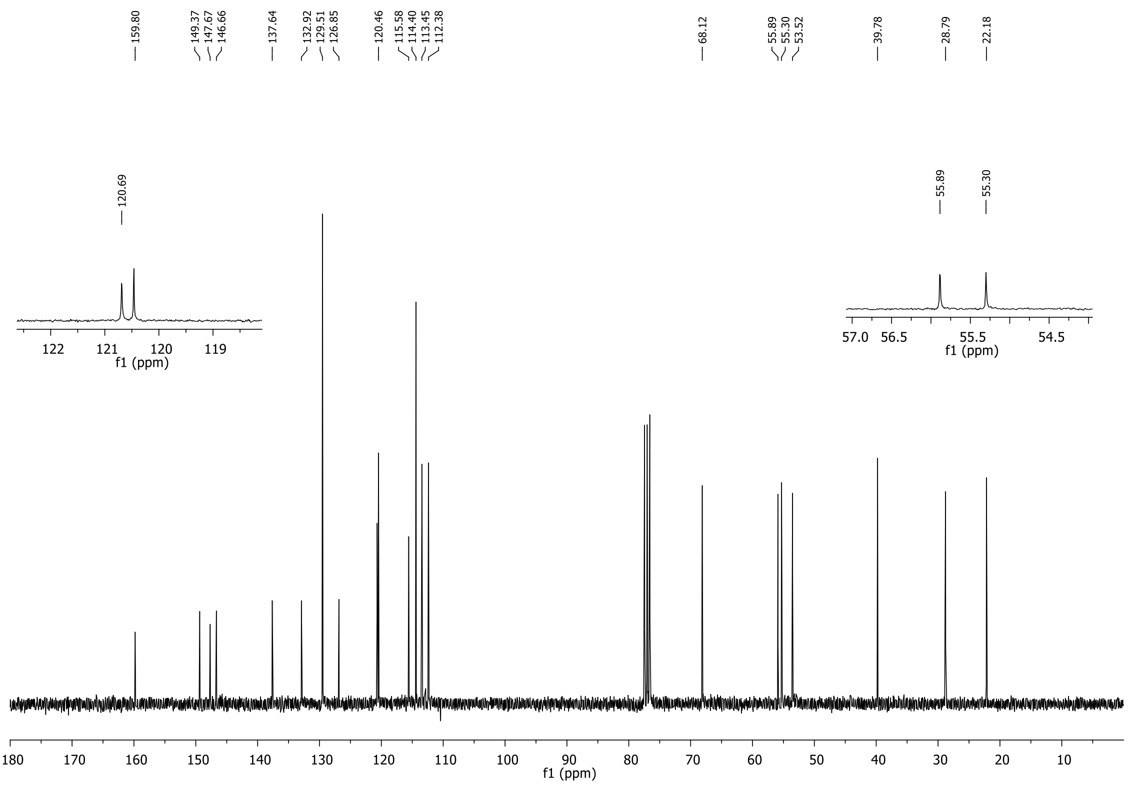

Fig. BK. ^{13}C NMR spectrum (75 MHz, $CDCl_3$) of 4-(3-(4-allyl-2-methoxyphenoxy)propyl)-1-(4-methoxybenzyl)-*1H*-1,2,3-triazole (**43**).

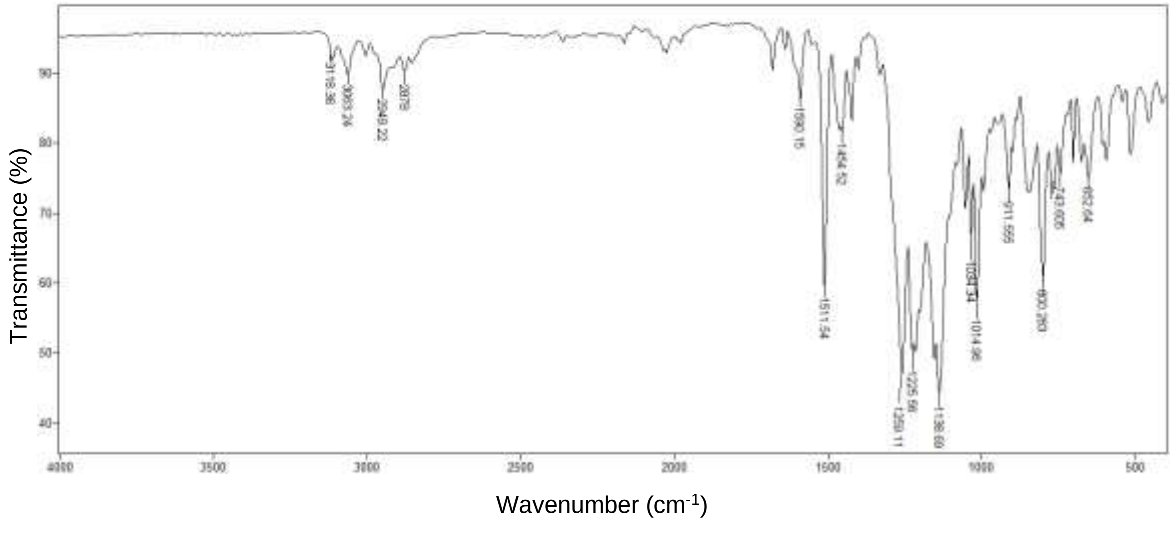

Fig. BL. IR spectrum (ATR) of 4-(3-(4-allyl-2-methoxyphenoxy)propyl)-1-(4-(trifluoromethoxy)benzyl)-*1H*-1,2,3-triazole (**44**).

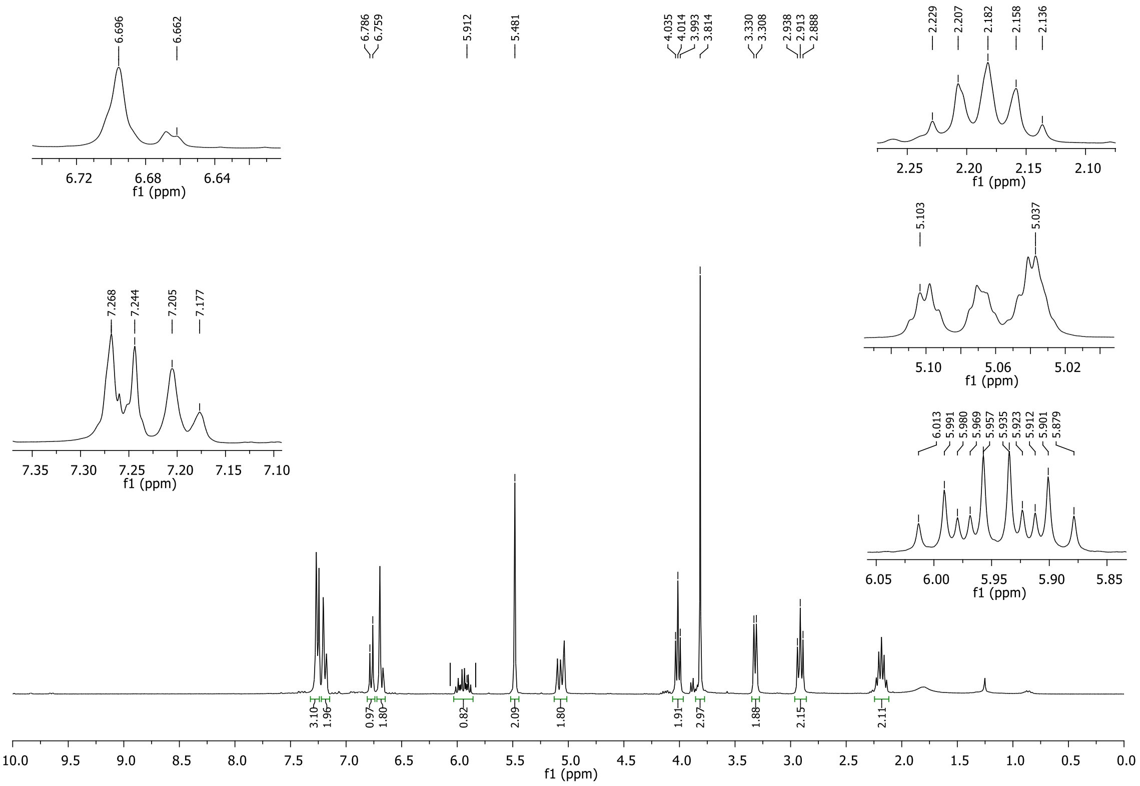

Fig. BM. ^{1}H NMR spectrum (300 MHz, $CDCl_3$) of 4-(3-(4-allyl-2-methoxyphenoxy)propyl)-1-(4-(trifluoromethoxy)benzyl)-*1H*-1,2,3-triazole (**44**).

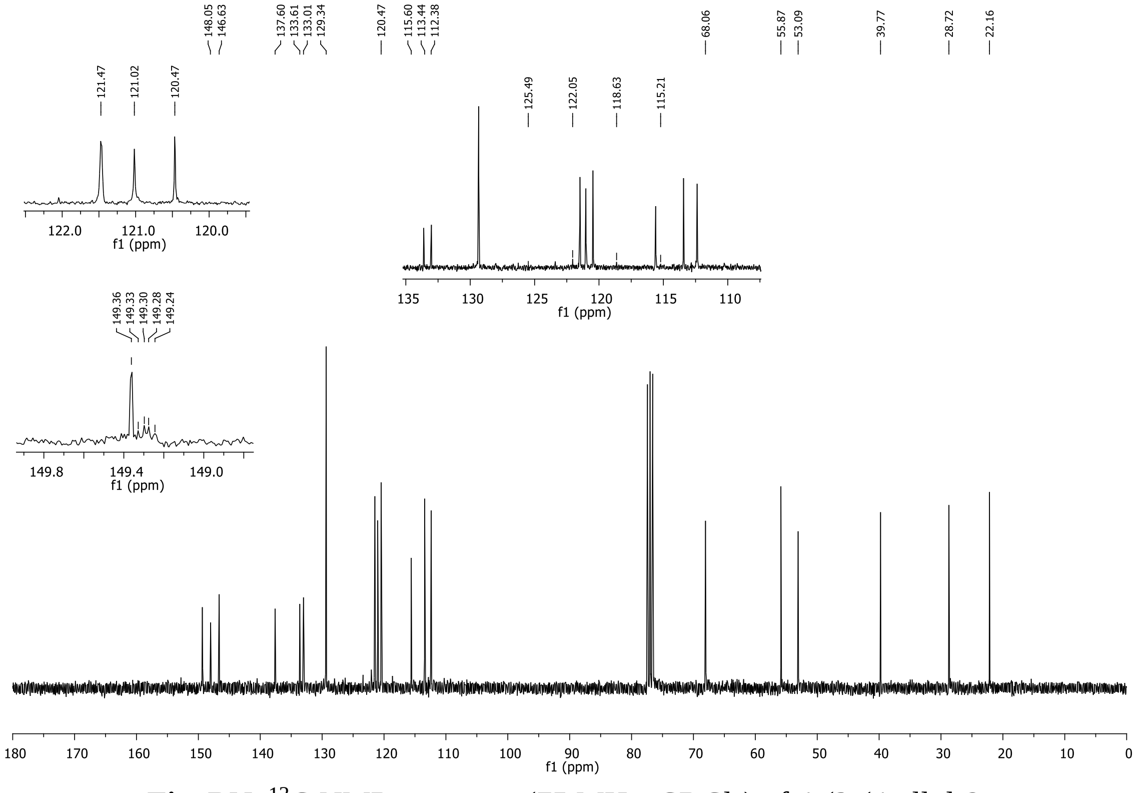

Fig. BN. ^{13}C NMR spectrum (75 MHz, $CDCl_3$) of 4-(3-(4-allyl-2-methoxyphenoxy)propyl)-1-(4-(trifluoromethoxy)benzyl)-*1H*-1,2,3-triazole (**44**).

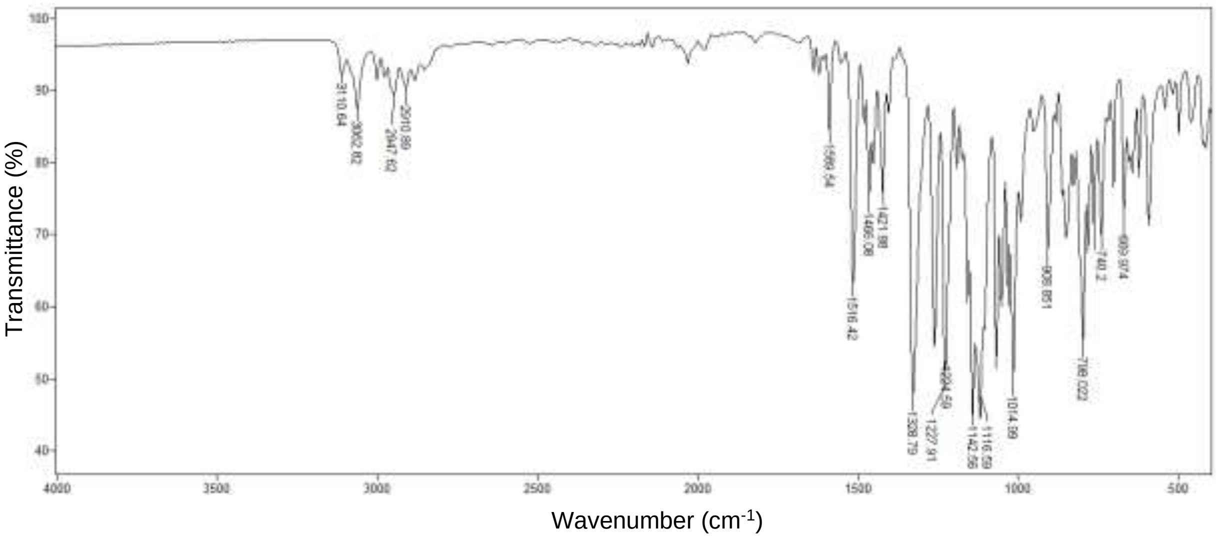

Fig. BO. IR spectrum (ATR) of 4-(3-(4-allyl-2-methoxyphenoxy)propyl)-1-(4-(trifluoromethyl)benzyl)-*1H*-1,2,3-triazole (**45**).

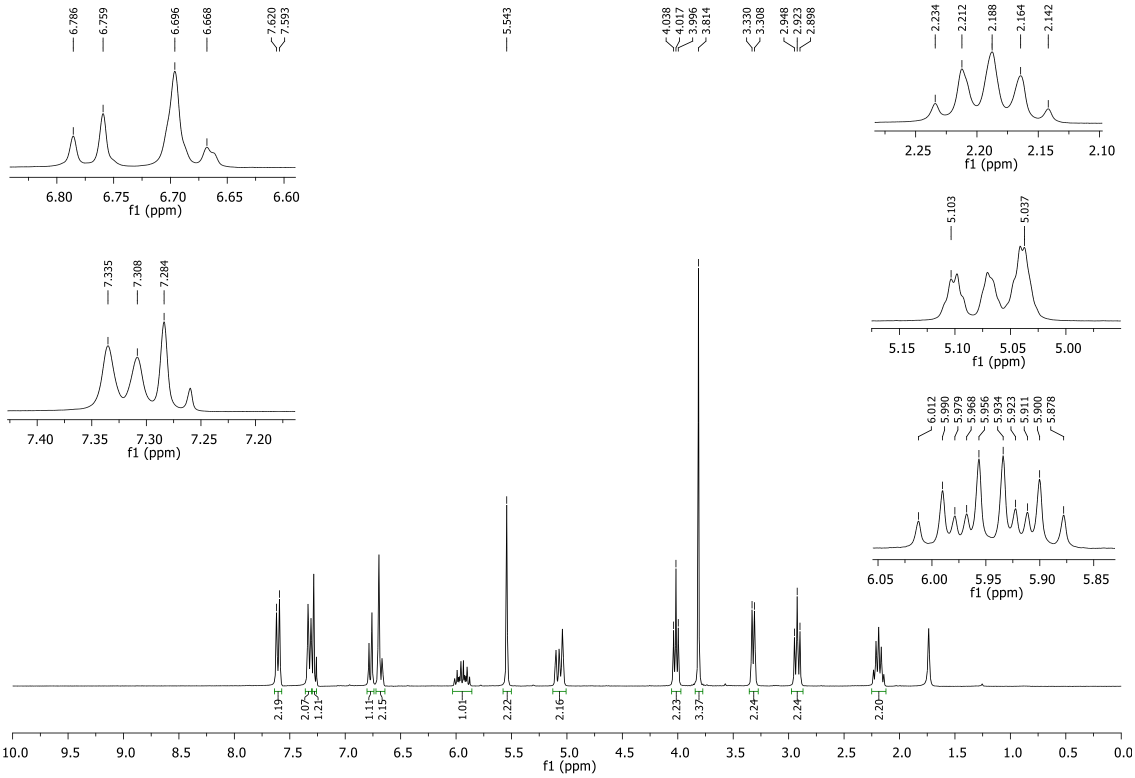

Fig. BP. ^{1}H NMR spectrum (300 MHz, $CDCl_3$) of 4-(3-(4-allyl-2-methoxyphenoxy)propyl)-1-(4-(trifluoromethyl)benzyl)-*1H*-1,2,3-triazole (**45**).

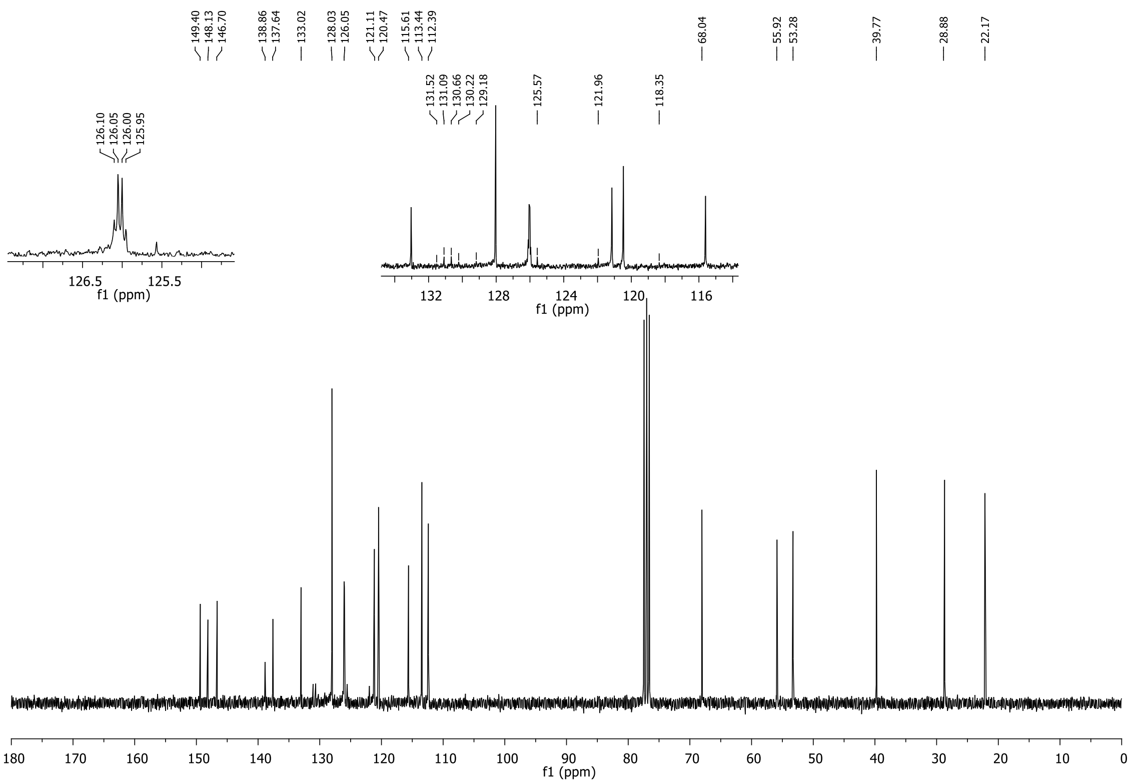

Fig. BQ. ^{13}C NMR spectrum (75 MHz, $CDCl_3$) of 4-(3-(4-allyl-2-methoxyphenoxy)propyl)-1-(4-(trifluoromethyl)benzyl)-*1H*-1,2,3-triazole (**45**).

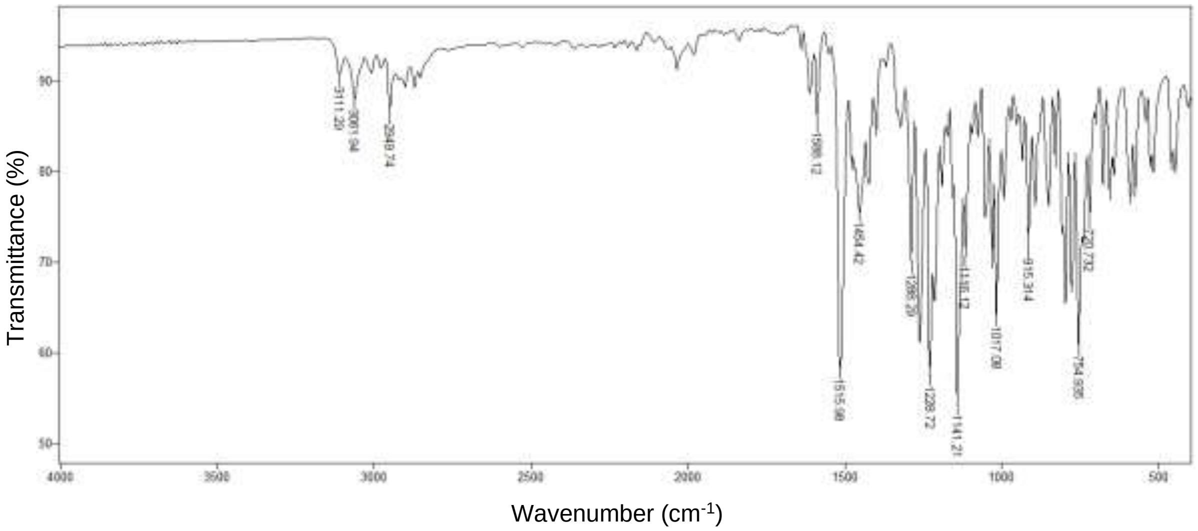

Fig. BR. IR spectrum (ATR) of 4-(3-(4-allyl-2-methoxyphenoxy)propyl)-1-(3,4-difluorobenzyl)-*1H*-1,2,3-triazole (**46**).

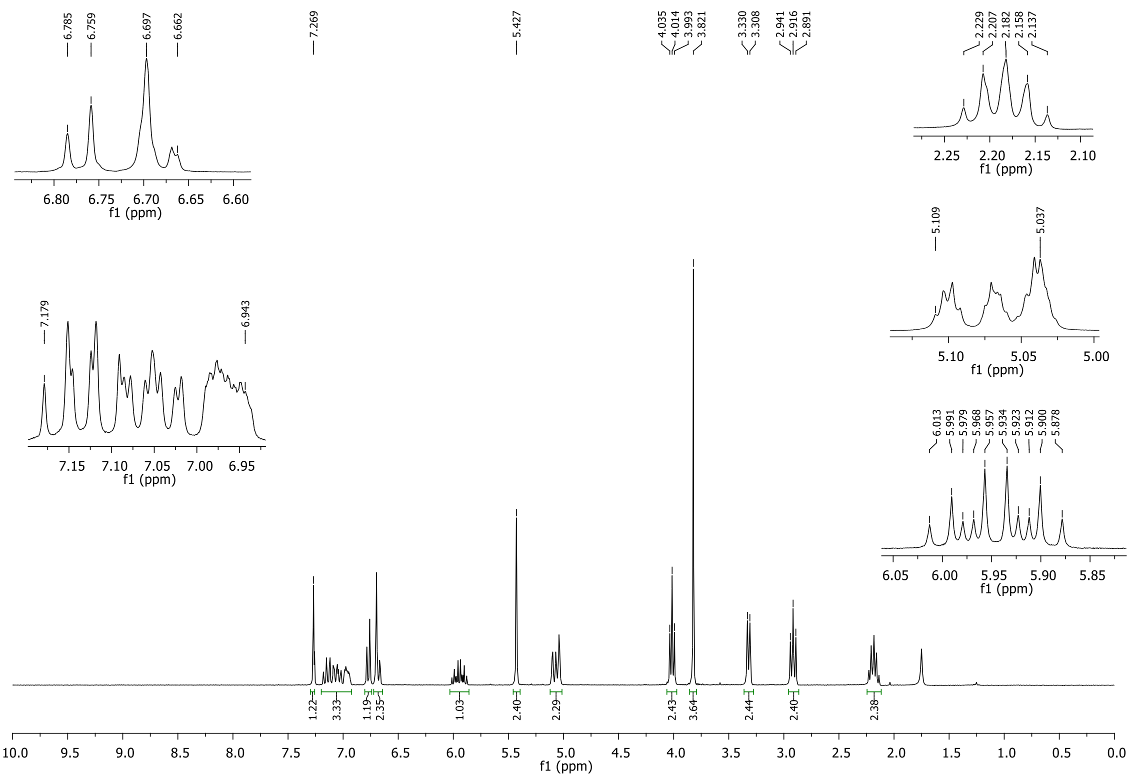

Fig. BS. ^{1}H NMR spectrum (300 MHz, $CDCl_3$) of 4-(3-(4-allyl-2-methoxyphenoxy)propyl)-1-(3,4-difluorobenzyl)-*1H*-1,2,3-triazole (**46**).

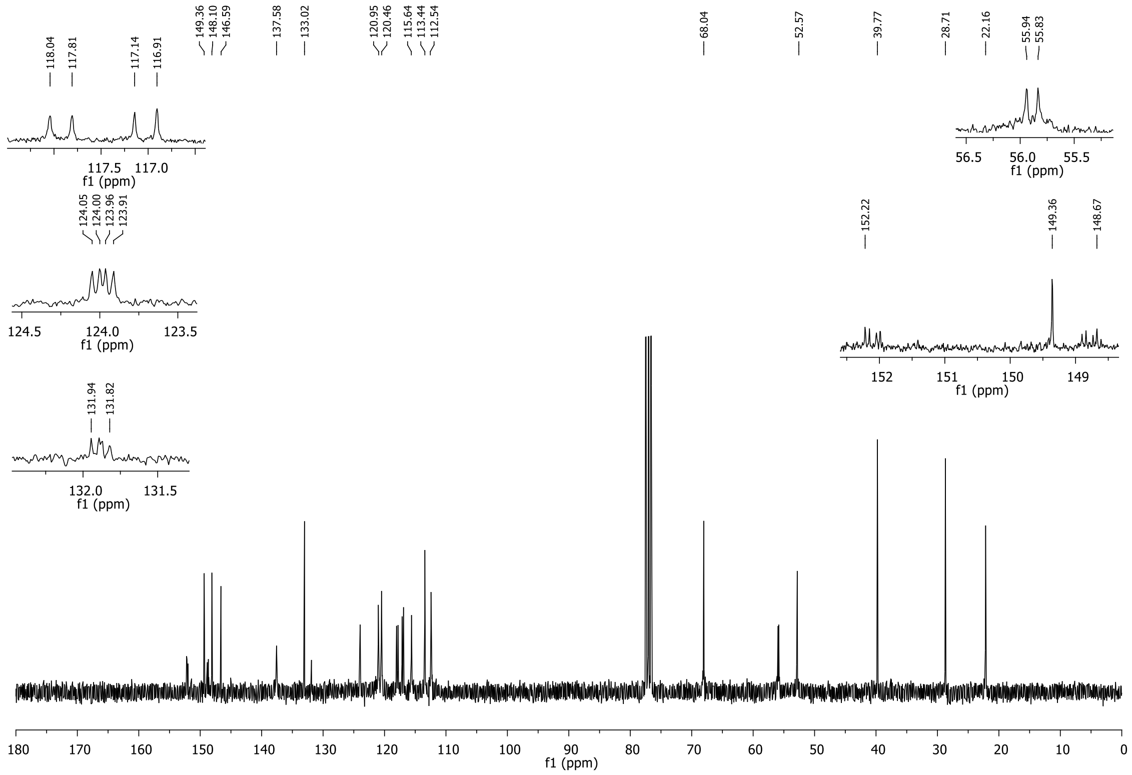

Fig. BT. ^{13}C NMR spectrum (75 MHz, $CDCl_3$) of 4-(3-(4-allyl-2-methoxyphenoxy)propyl)-1-(3,4-difluorobenzyl)-*1H*-1,2,3-triazole (**46**).

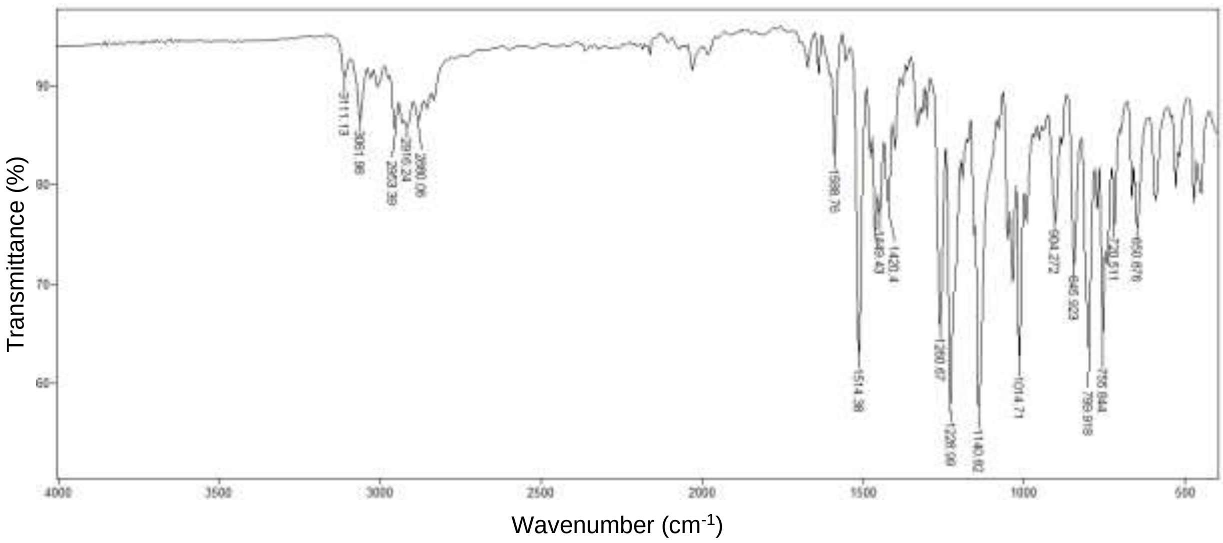

Fig. BU. IR spectrum (ATR) of 4-(3-(4-allyl-2-methoxyphenoxy)propyl)-1-(4-methylbenzyl)-*1H*-1,2,3-triazole (**47**).

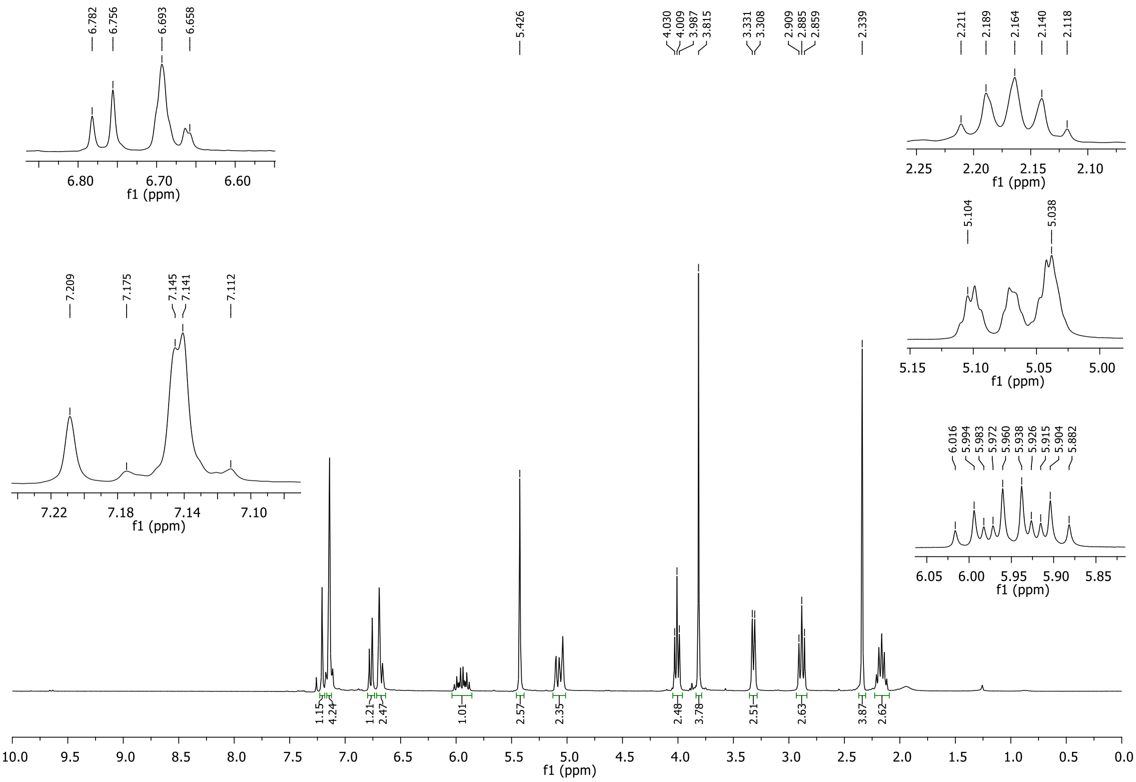

Fig. BV. ^{1}H NMR spectrum (300 MHz, $CDCl_3$) of 4-(3-(4-allyl-2-methoxyphenoxy)propyl)-1-(4-methylbenzyl)-*1H*-1,2,3-triazole (**47**).

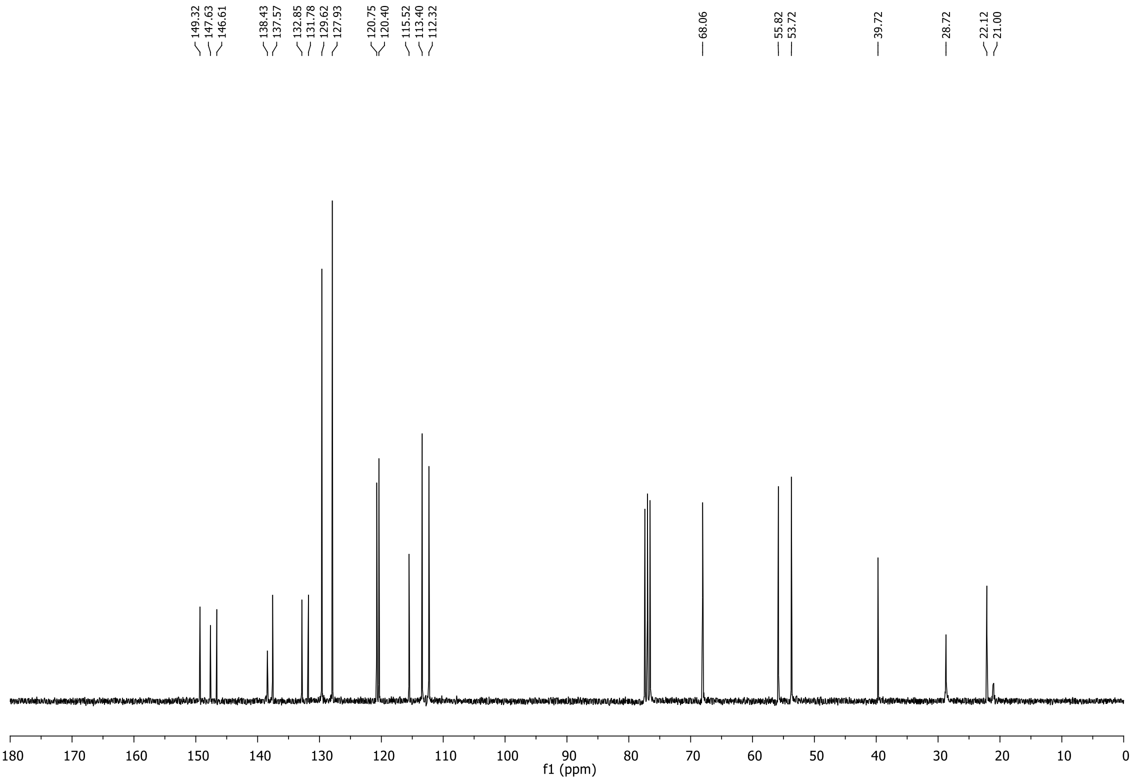

Fig. BX. ^{13}C NMR spectrum (75 MHz, $CDCl_3$) of 4-(3-(4-allyl-2-methoxyphenoxy)propyl)-1-(4-methylbenzyl)-*1H*-1,2,3-triazole (**47**).

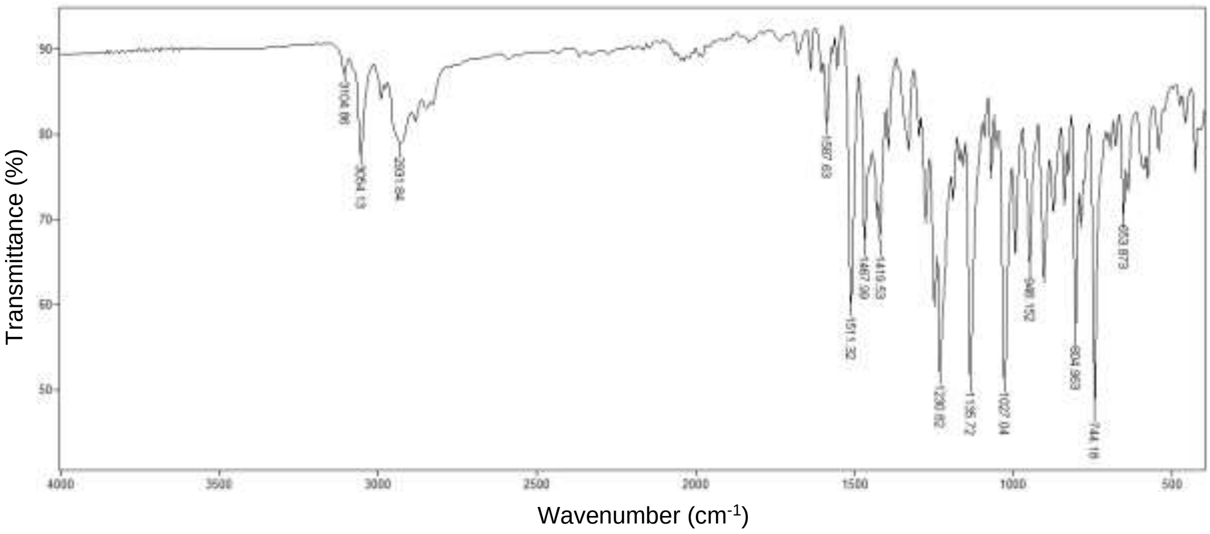

Fig. BW. IR spectrum (ATR) of 4-(3-(4-allyl-2-methoxyphenoxy)propyl)-1-(2-bromobenzyl)-*1H*-1,2,3-triazole (**48**).

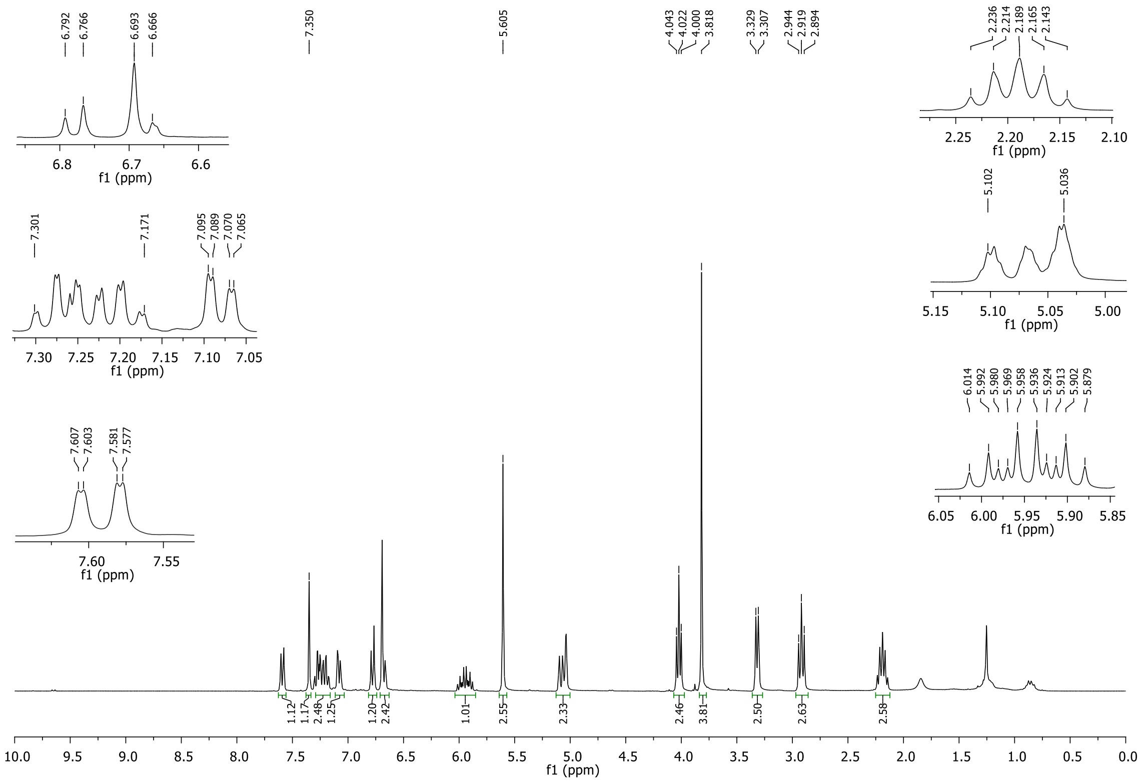

Fig. BY. ^{1}H NMR spectrum (300 MHz, $CDCl_3$) of 4-(3-(4-allyl-2-methoxyphenoxy)propyl)-1-(2-bromobenzyl)-*1H*-1,2,3-triazole (**48**).

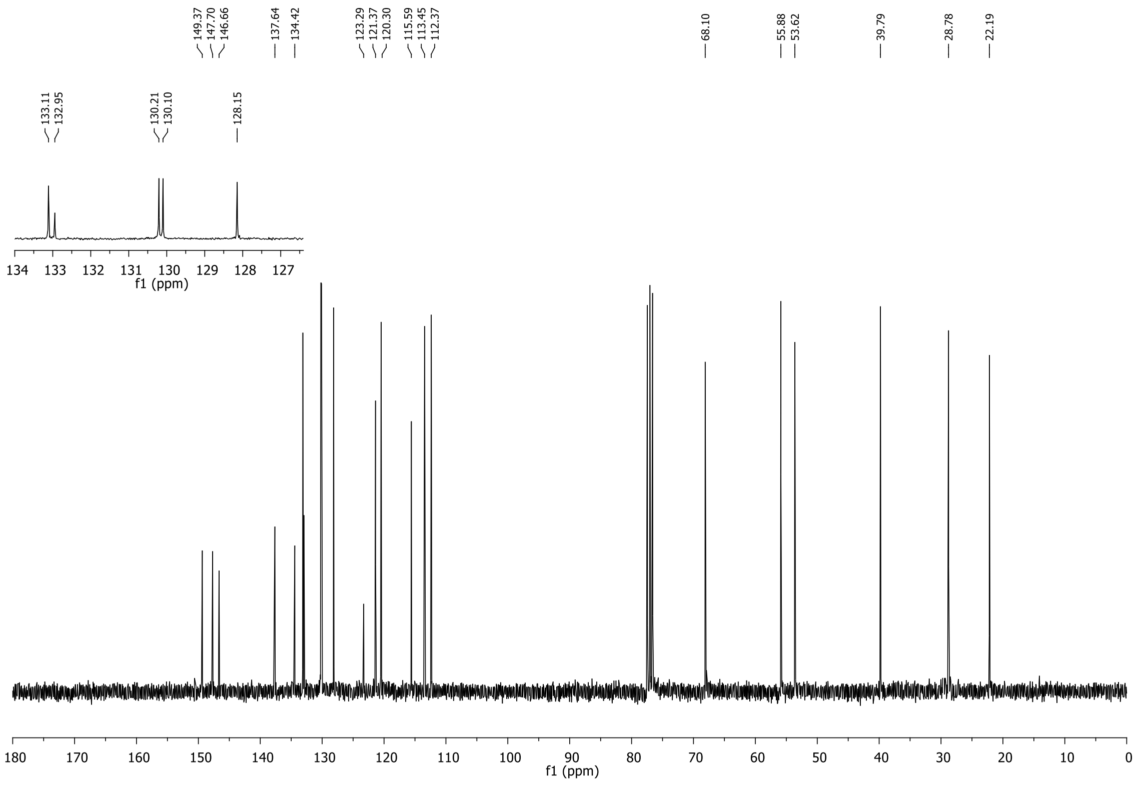

Fig. BZ. ^{13}C NMR spectrum (75 MHz, $CDCl_3$) of 4-(3-(4-allyl-2-methoxyphenoxy)propyl)-1-(2-bromobenzyl)-*1H*-1,2,3-triazole (**48**).

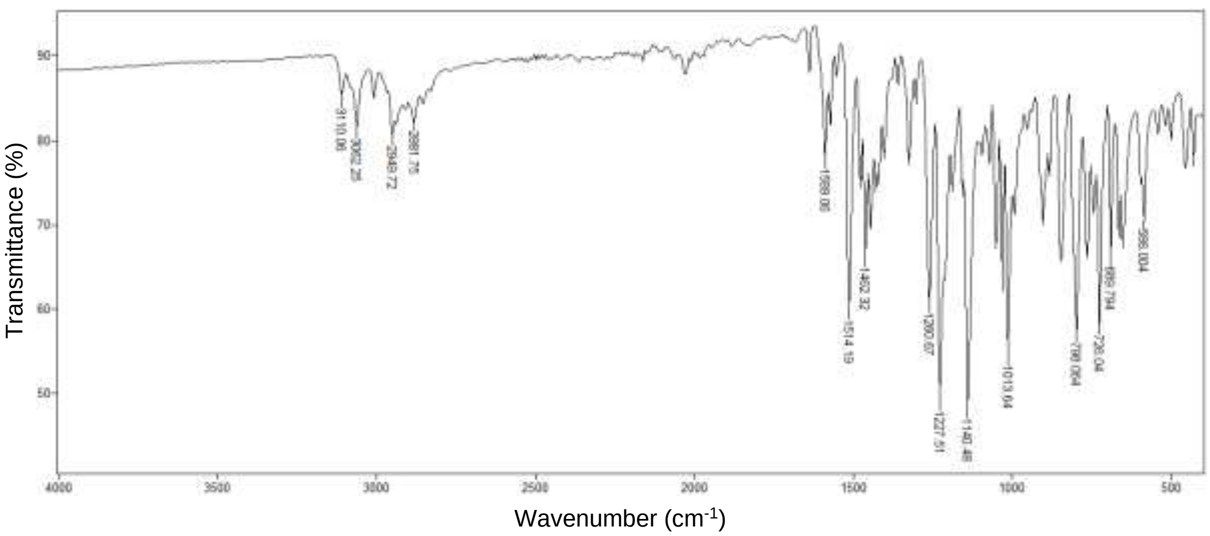

Fig. CA. IR spectrum (ATR) of 4-(3-(4-allyl-2-methoxyphenoxy)propyl)-1-(3-bromobenzyl)-*1H*-1,2,3-triazole (**49**).

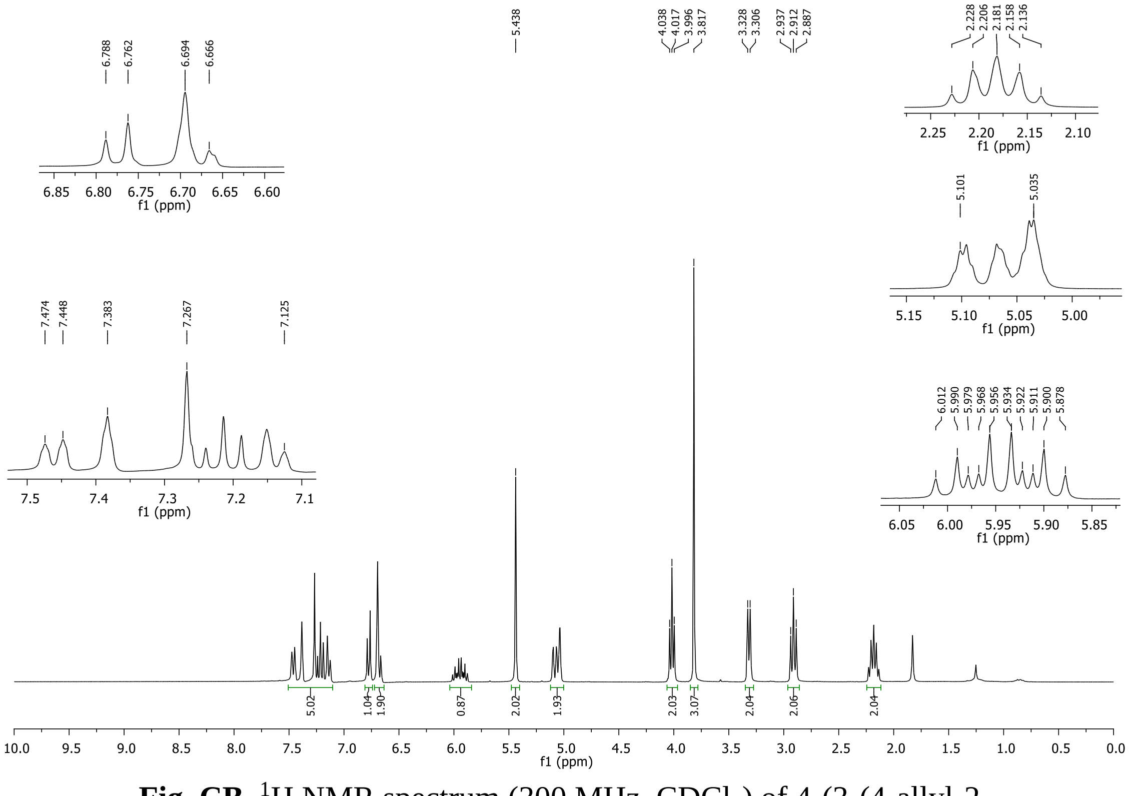

Fig. CB. ^{1}H NMR spectrum (300 MHz, $CDCl_3$) of 4-(3-(4-allyl-2-methoxyphenoxy)propyl)-1-(3-bromobenzyl)-*1H*-1,2,3-triazole (**49**).

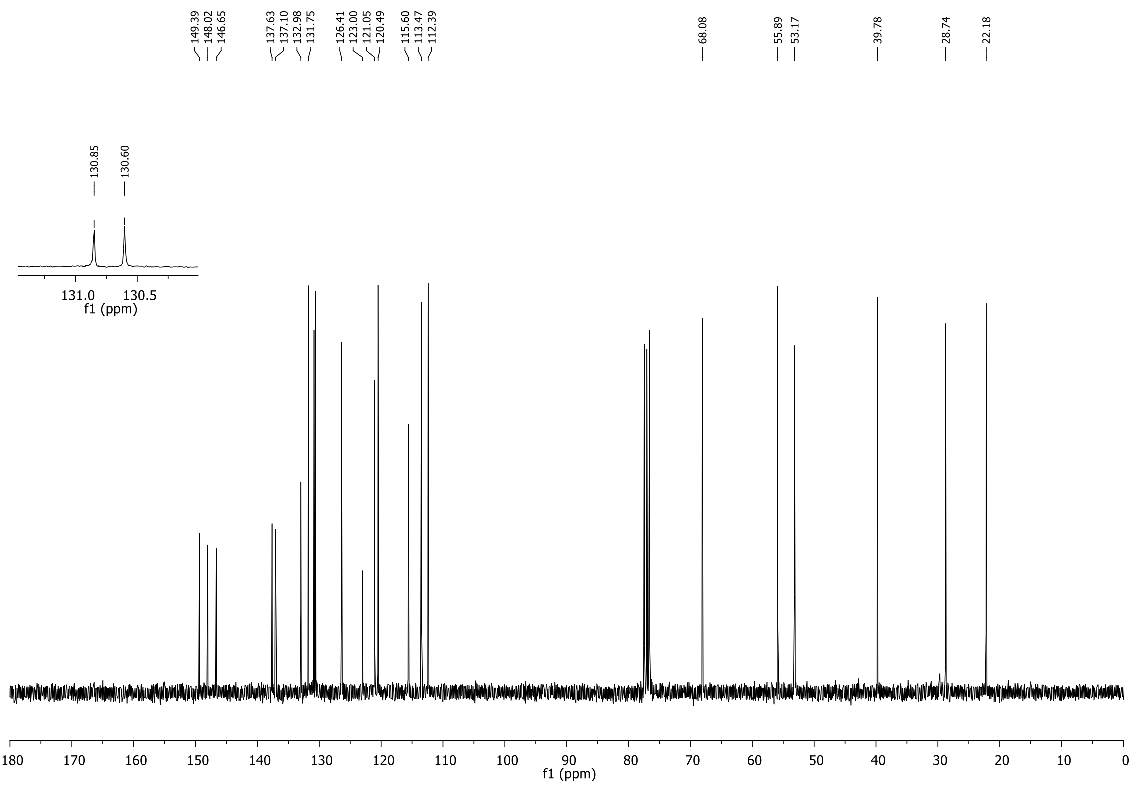

Fig. CC. ^{13}C NMR spectrum (75 MHz, $CDCl_3$) of of 4-(3-(4-allyl-2-methoxyphenoxy)propyl)-1-(3-bromobenzyl)-*1H*-1,2,3-triazole (**49**).

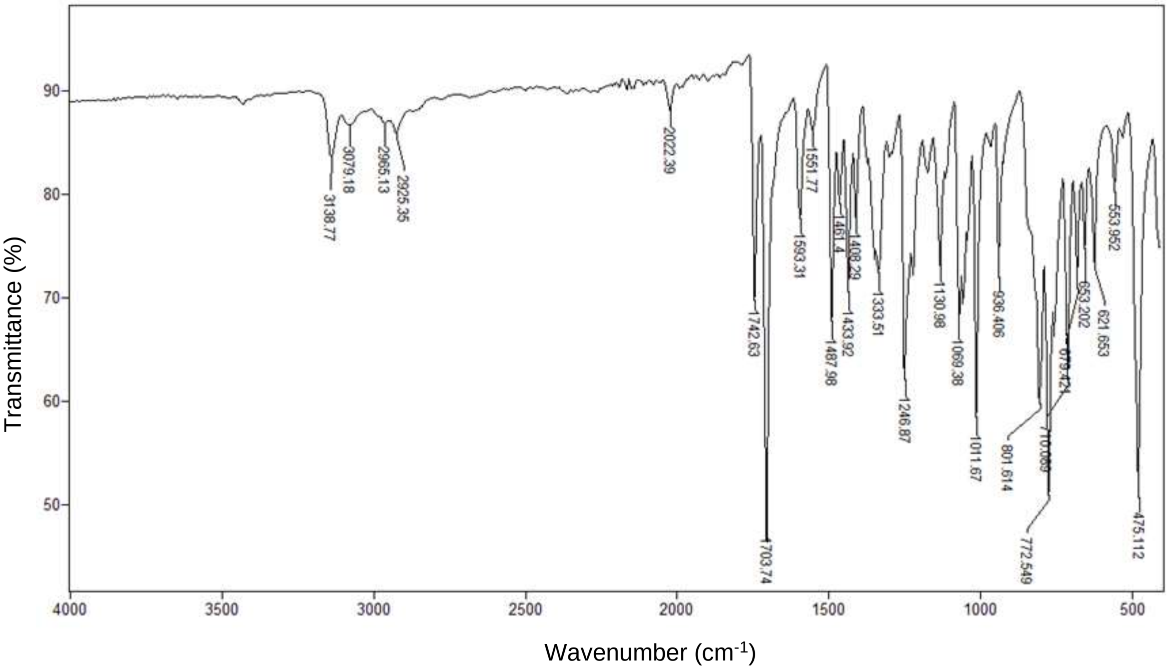

Fig. CD. IR spectrum (ATR) of 2,2-*bis*((1-(4-bromobenzyl)-1*H*-1,2,3-triazol-4-yl)methyl)-1*H*-indene-1,3(2*H*)-dione (**51**).

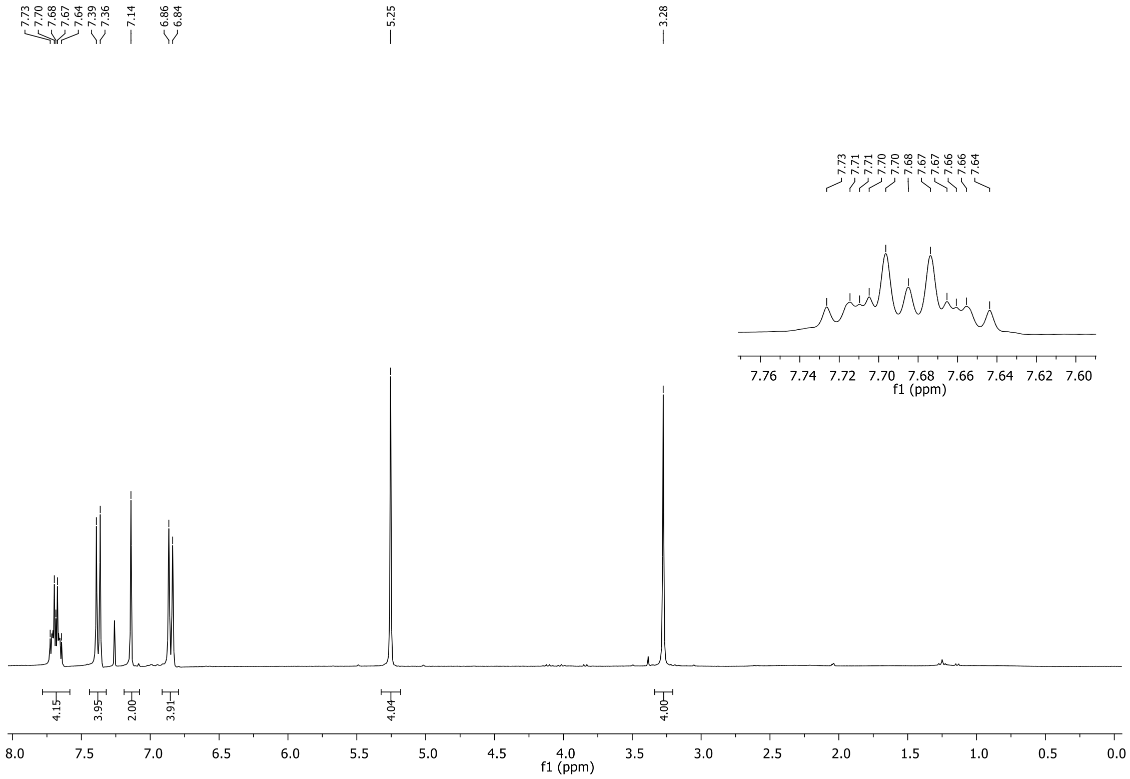

Fig. CE. ^{1}H NMR spectrum (300 MHz, $CDCl_3$) of 2,2-*bis*((1-(4-bromobenzyl)-1*H*-1,2,3-triazol-4-yl)methyl)-1*H*-indene-1,3(2*H*)-dione (**51**).

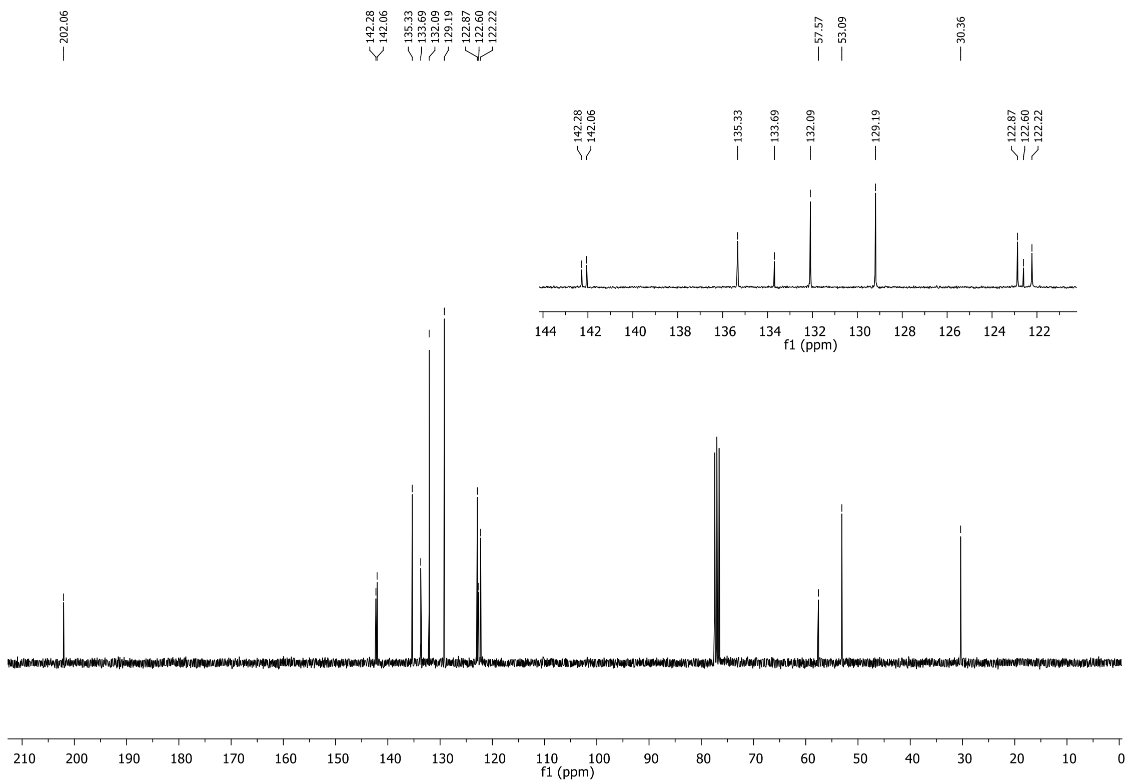

Fig. CF. ^{13}C NMR spectrum (75 MHz, $CDCl_3$) of 2,2-*bis*((1-(4-bromobenzyl)-1*H*-1,2,3-triazol-4-yl)methyl)-1*H*-indene-1,3(2*H*)-dione (**51**).

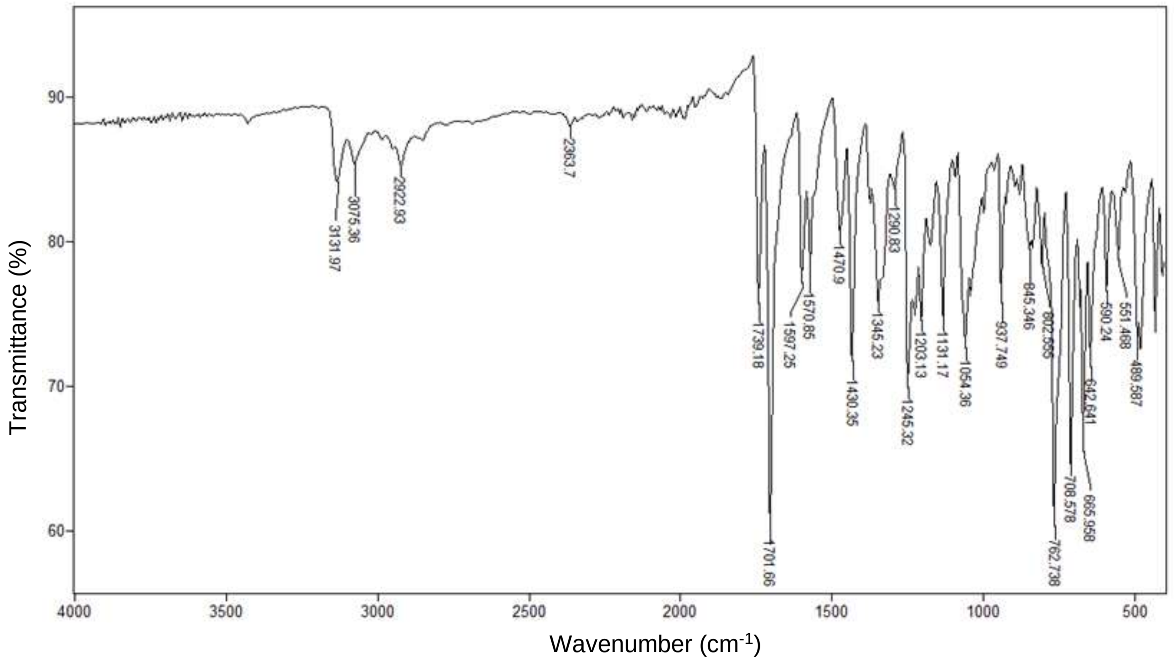

Fig. CG. IR spectrum (ATR) of 2,2-*bis*((1-(3-bromobenzyl)-1*H*-1,2,3-triazol-4-yl)methyl)-1*H*-indene-1,3(2*H*)-dione (**52**).

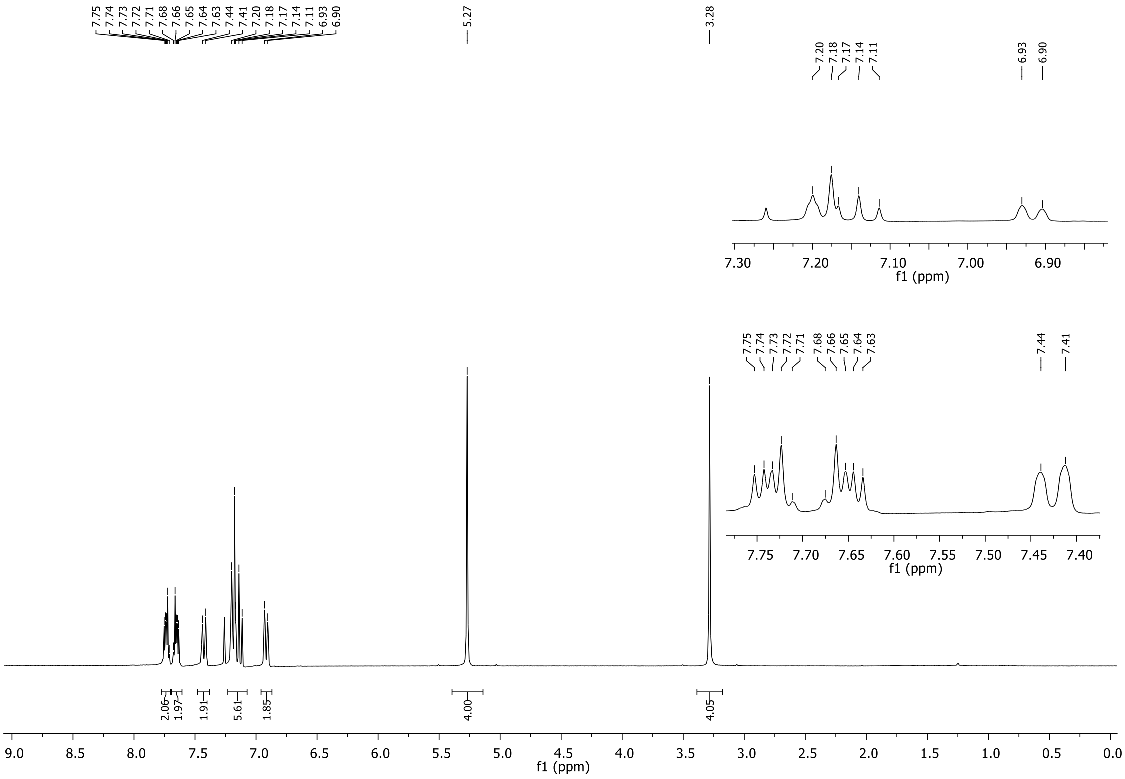

Fig. CH. ^{1}H NMR spectrum (300 MHz, $CDCl_3$) of 2,2-*bis*((1-(3-bromobenzyl)-1*H*-1,2,3-triazol-4-yl)methyl)-1*H*-indene-1,3(2*H*)-dione (**52**).

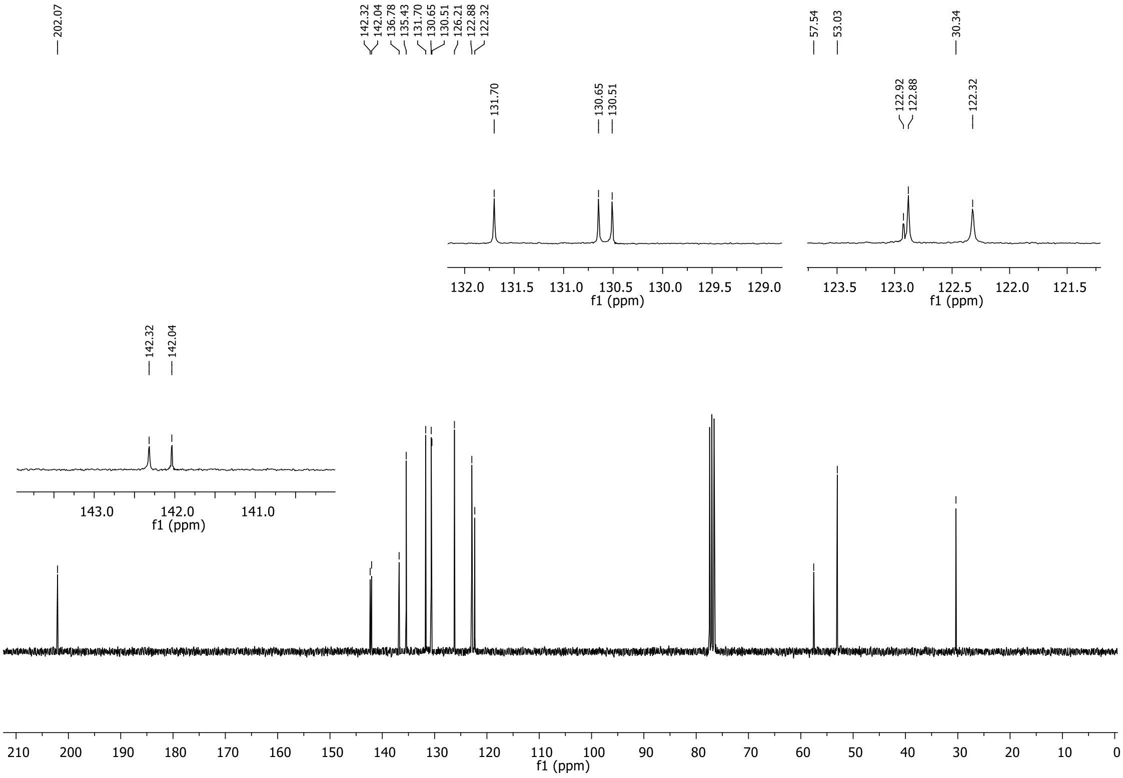

Fig. CI. ^{13}C NMR spectrum (75 MHz, $CDCl_3$) of 2,2-*bis*((1-(3-bromobenzyl)-1*H*-1,2,3-triazol-4-yl)methyl)-1*H*-indene-1,3(2*H*)-dione (**52**).

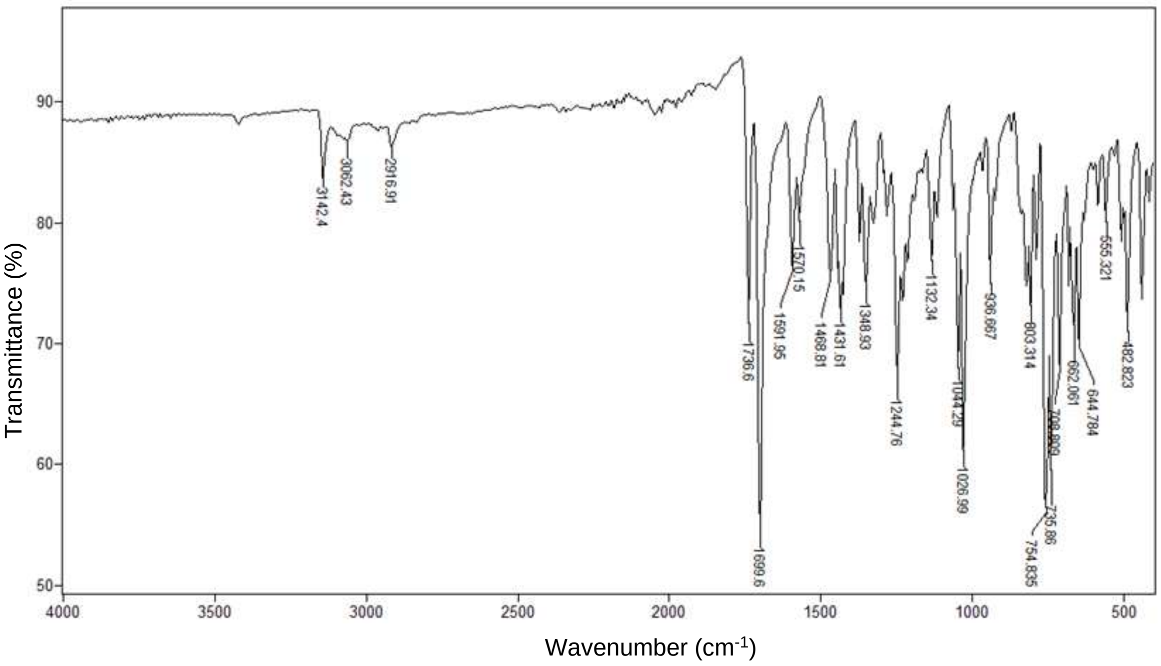

Fig. CJ. IR spectrum (ATR) of 2,2-*bis*((1-(2-bromobenzyl)-1*H*-1,2,3-triazol-4-yl)methyl)-1*H*-indene-1,3(2*H*)-dione (**53**).

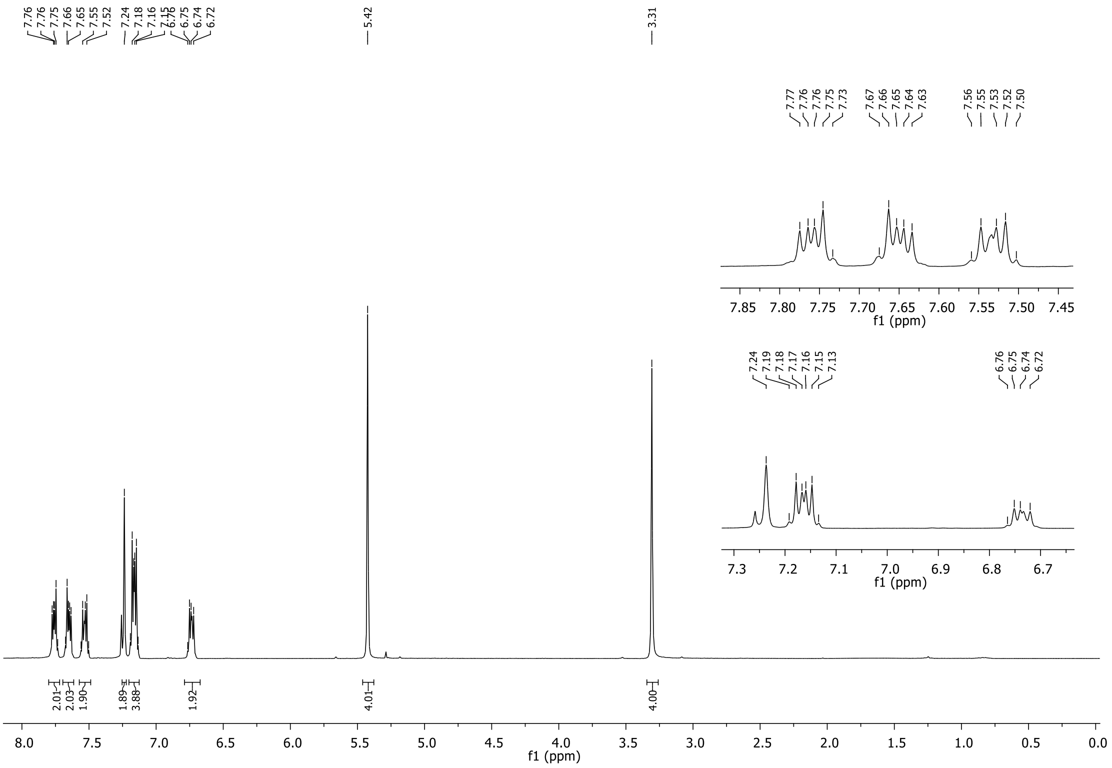

Fig. CK. ^{1}H NMR spectrum (300 MHz, $CDCl_3$) of 2,2-*bis*((1-(2-bromobenzyl)-1*H*-1,2,3-triazol-4-yl)methyl)-1*H*-indene-1,3(2*H*)-dione (**53**).

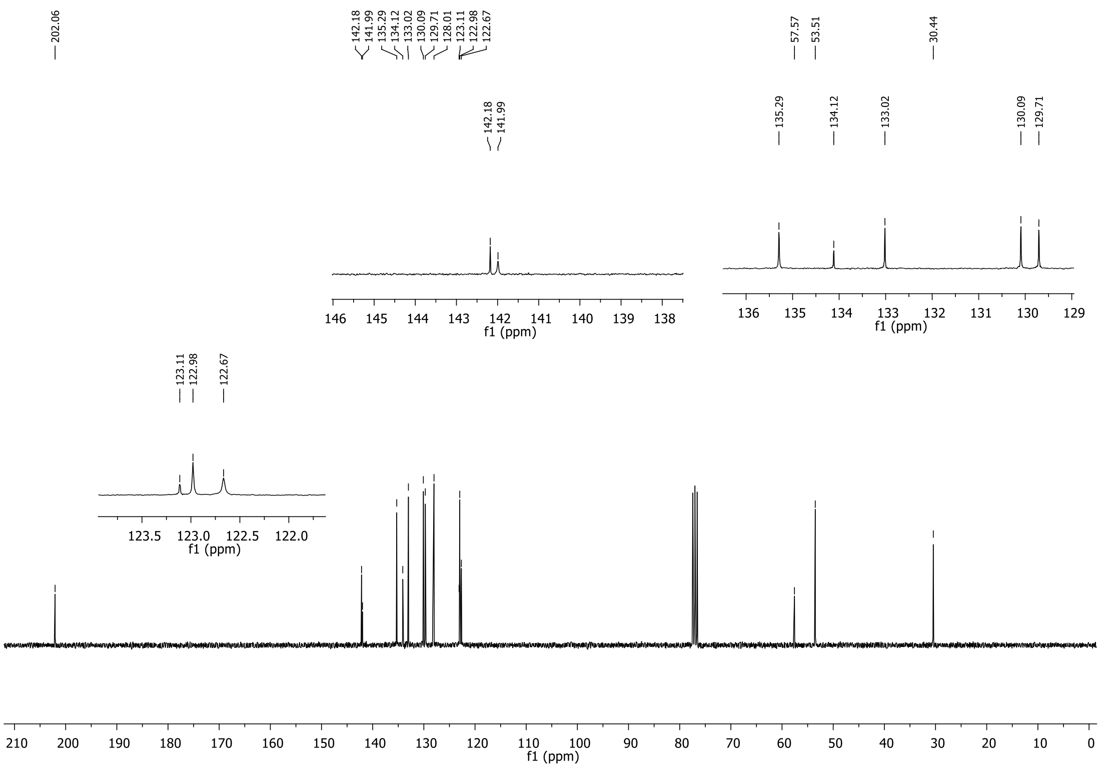

Fig. CL. ^{13}C NMR spectrum (75 MHz, $CDCl_3$) of 2,2-*bis*((1-(2-bromobenzyl)-1*H*-1,2,3-triazol-4-yl)methyl)-1*H*-indene-1,3(2*H*)-dione (**53**).

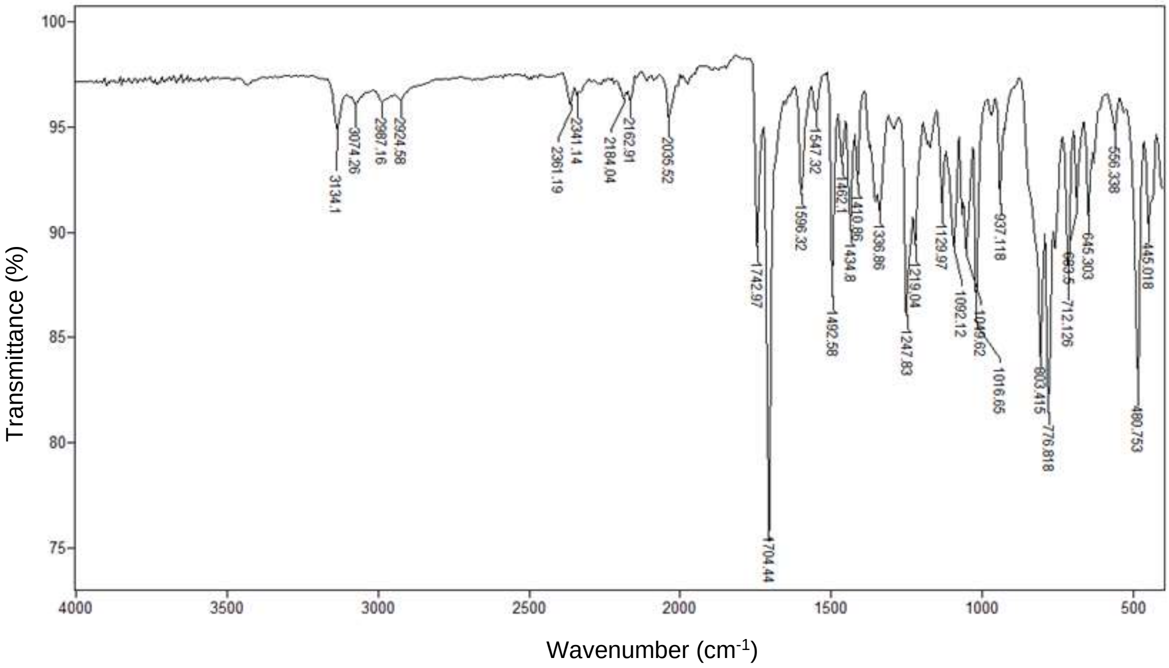

Fig. CM. IR spectrum (ATR) of 2,2-*bis*((1-(4-chlorobenzyl)-1*H*-1,2,3-triazol-4-yl)methyl)-1*H*-indene-1,3(2*H*)-dione (**54**).

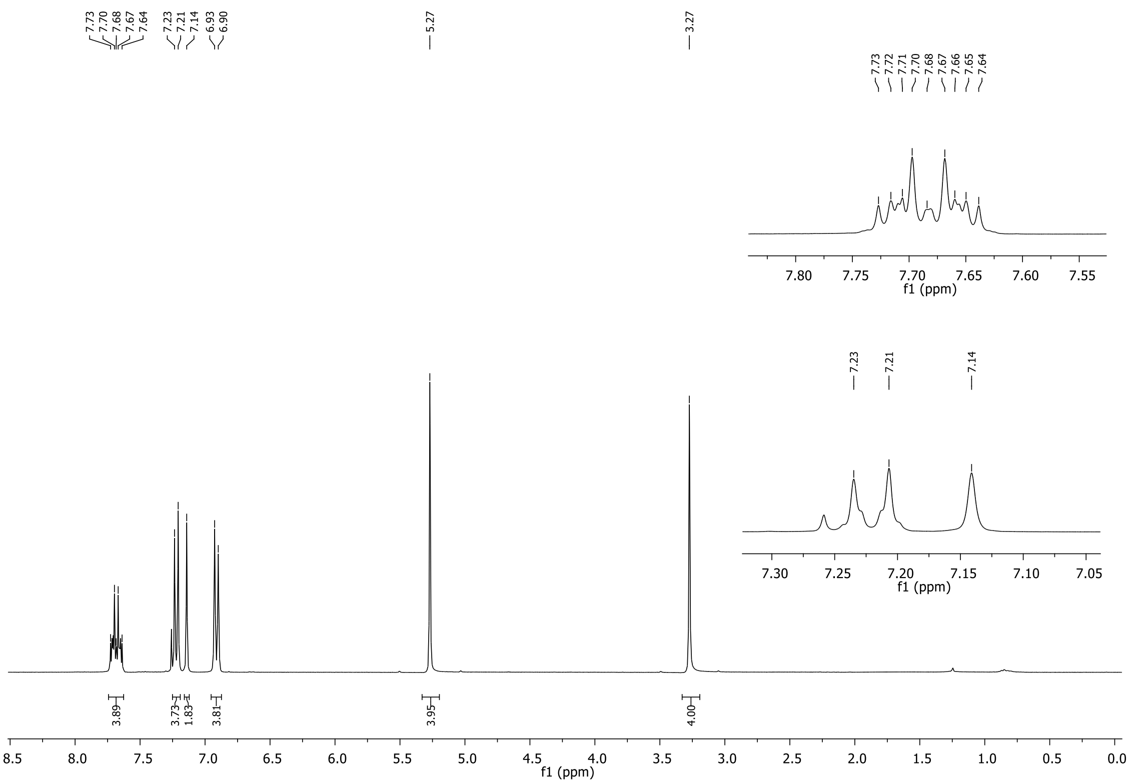

Fig. CN. ^{1}H NMR spectrum (300 MHz, $CDCl_3$) of 2,2-*bis*((1-(4-chlorobenzyl)-1*H*-1,2,3-triazol-4-yl)methyl)-1*H*-indene-1,3(2*H*)-dione (**54**).

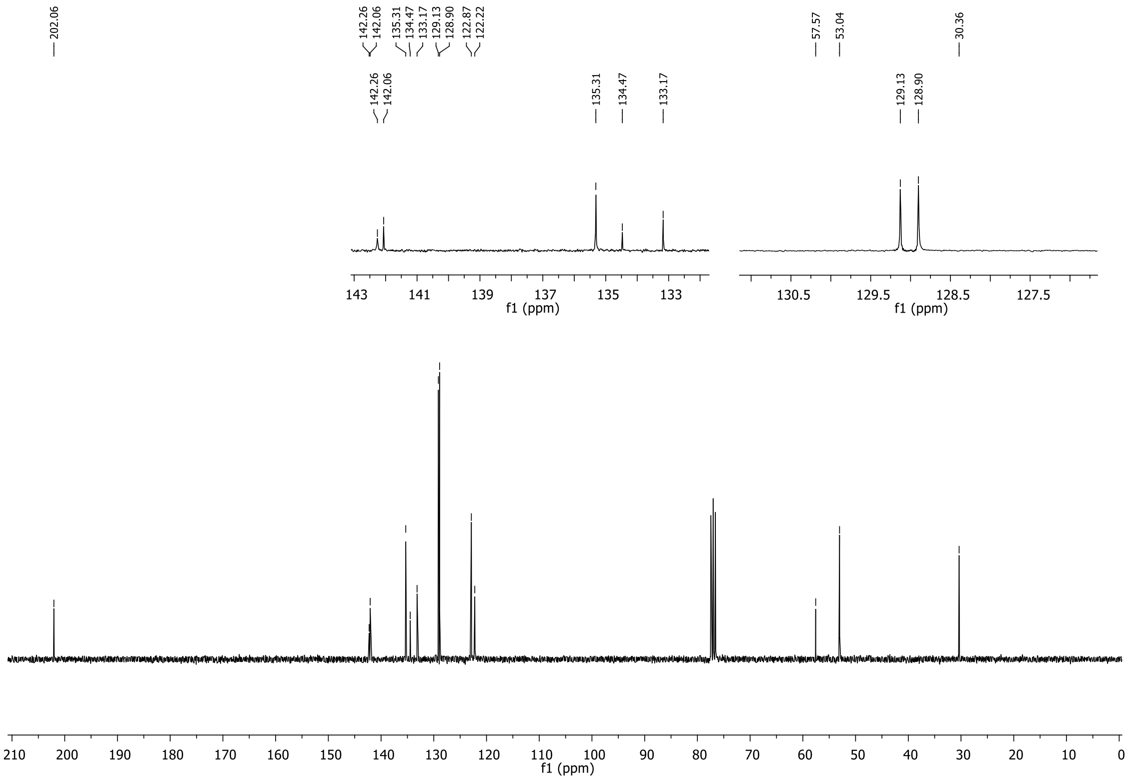

Fig. CO. ^{13}C NMR spectrum (75 MHz, $CDCl_3$) of 2,2-*bis*((1-(4-chlorobenzyl)-1*H*-1,2,3-triazol-4-yl)methyl)-1*H*-indene-1,3(2*H*)-dione (**54**).

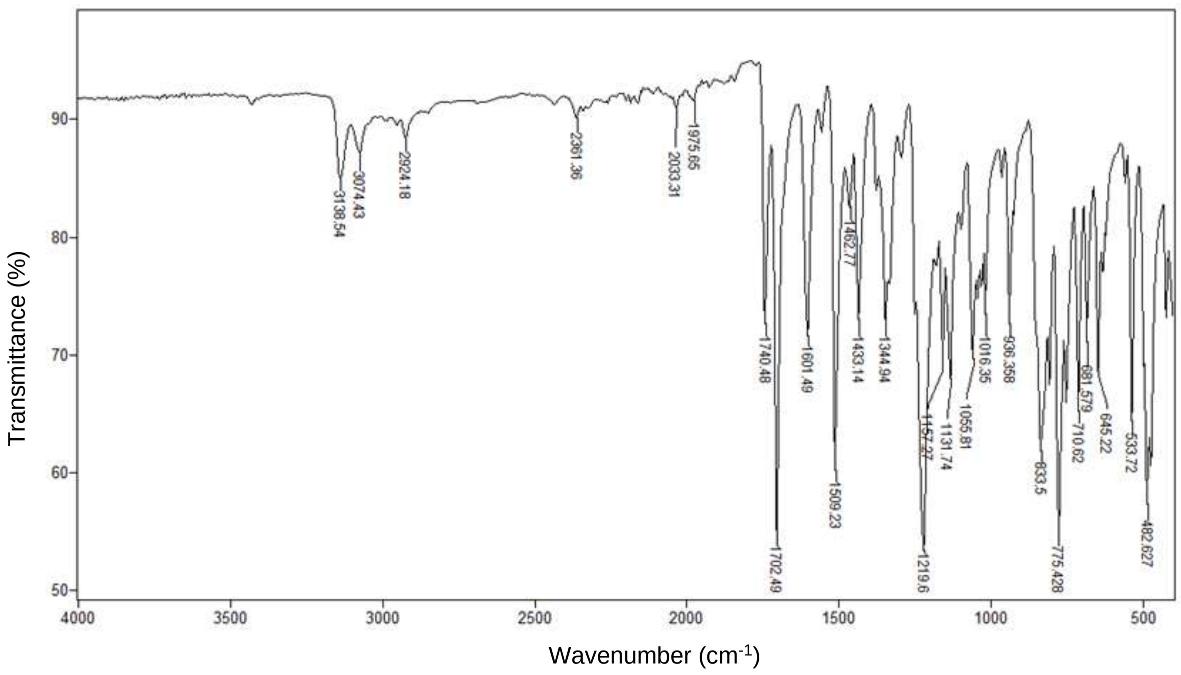

Fig. CP. IR spectrum (ATR) of 2,2-*bis*((1-(4-fluorobenzyl)-1*H*-1,2,3-triazol-4-yl)methyl)-1*H*-indene-1,3(2*H*)-dione (**55**).

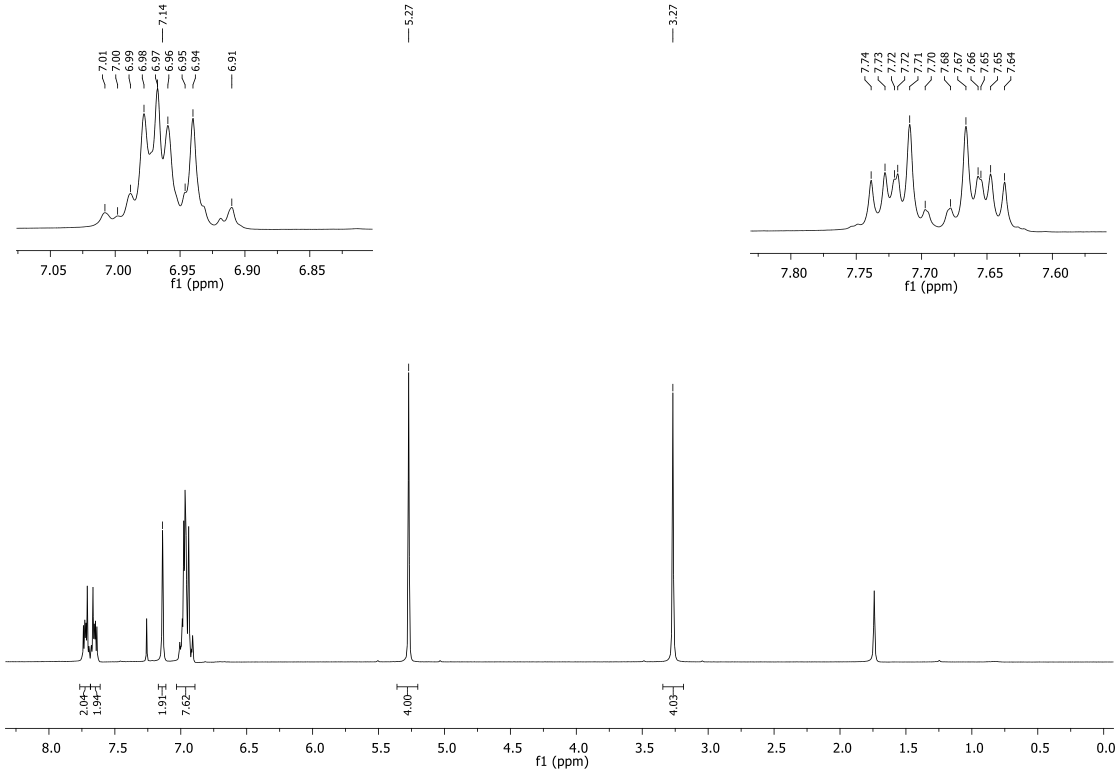

Fig. CQ. ^{1}H NMR spectrum (300 MHz, $CDCl_3$) of 2,2-*bis*((1-(4-fluorobenzyl)-1*H*-1,2,3-triazol-4-yl)methyl)-1*H*-indene-1,3(2*H*)-dione (**55**).

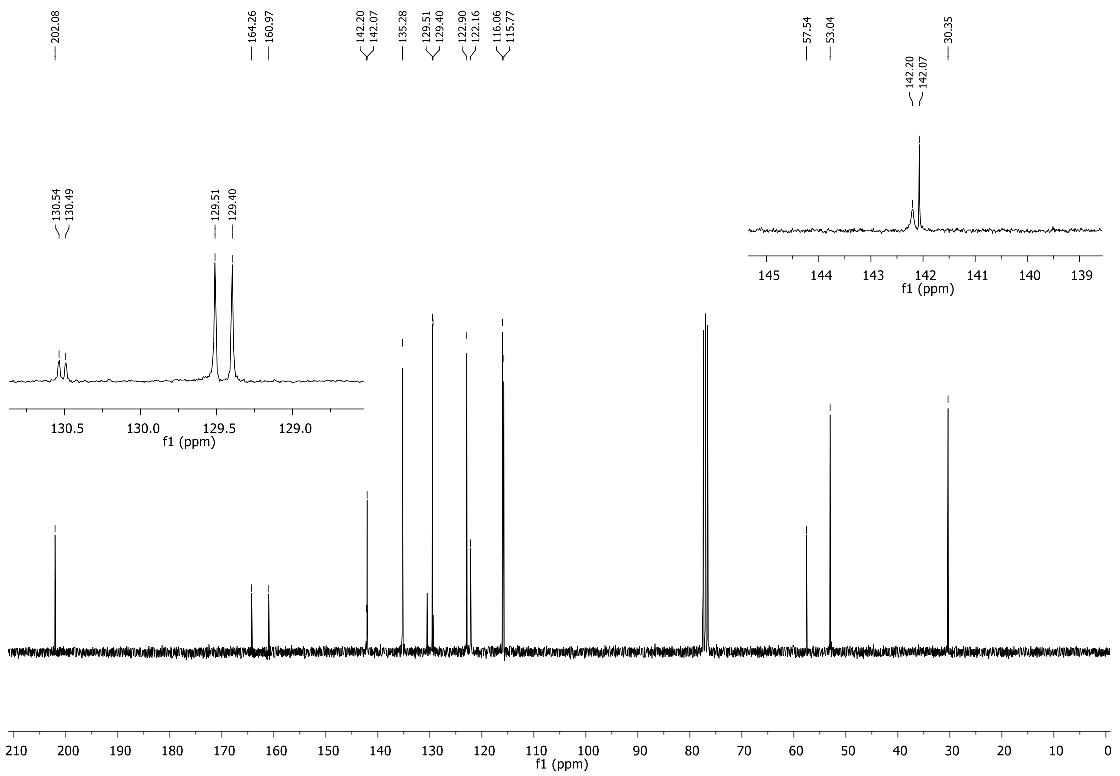

Fig. CR. ^{13}C NMR spectrum (75 MHz, $CDCl_3$) of 2,2-*bis*((1-(4-fluorobenzyl)-1*H*-1,2,3-triazol-4-yl)methyl)-1*H*-indene-1,3(2*H*)-dione (**55**).

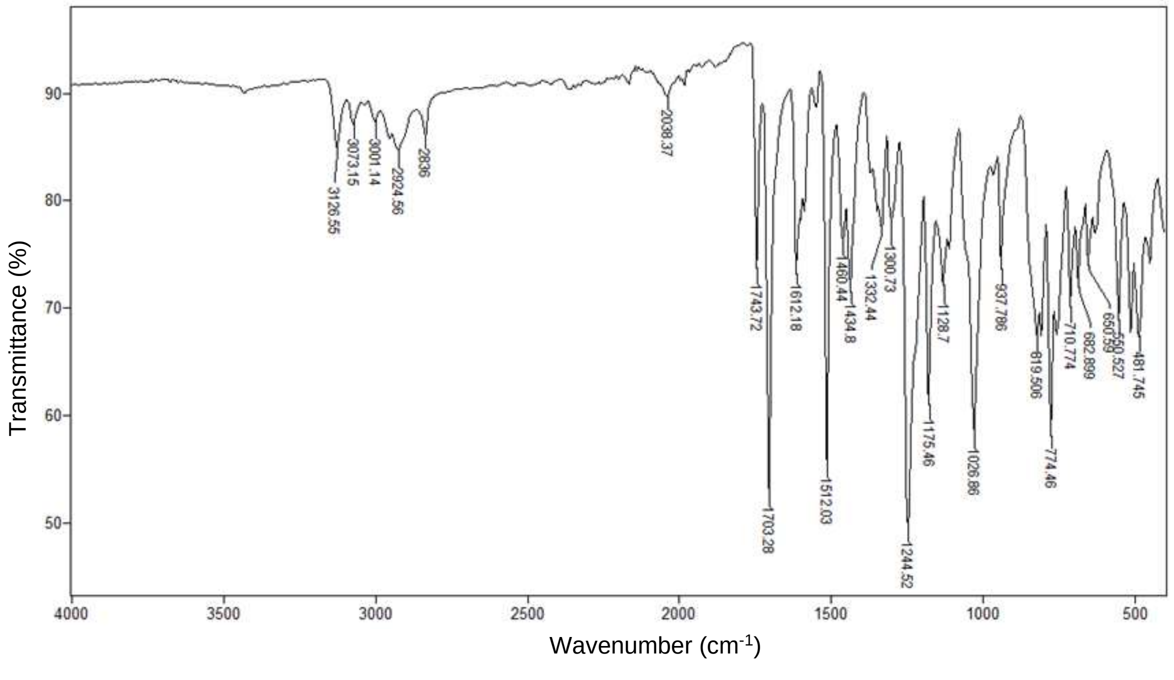

Fig. CS. IR spectrum (ATR) of 2,2-*bis*((1-(4-methoxybenzyl)-1*H*-1,2,3-triazol-4-yl)methyl)-1*H*-indene-1,3(2*H*)-dione (**56**).

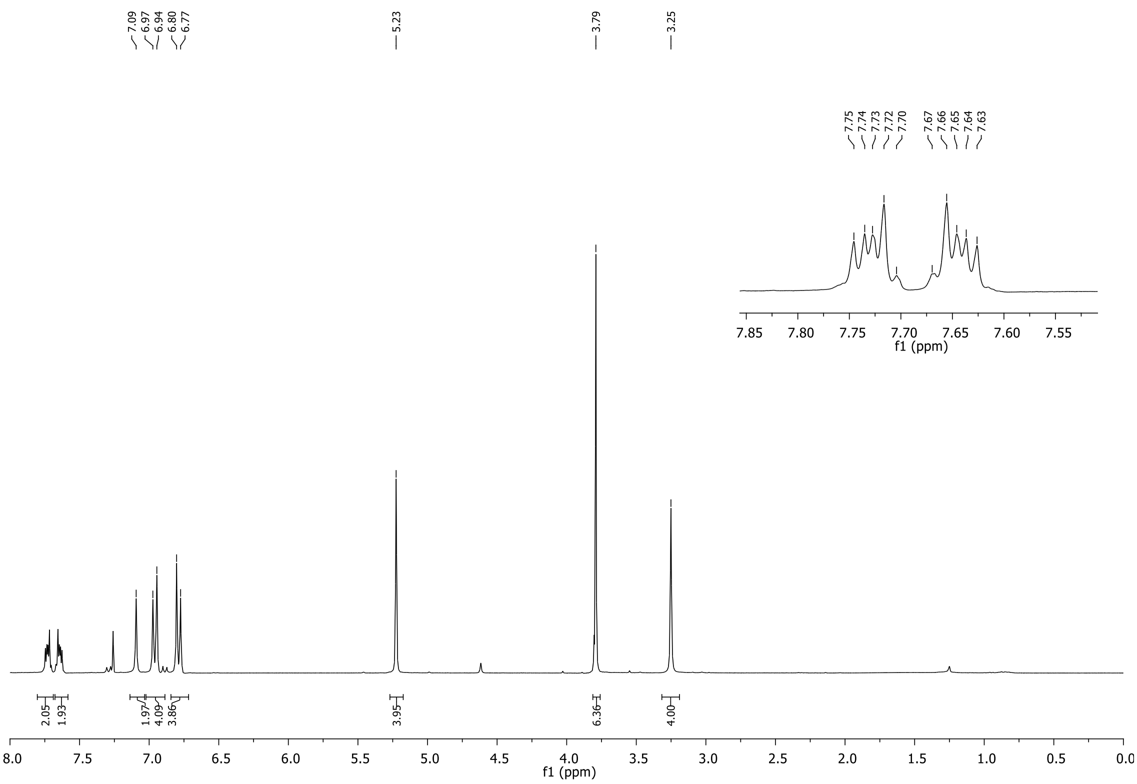

Fig. CT. ^{1}H NMR spectrum (300 MHz, $CDCl_3$) of 2,2-*bis*((1-(4-methoxybenzyl)-1*H*-1,2,3-triazol-4-yl)methyl)-1*H*-indene-1,3(2*H*)-dione (**56**).

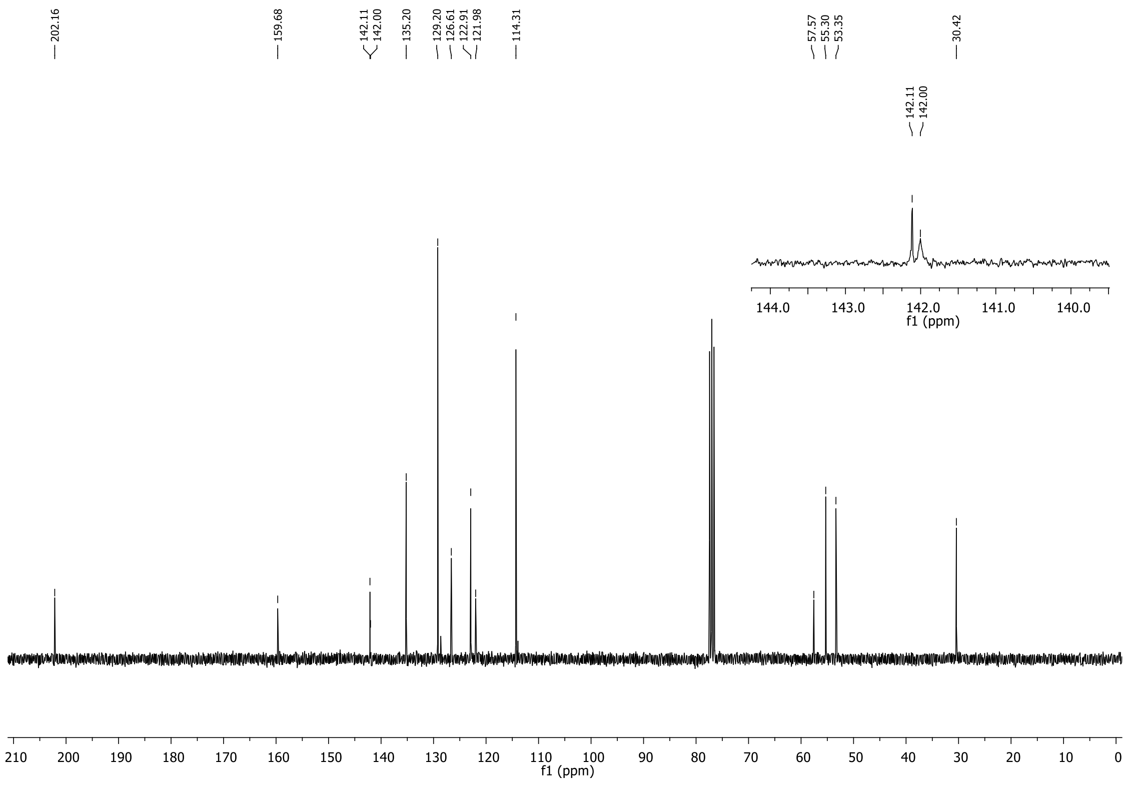

Fig. CU. ^{13}C NMR spectrum (75 MHz, $CDCl_3$) of 2,2-*bis*((1-(4-methoxybenzyl)-1*H*-1,2,3-triazol-4-yl)methyl)-1*H*-indene-1,3(2*H*)-dione (**56**).

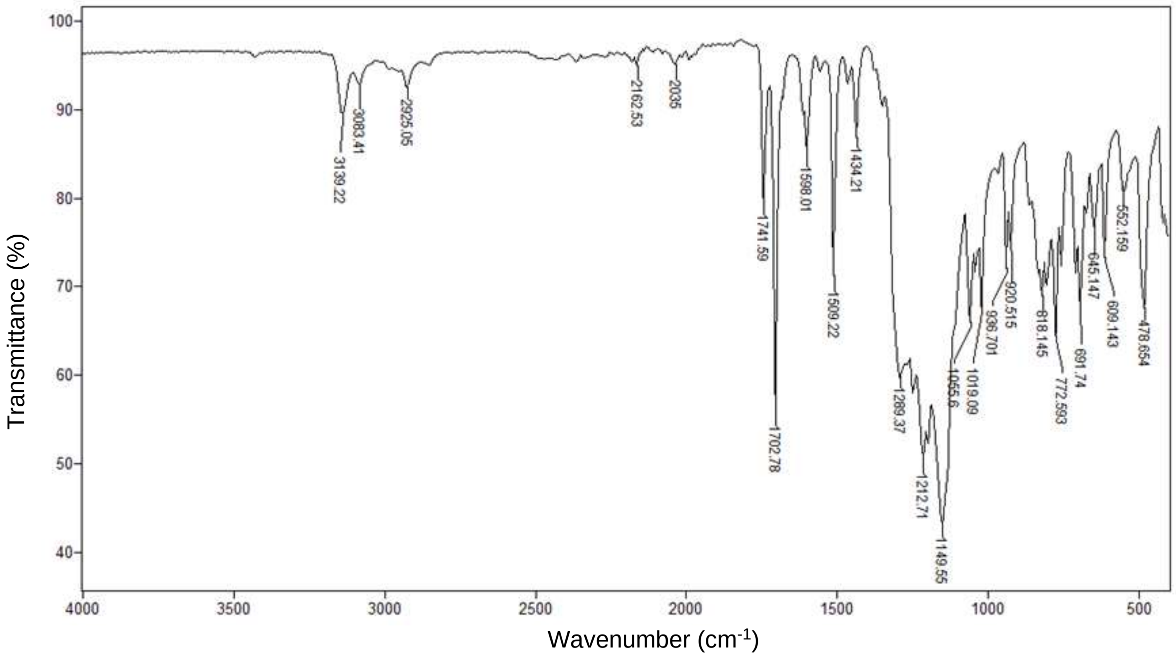

Fig. CV. IR spectrum (ATR) of 2,2-*bis*((1-(4-(trifluoromethoxy)benzyl)-1*H*-1,2,3-triazol-4-yl)methyl)-1*H*-indene-1,3(2*H*)-dione (**57**).

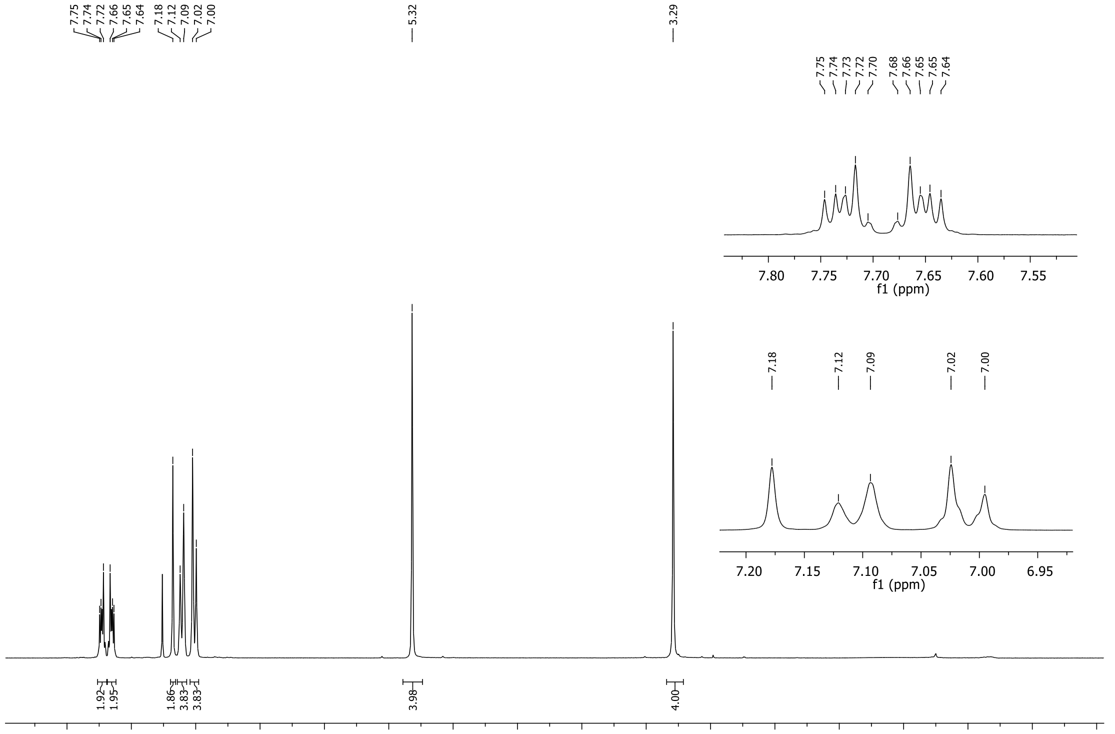

Fig. CX. ^{1}H NMR spectrum (300 MHz, $CDCl_3$) of 2,2-*bis*((1-(4-(trifluoromethoxy)benzyl)-1*H*-1,2,3-triazol-4-yl)methyl)-1*H*-indene-1,3(2*H*)-dione (**57**).

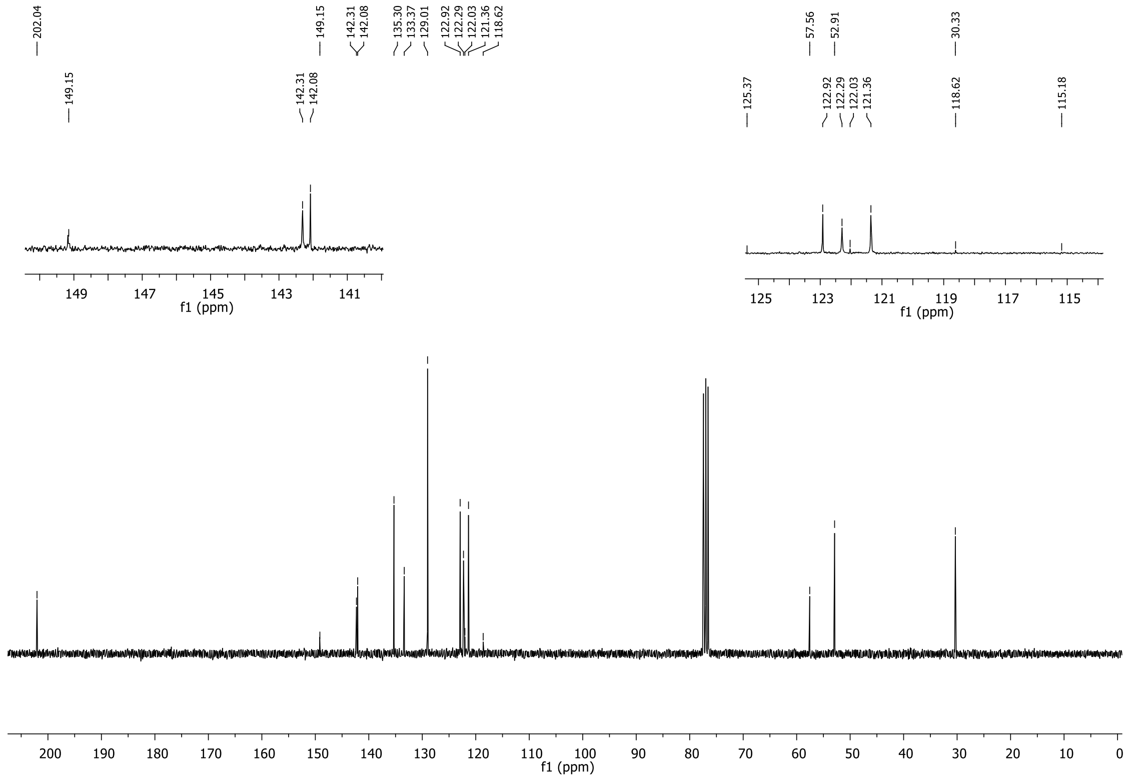

Fig. CW. ^{13}C NMR spectrum (75 MHz, $CDCl_3$) of 2,2-*bis*((1-(4-(trifluoromethoxy)benzyl)-1*H*-1,2,3-triazol-4-yl)methyl)-1*H*-indene-1,3(2*H*)-dione (**57**).

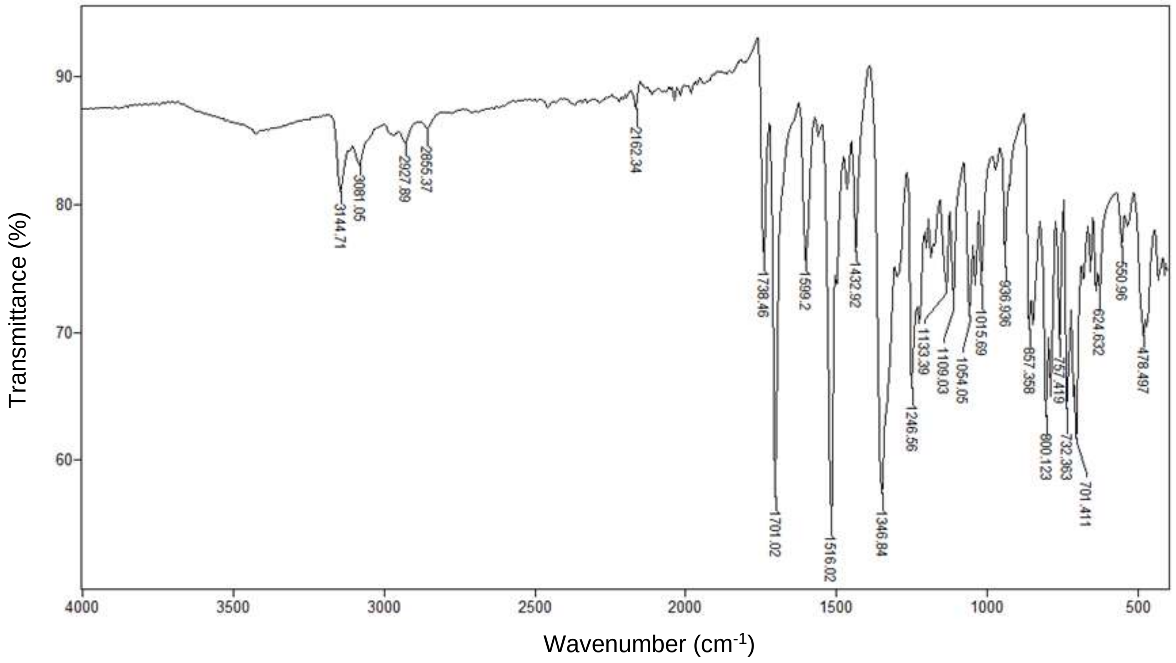

Fig. CY. IR spectrum (ATR) of 2,2-*bis*((1-(4-nitrobenzyl)-1*H*-1,2,3-triazol-4-yl)methyl)-1*H*-indene-1,3(2*H*)-dione (**58**).

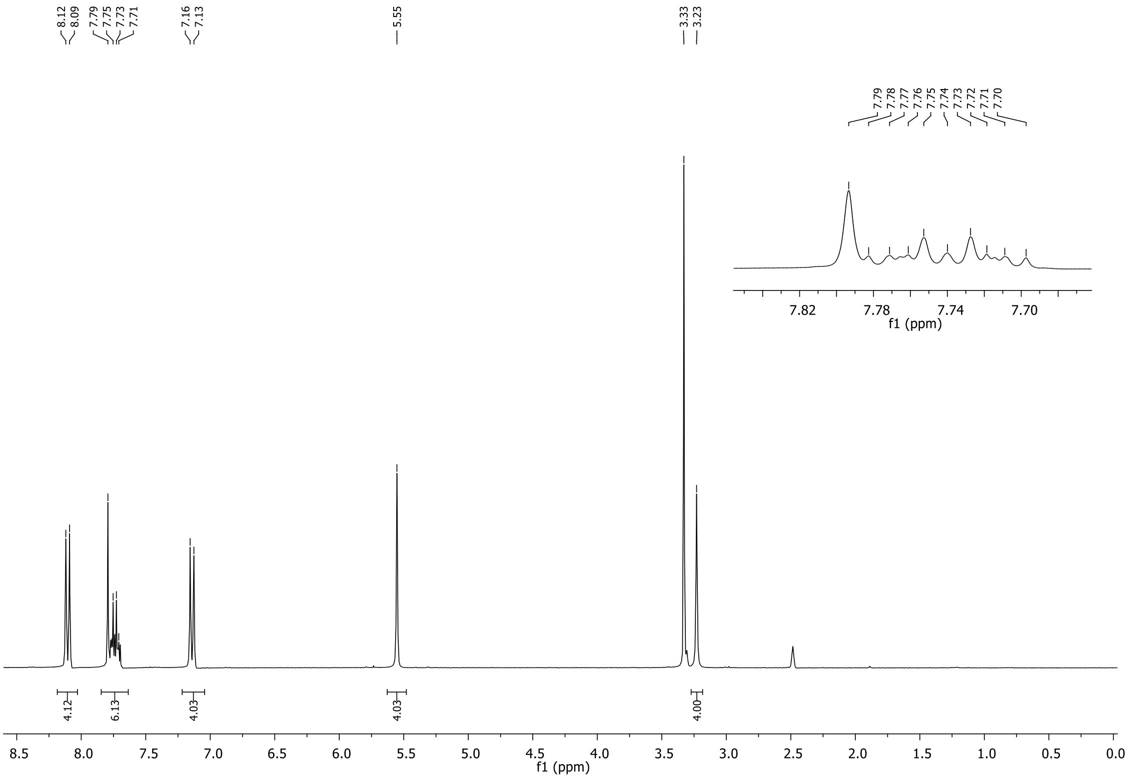

Fig. CZ. ^{1}H NMR spectrum (300 MHz, DMSO-d_6) of 2,2-*bis*((1-(4-nitrobenzyl)-1*H*-1,2,3-triazol-4-yl)methyl)-1*H*-indene-1,3(2*H*)-dione (**58**).

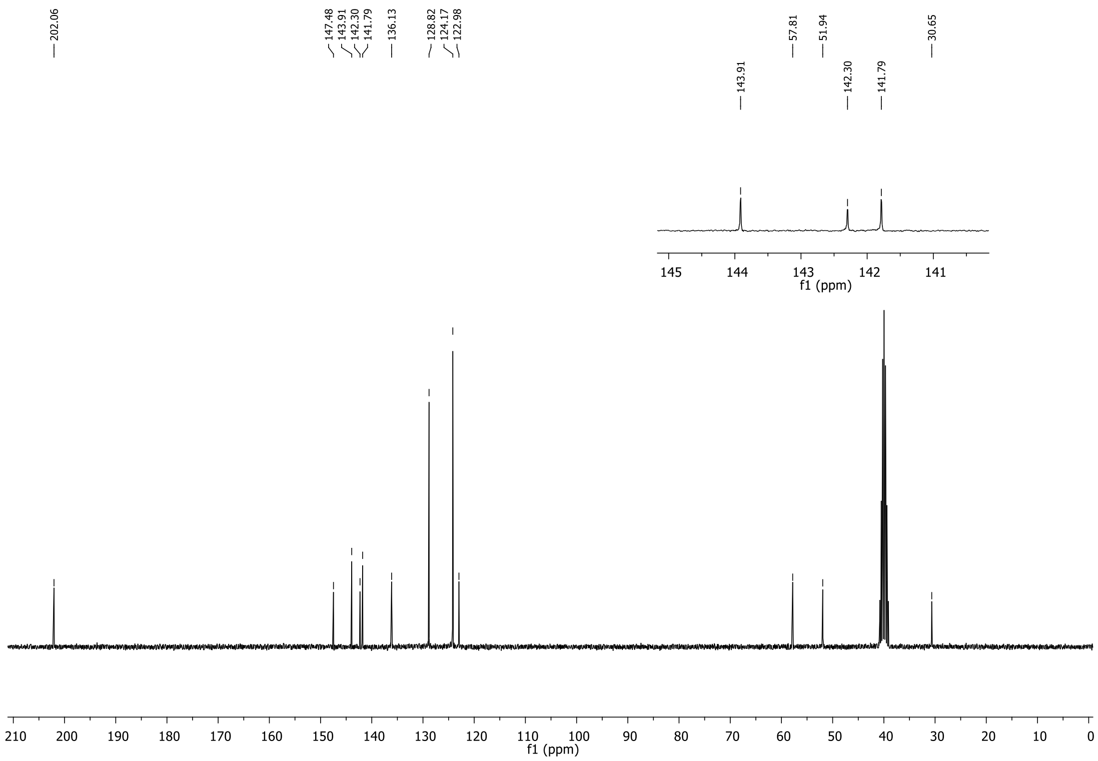

Fig. DA. ^{13}C NMR spectrum (75 MHz, DMSO-d_6) of 2,2-*bis*((1-(4-nitrobenzyl)-1*H*-1,2,3-triazol-4-yl)methyl)-1*H*-indene-1,3(2*H*)-dione (**58**).

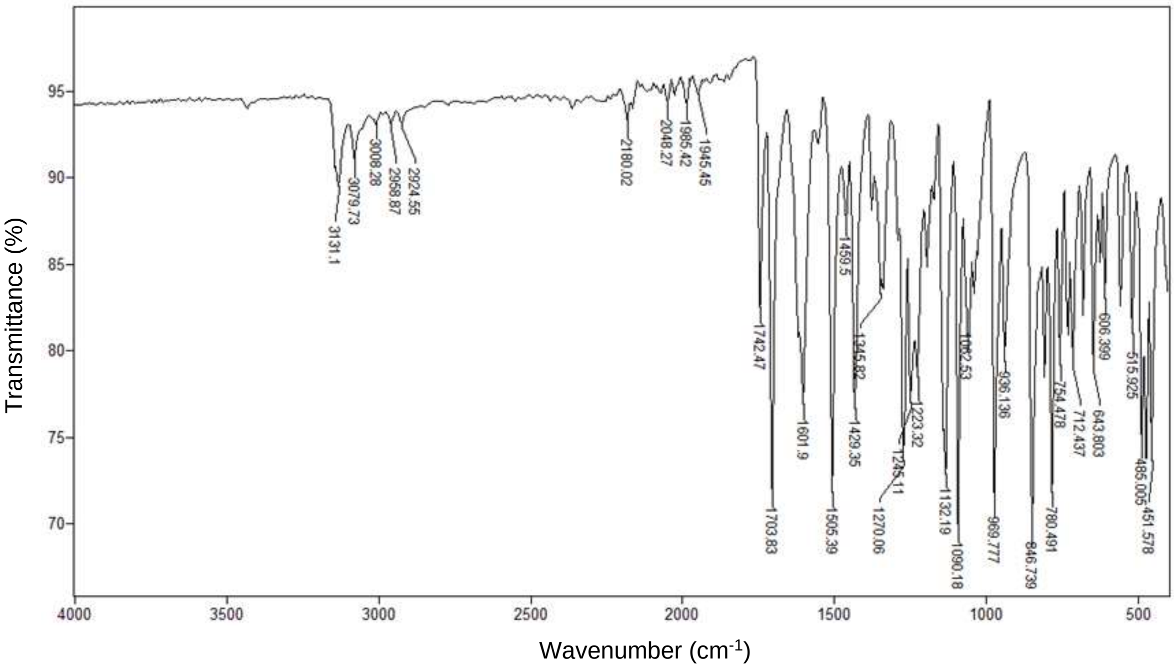

Fig. DB. IR spectrum (ATR) of 2,2-*bis*((1-(2,4-difluorobenzyl)-1*H*-1,2,3-triazol-4-yl)methyl)-1*H*-indene-1,3(2*H*)-dione (**59**).

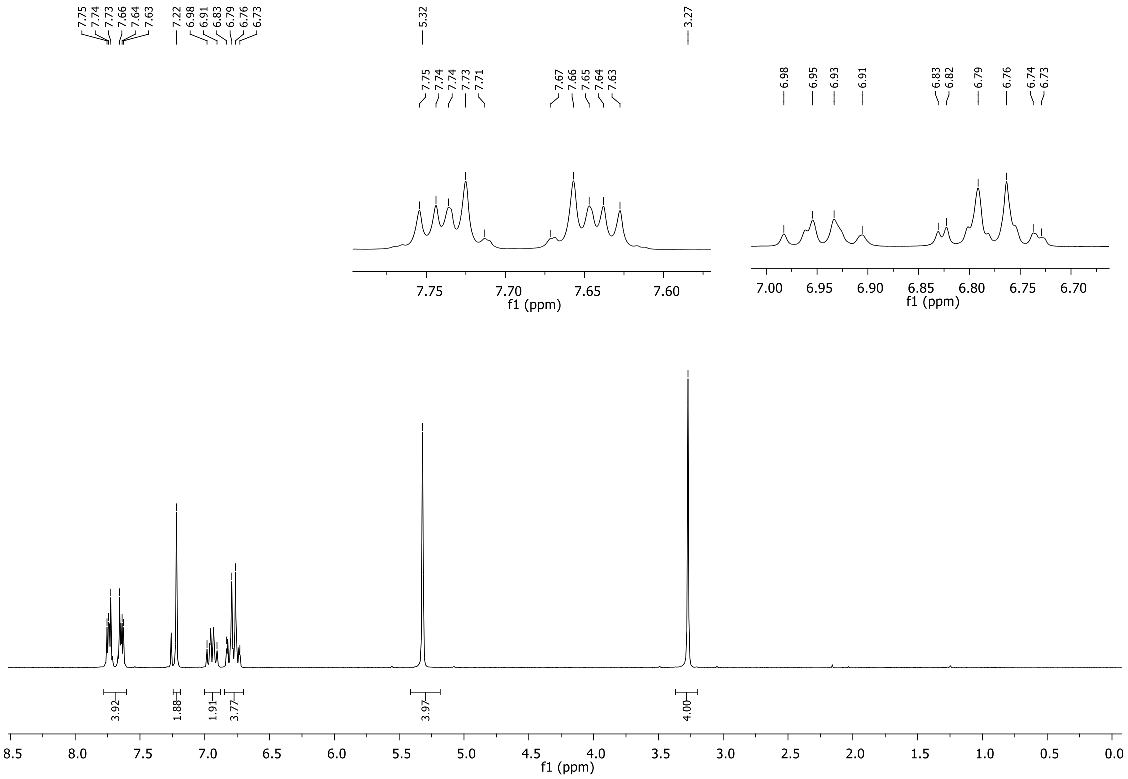

Fig. DC. ^{1}H NMR spectrum (300 MHz, $CDCl_3$) of 2,2-*bis*((1-(2,4-difluorobenzyl)-1*H*-1,2,3-triazol-4-yl)methyl)-1*H*-indene-1,3(2*H*)-dione (**59**).

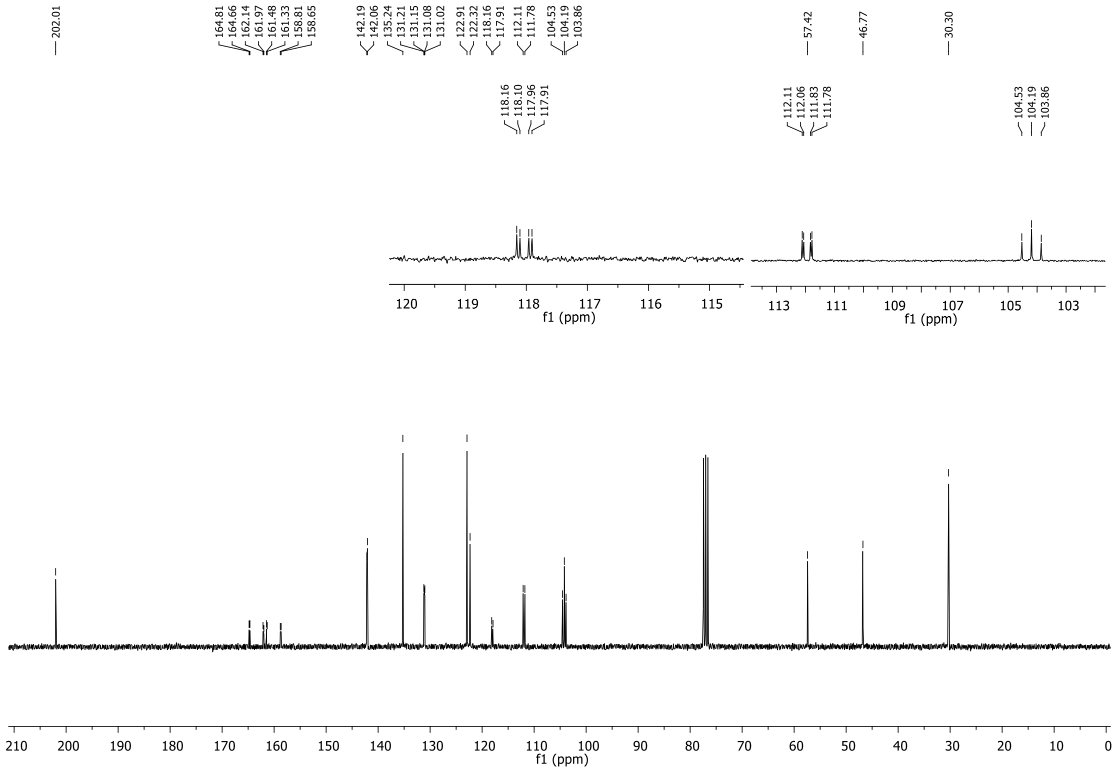

Fig. DD. ^{13}C NMR spectrum (75 MHz, $CDCl_3$) of 2,2-*bis*((1-(2,4-difluorobenzyl)-1*H*-1,2,3-triazol-4-yl)methyl)-1*H*-indene-1,3(2*H*)-dione (**59**).

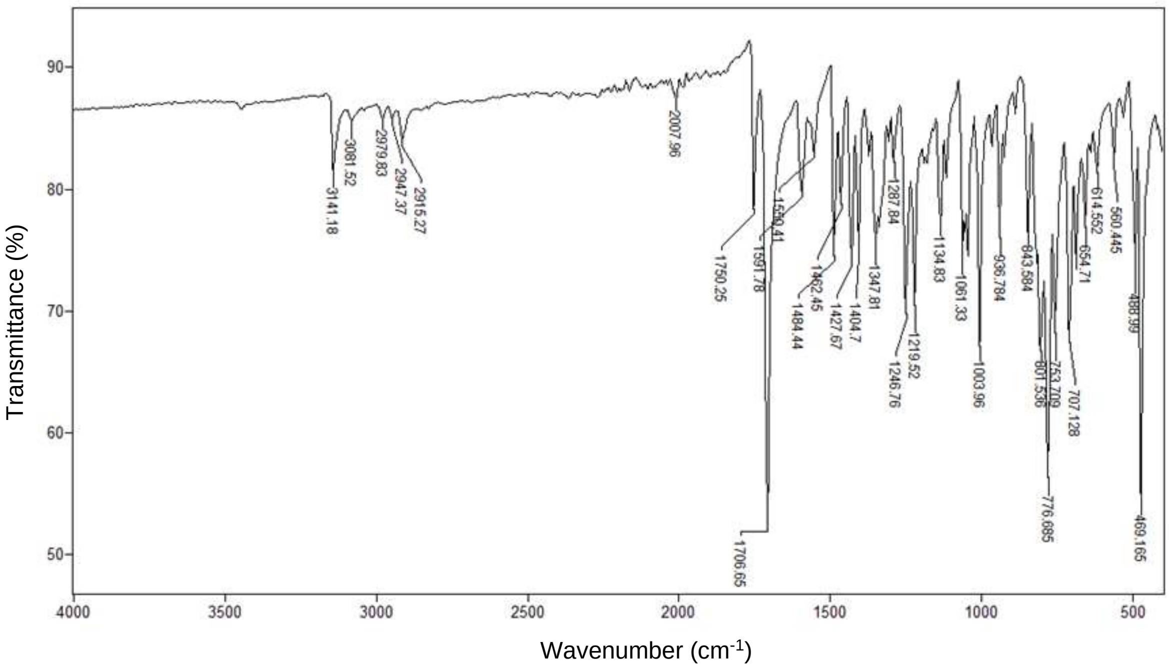

Fig. DE. IR spectrum (ATR) of 2,2-*bis*((1-(4-iodobenzyl)-1*H*-1,2,3-triazol-4-yl)methyl)-1*H*-indene-1,3(2*H*)-dione (**60**).

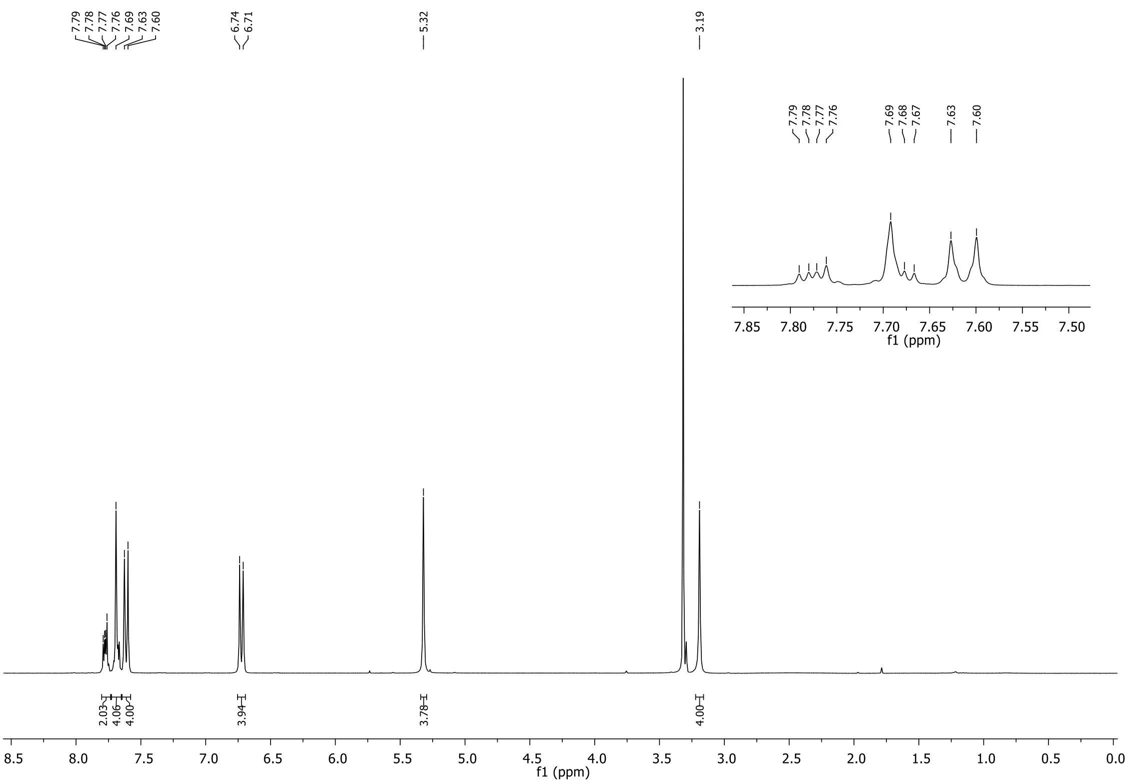

Fig. DF. ^{1}H NMR spectrum (300 MHz, DMSO-d_6) of 2,2-*bis*((1-(4-iodobenzyl)-1*H*-1,2,3-triazol-4-yl)methyl)-1*H*-indene-1,3(2*H*)-dione (**60**).

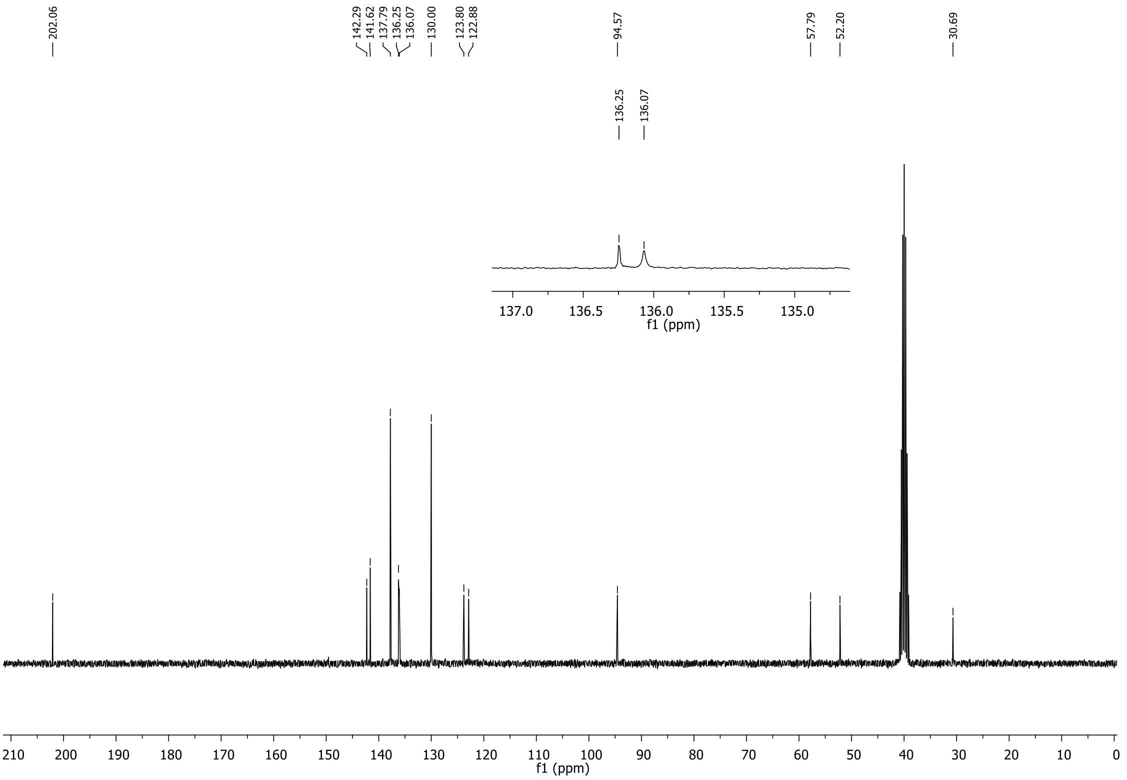

Fig. DG. ^{13}C NMR spectrum (75 MHz, DMSO-d_6) of 2,2-*bis*((1-(4-iodobenzyl)-1*H*-1,2,3-triazol-4-yl)methyl)-1*H*-indene-1,3(2*H*)-dione (**60**).

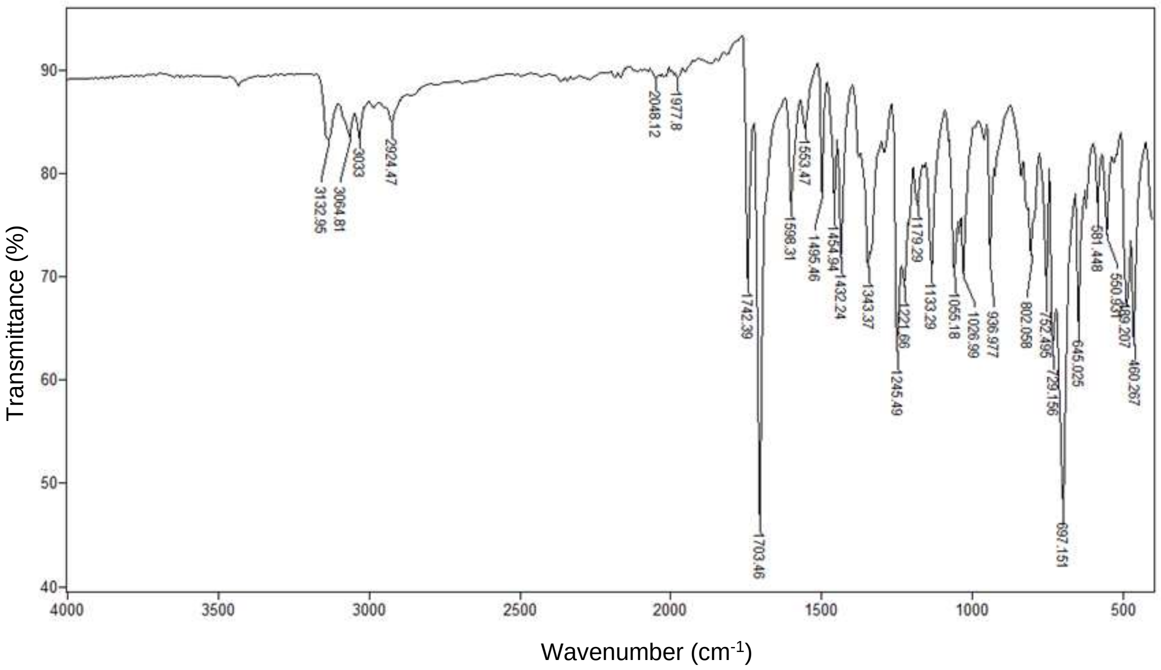

Fig. DH. IR spectrum (ATR) of 2,2-*bis*((1-(2-benzyl-1*H*-1,2,3-triazol-4-yl)methyl)-1*H*-indene-1,3(2*H*)-dione (**61**).

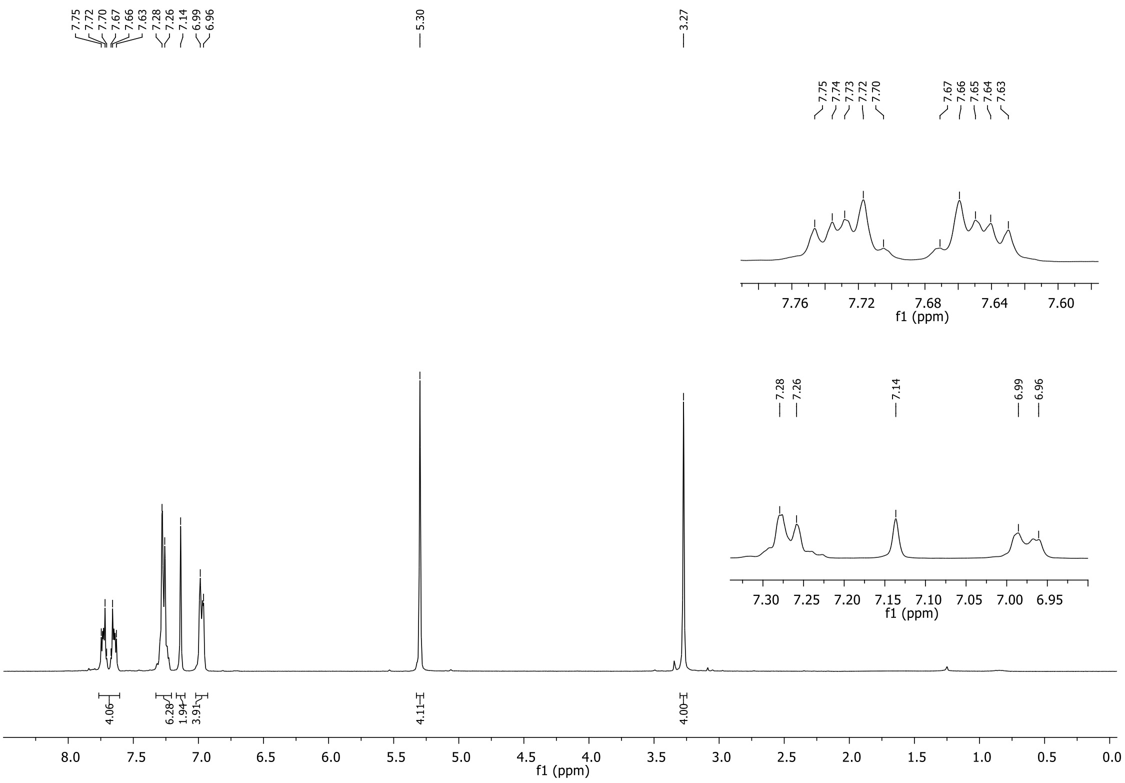

Fig. DI. ^{1}H NMR spectrum (300 MHz, $CDCl_3$) of 2,2-*bis*((1-(2-benzyl-1*H*-1,2,3-triazol-4-yl)methyl)-1*H*-indene-1,3(2*H*)-dione (**61**).

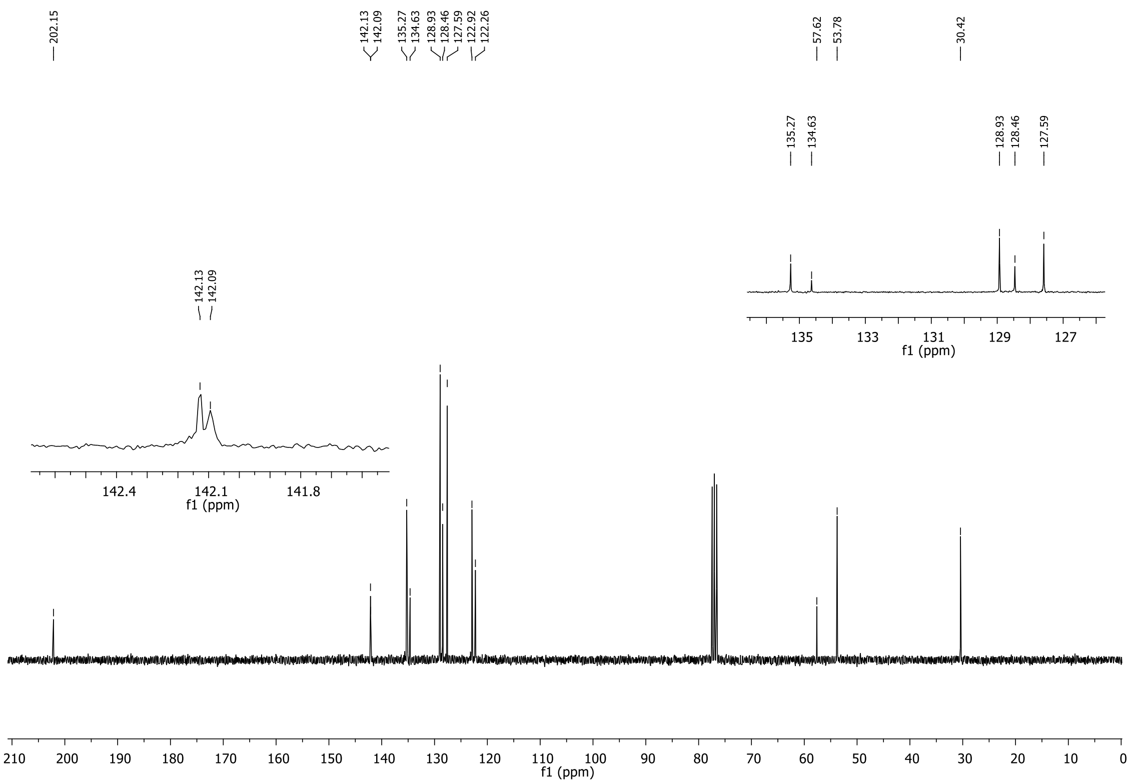

Fig. DJ. ^{13}C NMR spectrum (75 MHz, $CDCl_3$) of 2,2-*bis*((1-(2-benzyl-1*H*-1,2,3-triazol-4-yl)methyl)-1*H*-indene-1,3(2*H*)-dione (**61**).

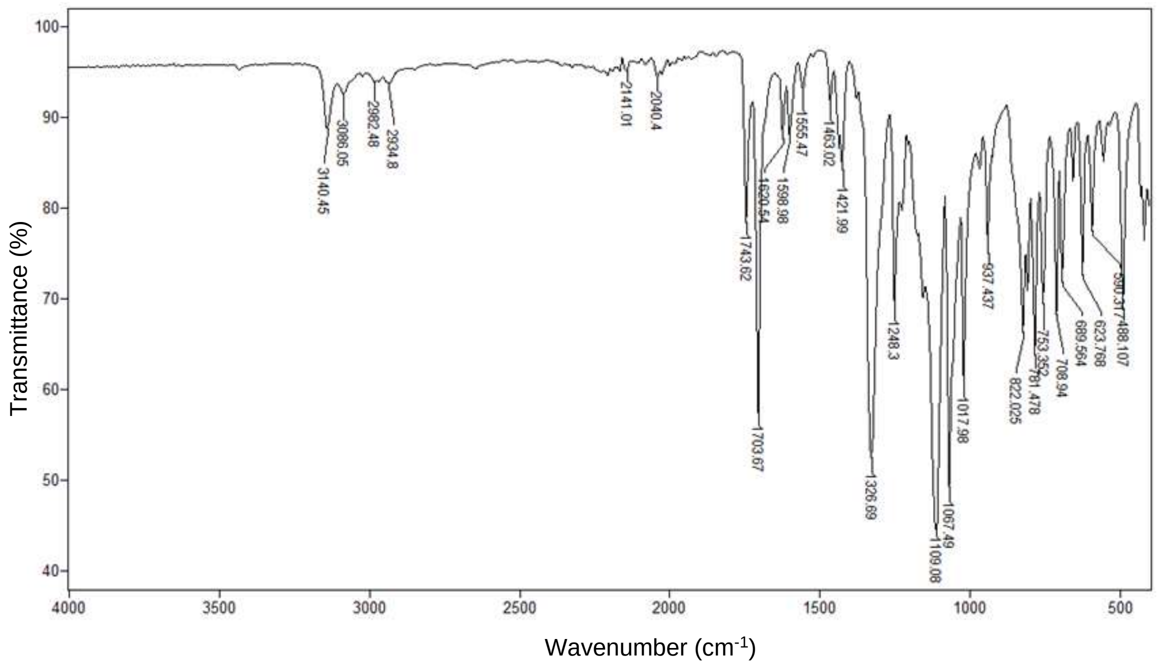

Fig. DK. IR spectrum (ATR) of 2,2-*bis*((1-(4-(trifluoromethyl)benzyl)-1*H*-1,2,3-triazol-4-yl)methyl)-1*H*-indene-1,3(2*H*)-dione (**62**).

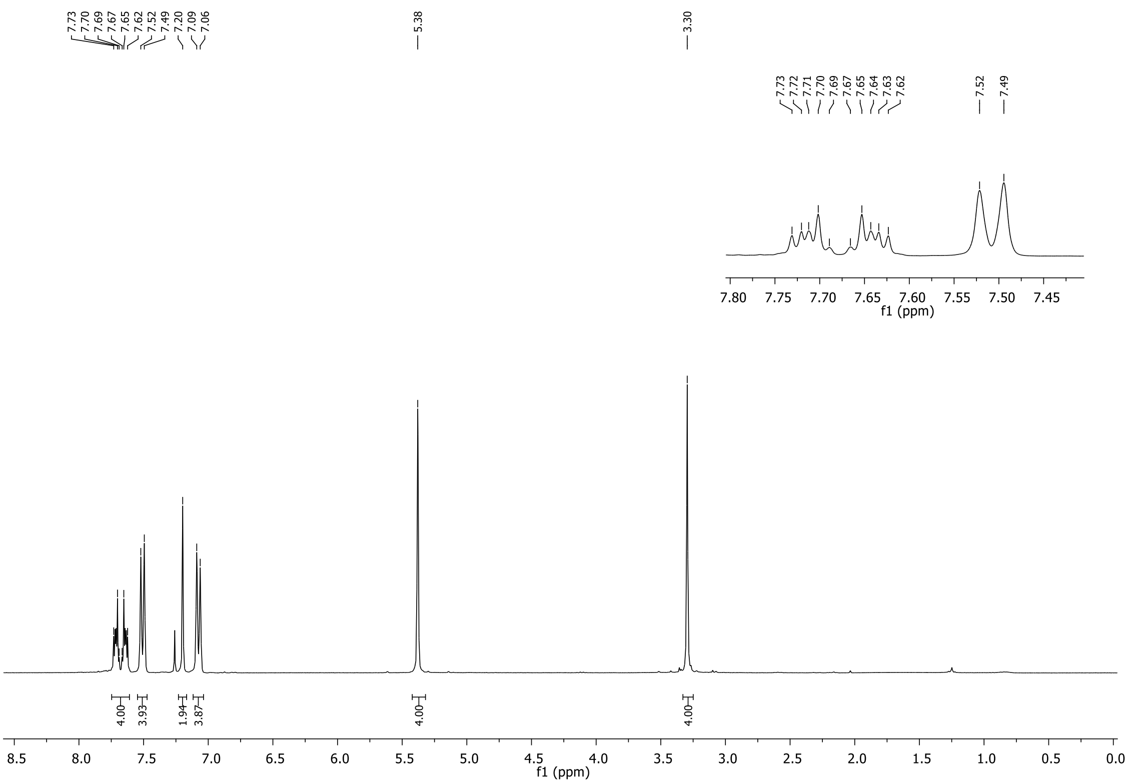

Fig. DL. ^{1}H NMR spectrum (300 MHz, $CDCl_3$) of 2,2-*bis*((1-(4-(trifluoromethyl)benzyl)-1*H*-1,2,3-triazol-4-yl)methyl)-1*H*-indene-1,3(2*H*)-dione (**62**).

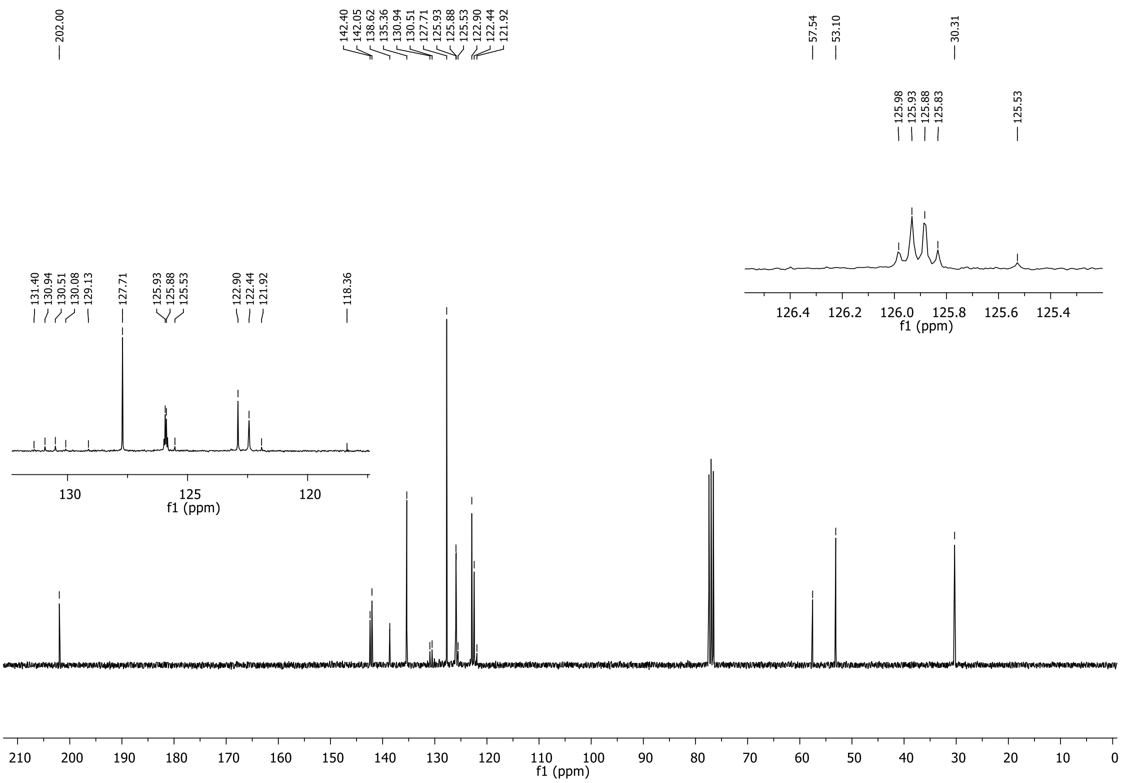

Fig. DM. ^{13}C NMR spectrum (75 MHz, $CDCl_3$) of 2,2-*bis*((1-(4-(trifluoromethyl)benzyl)-1*H*-1,2,3-triazol-4-yl)methyl)-1*H*-indene-1,3(2*H*)-dione (**62**).

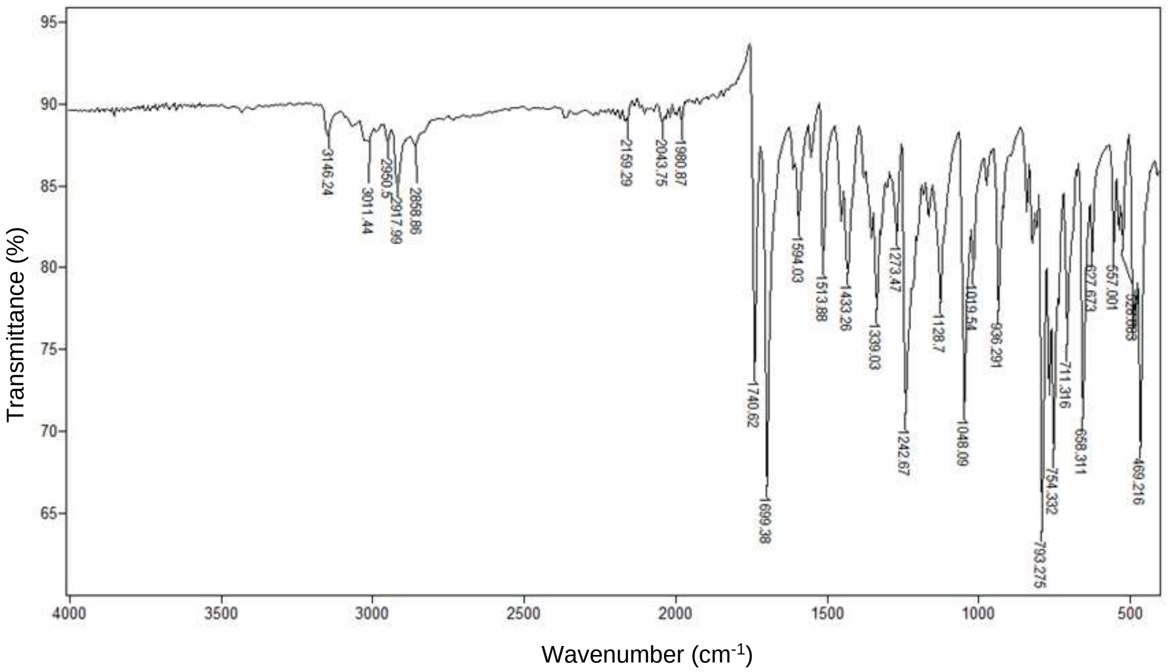

Fig. DN. IR spectrum (ATR) of 2,2-*bis*((1-(4-methylbenzyl)-1*H*-1,2,3-triazol-4-yl)methyl)-1*H*-indene-1,3(2*H*)-dione (**63**).

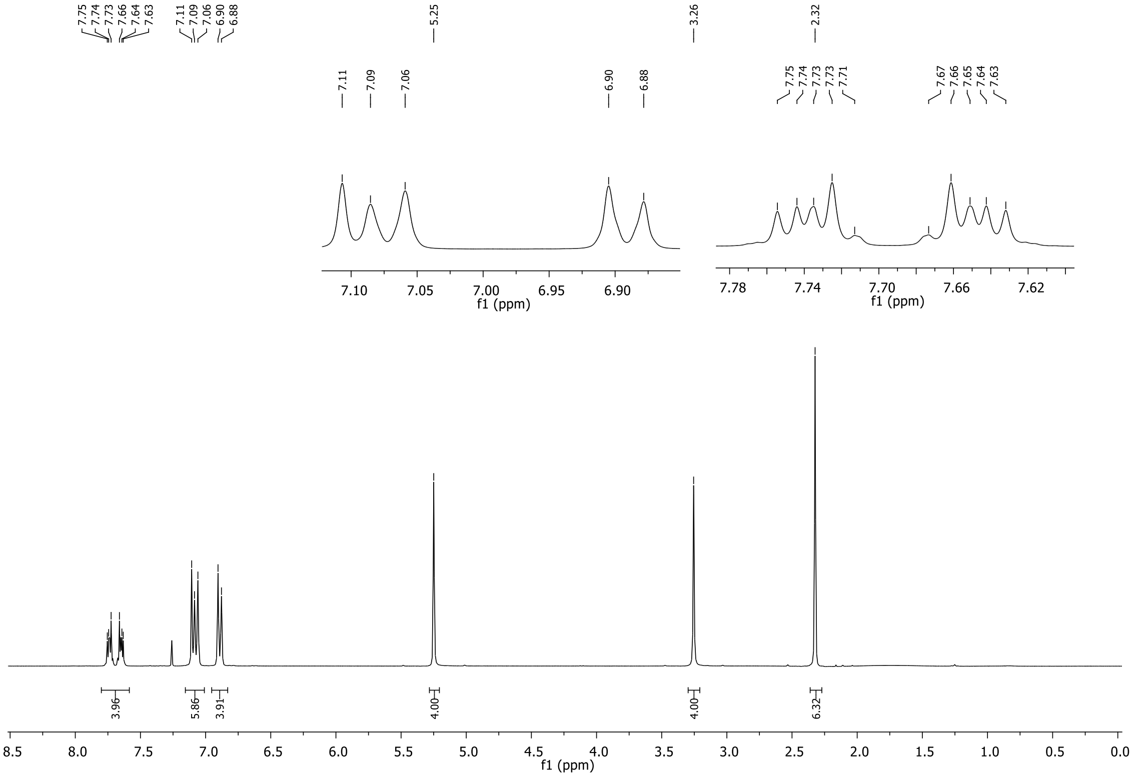

Fig. DO. ^{1}H NMR spectrum (300 MHz, $CDCl_3$) of 2,2-*bis*((1-(4-methylbenzyl)-1*H*-1,2,3-triazol-4-yl)methyl)-1*H*-indene-1,3(2*H*)-dione (**63**).

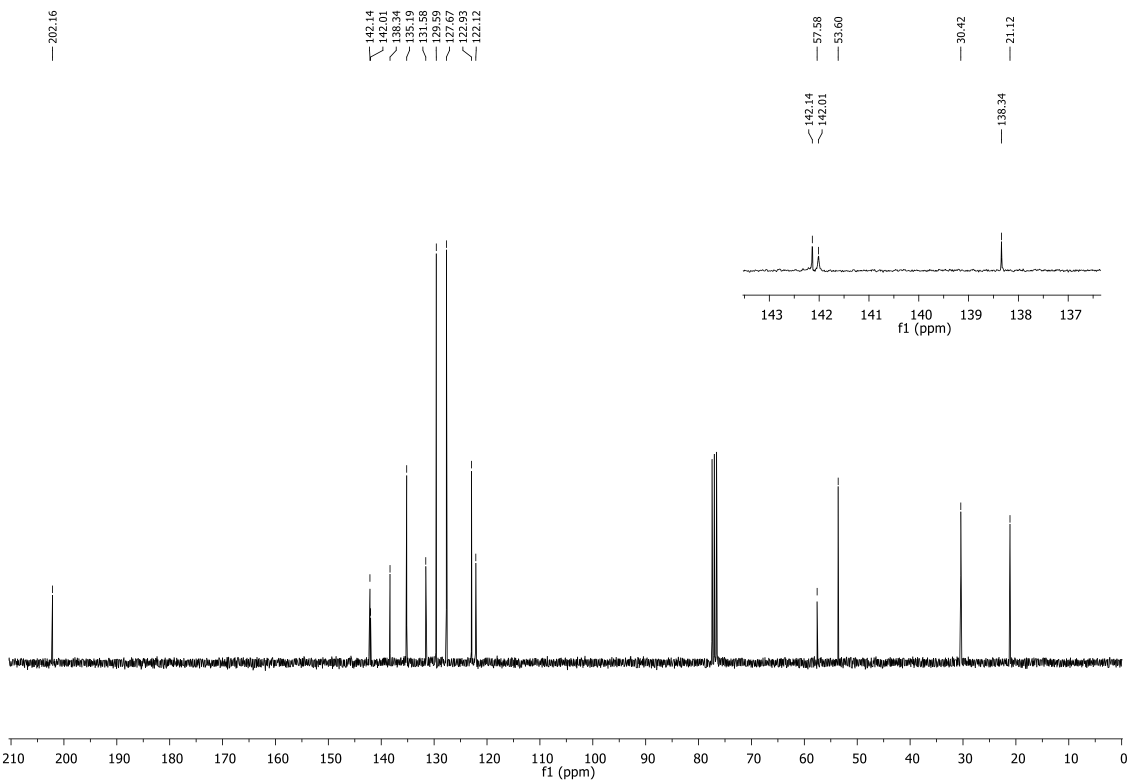

Fig. DP. ^{13}C NMR spectrum (75 MHz, $CDCl_3$) of 2,2-*bis*((1-(4-methylbenzyl)-1*H*-1,2,3-triazol-4-yl)methyl)-1*H*-indene-1,3(2*H*)-dione (**63**).
