# Supplementary figures and images for: Discovery of novel West Nile Virus protease inhibitor based on isobenzonafuranone and triazolic derivatives of eugenol and indan-1,3-dione scaffolds
Source: PLoS One. 2019 Sep 26;14(9):e0223017. doi: 10.1371/journal.pone.0223017 (PMC6762200; doi:10.1371/journal.pone.0223017)

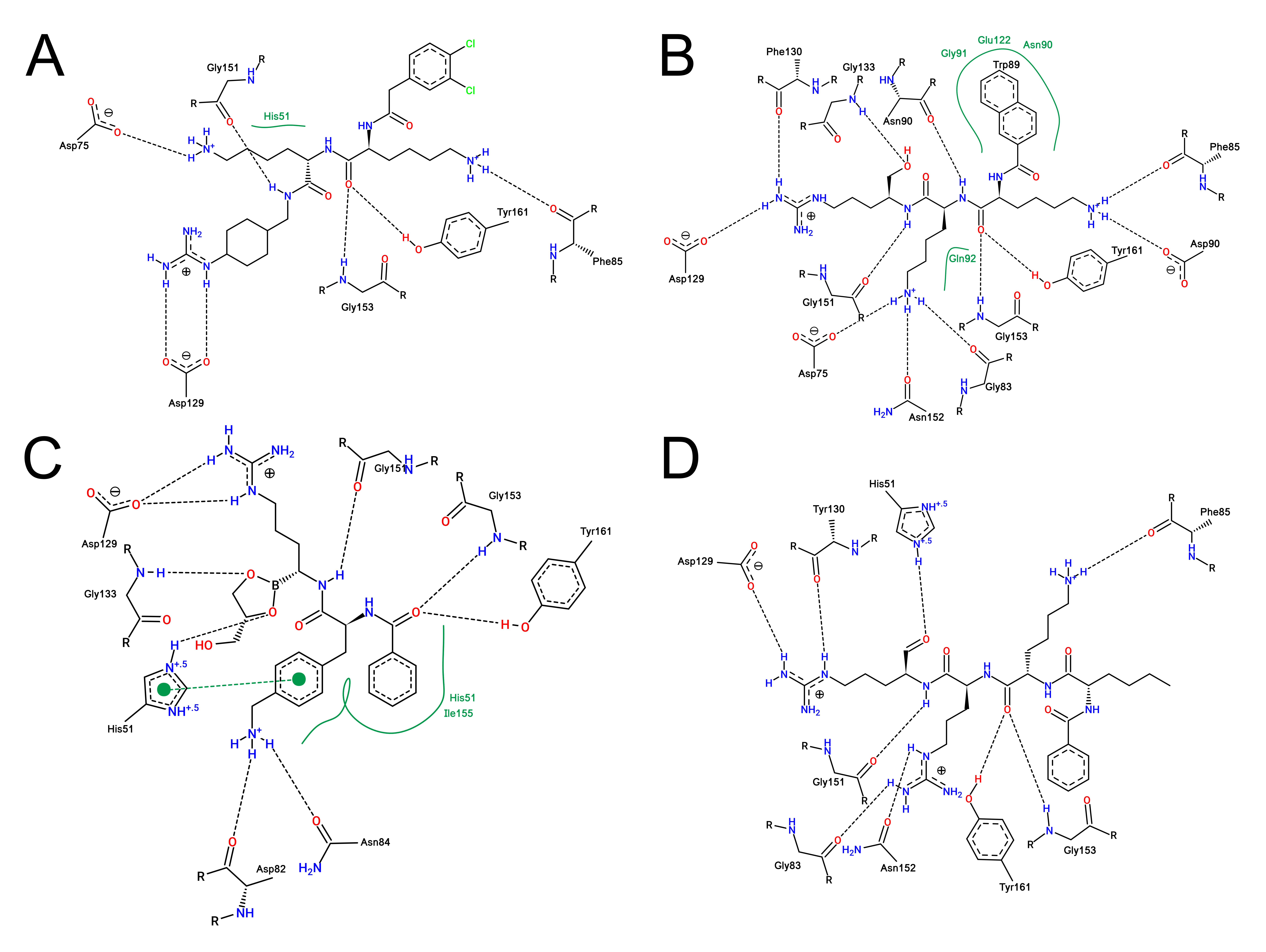

Supplement: S1 Fig — A. 2YOL; B. 3E90; C. 5IDK; and D. 2FP7. (TIFF) [file pone.0223017.s002.tiff]
